# Panoramic Visualization of Circulating MicroRNAs Across Neurodegenerative Diseases in Humans

**DOI:** 10.1007/s12035-019-1615-1

**Published:** 2019-04-29

**Authors:** Samuel Brennan, Matthew Keon, Bing Liu, Zheng Su, Nitin K. Saksena

**Affiliations:** Neurodegenerative Disease section, Iggy Get Out, 19a Boundary Street, Darlinghurst NSW 2010, Sydney, Australia

**Keywords:** MiRNA, Biomarker, Neurodegenerative disease, Neuropathology

## Abstract

Neurodegenerative diseases (NDs) such as Alzheimer’s disease (AD), Parkinson’s disease (PD), multiple sclerosis (MS), amyotrophic lateral sclerosis (ALS), and dementia pose one of the greatest health challenges this century. Although these NDs have been looked at as single entities, the underlying molecular mechanisms have never been collectively visualized to date. With the advent of high-throughput genomic and proteomic technologies, we now have the opportunity to visualize these diseases in a whole new perspective, which will provide a clear understanding of the primary and secondary events vital in achieving the final resolution of these diseases guiding us to new treatment strategies to possibly treat these diseases together. We created a knowledge base of all microRNAs known to be differentially expressed in various body fluids of ND patients. We then used several bioinformatic methods to understand the functional intersections and differences between AD, PD, ALS, and MS. These results provide a unique panoramic view of possible functional intersections between AD, PD, MS, and ALS at the level of microRNA and their cognate genes and pathways, along with the entities that unify and separate them. While the microRNA signatures were apparent for each ND, the unique observation in our study was that hsa-miR-30b-5p overlapped between all four NDS, and has significant functional roles described across NDs. Furthermore, our results also show the evidence of functional convergence of miRNAs which was associated with the regulation of their cognate genes represented in pathways that included fatty acid synthesis and metabolism, ECM receptor interactions, prion diseases, and several signaling pathways critical to neuron differentiation and survival, underpinning their relevance in NDs. Envisioning this group of NDs together has allowed us to propose new ways of utilizing circulating miRNAs as biomarkers  and in visualizing diverse NDs more holistically . The critical molecular insights gained through the discovery of ND-associated miRNAs, overlapping miRNAs, and the functional convergence of microRNAs on vital pathways strongly implicated in neurodegenerative processes can prove immensely valuable in the identifying new generation of biomarkers, along with the development of miRNAs into therapeutics.

**Electronic supplementary material:**

The online version of this article (10.1007/s12035-019-1615-1) contains supplementary material, which is available to authorized users.

## Background

Neurodegenerative diseases (NDs) such as Alzheimer’s disease (AD), Parkinson’s disease (PD), multiple sclerosis (MS), amyotrophic lateral sclerosis (ALS), and dementia pose one of the greatest health challenges this century [1].

NDs are a set of devastating, progressive diseases defined by neuron loss or irreversible dysfunction. Sporadic NDs are those with poorly defined causes or underlying pathophysiology, or those that show extreme variability in disease presentation and progression. For the purpose of this study, we have focused on four major neurodegenerative diseases with sporadic presentations: ALS, MS, AD, and PD because of the availability of considerable genomic knowledge across these four diseases. Although these four NDs fall under the broad umbrella of neurodegenerative diseases, other NDs such as frontotemporal dementia, Huntington’s disease (HD), spinocerebellar ataxia (SCA), and spinal muscular atrophy (SMA) could not be included in this analysis as it is beyond the scope of this study to cover all these diseases.

At present, the most important unmet need is for sensitive and accurate diagnostic techniques capable of early detection of these NDs, and there is a considerable lack of new generation genomic biomarkers to detect them and intervene early in order to improve clinical outcomes [2]. High-throughput genomic technologies coupled with innovative bioinformatic approaches provide us an opportunity for the development of new generation of diagnostic biomarkers that can provide early and effective diagnosis of NDs. More recently however, epigenetic elements of these diseases, the small non-coding RNAs known as microRNAs (miRNAs), have shown considerable promise in therapeutics and diagnostics in both NDs and cancer owing to their enrichment across body fluids (plasma, serum, CSF, urine, etc.) during disease. MiRNAs are approximately 22 nt in length and they act on target mRNA strands at complementary sequences in the 3′ untranslated region (3′ UTR) of this mRNA. The miRNA carries an enzymatic complex known as the RNA-induced silencing complex (RISC) which is responsible for repressing translation from its target mRNA and eventually destroying the transcript [3].

MiRNA dysregulation has been observed more commonly in cancer, followed by neurodegenerative disorders such as AD, PD, MS, HD, ALS, and the neurological disorders, schizophrenia, autism, and epilepsy [4]. It is well recognized that these disorders are the culmination of diverse genetic and environmental factors. There is now considerable evidence implying that miRNAs are pathologically altered during the inexorable course of neurodegenerative diseases, and their dysregulation may be a contributing factor in causing neurodegeneration. Neuronal degradation and neuronal death are hallmarks of neurodegenerative disorders that unify all NDs, alongside apoptosis [5, 6], mitochondrial dysfunction [7, 8], and fatty acid metabolism [9, 10]. Additionally, abnormalities in metabolism, synapsis, and axonal transport have been associated with AD, PD, and frontotemporal dementia (FTD) [4]. The imprint of these pathways and others can be visualized in a genome-wide manner with the availability of high-throughput molecular biology techniques.

To date, > 70% of the annotated miRNA detected from the brain are known to play a vital role in several brain functions encompassing neural and synaptic development, in addition to dynamically engaging in regulating stress response genes [11, 12]. Although many of the miRNAs display cell-type or regional specificities in the brain [13, 14], the majority of neuronal miRNAs are distributed at higher levels in the soma, and lower levels in the distal dendrites, with only a fraction being enriched in the dendrites [15]. Because of this tight transcriptional regulation, the alteration of miRNA levels has an intrinsic relationship with not only neuro-embryogenesis, but also with neurological disorders that afflict humans [16, 17].

The current evidence gathered during the creation of this knowledge database suggests that the control of mRNA by the dysregulated miRNAs may be a critical factor in guiding neurodegenerative processes. In the absence of a curated and established database for miRNA in NDs, this study is the first to provide a functional roadmap of all the miRNAs currently known in the context of these four NDs. The rationale for this study stems from the juxtaposition and the proximity of the brain regions that are involved in the manifestation of these four NDs analyzed herein; in addition to increasing evidence suggesting the relevance of miRNAs in disease development, pathogenesis and their possible utility in diagnostics and therapeutics. Therefore, in the body of this unique research study, we have sought to identify and assemble all the known human miRNAs from all of the previous studies pertaining to, and encompassing all four NDs. We have created a knowledge database of all the known and relevant miRNAs originating from various body compartments that include patient body fluids (plasma, CSF, and serum), as well as circulating cells derived from these fluids. We have analyzed these miRNAs in the context of these diseases and provide a snapshot of miRNAs that overlap between these NDs and the ones which separate them, along with possible functional annotations of their cognate genes/pathways. Through the use of innovative omics-based panoramic visualization, the main goal of this study is to provide not only the current perspective on the role of the currently known miRNAs in neurodegeneration, but also shed light on how these miRNAs regulate gene expression during neurodegeneration, unveil common pathways which can be exploited in defining the utility of small RNAs in diagnostics, prognostics and therapeutics for all four diseases, and provide insights into how these small RNAs regulate gene machinery at the sub-genomic level during disease development. This simultaneous omics-based visualization of these NDs provides a new and panoramic interpretation of neurodegenerative processes at the sub-genomic levels, which will shed light on the current status and potential functions of these miRNAs in NDs, along with their utility in diagnostics, prognostics and therapeutics.

## Methods

### Knowledge Base Building

The knowledge base (shown in Supplementary Table 1) was constructed by performing multiple literature searches using various permutations and combinations of search terms encompassing AD, PD, ALS, or MS on the NCBI, Pubmed, and Google Scholar platforms. Our study is aimed at identifying body fluid-based biomarkers for fALS, sALS, PD, AD, and MS. The search terms used were “miRNA biomarker (AD, PD, ALS or MS)” on the NCBI, Pubmed, and Google Scholar platforms. Any report of differential miRNA expression in a body fluid or circulating cell/particle was included so long as the study was performed in human subjects. The studies included in the knowledge base extend as far back as 2011. The resulting knowledge base encompasses 599 reports of differential miRNA expression from 72 different studies. There were a total of 347 unique miRNA entries in the knowledge base and 253 miRNAs were recorded more than once. The miRNAs recorded more than once were either (i) corroborating evidence for the miRNA expression in a single ND, (ii) conflicting evidence for the miRNA expression in a single ND, or (iii) evidence of miRNA dysregulation across multiple NDs. All 347 miRNAs that were included in the knowledge database were deemed statistically significant by different studies in terms of *p* and false discovery rate (FDR) value (adj *P* value < 0.05, FDR < 0.05), and the fold change (> 2 or <− 2). All studies included in the construction of the knowledge database were chosen based on these criteria including that they were performed on humans with appropriately diagnosed diseased groups, and with the inclusion of appropriate control groups from healthy populations as comparators.

### Hierarchical Clustering (Heatmap) Analysis

Heatmaps are useful for identifying genes or miRNAs that are commonly regulated, or biological signatures associated with a particular condition (e.g., a disease or an environmental condition) along with the phylogenetic relationship each of these genes/miRNAs share between them. All heatmap analyses were performed using DIANA tools MirPath V3 [18]. DIANA tools TarBase was used as the method of identifying target mRNA of the dysregulated miRNAs that were input in each search, as this is the only database that includes levels of evidence (high throughput, low accuracy versus low throughput, high accuracy). Pathway union was selected (with a FDR cut-off set to 0.5) to look for any common pathways potentially impacted by miRNA dysregulation. This information was then displayed as a heatmap of miRNA against targeted pathway(s).

### Cytoscape Analysis

Cytoscape was selected to visualize interaction networks amongst gene sets, as well as visualizing the functions of these gene sets. The analysis was performed by identifying the top 100 target genes (according to the context score for each gene) of the miRNAs of interest using TargetScan [19], as TargetScan results are downloadable and more easily imported into other analytical softwares. These mRNA targets were then merged into a single list and all duplicates were removed. This list was then input into Cytoscape’s GeneMania Plugin [20] which produced a network of interactions amongst the input gene set. This was limited to protein/protein interactions such as co-expression, direct interaction, and shared domains. The resulting networks were frequently very large (up to 1500 genes) and required further processing, which was done using ClusterViz [21], another Cytoscape plugin. Using the MCODE algorithm built into ClusterViz, sub-networks of very dense interactions amongst the input gene set were discovered and isolated in separate network files, which better facilitated downstream analyses. The genes in these sub-networks were interrogated for potential biological roles by performing gene ontology analysis in the ClueGO plugin [22] for Cytoscape. This produced weighted networks that revealed which biological pathways are most represented in the sub-networks produced by ClusterViz and simplified further analyses.

### Analysis of Common Target Genes Across All Four NDs

The purpose of this analysis was to identify genes that are likely to be targeted across all four NDs despite the largely unique miRNA expression profiles for each disease. The first step was to convert old miRNA names into the names used in miRBase version 21 using miEAA name converter utility [23]. MiRNA-target interaction data was then taken from miRTarBase [24] using strong evidence of interaction only (Luciferase reporter assay, qPCR, Western Blot). Genes targeted by two or more miRNAs from Supplementary Table 1 (229 miRNAs met this criteria) were then identified within each ND and we performed Venn diagram analysis to identify which target genes overlapped between all four NDs. This revealed 105 bona fide gene targets of miRNAs differentially expressed across AD, PD, ALS, and MS. The 105 target genes were then imported into Reactome [25] for pathway analysis to identify the roles these miRNA targets are likely to play in neurodegenerative disease processes. To ensure that the 105 target genes were not discovered due to bias inherent in the softwares used to identify them, we performed a simulation by randomly drawing the same number of miRNAs as in the knowledge base specific to each ND from miRTarBase and performing the same analysis. The distribution of the number of genes targeted in all four NDs over 1000 random draws was plotted and it showed that the number of genes discovered in our actual analysis was at least two times higher than in any of the random draws (Fig. 3a). Further interrogation of this data set was performed using Cytoscape and associated plugins (see previous section) as well as AgriGO V2 [26] and Reactome [25] to perform pathway analysis on this gene set.

### Phylogenetic Tree Analysis

This analysis was performed by acquiring the sequences of miRNAs of interest from miRbase [27]. The seed sequences (nucleotides 2–8 in the mature miRNA sequence [28]) were taken from the mature miRNA sequence and multiple sequence alignment (MSA) was performed using CLUSTAL Omega [29] which output the phylogenetic tree as part of the MSA analysis. The Newick string was then copied from CLUSTAL Omega and imported into Interactive Tree of Life (iTOL) for further visualization [30]. iTOL also produces images more suitable for publication.

### MiRNA Cluster Analysis

To investigate the homologies of the identified miRNAs, we searched MetaMirClust database for miRNA clusters. Different from conventional miRNA clusters which were arbitrarily defined by a fixed distance, MetaMIrClust identifies miRNA clusters (MirClust) based on miRNA classes with respect to maximum inter-miRNA distances (MID). Furthermore, the conserved co-occurrence MetaMirClusts in the same MID were discovered using a data mining method [31].

### Chromosomal Analysis, MiRNA Locations, and Functional Correlations

Chromosomal locations of miRNA genes were assessed to determine the association between miRNA gene location on chromosome and NDs. For this, the chromosomal locations for each miRNA were recorded from NCBI and added to the database. The start position for each miRNA gene was also recorded in the format required to use phenogram [32], which was then used to determine which miRNA containing genomic loci have the most associations with NDs.

## Results

### Differential Expression and Compartmental Distribution of MiRNAs Between NDs

**Fig. 1:**
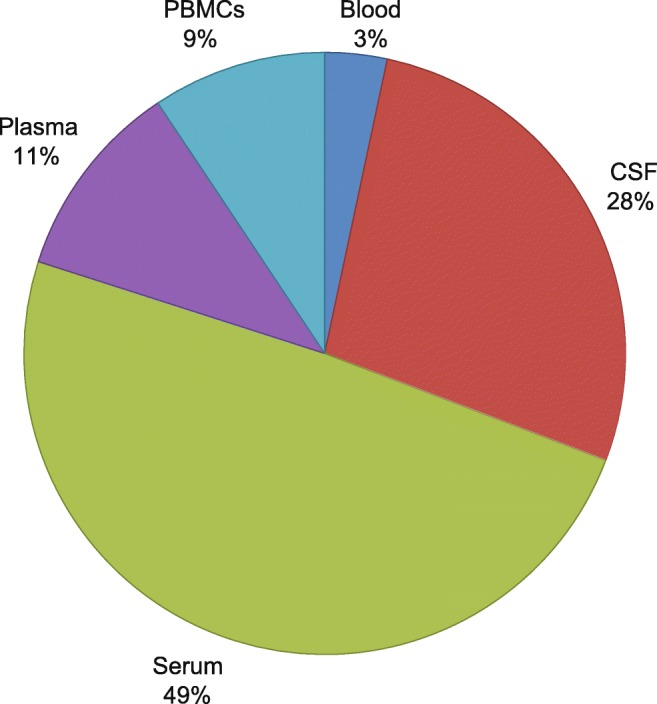
Pie chart showing the proportion of DE miRNAs derived from each source in various studies of ND. Forty-nine percent of DE miRNAs were recorded in serum, 28% in CSF, 11% in plasma, 9% in PBMCs, and 3% in whole blood

A total of 599 reports of differentially expressed miRNAs were referred to for building the knowledge database. It is evident from these human studies that the miRNAs were derived from diverse compartments such as blood, leukocytes, whole blood, PBMCs, plasma, serum, exosomes, and CSF (Fig. 1). Across all studies included herein, a total of 346 unique miRNAs were identified as differentially expressed (DE) in ALS, AD, PD, and MS. Of the 598 reports of differential expression, 347 reported downregulation whereas 251 reported upregulation of miRNAs across different compartments shown in Fig. 1 and Supplementary Table 1. As evident from Fig. 1, 49% of these miRNAs were analyzed from the serum compartment, 28% from the CSF, 11% from plasma, and 9% from PBMCs, with the remainder coming from whole blood. Interestingly, about 17% of the miRNAs were found within membranous structures, either in exosomes or other circulating cell types. The functional similarities and differences between cell versus biofluid-derived miRNAs have never been analyzed in this detail, especially in the context of NDs, which will shed most needed light on functional interactions between these miRNA in different blood compartments and their association with disease development and manifestation.

**Fig. 2:**
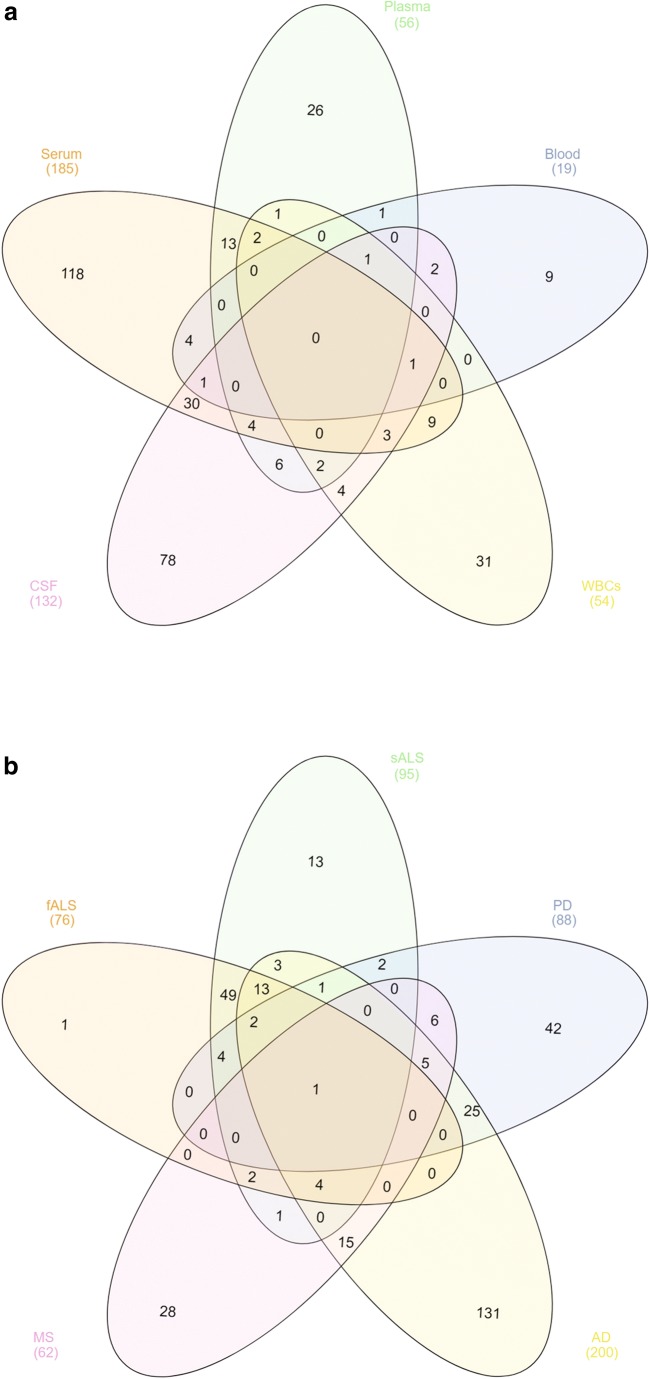
**a** Venn diagram of DE miRNAs in different compartments. Three hundred forty-six unique miRNAs were analyzed from diverse compartments obtained from 72 different human studies on miRNAs in AD, PD, ALS (fALS and sALS), and MS. No DE miRNAs were discovered in all of these fluids and expression profiles were mostly unique to each fluid. The largest crossover exists between CSF and serum. **b** Venn diagram illustrating the relationships between 346 differentially expressed miRNAs between diverse NDs. Hsa-miR-30b-5p was the single miRNA that was dysregulated in all four neurodegenerative diseases. The largest overlap was between fALS and sALS as these are clinical variants of a single pathology. The next largest overlap was between AD and PD

From the cumulative analysis of miRNAs from all the compartments to delineate relationships between miRNAs from diverse compartments, notable was the absence of generalized overlaps between different body fluids and cellular miRNAs as shown in Fig. 2a*,* though inter-compartmental overlaps were observed. Moreover, miRNA specificity for each compartment was also apparent with 46% miRNA specific to plasma (26/56); 63% to serum (118/185); 47% to blood (9/19); 59% to the CSF (78/132); and 57% to WBC (31/54) (Fig. 2a). Also, when inter-compartmental relationships were visualized, the largest number of miRNAs was shared between plasma, serum, and CSF, and to a lesser degree between plasma, blood, and WBCs, with surprisingly no overlaps between blood and WBCs. We believe this could be attributed to (i) that only three studies have been done on blood as opposed to several on WBCs, and (ii) that new miRNAs that do not have functional annotations assigned yet are not included in this analysis. Nonetheless, the difference between blood cells and WBCs is an interesting one, and if there are distinct miRNAs specific to WBCs, they may have important roles in immune defense and fighting off infections.

Overall, it is plausible to hypothesize that since the cells, plasma, serum, and exosomes are a part of the same biological milieu, the likelihood of functional interactions is higher, but how these functional interactions between miRNAs in diverse body fluid compartments influence disease outcome is difficult to ascertain. Further, how these blood compartment-based miRNAs maintain functional interactions with miRNAs in the CSF remains obscure. We believe that as more data is curated on miRNAs from diverse compartments and newly discovered miRNAs are assigned functional annotations, the definition of functional entities, interactions, and intersections will provide reliable leads for disease prediction and identification of new generation biomarkers and therapeutic candidates.

Next, we examined the logical relationships between miRNAs in different NDs and cumulatively visualized these in a Venn diagram *(*Fig. 2b). Most of the DE miRNAs showed four different expression patterns (i) disease-specific expression miRNAs, (ii) miRNA overlapping between two NDs, (iii) miRNAs overlapping between three different NDs, and (iv) expression intersections across all four NDs as shown in the Venn diagram (Fig. 2b). These expression patterns showed both up- and downregulation across NDs, with the preponderance of downregulation being the main feature of miRNA expression across NDs (58% downregulated; Supplementary Table 1). Although it is highly complex to delineate the functional biological relevance of miRNAs in body fluids as opposed to cellular miRNA in the context of NDs, the data shown in Fig. 2b implies that despite some overlaps between different NDs, there were miRNA unique to each ND (1 to fALS, 13 to sALS, 42 to PD, 131 to AD, and 28 to MS, respectively). Thus, the disease-specific entities may have considerable functional relevance too. Notable was the identification of hsa-miR-30b-5p, which was differentially expressed in all four NDs, which is the first evidence of a miRNA species that unifies all four NDs (Fig. 2b).

### Generalized Overlap of Hsa-miR-30b-5p in Neurodegenerative Diseases

Although hsa-miR-30b-5p was differentially expressed in all NDs profiled in this study, it was not differentially expressed in the same direction across all four NDs (hence its exclusion from Table 1; see supplementary Table 3 for miR-30 family). This miRNA was most frequently downregulated in either serum or plasma across ALS, AD, and PD. Interestingly, hsa-miR-30b-5p was upregulated in MS patients in both plasma and PBMCs. We believe that given the differential expression of hsa-miR-30b-5p across all four NDs, it is likely to be functionally relevant. To demonstrate this, we first looked for its cognate gene targets using DianaTools and identified KEGG pathways where these gene targets are enriched. The KEGG pathways were then analyzed for relevance to neurodegenerative diseases and processes in order to derive their functional relevance in the context of the diseases studied herein. Examination of the KEGG pathway annotations for miR-30b-5p showed that it not only overlapped between all four NDs, but was able to target genes in multiple pathways relevant to neurodegeneration across NDs. These pathways include histone methylation, ERBB2 and wnt signaling, protein folding and ubiquitination, mitochondrial function in apoptosis and necroptosis, and ion transport (Supplementary figures 1a & 1b).

**Table 1:** MiRNAs differentially expressed in the same direction across multiple NDs

MiRNA ID	fALS	sALS	PD	AD	MS	Analysis method	Source	Ref
Hsa-let-7a			↓(a)		↓(b)	miRGenes qPCR panel(a), qPCR(b)	Plasma(a, b)	[33](a), [34](b)
Hsa-let7i-5p	↑(a)	↑(a)		↑(b)		Microarray meta-analysis(a), qPCR(b)	Serum(a), CSF(b)	[35](a), [36](b)
Hsa-miR-106a-5p	↑(a)	↑(a)		↑(b)		Microarray meta-analysis(a), NGS and qPCR(b)	Serum(a), exosomes(b)	[35](a), [37](b)
Hsa-miR-10a-5p			↓(a), ↑(b)	↓(c)		NGS(a, c), TLDA(b)	CSF(a, c), CSF exosomes(c)	[38](a, c), [39]
Hsa-miR-132-3p		↓(a)		↓(b)		qPCR(a), NGS(b)	CSF(a, b)	[40](a), [38](b)
Hsa-miR-132-5p		↓(a)	↓(b)			qPCR(a), NGS(b)	CSF(a, b)	[40](a), [38](b)
Hsa-miR-136-3p	↓(a)	↓(a)	↓(b)	↓(c, d)		Exiqon qPCR panel and qPCR(a), NGS(b, d), TLDA(c)	Serum(a), CSF(b, c), CSF exosomes(d)	[41](a), [38](b, c), [39](d)
Hsa-miR-139-5p	↓(a)	↓(a)		↓(b)		Exiqon qPCR panel and qPCR(a), NGS(b)	Serum(a), CSF(b)	[41](a), [38](b)
Hsa-miR-143-3p	↓(a)	↓(a), ↑(b)		↑(c), ↓(a)		qPCR(a), microarray and qPCR(b), NGS and qPCR(c), TLDA and qPCR(d)	CSF(a), serum(b, c), serum exosomes(d)	[40](a), [42](b), [37](c), [43](d)
Hsa-miR-144-5p	↑(a)	↑(a)	↑(b)	↑(c)		Exiqon qPCR panel and qPCR(a), qPCR(b), NGS and qPCR(c)	Serum(a, c), CSF(b)	[41](a), [44](b), [45](c)
Hsa-miR-146a-5p			↓(a)	↓(b, c)		qPCR(a, c), TLDA(b),	Serum, CSF(b), plasma(c)	[46](a), [43](b), [47](c)
Hsa-miR-15b			↓(a)		↑(b), ↓(c, d)	Microarray and qPCR(a, d), microarray(b), qPCR(c)	Serum(a, c), T cells(b), plasma(d)	[48]|(a), [49](b), [50](c), [51](d)
Hsa-miR-15b-5p	↓(a)	↓(a)		↓(b, c)	↓(d)	Human miFinder PCR array(a), TLDA(b), qPCR(c), NGS(d)	CSF(a, b), plasma(c), serum exosomes(d)	[52](a), [43](b), [47](c), [53](d)
Hsa-miR-16-2-3p			↑(a), ↓(b)		↑(C)	Microarray and qPCR(a), NGS(b), NGS, microarray and qPCR(c)	Blood(a, c), serum(b),	[54](a), [38](b), [55](c)
Hsa-miR-193b		↓(a)		↓(a)		Microarray and qPCR(a), qPCR(b)	Leukocytes(a), serum(b)	[56](a), [57](b)
Hsa-miR-19a-3p	↑(a)	↑(a)	↑(b), ↓(c)			Exiqon qPCR panel and qPCR(a), NGS(b), qPCR(c)	Serum(a, c), CSF(b)	[41](a), [38](b), [58](c)
Hsa-miR-200a-3p			↑(a)	↑(b)		qPCR(a), Exiqon qPCR panel(b)	CSF(a), plasma(b)	[44](a), [59](b)
Hsa-miR-219				↓(a)	↓(b)	OpenArray qPCR(a), qPCR(b)	CSF(a, b)	[60](a), [61](b)
Hsa-miR-221-3p	↑(a)	↑(a)		↑(b)		Microarray meta-analysis(a), NGS and qPCR(b)	Serum(a, b)	[35](a), [45](b)
Hsa-miR-223-3p				↓(a)	↓(b)	TLDA(a), NGS(b)	CSF(a), serum exosomes(b)	[43](a), [53](b)
Hsa-miR-22-3p			↓(a)	↓(b)		NGS(a), NGS and qPCR(b)	CSF(a), serum(b)	[62](a), [45](b)
Hsa-miR-22-5p			↑(a)	↑(b)		Microarray and qPCR(a), NGS(b)	Blood(a), serum(b)	[54](a), [38](b)
Hsa-miR-24			↑(a, b)		↑(c)	qPCR(a, b, c)	CSF(a, c), serum exosomes(b)	[63](a), [64](b), [65](c)
Hsa-miR-25-3p	↑(a)	↑(a)		↑(b)		Microarray meta-analysis(a), Exiqon qPCR panel and qPCR(b)	Serum(a, b)	[35](a), [66](a)
Hsa-miR-29a			↓(a)	↑(b), ↓(c, d)		qPCR(a, b, c), microarray and qPCR(d)	Serum(a, c, d), CSF(b)	[67](a), [68](b), [69](c), [70](d)
Hsa-miR-29b-3p			↓(a)	↓(b)		qPCR(a), NGS and qPCR(b)	Serum(a), plasma exosomes(b)	[67](a), [71](b)
Hsa-miR-29c			↓(a,b)	↓(c)		qPCR(a, b), TLDA(c)	Serum(a, b), CSF exosomes(c)	[46](a), [67](b), [39](c)
Hsa-miR-29c-3p			↓(a), ↑(b)	↓(c)		qPCR(a, c), NGS(b),	Serum(a), PBMCs(b), CSF(c)	[58](a), [72](b), [36](c)
Hsa-miR-301a-3p				↓(a)	↓(b)	Exiqon qPCR panel(a), NGS and ddPCR(b)	Plasma(a), serum exosomes(b)	[59](a), [73](a)
Hsa-miR-30e-5p			↓(a), ↑(b)	↓(c), ↑(d)		NGS(a, b), NGS and qPCR(c, d)	Serum(a, c), PBMCs(b), serum exosomes(d)	[38](a), [72](b), [74](c), [37](d)
Hsa-miR-324-3p	↑(a)	↑(a)	↑(b)			Microarray meta-analysis(a), microarray(b)	Serum(a, b)	[35](a), [75](b)
Hsa-miR-328		↓(a)			↓(b)	Microarray and qPCR(a, b)	Leukocytes(a), PBMCs(b)	[76](a), [77](b)
Hsa-miR-338-3p		↑(a)	↑(b)			Microarray and qPCR(a), NGS(b)	Leukocytes(a), serum(b)	[76](a), [38](b)
Hsa-miR-342-3p				↓(a, b, c)	↓(d)	NGS and qPCR(a, b, c), NGS(d)	Plasma exosomes(a), serum exosomes(b, d), serum(c)	[71](a), [37](a), [74](c), [53](d)
Hsa-miR-365a-3p				↓(a)	↓(b)	TLDA(a), Exiqon qPCR panel and qPCR(b)	CSF(a), serum(b)	[43](a), [66](b)
Hsa-miR-370			↓(a)		↓(b)	CSF(a), serum exosomes(b)	NGS(a, b)	[38](a), [53](b)
Hsa-miR-375				↓(a, b),	↓(c)	OpenArray qPCR(a), NGS(b), qPCR(c)	CSF(a), serum(b, c)	[60](a), [38](b), [78](c)
Hsa-miR-409-3p			↓(a), ↑(b)		↓(c)	NGS(a, c), TLDA(b)	CSF(a), CSF exosomes(b), serum exosomes(c)	[38](a), [39](b), [53](c)
Hsa-miR-424-5p			↑(a)	↑(b)		NGS(a), NGS and qPCR(b)	PBMCs(a), serum exosomes(b)	[72](a), [37](b)
Hsa-miR-431-3p			↓(a)	↓(b)		NGS(a, b)	CSF(a, b)	[38](a, b)
Hsa-miR-433			↓(a, b)	↓(c)		qPCR(a), NGS(b, c)	Plasma(a), CSF(b, c)	[79](a), [38](b, c)
Hsa-miR-485-5p			↑(a)	↑(a)		TLDA(a)	CSF exosomes(a)	[39](a)
Hsa-miR-486-5p				↑(a)	↑(b)	Exiqon qPCR panel and qPCR(a, b)	Plasma(a), serum(b)	[59](a), [66](b)
Hsa-miR-505-3p			↑(a)	↑(b)		Microarray and qPCR(a), OpenArray qPCR(b)	Plasma(a), CSF(b)	[80](a), [60](b)
Hsa-miR-532-5p				↓(a, b)	↓(c)	NGS and qPCR(a), TLDA(b), NGS and ddPCR(c)	Blood(a), CSF(b), serum exosomes(c)	[81](a), [43](b), [73](c)
Hsa-miR-769-5p			↓(a)	↓(b)		NGS(a, b)	PBMCs(a), CSF(b)	[72](a), [38](b)
Hsa-miR-873-3p			↑(a)	↑(b)		NGS(a, b)	CSF(a), serum(b)	[38](a, b)

↑ denotes upregulation of miRNA expression, and ↓ denotes downregulation of miRNA expression

qPCR, quantitative polymerase chain reaction; NGS, next generation sequencing; TLDA, Taqman low-density arrays; ddPCR, digital droplet PCR

**Fig. 3:**
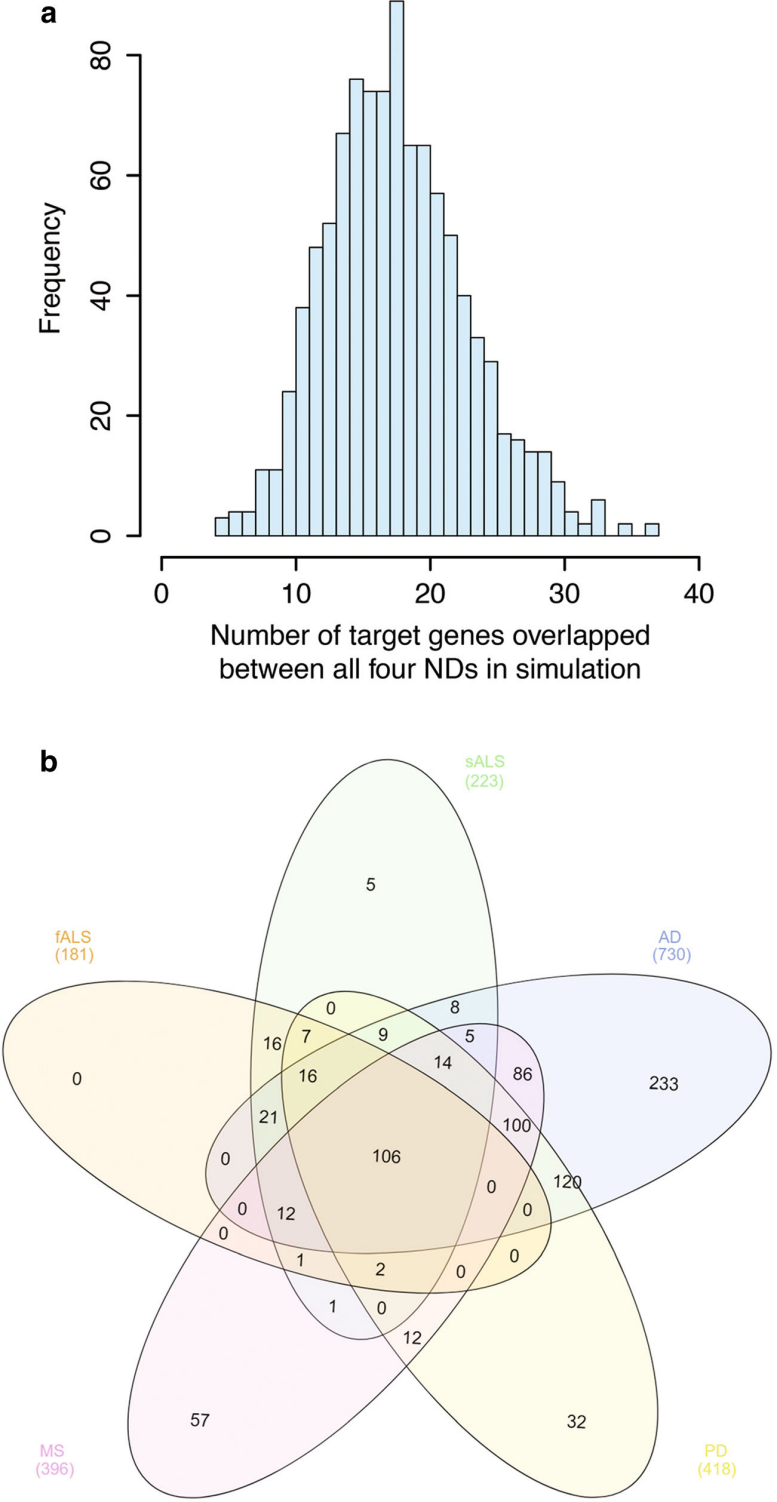
**a** Results of miRNA-target gene simulation. The results show that random draws of miRNAs from the knowledge base discover only 20 overlapping target genes or less in the majority of simulations. At most, 40 overlapping target genes are discovered using random miRNA draws. This shows that the 106 overlapping target genes are not random or from bias in the analysis, but are the result of unbiased functional enrichment at both the miRNA and target gene level across the NDs profiled in this study. **b** Venn diagram analysis of target genes of miRNAs involved in NDs. One hundred six target genes are common across all NDs analyzed in this study. This gene set was further analyzed using Reactome [25]

**Fig. 4:**
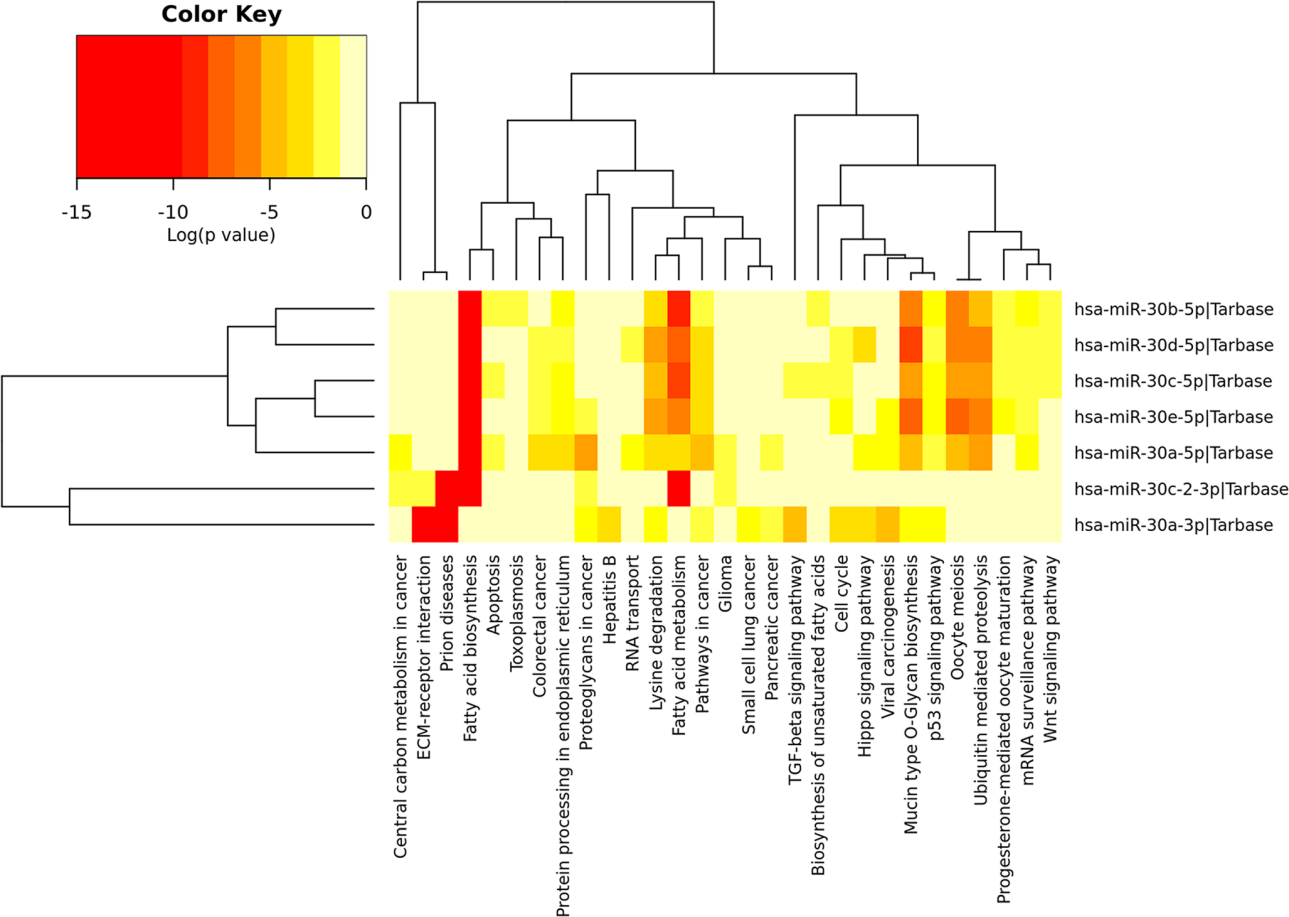
Heatmap of biological pathways targeted by the miR-30 family. Most of the miRNAs in this family target genes associated with fatty acid biosynthesis and metabolism except for hsa-miR-30a-3p. Hsa-miR-30c-2-3p and hsa-miR-30a-3p target genes related to prion disease and ECM receptor interaction. Ubiquitin-mediated proteolysis is also statistically significantly associated with this miRNA family, as is mucin-type O-glycan biosynthesis

Although only hsa-miR-30b-5p overlapped between NDs, we further investigated the possible functional significance and involvement of other members of the miR-30 family (hsa-miR-30b-5p, 30d-5p, 30c-p, 30e-5p, 30a-5p, 30c-2-3p, and 30a-3p) in neurodegenerative diseases. Comparative and cumulative visualization of the miR-30 family members showed critical differences between them with hsa-miR-30b-5p, 30d-5p, 30c-p, 30e-5p, and 30a-5p collectively targeting fatty acid biosynthesis pathway, the miR-30c-2-3p targeting genes in prion disease pathway, and miR30a-3p targeting both the prion and the ECM receptor interaction (Figs. 3 and 4), implying a division of labor between -5p and -3p miRNA species at the level of their cognate targets. It appears that the miR-30 family as a whole is functionally versatile, and may therefore play a critical role in neurodegeneration.

### MiRNAs Wear Many Different Hats: Functional Convergence of MiRNAs on Several Common Pathways

As seen through a plethora of studies on NDs referenced herein, it is apparent that considerable variability exists in miRNAs between patient groups and also between the diverse compartments they were derived from. Despite this variability, one of the key questions is how these miRNAs functionally unite to incur a disease-specific effect (positive or negative) despite this variability? In order to understand this functional aspect and its significance, the  347 ND-associated DE miRNAs were analyzed for their cognate gene targets which were further subjected to KEGG pathway analysis to derive functional annotations of miRNAs and their gene targets using DIANA MirPath v3 [18]. The miRNAs associated with each ND were analyzed individually on this platform to perform heatmap analysis (supplementary Figures 2a*-e*). These analyses revealed that the most statistically significant pathways targeted by the ND-associated miRNAs were common to each disease, even though the sets of miRNAs were not identical. This implies that diverse miRNAs functionally converge on the same biological pathways by targeting common mRNA transcripts that are intimately and intrinsically related to neurodegenerative processes. In other words, several miRNAs may act as regulators of both known and novel biological processes involved in neurodegeneration. For instance, hsa-miR-19a-3p, hsa-miR-221-3p, and hsa-miR-29a-3p all target WWTR1 in ALS, PD, and AD, a pivotal effector gene in the Hippo signaling pathway. These miRNAs were identified in the serum, CSF, blood, and PBMCs across ALS, PD, and AD.

**Fig. 5:**
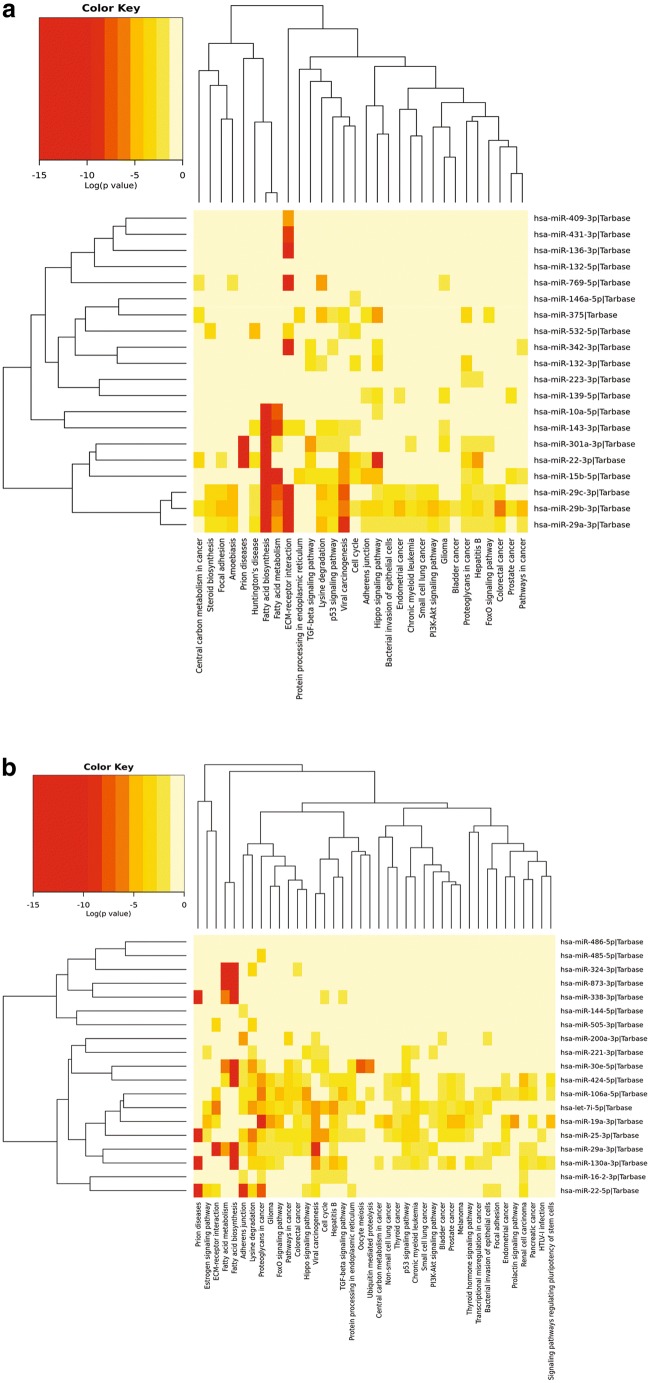
**a** Heatmap analysis of miRNAs downregulated across multiple NDs. Fatty acid biosynthesis, fatty acid metabolism, and ECM receptor interactions are the most statistically significant pathways discovered in this analysis. Viral carcinogenesis was discarded as it was not linked to neurodegeneration and may be the result of biases inherent in this type of software analysis [120]. **b** Heatmap analysis of miRNAs upregulated across multiple NDs. Fatty acid biosynthesis, fatty acid metabolism, ECM receptor interactions, and prion diseases are the most statistically significant pathways discovered in this analysis. Proteoglycans in cancer were discarded as it was not linked to neurodegeneration and may be the result of biases inherent in this type of software analysis [120]. Downregulation of miRNAs: a generalized feature of NDs

The other pathways that were consistently targeted collectively by several miRNAs were the ECM receptor interactions, fatty acid metabolism/synthesis, prion diseases, adherens junctions, Hippo signaling, and TGFβ signaling (Fig. 5a, b), all of which bear immense relevance to neurodegenerative diseases. To address this further, we simultaneously examined the relationships in an unbiased manner between miRNAs derived from each body fluid compartment, irrespective of the disease to derive explanation on how diverse compartments contribute to disease development. We also sought to determine if there were functional intersections between miRNA, for instance between blood and CSF. Interestingly, there was very limited crossover between miRNAs in each compartment (Fig. 2b). To our knowledge, this is the first integrated data set showing that miRNAs were largely unique to each compartment in these NDs, implying possible compartment-specific regulation of relevant biological processes and functional convergence of miRNAs from diverse compartments. This was apparent from further examination of pathways and gene ontologies that showed substantial functional crossover amongst these compartments, despite having non-identical (and largely unique) miRNA signatures associated with them. ECM receptor interactions, fatty acid metabolism/synthesis, prion diseases, adherens junctions, Hippo signaling, and TGFβ signaling were statistically significant pathways that were most likely to be targeted by each set of miRNAs present in each fluid (supplementary Figure 3a*-e*).

To further probe the convergence of these miRNAs, we determined which cognate target mRNAs could be targeted by at least two miRNAs in each ND listed in Supplementary table 1. We then performed Venn diagram analysis (Fig. 3b) to find which of these target mRNAs were associated with all four NDs. This analysis revealed 105 mRNAs that were targeted by at least two miRNAs from *Supplementary Table* 1. Gene ontology analysis of these overlapping mRNAs was then performed using multiple platforms (AgriGO v2, Reactome, ClueGO/Cluepedia) to better understand exactly which pathways are being converged upon across multiple NDs. Surprisingly, fatty acid biosynthesis and metabolism were not implicated as prominently as they were in the MiRPath analysis as the only related pathways that came out as statistically significant were related to PPARA and SREBF which are involved in lipid metabolism and cholesterol synthesis respectively (Supplementary Fig. 4; Reactome report). The most significant linked pathways in this analysis were gene expression (transcription), signal transduction, and immune system functions. Interestingly, the signal transduction pathways included TGFβ and wnt signaling pathways requiring function of components of the Hippo signaling pathway, which is itself statistically associated with the genes targeted by multiple miRNAs. This is corroborated by the MiRPath analysis, strongly suggesting that these pathways are functionally relevant across NDs (Fig. 5a, b).

### MiRNA Sequence Homology: a Guide to Functional Convergence Across NDs

Seeing the participation of multiple miRNAs in regulating the same biological function or pathways as described in the previous section, we sought to phylogenetically determine the relationship based on sequence homologies between these DE miRNAs both at the level of seed sequence and the whole miRNA sequence (Fig. 8a, b). Three different patterns emerged from these analyses (family-based clustering, miRNA clusters under a common promoter, and clustering between unrelated miRNAs) suggesting the role of sequence homologies between partner miRNAs that guide the same function or a biological process.

**Fig. 6:**
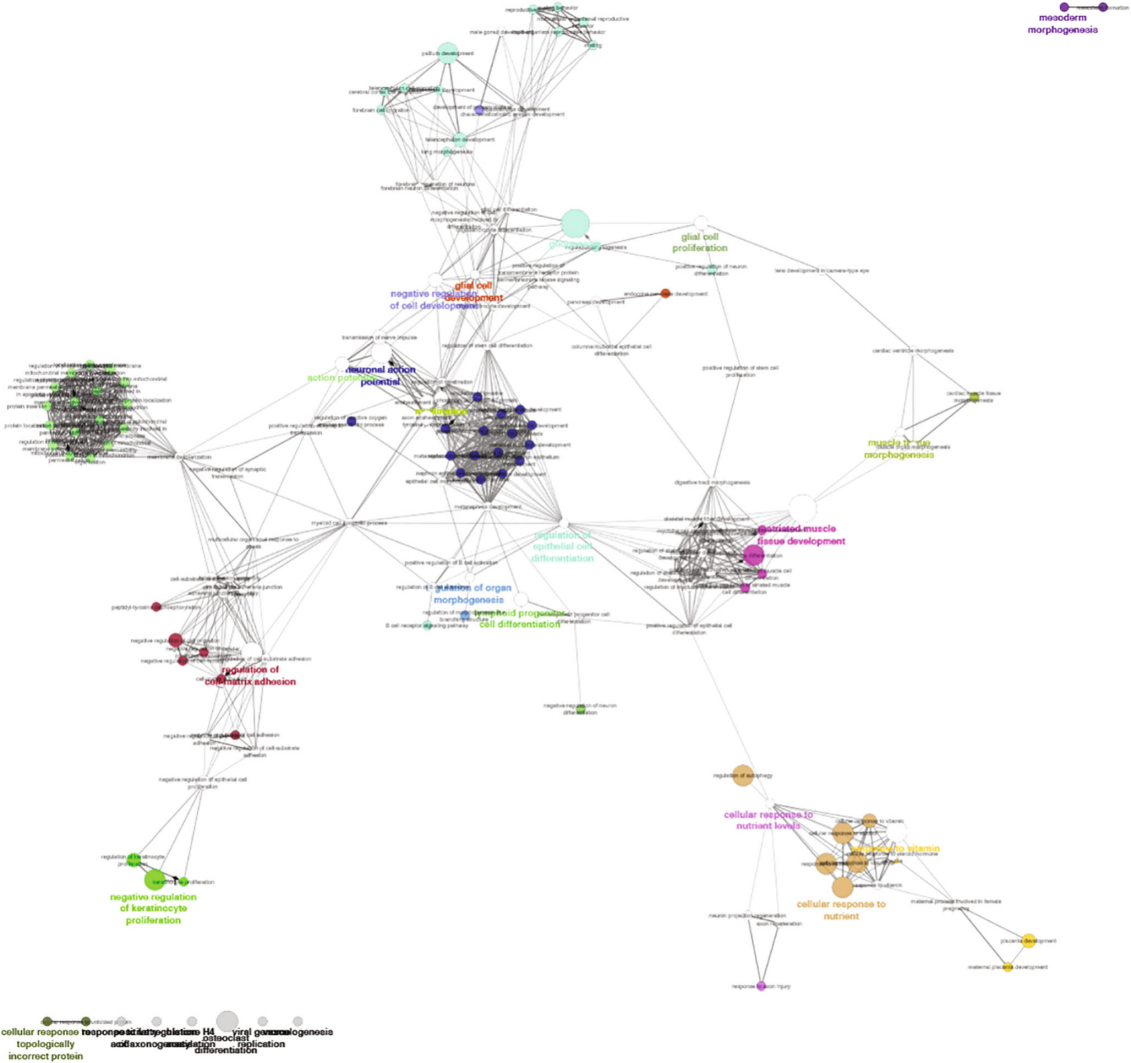
Gene ontology output from ClueGO. The top 100 target genes of each downregulated miRNA in Table 1 were identified using Targetscan according to context score. These target lists were merged into one large list which was used as input into the GeneMANIA plugin in Cytoscape. This identified protein-protein interactions amongst this gene set. ClusterViz was used to isolate hubs of particularly dense interactions which were imported into separate networks. These networks were then analyzed using the ClueGO plugin for Cytoscape which produced this gene ontology diagram

As the majority of DE miRNAs shown in Table 1 are downregulated, which is generally a trend seen across diverse studies on neurodegenerative diseases included herein, we sought to analyze the biological function of these downregulated miRNAs in detail. The hierarchical clustering analysis focused on the potential contribution of each miRNA individually, and some miRNAs could not be analyzed by this method. In order to probe deeper, based on the context score, we identified the top 100 target genes for each miRNA shown in Table 1 that were downregulated in multiple NDs using TargetScan [19]. This was to identify the most likely mRNAs to become de-repressed by the loss of these miRNAs. We combined these target genes into one large list and used Cytoscape and the Genemania plugin [20] to identify interactions amongst this gene set. We then used MCODE [21] to identify hubs of particularly dense interactions with the aim of identifying biological processes and pathways that could be especially de-repressed by the loss of the miRNAs that target them. These results are presented in the heatmap (Fig. 6)*,* which shows the functional pathways intrinsically involved across NDs, which functionally unify them.

The nodes related to the regulation of cell matrix adhesion in Fig. 6 are of particular importance as these processes are expected to be affected based on hierarchical clustering analysis and also given the strong statistical significance of the ECM receptor interaction ontology. However, the genes involved in this particular ontology have little to do with HSPGs, and more to do with the Hippo signaling pathway. NF2 is represented strongly in the cell matrix adhesion ontological cluster in Fig. 6. This gene is a non-canonical activator of the Hippo signaling pathway as it can cause the phosphorylation of LATS1/2 [82], potentially resulting in the increased apoptosis and decreased capacity for tissue repair put forth by Wang and Wang [83]. Thus, the loss of the miRNA(s) that target this gene may contribute towards this effect.

The potential for such an effect is further supported by the strong detection of processes involved in mitochondrion permeabilization in apoptosis. If cells involved in neurodegeneration are innately more likely to undergo apoptosis due to hyperactive Hippo signaling, it follows into other apoptotic pathways to become de-repressed. Of particular relevance here were the genes YWHAH, TFDP1, EYA2, and BCL2. YWHAH (targeted by miR-365-3p) which comes from a family of proteins known as 14-3-3, many of which are known to be involved in neurofibrillary tangles (NFT) commonly seen in AD [84]. TFDP1 (targeted by miR-30b-5p and miR-365a-3p) is also associated with NFTs in AD [85], and EYA2 (targeted by miR-219) has been associated with brain pathologies seen in Lewy body dementia [86]. BCL2 (targeted by miR-365a-3p) upregulation however seems to be a cause of neuroprotection through decreased reactive oxygen species (ROS) generation [87, 88]. This incongruity makes clear the need to determine which cellular processes are occurring as part of disease pathology and which processes are occurring to combat the disease process. This can only be resolved with further study.

The other ontological clusters of major importance are those related to neural tissue growth, differentiation, and function (cyan, light green and dark blue; Fig. 6). Within these clusters, CDK6 and HES5 were consistently found. CDK6 (targeted by miR-15b-5p) has been shown to be hyperactive in PD patients and was shown to be a promising therapeutic target by Alquézar et al., [89]. CDK6 is also an interacting partner of TDP43 [90], a gene known to be dysregulated in ALS [40] and in frontotemporal lobular dementia [91]. HES5 (targeted by miR-219) is an established factor in neuronal differentiation that becomes downregulated during this process to allow for MASH1 expression that is necessary for this process [92]. HES5 is also the target of a pre-clinical study aimed at treating AD and PD [93].

Gliogenesis may also be affected by overexpression of CDK6, HES5, and NF2, with several other genes contributing. APCDD1 (targeted by miR-301a-3p) for example is an inhibitor of the wnt signaling pathway in a similar manner to HES5 [94, 95]. ETV5 (targeted by miR-219) is a transcription factor (TF) that has been shown to be part of the molecular and functional signature of microglia that is dependent on TGFβ [96]. This gene is also suspected to be a central regulator in neurofibromatosis type 1 and low-grade optic gliomas [97]. Certain PD-associated mutations are suspected to allow binding of the ETV5 TF to the promoter for ATG12, which interferes with autophagy function and promotes the development of PD [98]. ETV5 is also upregulated in a mouse model of spinocerebellar ataxia [99].

Overall, the data presented in Fig. 5a, b, where we analyzed pathway association of DE miRNA across NDs, clearly suggest that both up- and downregulated miRNAs target similar pathways, and there was a high concordance in the nature of pathways targeted by both up- and downregulated miRNAs (Fig. 5a, b).

### Dysregulated MiRNA Families Across NDs Share Conserved Target Gene Sets

**Fig. 7:**
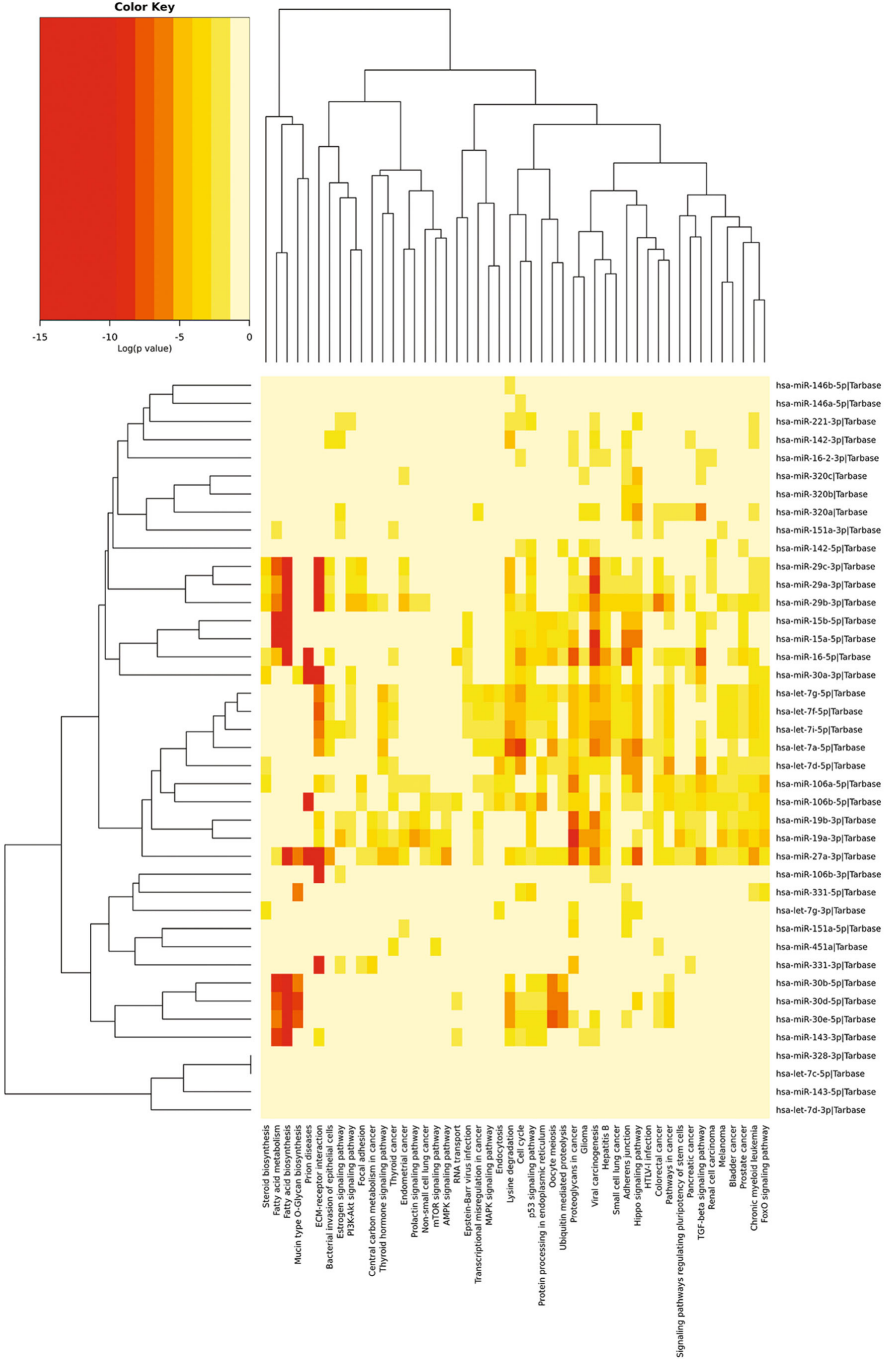
Heatmap analysis of miRNA families involved in neurodegenerative processes. Fatty acid biosynthesis, fatty acid metabolism, ECM receptor interactions, and prion disease are the most statistically significant pathways discovered in this analysis

Another perspective taken in analyzing this data set was to isolate families of miRNAs that are related in terms of their sequence and predicted target genes. This further illustrates the convergence of semi-unique miRNA profiles on a small group of biological pathways. For example, the heatmap (Fig. 7) shows strong functional overlap between the miR-29 family (miR-29a, miR-29b and miR-29c), the miR-15/16 cluster (miR-15a, miR-15b and miR-16), and the *miR-30 family* (*miR-30a, miR-30b, miR-30d and miR-30e*). This group of miRNAs is more likely to target the fatty acid biosynthesis pathways. There is also a great deal of overlap between the miR-29 family and the let-7 family (let-7a, d, f, g, and i). This is mainly through their ability to target ECM receptor interactions.

**Fig. 8:**
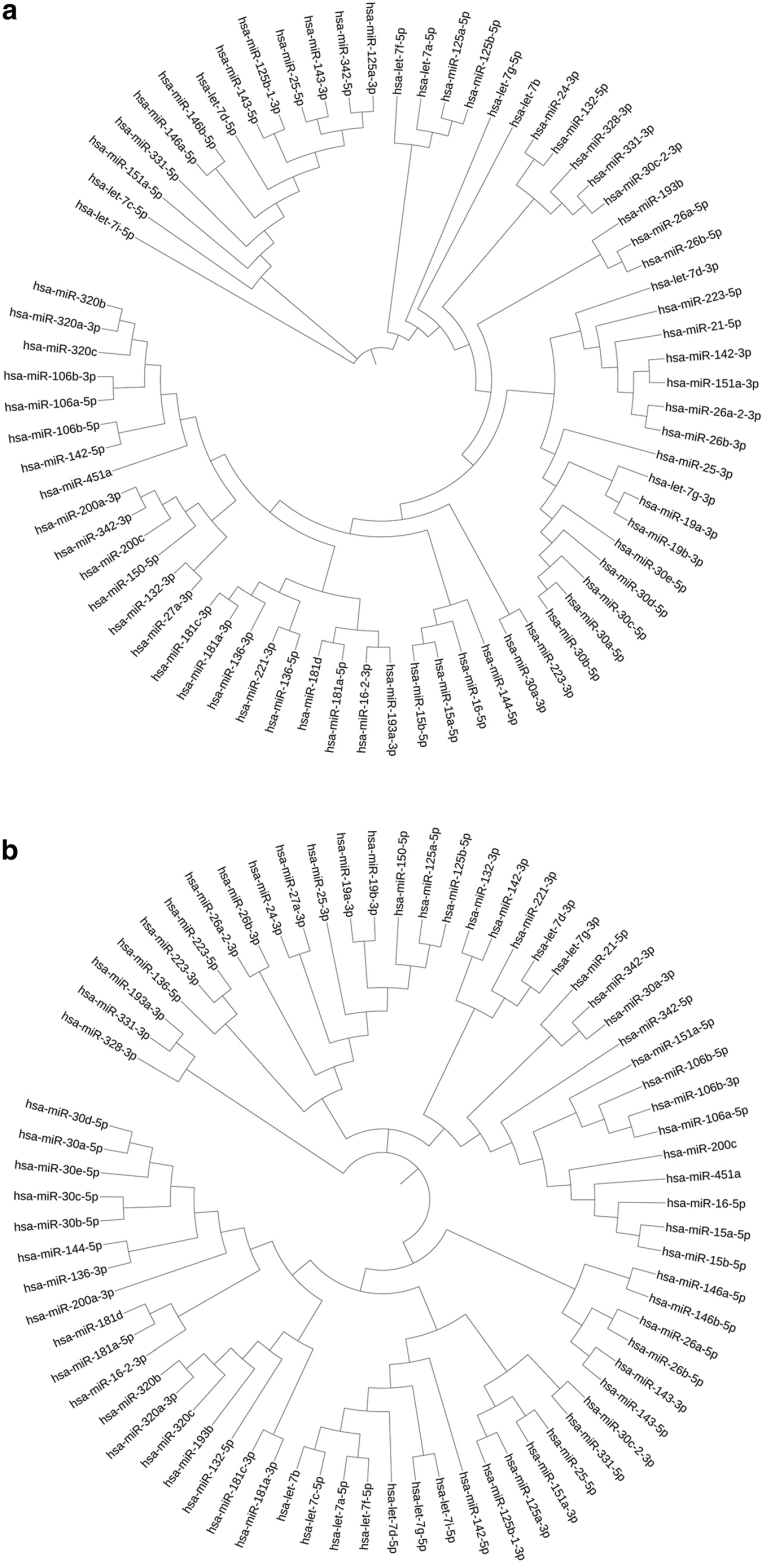
**a** Phylogenetic analysis of seed regions of miRNA families dysregulated in multiple NDs. MiRNAs from diverse families cluster closely together based on seed sequence MSA. **b** Phylogenetic analysis of entire miRNA sequence of miRNA families dysregulated in multiple NDs. MiRNAs from diverse families cluster closely together based on seed sequence MSA

Multiple sequence alignments performed amongst this miRNA set reveal that there is a high degree of sequence similarity amongst portions of this gene set, especially in the miRNA seed regions, which are most important in target binding [28] (see Fig. 8a). There are however many differences throughout the remainder of the sequence with unknown implications for target binding. This evidence coupled with the pathway analysis suggests that these miRNAs may lead us to common treatment options for several NDs. However, the direction of dysregulation is much less consistent amongst this group, and the split between upregulated and downregulated miRNAs is more even than in the previous analysis (63 reports of downregulation, 53 reports of upregulation). Although our study has laid a solid foundation for future work, the true biological significance of each miRNA to each condition will require further experimental work to support the computational evidence presented here.

**Fig. 9:**
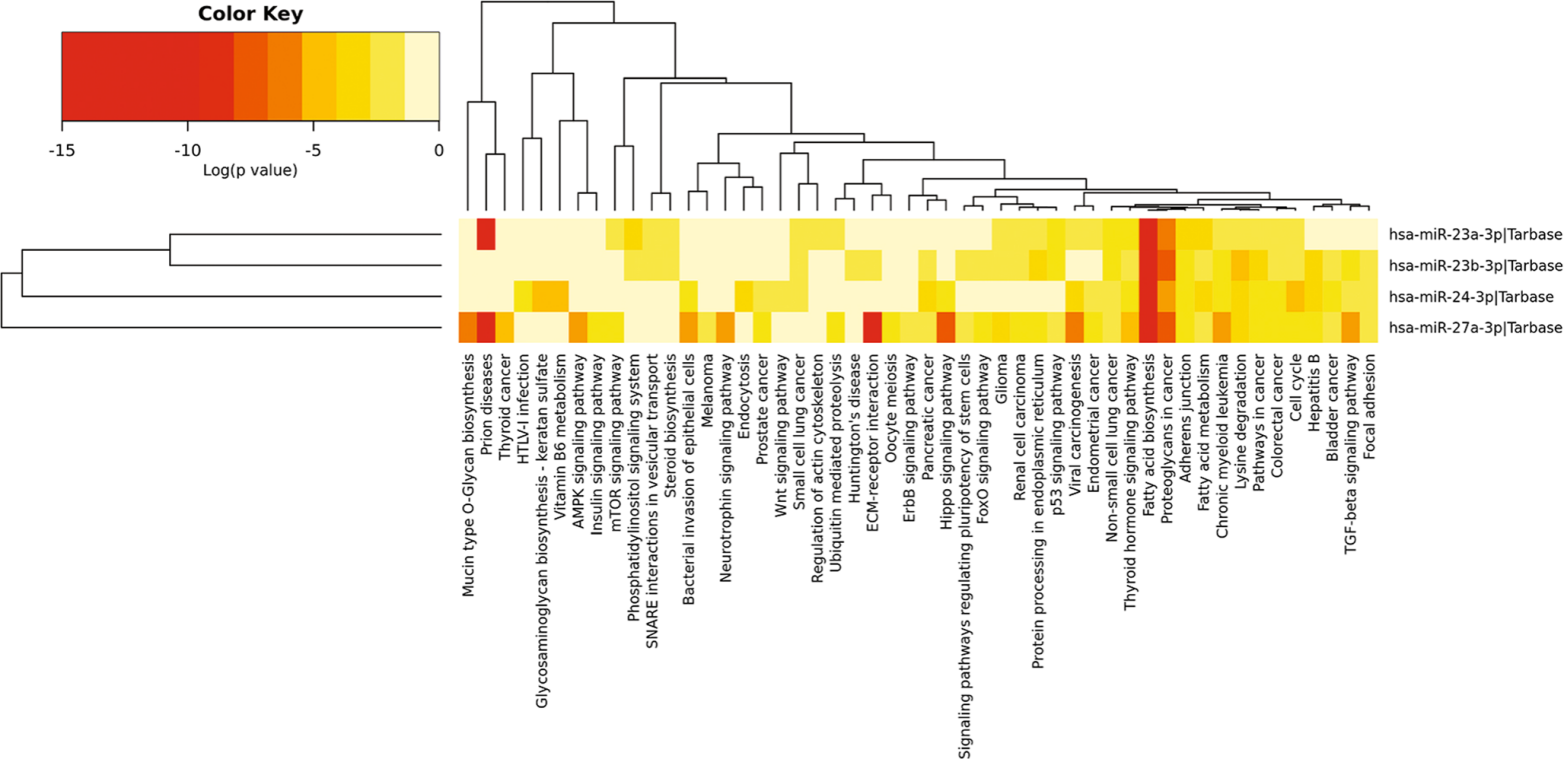
Heatmap analysis of targets of miR-23/24/27 cluster. The most statistically significant clusters of interest were fatty acid biosynthesis, prion diseases, ECM receptor interactions, and the Hippo signaling pathway

An important question that remains to be resolved is the presence and/or absence of miRNA clusters across NDs. In our analysis, only one cluster was detected, the miR-23/24/27 cluster (Supplementary Table 2). This is surprising given that many of the miRNAs shown in Supplementary Table 1 are members of miRNA clusters which are frequently co-transcribed and are usually under transcriptional control by the same promoter. Further analysis of the gene targets of the miR-23/24/27 cluster reveals that the most statistically significant pathways targeted by this miRNA are highly congruous with the analyses presented in Fig. 5a, b. The miR-23/24/27 cluster targets fatty acid biosynthesis, prion diseases, ECM receptor interactions, and the Hippo signaling pathway (Fig. 9*)* which suggests that the target processes of this miRNA cluster are shared within the cluster, and also overlap strongly with the miRNA families (Fig. 7) and the broader trends of miRNAs across these NDs (Fig. 5a, b).

### Genomic Spread of Dysregulated MiRNA Genes

**Fig. 10:**
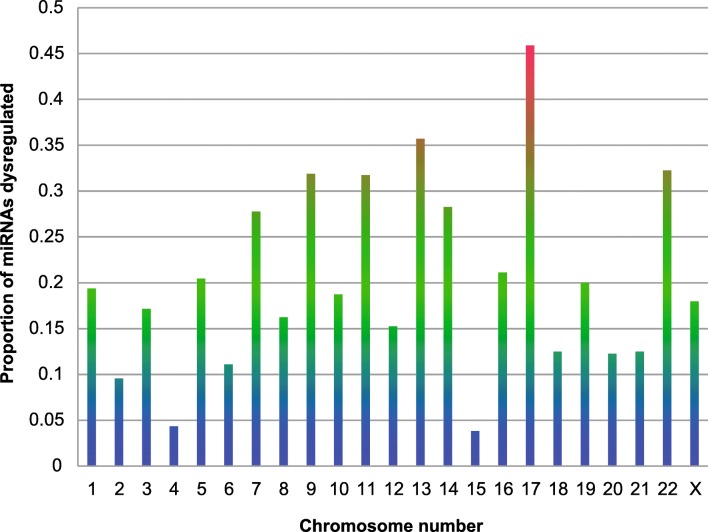
Representation of the proportion of miRNA genes resulting in a dysregulated mature miRNA in different NDs. All four NDs (AD, PD, ALS, and MS) were analyzed for evaluating the role of chromosomes in the context of miRNAs. Chromosome 17 has the highest proportion of miRNAs dysregulated across all four NDs

**Fig. 11:**
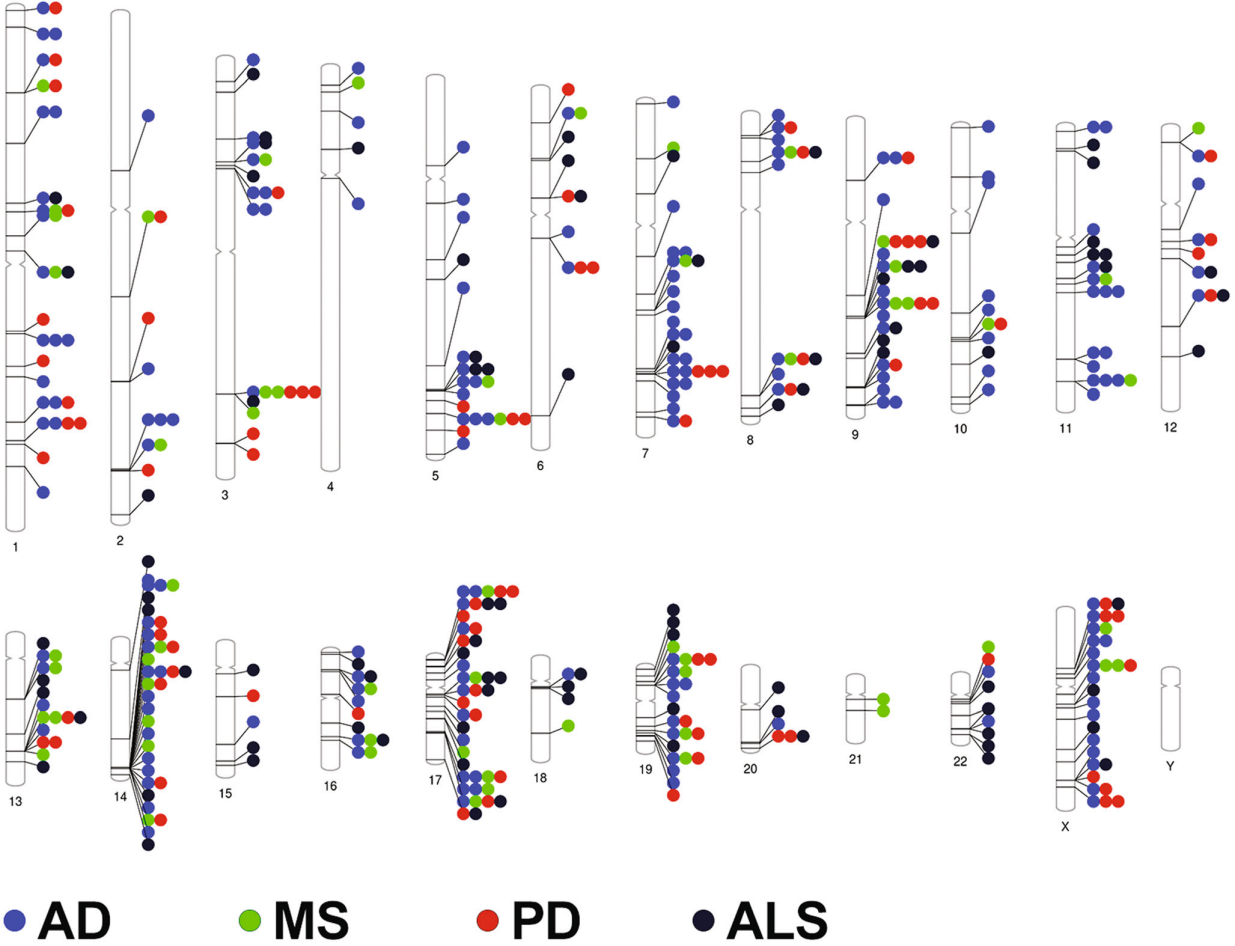
Phenogram showing mapping of dysregulated miRNA to genomic loci on diverse chromosomes. Chromosome 17 has the most multiple loci associated with multiple NDs, followed by chromosome 19. The long arm of chromosome 14 has a large number of miRNA gene loci located close to one another that are associated with multiple NDs. Chromosomes 7 and X host a large number of miRNA genes associated with AD. Chromosome 21 hosts miRNA genes associated with MS only. Chromosome 4 miRNA genes are relatively unaffected in NDs

Across all NDs assessed in this study, several notable trends were observed. Firstly, although the dysregulation was seen across multiple chromosomes, only a handful of chromosomes showed higher proportion of their miRNAs becoming dysregulated during NDs. These chromosomes were 9, 13, 17, and 22 respectively, and of these, the chromosome 17 appeared to be carrying the highest proportion of the dysregulated miRNA genes (approximately 42%) associated with at least one or more NDs (Fig. 10). This data is unique in showing the preponderance of chromosomal association of miRNA genes being enriched on some chromosomes over others, which implies that a very deep layer of miRNA dysregulation exists, which is possibly dictated by their chromosomal location. Interestingly, these dysregulated miRNA clusters on a particular chromosome were able to predict the association with a neurodegenerative disease. For instance, parallel comparisons of the chromosomal location breakdown revealed more unique features to each disease. fALS and sALS have mostly identical patterns of miRNA involvement as the studies on these NDs frequently did not differentiate between the two forms of the disease, or the expression pattern was common to both. However, we observed a much higher involvement of chromosome 17 miRNAs involved in sALS than in fALS, which has not been shown before (Fig. 11). MS also had unique elements with hsa-miR-let-7c and hsa-miR-648a being the only miRNA genes located on chromosome 21 (Fig. 11). For PD, we observed the representation of miRNA genes across diverse chromosomes, except chromosomes 21, 18, 11, and 4 which had no associations with this disease. The strongest association with PD was with miRNAs on chromosome 17 (Fig. 11).

Interestingly, the analysis of miRNA genes in AD showed quite distinct patterns with a higher representation in chromosomes 5 and 7 that differentiated AD from other NDs. A unique trait of AD was it being X-linked characterized by higher representation of miRNA genes on the X chromosome than any other NDs studied herein. Supporting this, research from the Mayo Clinic has shown that women who inherited two copies of a variant in the PCDH11X gene, found on the X chromosome, are at considerably greater risk of developing AD [100]. To date, no association study has clearly addressed the contribution of X chromosome-related miRNAs in AD, and we believe that miRNA genes on chromosome X can be potential biomarkers. The genes encoding X-linked miRNAs (hsa-miR-106a-5p, hsa-miR-18b-5p, hsa-miR-221-3p, hsa-miR-222, hsa-miR-223-3p, hsa-miR-361-5p, hsa-miR-384, hsa-miR-424-5p, hsa-miR-501-3p, hsa-miR-502-3p, hsa-miR-505-3p, hsa-miR-532-5p, hsa-miR-545-3p, hsa-miR-766, and hsa-miR-98-5p) may have immense relevance in unveiling the regulation of cognate gene targets, in addition to biomarker and therapeutic development for AD.

In the context of this analysis, it is important to iterate that there were miRNA genes found on chromosome X for other NDs, although their representation was much lower or insignificant except for the PD where miRNA genes were found to be encoding miRNAs—hsa-miR-221, hsa-miR-222,hsa-miR-222-3p, hsa-miR-223-5p, hsa-miR-424-5p, hsa-miR-505, hsa-miR-505-3p, and hsa-miR-542-3p, respectively, with some overlapping miRNA genes with AD (miR-222, miR-424-5p and 505-3p (see supplementary Table 1). Interestingly, X-linked dystonia-parkinsonism is a movement disorder that has been found only in people of Filipino descent. This condition affects men much more often than women. Parkinsonism is usually the first sign of X-linked dystonia-Parkinsonism. Thus, the miRNAs shown here in the context of X chromosome in PD can serve as valuable markers in predicting the early onset of disease or manifestation of symptoms of X-linked dystonia-Parkinsonism.

## Discussion

Although the identification of molecular targets from unbiased genome-wide association studies performed on various NDs has contributed to a considerable development of therapeutic target discovery [101], yet the picture on therapeutics for all four NDs studied herein is far from complete, as there is paucity of data on the regulatory small RNAs that control the mRNA expression, and data integration between miRNA and the RNAseq data from multiple NDs. Despite these challenges, the small RNAs offer significant advantages, the main being their size that makes them attractive therapeutic and diagnostic targets, along with the existence of chemistries that can be designed in quick time and can be used in targeting and inhibiting miRNAs in a safe manner. Therefore, we have attempted to perform the first global study on miRNAs, to provide insights into intrinsic relationship of miRNA with neurodegenerative diseases and create a foundation for the first miRNA database on NDs. Above all, the perspective gained from performing this study has revealed great potential for diagnostic and prognostic tests to be developed from circulating miRNAs based on miRNAs dysregulated in a specific ND or across multiple NDs. Interestingly, our study has been able to demonstrate that despite the differential expression profiles being largely unique to each ND, they also converge on highly relevant pathways pertinent to NDs with strong corroboration and high statistical significance, thereby providing the first snapshot of the panoramic landscape of miRNA in the context of NDs. Each of these convergent biological functions is demonstrably relevant to multiple facets of neurodegeneration across each ND profiled in this study, and a deeper understanding of this regulatory machinery will guide us to the new generation of therapeutic modalities.

### First Steps Towards Curation of a MiRNA Database for AD, PD, ALS, and MS

The diagnosis and therapy of neurodegenerative diseases that afflict humans requires the identification of novel molecular targets. However, caution is advised when pursuing miRNAs in body fluids as miRNA levels have been known to vary in response to both change in diet [102] and exercise [103, 104]. There are also many other chronic and acute conditions that may produce changes in body fluid miRNAs such as infection with various pathogens, or cancer. For these reasons, longitudinal studies of ND patients will likely be required to confirm the validity of any biomarker candidates put forth by this study.

To date, the four NDs studied herein have not been visualized together in order to critically understand not only the differences between them, but also the key intersections which functionally unify them. This is the first study to have assembled a knowledge-based database of miRNA which shows the roadmap for creating a curated database for miRNAs derived from patients with NDs. The ND-miRNA knowledge-based data (Supplementary Table 1) offers a number of new ways to utilize existing biomarker profiles from multiple NDs. Firstly, several studies can be used for cross-comparing and corroborating findings in the same disease, or across multiple diseases, body fluids/cell types, and other anatomic compartments. Currently, there is no such platform to do this for multiple NDs using miRNAs.

Through this combined analysis, several novel aspects have emerged that will change the way we visualize NDs in humans, and this study provides a panoramic view of these aspects which underpins the value of miRNA in understanding the biological processes that underlie human NDs. Further, this database shows immense potential for biomarker discovery for NDs, as we were able to identify several disease—and compartment (cell and body fluid)—specific miRNA signatures assembled from small to large study cohorts, and more importantly the miRNA candidates that unify all four NDs, which is unique to this study. These cross-comparisons of diverse compartments were reassuring, as a significant number of miRNAs analyzed, to date, from human ND patients emanated from the serum and plasma, owing to the ease of acquiring them. However, CSF remains the gold standard for understanding NDs, because of its constant contact with the brain and peripheral neural tissues. Although several studies with CSF miRNA exist, there is a bigger need for CSF-based or autopsied brain tissue-derived miRNA to be analyzed and added to the database. Further, there is a need for unannotated miRNA to be added for the evolution of this database.

It should be emphasized that to truly utilize this type of data for diagnostic purposes, curation of a miRNA database is needed which will require a global effort from various scientific groups, along with the integration of artificial intelligence (AI) and machine learning tools for developing algorithms that will be able to recognize disease-specific signatures, patterns, and biomarkers of value that can translate into sensitive diagnostics and specific therapeutics. This has recently been realized in the form of a diagnostic entity for ovarian cancer-based on miRNA expression data from serum. The algorithm developed was capable of identifying ovarian cancer with absolute specificity, and was able to do this regardless of patient age, histology, or disease stage [105]. Notably in our study, we have also uncovered miRNA specific to all four NDs regardless of age, sex, and disease staging. Furthermore, a breath biopsy has also been able to diagnose 17 different diseases (two of which are subtypes of PD) with 86% accuracy using an artificially intelligent nanoarray [106]. We emphasize that the validation for bioinformatic-based observations, further experimental assays, and longitudinal analysis of both small RNA and mRNA is sorely needed.

### MiRNA Compartmentalization and Functional Convergence of MiRNA Across NDs

The common pathways targeted by miRNAs across NDs are highly relevant to neurodegenerative processes. For instance, the extracellular matrix (ECM) was consistently highlighted in the heatmap analyses and is a fundamental part of neural tissue, in particular cellular architecture, synapse construction, and stabilization. They are also important regulators of neuroplasticity and memory formation/retention [107]. Of particular relevance are heparan sulfate proteoglycans (HSPGs), a family of glycoproteins with a small heparan sulfate side chain. HSPGs can be attached directly to the ECM, secreted into the extracellular space, or on secreted vesicles [108]. HSPGs are found throughout neural ECM and have been found localized with Aβ amyloid plaques and neurofibrillary tangles in AD patients [107], having potentially been secreted by activated glial cells [109]. HSPGs have also been associated with ALS pathology [110], PD pathology [111], and MS pathology [112]. Furthermore, HSPGs are involved in blood brain barrier (BBB) organization [113] and control the ability of monocytes to cross the BBB [114]. Adherens junctions are also involved in maintaining the structure of the BBB. They primarily control vascular permeability [115] and are an absolute requirement for vascular homeostasis in the brain [116]. Adherens junction dysfunction has been described in AD [117], PD [118], and MS [119]. Taken together, these findings suggest that a loss of intercellular structural components result in the dysfunction and death of neural tissues. Several other functional pathways were also converged upon and are described in subsequent sections. A causational relationship between differential miRNA expression identified in body fluids and these features of neurodegeneration has yet to be conclusively demonstrated however, and must be resolved with further study.

### What Guides Functional Convergence of MiRNAs?

The data in Fig. 8 clearly show that functional convergence of miRNA, which is evolutionally conserved, is guided by the functional conservation of sequence homologies between diverse miRNAs. This multiple sequence alignment (MSA)-based approach is unbiased and supports the view that the convergence seen in the miRPath analysis is not merely chance or an artifact of biases in the miRPath algorithm, previously cautioned by Godard & van Eyll [120]. These authors noted several KEGG pathways that are statistically significantly associated with random sets of input miRNAs, thanks to the preponderance of cancer pathways in the KEGG database. In our analysis, these pathways rarely emerged as statistically significant and if they did, they were excluded from further analyses. Furthermore, the current version of miRPath (version 3.0) has been redesigned to better account for these biases [18].

Alternatively, it is important to also refer to the promoters from which the miRNAs are being transcribed, and it is plausible to hypothesize that shared promoters confer common functions. This is the case with miRNA clusters which are usually found between 1 and 3 kbps apart, are often transcribed at the same time and share evolutionally conserved targets and biological functions [121, 122]. Unfortunately, this was less consistent than the miRNA families such as the miR-15 family and the miR-30 family. Unfortunately, only one complete miRNA cluster was detected in this analysis (the miR-23/24/27 cluster; Supplementary Table 2). Other miRNAs in Supplementary Table 1 belong to miRNA clusters, but not all members of these clusters are reported as differentially expressed in any of the NDs assessed in this study. This raises questions regarding the detection of some miRNA cluster members over others (as discussed in the following section). There is currently nothing in the literature that explains why one miRNA from a cluster may become differentially expressed while other members do not, despite being under the transcriptional control of the same promoter.

### MiRNA Clusters Indicate MiRNA Partnership in Neurodegenerative Pathogenesis

We know that miRNA families carry out synergistic functions in tandem, but the miRNA clusters have largely been overlooked in the functional context. Disparate miRNA can unite to carry out function or work in partnership in regulating mRNA. For instance, the miR-23/24/27 cluster has shown to be implicated across a range of neurodegenerative and neurological disorders. For example, hsa-miR-23 is associated with ALS muscle pathology via the mitochondrial protein PGC-1α and has been suggested as a target for future therapeutics [123]. Hsa-miR-24 and hsa-miR-27 have been similarly implicated in both normal muscle development and ALS pathology [124], and hsa-miR-27 has been linked to PD pathogenesis, also via a mechanism revolving around mitochondrial dysfunction [125]. MiR-27 is also associated with AD as it has been discovered in the hippocampus of AD patients. Hsa-miR-23 and hsa-miR-27a are also dysregulated in a rat model of epilepsy [126]. In light of our data as well as these previous studies, it is likely that the miR-30 family (as discussed in the earlier) and the miR-23/24/27 clusters are intimately associated with neurodegenerative processes in multiple NDs. Further study is required to test this hypothesis.

### Evidence of Prion Disease Pathway Common to the NDs

One of the unique findings in our study was the involvement of the prion disease pathway across all four NDs. Prion disease can be caused by either infectious proteins or mutations to the PRNP gene in humans. They are progressive neurodegenerative diseases resulting in symptoms similar to other dementias such as AD or Lewy body dementia. The function of normal prion protein is not fully described, but there is evidence that it is an important component of neuronal proliferation, differentiation, and formation of intercellular contacts required for efficient signal transmission [127]. The prion disease pathway is one of the most consistently represented, highly statistically significant functional pathways in our analysis. This was unexpected given that miRNA signatures for these diseases were often derived from animal models or neural tissue samples and were therefore excluded from this analysis. When looking at the signatures put forth in prion-focused literature [128–133], there was substantial overlap with the miRNAs reported in this analysis (Supplementary Table 1), in spite of the observation that tissue miRNA profiles and body fluid miRNA profiles overlap poorly [128].

For example, hsa-miR-146a is recorded as dysregulated in AD, PD, and MS (Supplementary Table 1), and has been associated with "Creutzfeldt-Jakob disease (CJD) in various brain regions [128], as well as in various other human prion diseases [134] and mouse models of prion diseases [129]. MiR-26a is also associated with AD, PD, and ALS [129]. Fifty-seven percent of all miRNAs reported as dysregulated in prion disease are also found in this analysis (Supplementary Table 1). Hsa-let-7i, hsa-miR-424, and hsa-miR-128 are also found in this analysis and within prion literature, but lack of either isoform (a, b, etc.) or the -3p/-5p annotation meant that these miRNAs could not be directly linked in both prions and the knowledge base presented in this study. This highlights the need to be consistent in reporting miRNA identities to facilitate construction of the most accurate biological pathways possible. Where relevant, the genomic location identifier should also be included. Without isoform, -3p/-5p notation or genomic location features accounted for, it becomes impossible to correctly identify potential cognate target mRNAs or to map a mature miRNA transcript back to the genomic loci from which it originated.

Overall, the evidence presented here based on miRNA profiles strongly implicates the prion pathway in other neurodegenerative diseases, and potentially without mutations to the PRNP gene. Interestingly, there were more upregulated miRNAs targeting this pathway than downregulated miRNAs (Table 1), which suggests that overall this pathway may be actively repressed in NDs. This assertion must be experimentally validated, but if it can be proven then it will greatly aid in elucidating the function and dysfunction of this protein and clarify the associating pathway. There is also a strong precedent for prion-like protein aggregation causing NDs such as the Aβ plaques in AD or the synuclein deposition seen in PD [135] and TDP-43, FUS, and SOD-1 in ALS [179], which are RNA-binding proteins having a tendency to aggregate. These are not always caused by mutations within these genes themselves. In fact, mutations to either of these genes are only associated with the familial forms of the disease which account for 10 to 15% of PD [136] and less than 1% of AD [137]. This may prove to be the case with prion proteins as well as they may be involved in the disease process without being the underlying genetic cause.

Exosomes can have a considerable contribution, and have been found to carry prion-like proteins, α-synuclein in PD and SOD1 in fALS [138]. Exosomes are also enriched with mRNA, small species of RNA including miRNA [139], and these miRNA can be functionally transferred to neural tissue. For example, it has recently been shown that macrophages differentiate to a pro-inflammatory phenotype after phagocytosing dorsal root ganglia exosomes enriched with miR-21-5p [140]. During neurodegeneration, exosomes, miRNAs, and aggregate proteins may also participate together to spread neurodegenerative signals throughout neural tissues, especially given the inclusion of a substantial amount of exosomal miRNA literature in this study.

### Fatty Acid Metabolism as One of the Significant Pathways in NDs

Our analysis revealed that fatty acid synthase (FASN) is targeted by six of the miRNAs in Table 1 alone, and many more across the entire ND-miRNA knowledge base (Supplementary Table 1). Previous studies concur with this observation on fatty acid metabolism defects being spread widely across many NDs, including ALS [141], PD [142], AD [9, 143], and MS [144]. However, fatty acids have been seen to both ameliorate and exacerbate neurodegenerative processes [145], and there is currently no consensus on which processes are helpful and which processes are harmful, or if there are any common pathways amongst NDs. However, mutations in this gene have been reported in epileptic encephalopathies by disruption of synaptic transmission [146]. It has also recently been shown that FASN interacts with protrudin to facilitate protrusion formation which is an essential step in neuron development [147]. It has also been shown that inhibition of FASN suppresses mitochondrial dysfunction in a mouse model of PD [148]. This is particularly relevant as most of the miRNAs that target FASN in our analysis are downregulated, which would presumably disinhibit FASN, promoting neural toxicity. The true role of FASN in NDs however must be resolved experimentally as it is currently lacking thorough investigation in any of the NDs analyzed here.

### Differentially Expressed MiRNAs May Lead to Dysfunctional Hippo Signaling in NDs

As mentioned earlier, the Hippo signaling pathway is also strongly implicated in several NDs, and was highlighted in our analysis. It is known to be extensively dysregulated in cancer, generally becoming hypoactive allowing its interacting partners YAP/TAZ to translocate to the nucleus and induce expression of cell growth-promoting genes [149]. However, in the case of neurodegenerative disease, the Hippo signaling pathway can become hyperactive. This can impair a tissue’s ability to regenerate given the overall decrease in proliferation signals that would be received through the Hippo signaling pathway in homeostatic conditions [83]. This process has been elucidated in AD [150, 151], fALS [152, 153], and most recently in HD [154]. As most of the miRNAs that target this pathway are being lost according to Table 1, this could potentially allow hyperactivation of the Hippo signaling pathway and result in decreased proliferation and anti-apoptotic gene expression as a general feature of NDs.

However, there are also a number of other Hippo signaling targeting miRNAs that are increased in multiple NDs. This is also occurring across the other convergent pathways discussed thus far. This feature of convergent pathways raises the question of whether these dysregulated miRNAs are a cause of pathology common amongst NDs, or if these miRNAs are becoming dysregulated in order to resist pathological changes that result in impaired neuron function and survival. Unfortunately, this is a question that can only be resolved experimentally, but the information presented here offers a strong foundation for answering this question.

### The Highly Convergent Targets of ND-Associated MiRNAs Are Associated with Immune Signaling, Apoptosis Pathways, and Proteostasis

The 105 highly convergent target genes supported many of the findings made using DIANA tools, but also highlighted some other highly disease relevant pathways (Supplementary fig. 1*,* Supplementary fig. 4). Of particular interest from this analysis is the discovery of IL-6 signaling pathways being targeted by multiple miRNAs across each ND analyzed in this study. This pathway is highly implicated for its many pleiotropic effects in the nervous system in several NDs [155]. This analysis has not helped elucidate the function of IL-6 in NDs, but it does place miRNA dysregulation at the root of IL-6 dysfunction in NDs. Analysis of the miRNAs that target this pathway may also be analyzed and tracked longitudinally using patient body fluid samples. This may provide more biological information to fully understand the paradoxical neurotropic and neurodegenerative effects of IL-6 in the nervous system, with further study.

A similar story unfolded regarding other signaling pathways, such as IL-4 and IL-13, IL-3, IL-10, IL-12, and IL-7, which are also likely to be affected across multiple NDs. Each of these cytokines have their own associations with neurodegenerative processes and seem to elicit neuroprotective or neurodegenerative effects depending on circumstance [156–159]. Furthermore, many downstream interacting partners in the signal transduction pathway for these cytokines were targeted by multiple ND-associated miRNAs, for example, STAT3. This may alter the way cells respond to cytokine stimuli, tipping the outcome towards a neuroprotective or neurodegenerative effect. Further research is required to test this hypothesis.

Another biological pathway that is likely to be affected by the differentially targeted 105 mRNAs is the intrinsic apoptotic pathway. This pathway is reliant upon mitochondria-initiated processes [160], and mitochondrial dysfunction is a well-established feature of neurodegenerative diseases, especially those associated with aging (PD and AD) [7, 161]. Furthermore, two protein metabolism procedures were statistically significant in the Reactome analysis (supplementary fig. 4), the TRiC/CCT pathway, and the protein SUMOylation pathway. TRiC/CCT is heavily involved in maintaining proteostasis (normal protein folding) and prevents formation of neurotoxic protein aggregates, an established feature of AD, PD, and ALS with limited evidence for this type of mechanism occurring in MS also [162]. SUMOylation is also associated with the formation of toxic protein aggregates in NDs, as well as mitochondrial dysfunction, oxidative stress, and RNA metabolism [163]. It must be experimentally confirmed that diverse miRNAs converge on these genes/pathways in appropriate disease models, but the evidence presented here suggests that their involvement is likely.

Given that these mRNAs are all biologically confirmed targets of ND-associated miRNAs, it is highly likely that these genes are involved in initiating or driving the core pathologies in each of the NDs profiled in this study. Further analysis of these genes in disease models and patient tissue samples is essential to definitively show them in this role.

### DNA Methylation and MiRNA Dysregulation

DNA methylation may be linked to differential miRNA expression via the human leukocyte antigen (HLA) genes, which produce major histocompatibility complexes (MHC) required for adaptive immune responses. It has recently been determined that DNMT1 (a DNA methyl-transferase) regulates the expression of MHC class 1 in neurons [170], which is known to become frequently upregulated in neurodegenerative processes. This has been shown to exacerbate neuronal cell death in PD [171], and to ameliorate motor neuron toxicity in ALS [172, 173]. Differential expression of MHC expression is important to miRNA expression because a number of miRNAs have been discovered in intronic regions of the HLA genes. These miRNAs are almost all novel, but they are often found at known mutation sites that are associated with disease phenotypes. Several of these miRNAs are highly homologous to miRNAs associated with NDs in Supplementary Table 1 [174]. These miRNAs are hsa-miR-143, hsa-miR-196b, and hsa-miR-590. Each of these miRNAs has been reported as downregulated in a ND suggesting that differential methylation and expression of HLA genes may give rise to some of the miRNA dysregulation seen in multiple NDs.

Another manner in which miRNA dysregulation may occur is through differential methylation of the genomic loci from which these miRNAs originate. Differential methylation is the addition of a methyl residue to a cytosine in the DNA strand (often in the gene promoter region), which makes transcription from this site highly unlikely. DNA methylation is strongly associated with neurological development throughout the central nervous system [175], and mutations in the genes responsible for DNA methylation have also been associated with a great many neurological deficiencies and disease states (reviewed in [176]). It has also been demonstrated that DNA methylation patterns are similar between different NDs [177]. Unfortunately, there are very few studies that have focused on methylation of miRNA sites. However, one such study has shown that hsa-miR-9, hsa-miR-181c, hsa-miR-124, hsa-miR-146b, and hsa-miR-451 are differentially hyper methylated in AD [178]. This is congruous with the observations made in our study, as these miRNAs were frequently downregulated in whatever ND/compartment they were recorded in. Methylation patterns are also inheritable [179], which offers an enticing explanation for the NDs studied here which have familial associations despite the absence of canonical genetic mutations. Taken together, this evidence suggests it is well worth the effort to catalog the differential methylation in miRNA gene loci in order to reveal new modalities of disease initiation and progression for NDs. In so doing, we may reveal new patient classifying mechanisms and/or therapeutic targets.

### Genomic Loci: Indicators of MiRNA Dysregulation in NDs

The most fundamental element to understand when attempting to determine the source of miRNA dysregulation is the genomic loci of the miRNA genes involved. This will yield vital information regarding the promoter regions and potentially transcription factors that enhance or inhibit transcription from these sites. Unfortunately, the factors which govern miRNA transcription are not studied as deeply as the results of miRNA function and dysfunction [166]. However, tools are now emerging capable of predicting miRNA transcription factor functions [167] and promoter locations [168, 169]. This requires a great deal of extra study as to our knowledge, there are no publications regarding transcriptional control of miRNAs in any of the NDs analyzed in this study. As a first step towards this, we have analyzed the genomic loci from which dysregulated miRNAs are being transcribed.

A key finding of this study was that miRNAs located on chromosome 17 are over-represented across all four NDs. Apart from this, our study also shows the miRNA genes for AD were highly enriched on chromosome X, which is consistent with literature where AD has been shown to be X-linked. Our study was able to show the role of miRNAs that regulate these processes and could be exploited as disease biomarkers. Analysis of the genomic location of the dysregulated ND-associated miRNAs revealed a number of relationships between the four NDs included in this study. Firstly, the chromosomal location suggests that not all miRNA containing genomic loci are affected equally by NDs. For instance, chromosome 7 is responsible for a large number of dysregulated miRNAs specifically associated with AD (66% of dysregulated miRNAs located on this chromosome are associated with AD). Conversely, chromosome 14, chromosome 17, and chromosome 19 located miRNAs are all broadly involved with all four NDs analyzed in this study. In fact, approximately 45% of the miRNA genes were found to be located on chromosome 17 were dysregulated across the four NDs. This suggests that there may be some very broad and common regulatory shifts in miRNA transcription during neurodegeneration.

### Hsa-miR-30b-5p: a Unifying Feature of ALS, AD, PD, and MS

We believe that the network-driven neuronal vulnerability is key to understanding these diseases [164], as many of the regions involved in the manifestation of MS, AD, PD, and ALS are juxtaposed. Therefore, it is plausible to say that the understanding of common molecular and biological intersections that unify all NDs may give vital clues to underlying mechanisms in neuronal degeneration. In this context, the most unique finding of this study was the identification of hsa-miR-30b-5p, which was dysregulated in the four NDs analyzed in this study. To our knowledge, this is the first such miRNA that unifies all four NDS and could be a possible biomarker, as it encompasses some of the most relevant pathways that underlie neurodegeneration. This miRNA has also been shown to be highly abundant in the human neocortex alongside other members of the miR-30 family [165]. Moreover, hsa-miR-30b-5p has also been shown to correlate with neurofibrillary tangles in the CSF of patients with AD and PD, along with a significant fit across increasing plaque density stages [38]. This miRNA and the family to which it belongs are all worthy of further research aimed at elucidating disease driving factors across each of these NDs.

## Conclusions

The critical molecular insights gained through the discovery of ND-associated miRNAs, overlapping miRNAs and the functional convergence of miRNAs on vital pathways strongly implicated in neurodegenerative processes can prove immensely valuable in the identification of a new generation of biomarkers, along with the exploitation of miRNAs as therapeutics to treat these diseases.

## Electronic Supplementary Material

Supplementary Figure 1 (PDF 463 kb)

Supplementary Figure 2 (PDF 1509 kb)

Supplementary Figure 3 (PDF 1917 kb)

Supplementary Figure 4 (PDF 23413 kb)

Supplementary Table 1 (PDF 310 kb)

Supplementary Table 2 (PDF 242 kb)

Supplementary Table 3 (DOCX 36 kb)

## Abbreviations

ND: Neurodegenerative disease
AD: Alzheimer’s disease
PD: Parkinson’s disease
ALS: Amyotrophic lateral sclerosis
MS: Multiple sclerosis
SCA: Spinocerebellar ataxia
SMA: Spinal muscular atrophy
HD: Huntington’s disease
FTD: Frontotemporal dementia
3′ UTR: Three prime untranslated region
RISC: RNA-induced silencing complex
MSA: Multiple sequence alignment
CSF: Cerebrospinal fluid
WBC: White blood cell
PBMC: Peripheral blood mononuclear cell
DE: Differentially expressed
HSPG: Heparan sulfate proteoglycan
qPCR-: Quantitative polymerase chain reaction
NGS: Next generation sequencing
TLDA: Taqman low-density arrays
ddPCR: Digital droplet PCR
GWAS: Genome-wide association study
BBB: Blood brain barrier
CJD: Creutzfeldt-Jakob disease
MHC: Major histocompatibility complex
RBP: Ribosome-binding protein
MRE: MicroRNA response element
