# Supplementary figures and images for: Panoramic Visualization of Circulating MicroRNAs Across Neurodegenerative Diseases in Humans
Source: Mol Neurobiol. 2019 Apr 29;56(11):7380–407. doi: 10.1007/s12035-019-1615-1 (PMC6815273; doi:10.1007/s12035-019-1615-1)

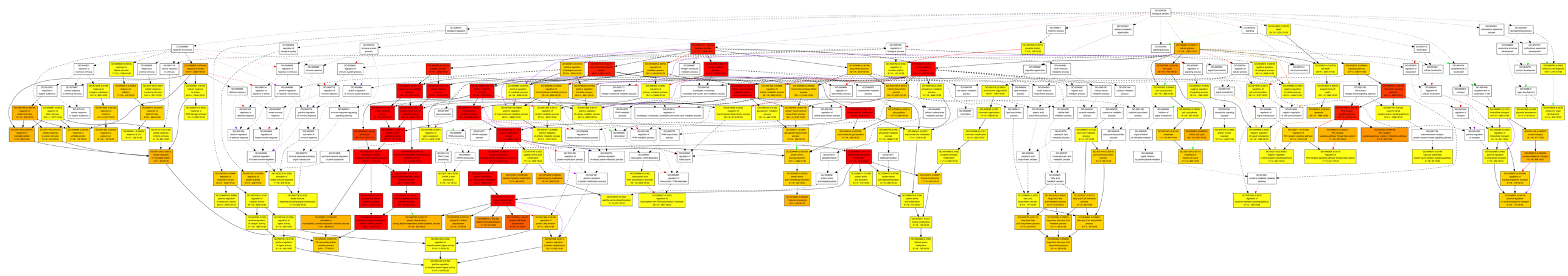

Supplement: Supplementary file 1 — (PDF 463 kb) [file 12035_2019_1615_MOESM1_ESM.pdf]
