# Supplementary material for: Panoramic Visualization of Circulating MicroRNAs Across Neurodegenerative Diseases in Humans
Source: Mol Neurobiol. 2019 Apr 29;56(11):7380–407. doi: 10.1007/s12035-019-1615-1 (PMC6815273; doi:10.1007/s12035-019-1615-1)
Supplement: Supplementary file 2 — (PDF 1509 kb) [file 12035_2019_1615_MOESM2_ESM.pdf]

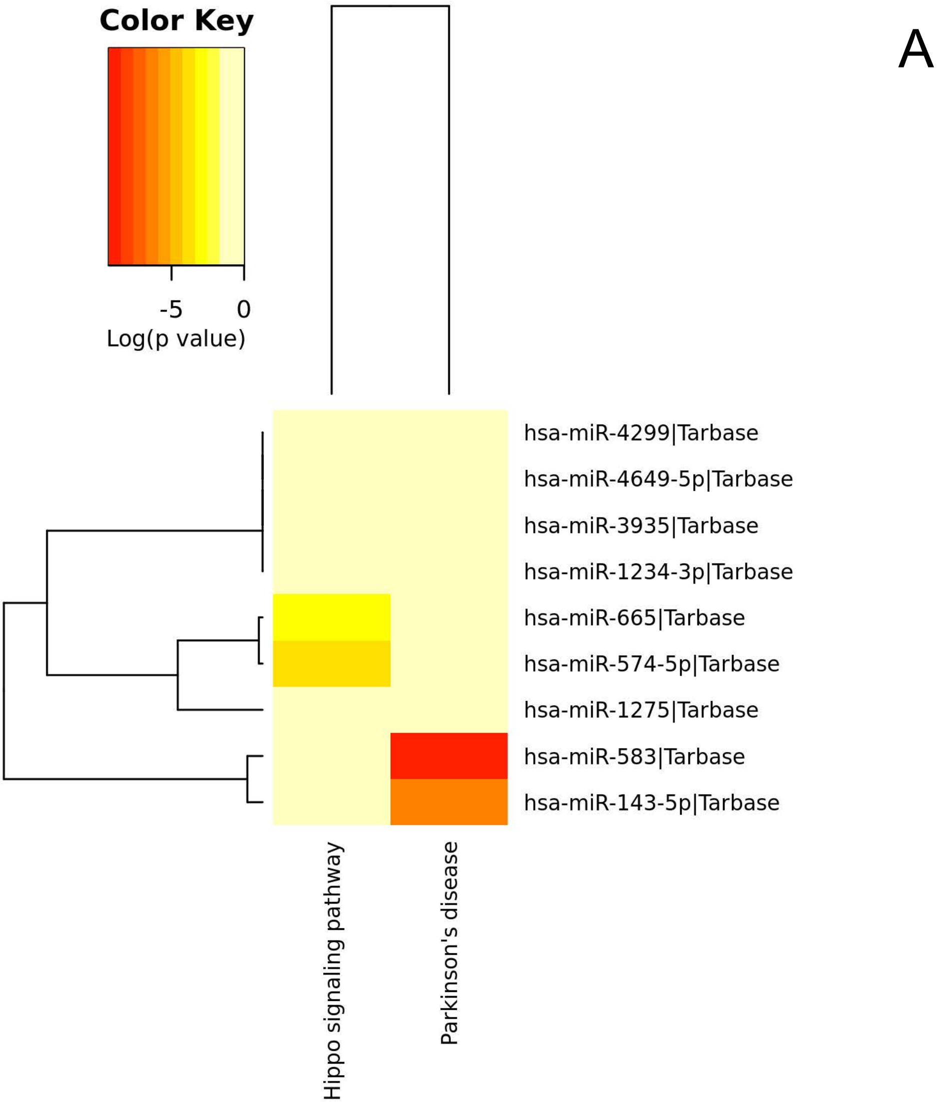

Panoramic visualization of circulating microRNAs across neurodegenerative diseases in humans, Acta Neuropathologica, Samuel Brennan, Matthew Keon, Bing Liu, Zheng Su, Nitin Saksena, Neurodegenerative Disease section, Iggy Get Out, 19a Boundary Street, Darlinghurst NSW 2010. Sydney. Australia. E-mail: nitin@iggygetout.com

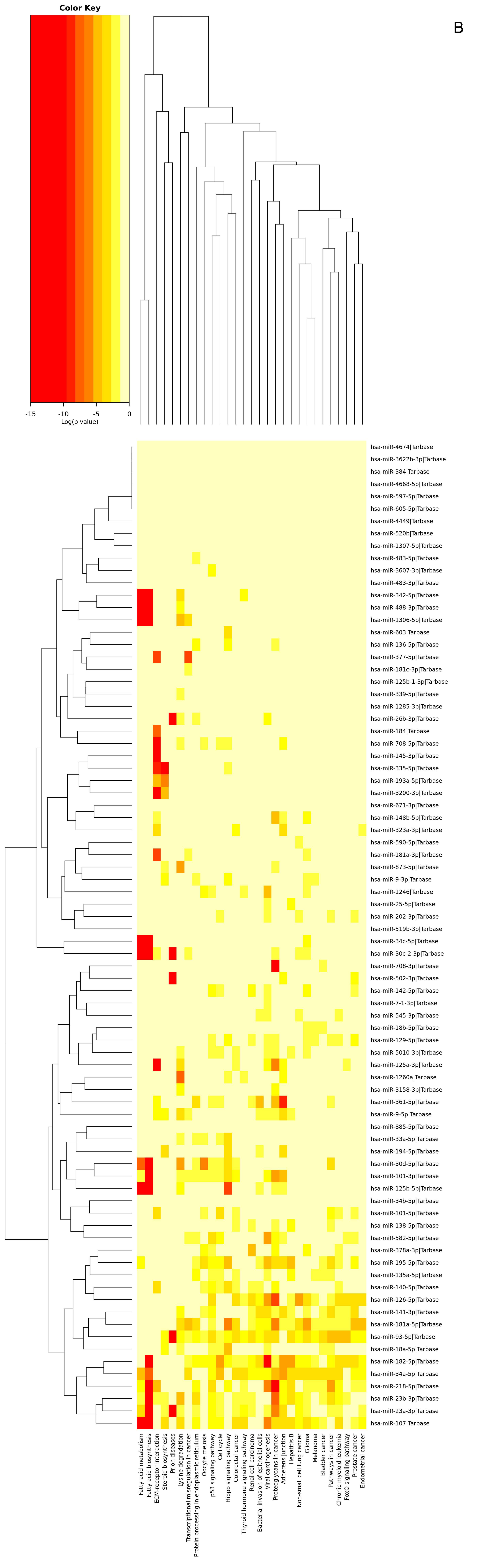
B
Color Key
-15
-10
-5
0
Log(p value)
hsa-miR-4674|Tarbase
hsa-miR-3622b-3p|Tarbase
hsa-miR-384|Tarbase
hsa-miR-4668-5p|Tarbase
hsa-miR-597-5p|Tarbase
hsa-miR-605-5p|Tarbase
hsa-miR-4449|Tarbase
hsa-miR-520b|Tarbase
hsa-miR-1307-5p|Tarbase
hsa-miR-483-5p|Tarbase
hsa-miR-3607-3p|Tarbase
hsa-miR-483-3p|Tarbase
hsa-miR-342-5p|Tarbase
hsa-miR-488-3p|Tarbase
hsa-miR-1306-5p|Tarbase
hsa-miR-603|Tarbase
hsa-miR-136-5p|Tarbase
hsa-miR-377-5p|Tarbase
hsa-miR-181c-3p|Tarbase
hsa-miR-125b-1-3p|Tarbase
hsa-miR-339-5p|Tarbase
hsa-miR-1285-3p|Tarbase
hsa-miR-26b-3p|Tarbase
hsa-miR-184|Tarbase
hsa-miR-708-5p|Tarbase
hsa-miR-145-3p|Tarbase
hsa-miR-335-5p|Tarbase
hsa-miR-193a-5p|Tarbase
hsa-miR-3200-3p|Tarbase
hsa-miR-671-3p|Tarbase
hsa-miR-148b-5p|Tarbase
hsa-miR-323a-3p|Tarbase
hsa-miR-590-5p|Tarbase
hsa-miR-181a-3p|Tarbase
hsa-miR-873-5p|Tarbase
hsa-miR-9-3p|Tarbase
hsa-miR-1246|Tarbase
hsa-miR-25-5p|Tarbase
hsa-miR-202-3p|Tarbase
hsa-miR-519b-3p|Tarbase
hsa-miR-34c-5p|Tarbase
hsa-miR-30c-2-3p|Tarbase
hsa-miR-708-3p|Tarbase
hsa-miR-502-3p|Tarbase
hsa-miR-142-5p|Tarbase
hsa-miR-7-1-3p|Tarbase
hsa-miR-545-3p|Tarbase
hsa-miR-18b-5p|Tarbase
hsa-miR-129-5p|Tarbase
hsa-miR-5010-3p|Tarbase
hsa-miR-125a-3p|Tarbase
hsa-miR-1260a|Tarbase
hsa-miR-3158-3p|Tarbase
hsa-miR-361-5p|Tarbase
hsa-miR-9-5p|Tarbase
hsa-miR-885-5p|Tarbase
hsa-miR-33a-5p|Tarbase
hsa-miR-194-5p|Tarbase
hsa-miR-30d-5p|Tarbase
hsa-miR-101-3p|Tarbase
hsa-miR-125b-5p|Tarbase
hsa-miR-34b-5p|Tarbase
hsa-miR-101-5p|Tarbase
hsa-miR-138-5p|Tarbase
hsa-miR-582-5p|Tarbase
hsa-miR-378a-3p|Tarbase
hsa-miR-195-5p|Tarbase
hsa-miR-135a-5p|Tarbase
hsa-miR-140-5p|Tarbase
hsa-miR-126-5p|Tarbase
hsa-miR-141-3p|Tarbase
hsa-miR-181a-5p|Tarbase
hsa-miR-93-5p|Tarbase
hsa-miR-18a-5p|Tarbase
hsa-miR-182-5p|Tarbase
hsa-miR-34a-5p|Tarbase
hsa-miR-218-5p|Tarbase
hsa-miR-23b-3p|Tarbase
hsa-miR-23a-3p|Tarbase
hsa-miR-107|Tarbase
Fatty acid metabolism
Fatty acid biosynthesis
ECM-receptor interaction
Steroid biosynthesis
Prion diseases
Lysine degradation
Transcriptional misregulation in cancer
Protein processing in endoplasmic reticulum
Oocyte meiosis
p53 signaling pathway
Cell cycle
Hippo signaling pathway
Colorectal cancer
Thyroid hormone signaling pathway
Renal cell carcinoma
Bacterial invasion of epithelial cells
Viral carcinogenesis
Proteoglycans in cancer
Adherens junction
Hepatitis B
Non-small cell lung cancer
Glioma
Melanoma
Bladder cancer
Pathways in cancer
Chronic myeloid leukemia
FoxO signaling pathway
Prostate cancer
Endometrial cancer

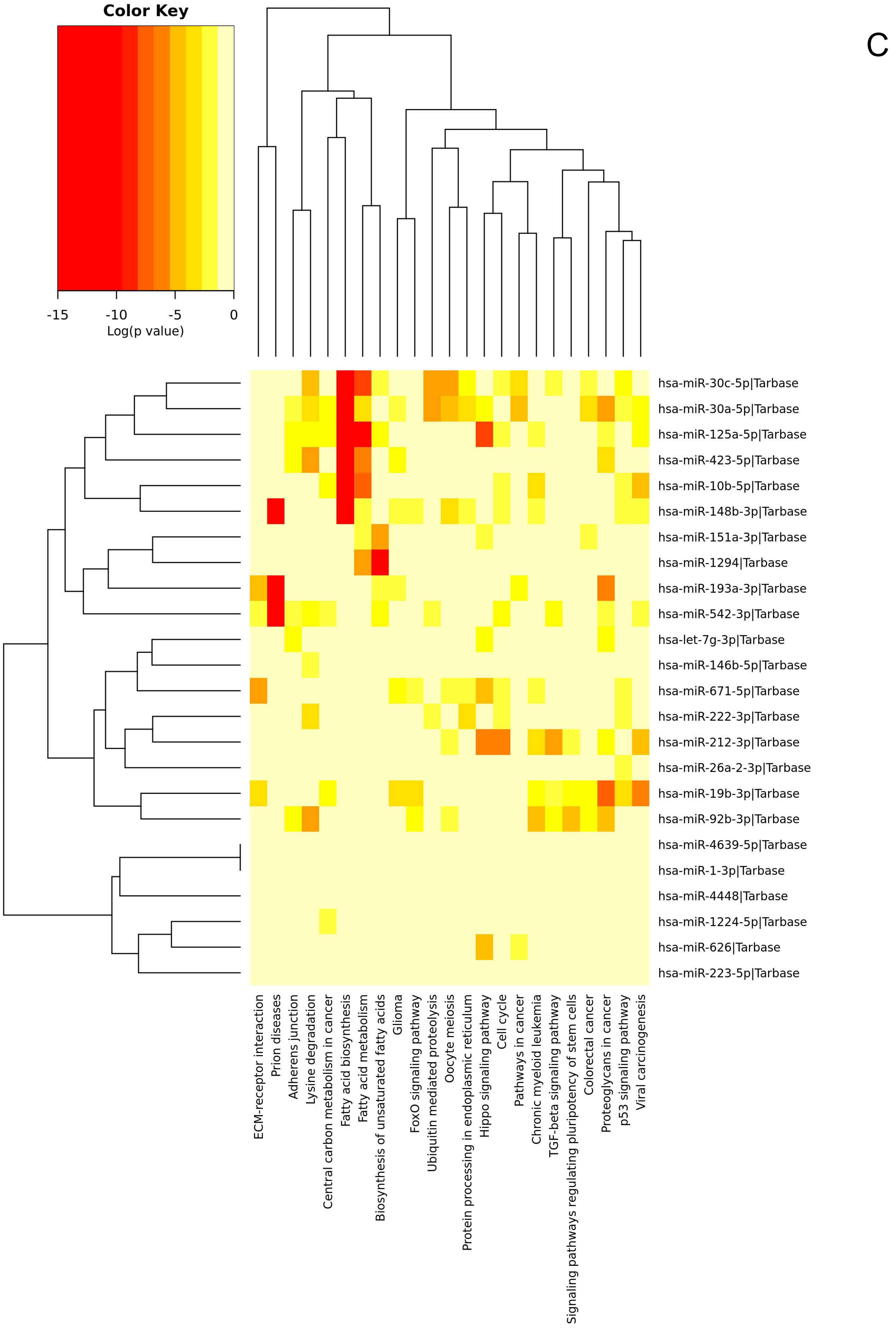
C
Color Key
-15
-10
-5
0
Log(p value)
hsa-miR-30c-5p|Tarbase
hsa-miR-30a-5p|Tarbase
hsa-miR-125a-5p|Tarbase
hsa-miR-423-5p|Tarbase
hsa-miR-10b-5p|Tarbase
hsa-miR-148b-3p|Tarbase
hsa-miR-151a-3p|Tarbase
hsa-miR-1294|Tarbase
hsa-miR-193a-3p|Tarbase
hsa-miR-542-3p|Tarbase
hsa-let-7g-3p|Tarbase
hsa-miR-146b-5p|Tarbase
hsa-miR-671-5p|Tarbase
hsa-miR-222-3p|Tarbase
hsa-miR-212-3p|Tarbase
hsa-miR-26a-2-3p|Tarbase
hsa-miR-19b-3p|Tarbase
hsa-miR-92b-3p|Tarbase
hsa-miR-4639-5p|Tarbase
hsa-miR-1-3p|Tarbase
hsa-miR-4448|Tarbase
hsa-miR-1224-5p|Tarbase
hsa-miR-626|Tarbase
hsa-miR-223-5p|Tarbase
ECM-receptor interaction
Prion diseases
Adherens junction
Lysine degradation
Central carbon metabolism in cancer
Fatty acid biosynthesis
Fatty acid metabolism
Biosynthesis of unsaturated fatty acids
Glioma
FoxO signaling pathway
Ubiquitin mediated proteolysis
Oocyte meiosis
Protein processing in endoplasmic reticulum
Hippo signaling pathway
Cell cycle
Pathways in cancer
Chronic myeloid leukemia
TGF-beta signaling pathway
Signaling pathways regulating pluripotency of stem cells
Colorectal cancer
Proteoglycans in cancer
p53 signaling pathway
Viral carcinogenesis

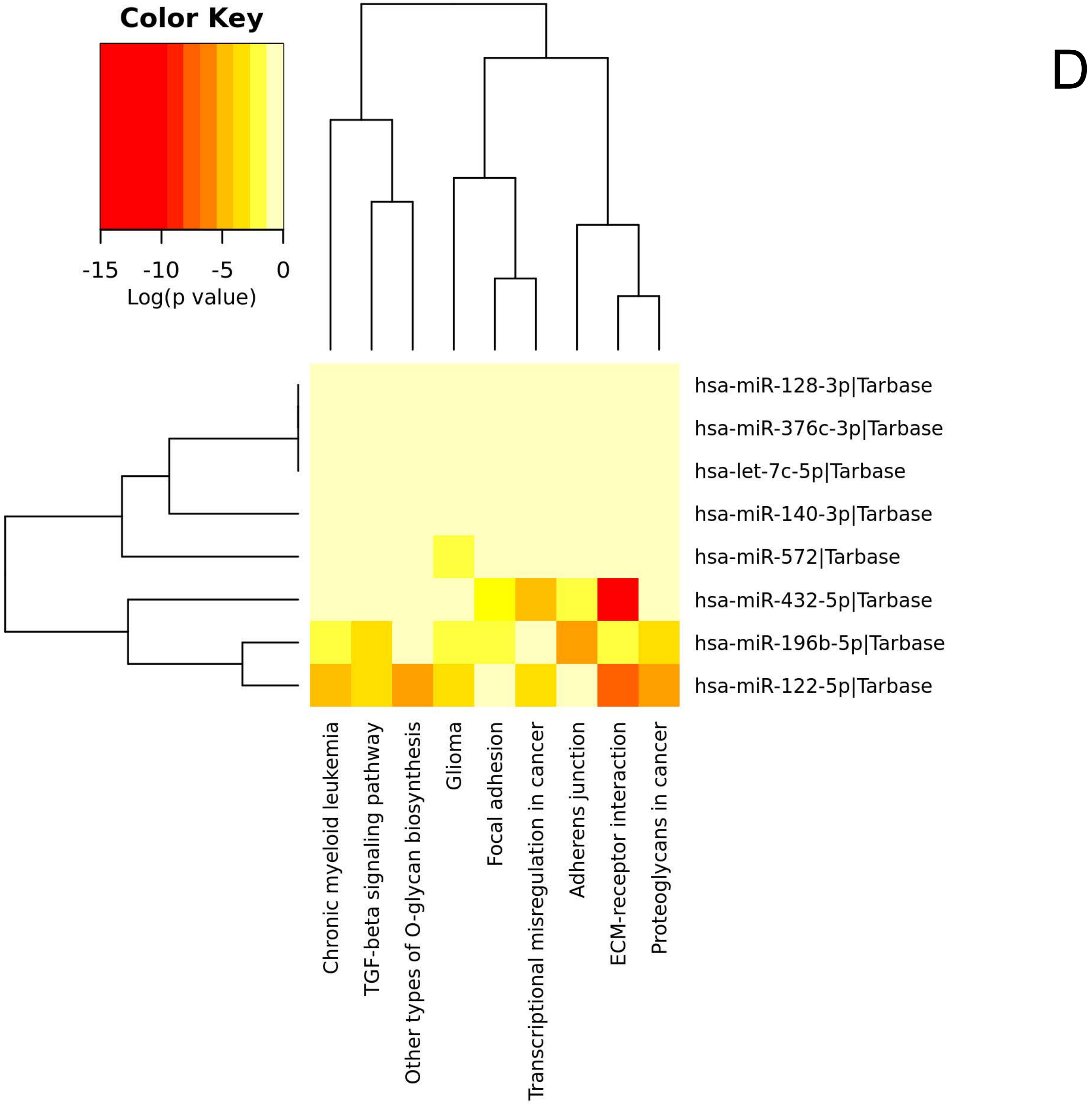
Color Key
-15
-10
-5
0
Log(p value)
D
hsa-miR-128-3p|Tarbase
hsa-miR-376c-3p|Tarbase
hsa-let-7c-5p|Tarbase
hsa-miR-140-3p|Tarbase
hsa-miR-572|Tarbase
hsa-miR-432-5p|Tarbase
hsa-miR-196b-5p|Tarbase
hsa-miR-122-5p|Tarbase
Chronic myeloid leukemia
TGF-beta signaling pathway
Other types of O-glycan biosynthesis
Glioma
Focal adhesion
Transcriptional misregulation in cancer
Adherens junction
ECM-receptor interaction
Proteoglycans in cancer
