# Supplementary material for: Panoramic Visualization of Circulating MicroRNAs Across Neurodegenerative Diseases in Humans
Source: Mol Neurobiol. 2019 Apr 29;56(11):7380–407. doi: 10.1007/s12035-019-1615-1 (PMC6815273; doi:10.1007/s12035-019-1615-1)
Supplement: Supplementary file 3 — (PDF 1917 kb) [file 12035_2019_1615_MOESM3_ESM.pdf]

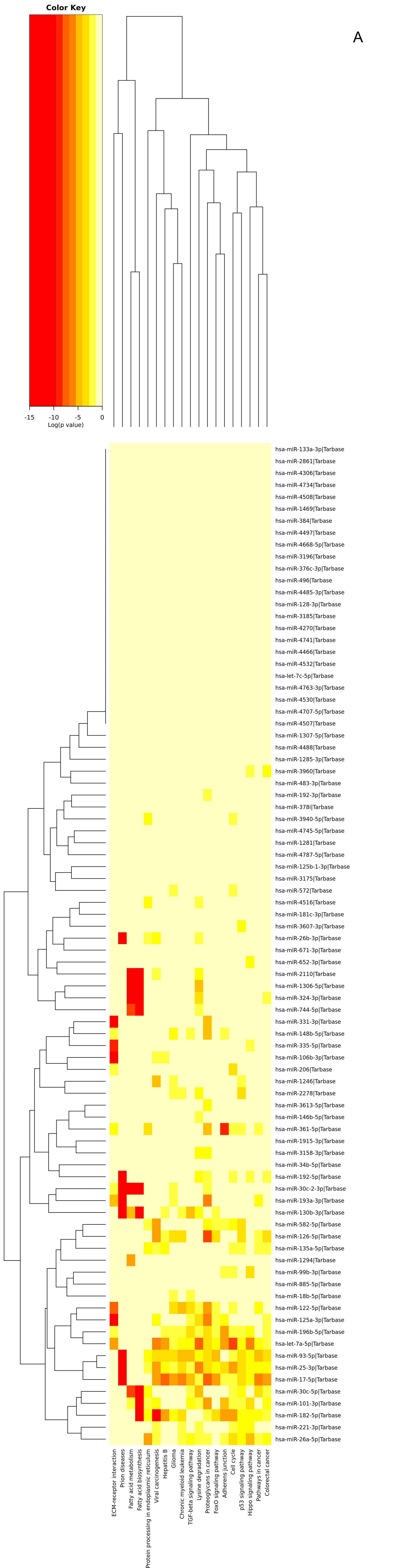


Panoramic visualization of circulating microRNAs across neurodegenerative diseases in humans, Acta Neuropathologica, Samuel Brennan, Matthew Keon, Bing Liu, Zheng Su, Nitin Saksena, Neurodegenerative Disease section, Iggy Get Out, 19a Boundary Street, Darlinghurst NSW 2010. Sydney. Australia. E-mail: nitin@iggygetout.com

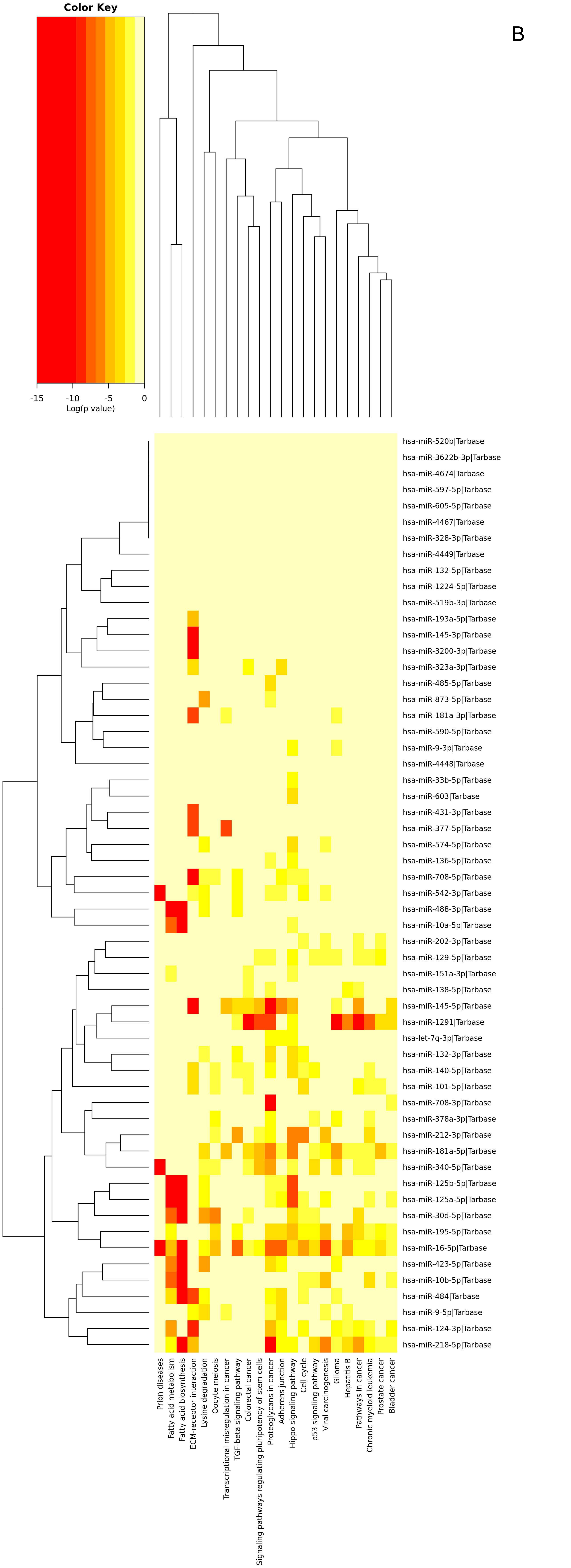
B
Color Key
-15
-10
-5
0
Log(p value)
hsa-miR-520b|Tarbase
hsa-miR-3622b-3p|Tarbase
hsa-miR-4674|Tarbase
hsa-miR-597-5p|Tarbase
hsa-miR-605-5p|Tarbase
hsa-miR-4467|Tarbase
hsa-miR-328-3p|Tarbase
hsa-miR-4449|Tarbase
hsa-miR-132-5p|Tarbase
hsa-miR-1224-5p|Tarbase
hsa-miR-519b-3p|Tarbase
hsa-miR-193a-5p|Tarbase
hsa-miR-145-3p|Tarbase
hsa-miR-3200-3p|Tarbase
hsa-miR-323a-3p|Tarbase
hsa-miR-485-5p|Tarbase
hsa-miR-873-5p|Tarbase
hsa-miR-181a-3p|Tarbase
hsa-miR-590-5p|Tarbase
hsa-miR-9-3p|Tarbase
hsa-miR-4448|Tarbase
hsa-miR-33b-5p|Tarbase
hsa-miR-603|Tarbase
hsa-miR-431-3p|Tarbase
hsa-miR-377-5p|Tarbase
hsa-miR-574-5p|Tarbase
hsa-miR-136-5p|Tarbase
hsa-miR-708-5p|Tarbase
hsa-miR-542-3p|Tarbase
hsa-miR-488-3p|Tarbase
hsa-miR-10a-5p|Tarbase
hsa-miR-202-3p|Tarbase
hsa-miR-129-5p|Tarbase
hsa-miR-151a-3p|Tarbase
hsa-miR-138-5p|Tarbase
hsa-miR-145-5p|Tarbase
hsa-miR-1291|Tarbase
hsa-let-7g-3p|Tarbase
hsa-miR-132-3p|Tarbase
hsa-miR-140-5p|Tarbase
hsa-miR-101-5p|Tarbase
hsa-miR-708-3p|Tarbase
hsa-miR-378a-3p|Tarbase
hsa-miR-212-3p|Tarbase
hsa-miR-181a-5p|Tarbase
hsa-miR-340-5p|Tarbase
hsa-miR-125b-5p|Tarbase
hsa-miR-125a-5p|Tarbase
hsa-miR-30d-5p|Tarbase
hsa-miR-195-5p|Tarbase
hsa-miR-16-5p|Tarbase
hsa-miR-423-5p|Tarbase
hsa-miR-10b-5p|Tarbase
hsa-miR-484|Tarbase
hsa-miR-9-5p|Tarbase
hsa-miR-124-3p|Tarbase
hsa-miR-218-5p|Tarbase
Prion diseases
Fatty acid metabolism
Fatty acid biosynthesis
ECM-receptor interaction
Lysine degradation
Oocyte meiosis
Transcriptional misregulation in cancer
TGF-beta signaling pathway
Colorectal cancer
Signaling pathways regulating pluripotency of stem cells
Proteoglycans in cancer
Adherens junction
Hippo signaling pathway
Cell cycle
p53 signaling pathway
Viral carcinogenesis
Glioma
Hepatitis B
Pathways in cancer
Chronic myeloid leukemia
Prostate cancer
Bladder cancer

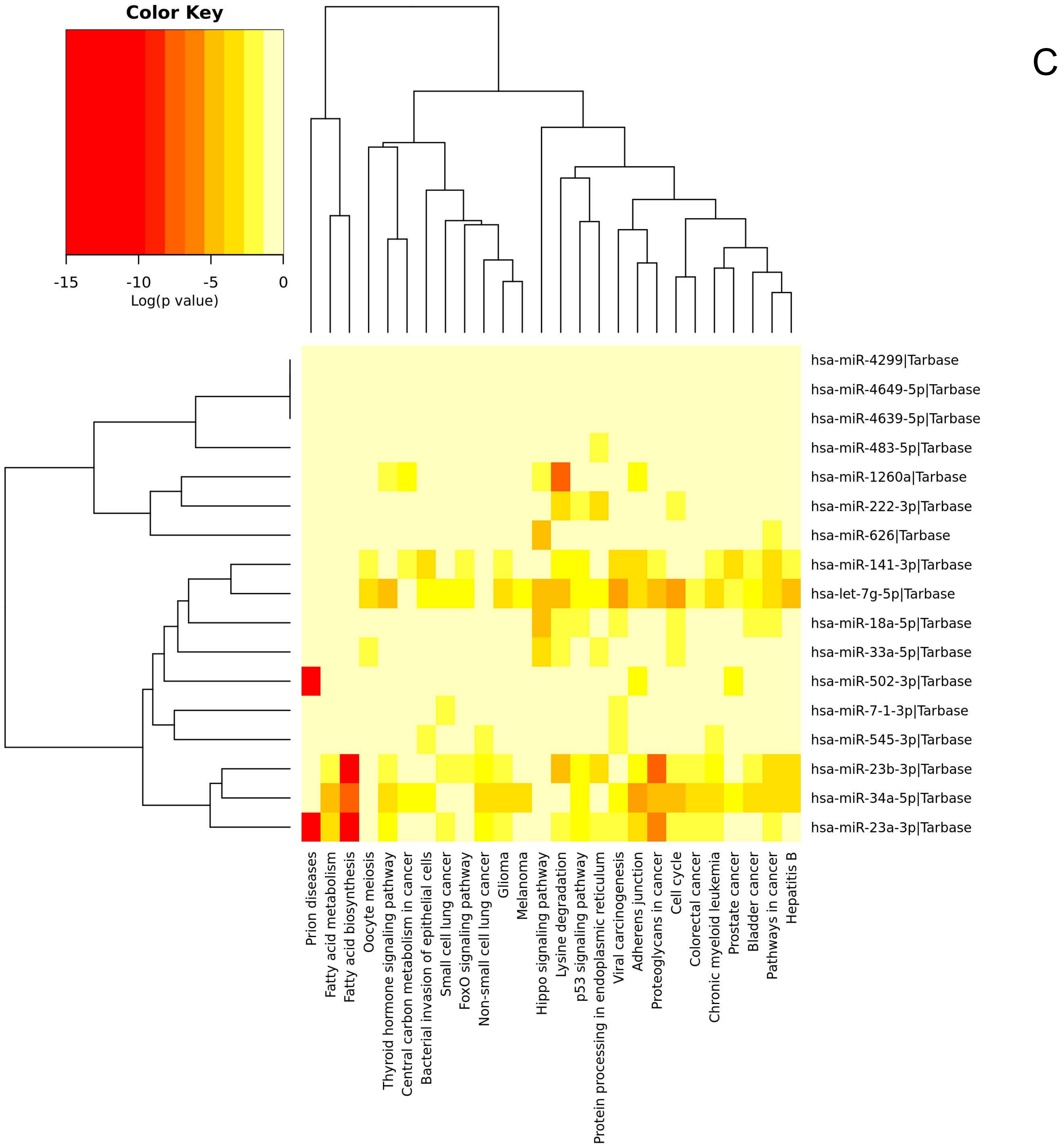
Color Key
-15
-10
-5
0
Log(p value)
C
hsa-miR-4299|Tarbase
hsa-miR-4649-5p|Tarbase
hsa-miR-4639-5p|Tarbase
hsa-miR-483-5p|Tarbase
hsa-miR-1260a|Tarbase
hsa-miR-222-3p|Tarbase
hsa-miR-626|Tarbase
hsa-miR-141-3p|Tarbase
hsa-let-7g-5p|Tarbase
hsa-miR-18a-5p|Tarbase
hsa-miR-33a-5p|Tarbase
hsa-miR-502-3p|Tarbase
hsa-miR-7-1-3p|Tarbase
hsa-miR-545-3p|Tarbase
hsa-miR-23b-3p|Tarbase
hsa-miR-34a-5p|Tarbase
hsa-miR-23a-3p|Tarbase
Prion diseases
Fatty acid metabolism
Fatty acid biosynthesis
Oocyte meiosis
Thyroid hormone signaling pathway
Central carbon metabolism in cancer
Bacterial invasion of epithelial cells
Small cell lung cancer
FoxO signaling pathway
Non-small cell lung cancer
Glioma
Melanoma
Hippo signaling pathway
Lysine degradation
p53 signaling pathway
Protein processing in endoplasmic reticulum
Viral carcinogenesis
Adherens junction
Proteoglycans in cancer
Cell cycle
Colorectal cancer
Chronic myeloid leukemia
Prostate cancer
Bladder cancer
Pathways in cancer
Hepatitis B

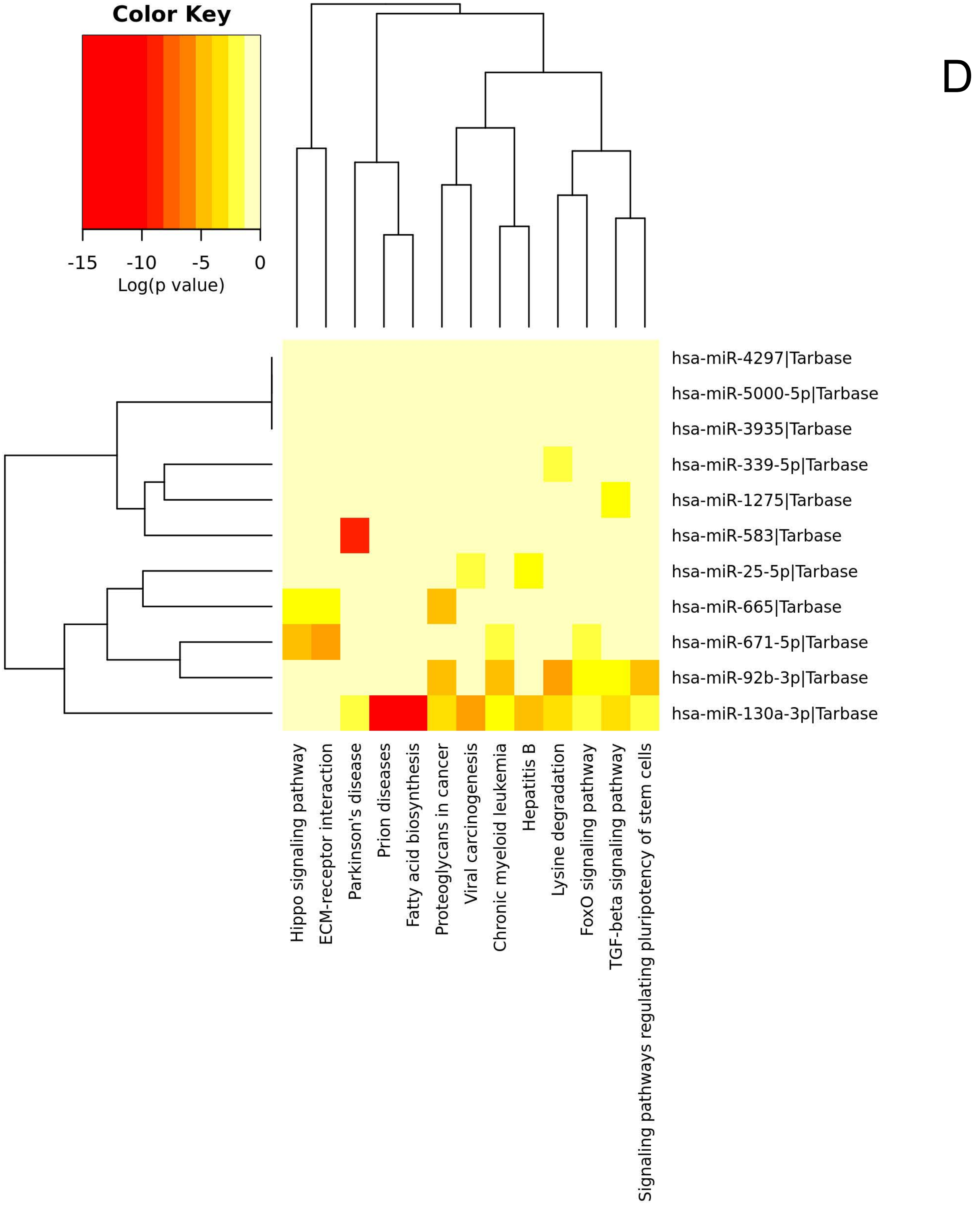

Color Key
-15
-10
-5
0
Log(p value)
D
hsa-miR-4297|Tarbase
hsa-miR-5000-5p|Tarbase
hsa-miR-3935|Tarbase
hsa-miR-339-5p|Tarbase
hsa-miR-1275|Tarbase
hsa-miR-583|Tarbase
hsa-miR-25-5p|Tarbase
hsa-miR-665|Tarbase
hsa-miR-671-5p|Tarbase
hsa-miR-92b-3p|Tarbase
hsa-miR-130a-3p|Tarbase
Hippo signaling pathway
ECM-receptor interaction
Parkinson's disease
Prion diseases
Fatty acid biosynthesis
Proteoglycans in cancer
Viral carcinogenesis
Chronic myeloid leukemia
Hepatitis B
Lysine degradation
FoxO signaling pathway
TGF-beta signaling pathway
Signaling pathways regulating pluripotency of stem cells

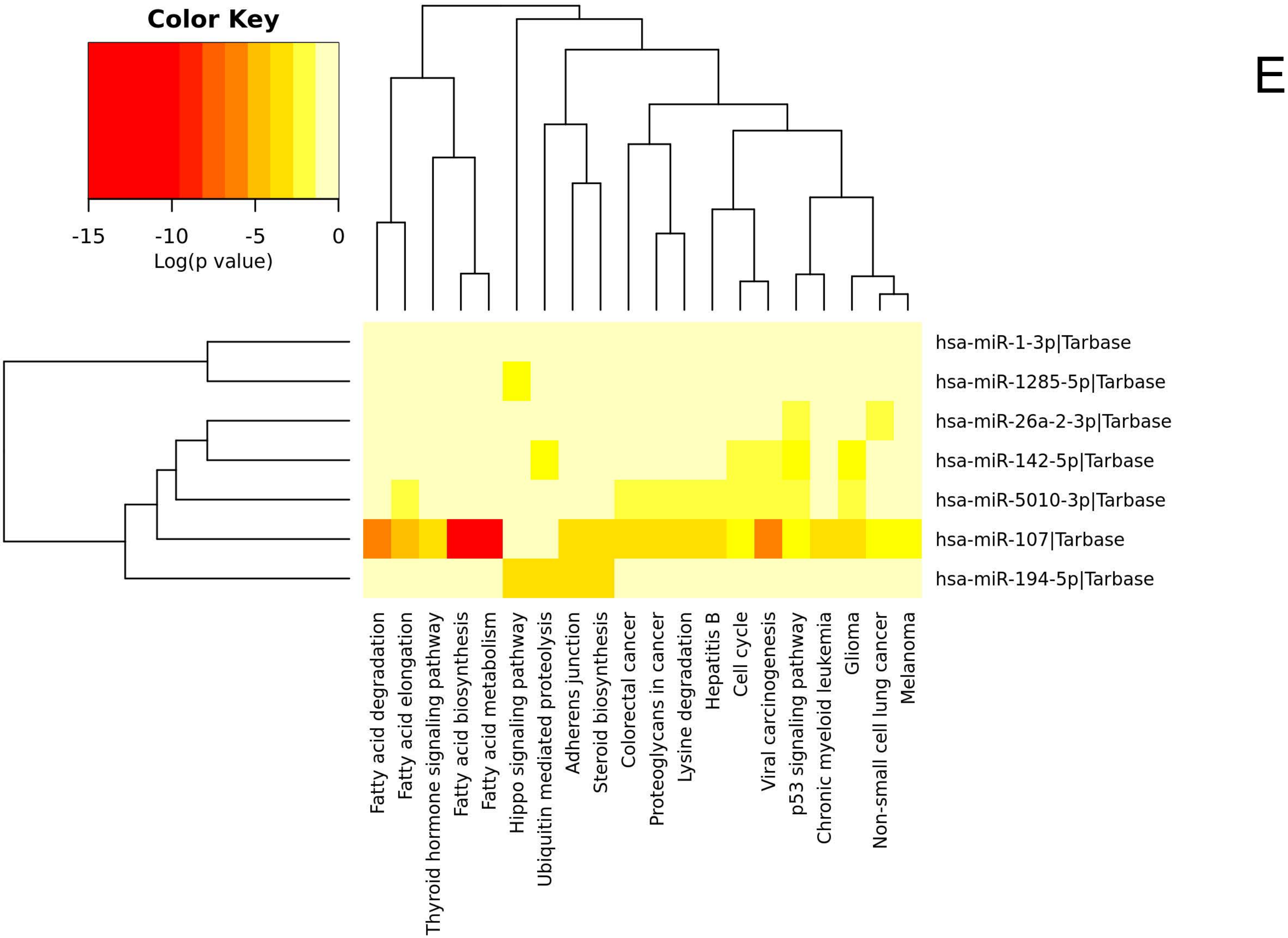

Color Key
-15
-10
-5
0
Log(p value)
E
hsa-miR-1-3p|Tarbase
hsa-miR-1285-5p|Tarbase
hsa-miR-26a-2-3p|Tarbase
hsa-miR-142-5p|Tarbase
hsa-miR-5010-3p|Tarbase
hsa-miR-107|Tarbase
hsa-miR-194-5p|Tarbase
Fatty acid degradation
Fatty acid elongation
Thyroid hormone signaling pathway
Fatty acid biosynthesis
Fatty acid metabolism
Hippo signaling pathway
Ubiquitin mediated proteolysis
Adherens junction
Steroid biosynthesis
Colorectal cancer
Proteoglycans in cancer
Lysine degradation
Hepatitis B
Cell cycle
Viral carcinogenesis
p53 signaling pathway
Chronic myeloid leukemia
Glioma
Non-small cell lung cancer
Melanoma
