# Supplementary material for: Panoramic Visualization of Circulating MicroRNAs Across Neurodegenerative Diseases in Humans
Source: Mol Neurobiol. 2019 Apr 29;56(11):7380–407. doi: 10.1007/s12035-019-1615-1 (PMC6815273; doi:10.1007/s12035-019-1615-1)
Supplement: Supplementary file 4 — (PDF 23413 kb) [file 12035_2019_1615_MOESM4_ESM.pdf]

reactome

Pathway Analysis Report

This report contains the pathway analysis results for the submitted sample ''. Analysis was performed against Reactome version 66 on 30/10/2018 using any resource identifiers for the mapping. The web link to these results is:

https://reactome.org/PathwayBrowser/#/ANALYSIS=MjAxODEwMjkyMjQ2NTdfMTU5OTY%3D

Please keep in mind that analysis results are temporarily stored on our server. The storage period depends on usage of the service but is at least 7 days. As a result, please note that this URL is only valid for a limited time period and it might have expired.

Table of Contents

1. Introduction
2. Properties
3. Genome-wide overview
4. Most significant pathways
5. Pathway details
6. Identifiers found
7. Identifiers not found

1. Introduction

Reactome is a curated database of pathways and reactions in human biology. Reactions can be considered as pathway 'steps'. Reactome defines a 'reaction' as any event in biology that changes the state of a biological molecule. Binding, activation, translocation, degradation and classical biochemical events involving a catalyst are all reactions. Information in the database is authored by expert biologists, entered and maintained by Reactome's team of curators and editorial staff. Reactome content frequently cross-references other resources e.g. NCBI, Ensembl, UniProt, KEGG (Gene and Compound), ChEBI, PubMed and GO. Orthologous reactions inferred from annotation for Homo sapiens are available for 17 non-human species including mouse, rat, chicken, puffer fish, worm, fly, yeast, rice, and Arabidopsis. Pathways are represented by simple diagrams following an SBGN-like format.

Reactome's annotated data describe reactions possible if all annotated proteins and small molecules were present and active simultaneously in a cell. By overlaying an experimental dataset on these annotations, a user can perform a pathway over-representation analysis. By overlaying quantitative expression data or time series, a user can visualize the extent of change in affected pathways and its progression. A binomial test is used to calculate the probability shown for each result, and the p-values are corrected for the multiple testing (Benjamini–Hochberg procedure) that arises from evaluating the submitted list of identifiers against every pathway.

To learn more about our Pathway Analysis, please have a look at our relevant publications:

Fabregat A, Sidiropoulos K, Garapati P, Gillespie M, Hausmann K, Haw R, … D'Eustachio P (2016). The reactome pathway knowledgebase. Nucleic Acids Research, 44(D1), D481–D487. https://doi.org/10.1093/nar/gkv1351.

Fabregat A, Sidiropoulos K, Viteri G, Forner O, Marin-Garcia P, Arnau V, … Hermjakob H (2017). Reactome pathway analysis: a high-performance in-memory approach. BMC Bioinformatics, 18.

2. Properties

- This is an **overrepresentation** analysis: A statistical (hypergeometric distribution) test that determines whether certain Reactome pathways are over-represented (enriched) in the submitted data. It answers the question 'Does my list contain more proteins for pathway X than would be expected by chance?' This test produces a probability score, which is corrected for false discovery rate using the Benjamani-Hochberg method.
- 101 out of 105 identifiers in the sample were found in Reactome, where 788 pathways were hit by at least one of them.
- All non-human identifiers have been converted to their human equivalent.
- This report is filtered to show only results and pathway diagrams for Homo sapiens.
- The unique ID for this analysis (token) is MjAxODEwMjkyMjQ2NTdfMTU5OTY%3D. This ID is valid for at least 7 days in Reactome's server. Use it to access Reactome services with your data.

3. Genome-wide overview

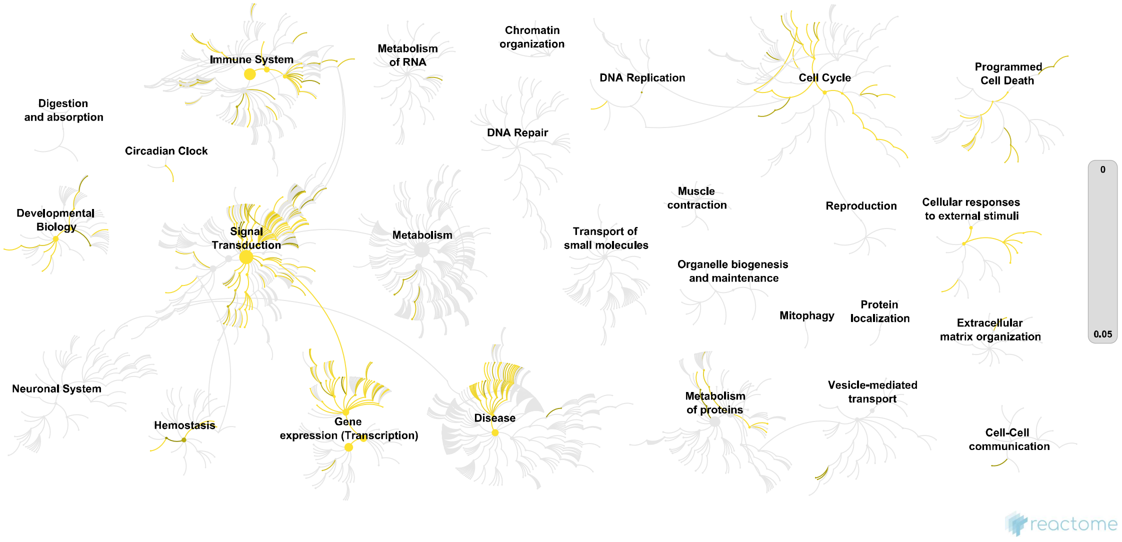

This figure shows a genome-wide overview of the results of your pathway analysis. Reactome pathways are arranged in a hierarchy. The center of each of the circular "bursts" is the root of one top-level pathway, for example "DNA Repair". Each step away from the center represents the next level lower in the pathway hierarchy. The color code denotes over-representation of that pathway in your input dataset. Light grey signifies pathways which are not significantly over-represented.

4. Top 25 pathways

Pathway name	Entities				Reactions	
	found	ratio	p-value	FDR*	found	ratio
Generic Transcription Pathway	79 / 1,454	0.104	1.11e-16	1.61e-14	377 / 726	0.062
RNA Polymerase II Transcription	79 / 1,593	0.114	1.11e-16	1.61e-14	388 / 787	0.067
Interleukin-4 and Interleukin-13 signaling	49 / 211	0.015	1.11e-16	1.61e-14	22 / 46	0.004
Gene expression (Transcription)	80 / 1,751	0.125	1.11e-16	1.61e-14	398 / 898	0.076
Signaling by Interleukins	58 / 640	0.046	1.11e-16	1.61e-14	122 / 491	0.042
Cytokine Signaling in Immune system	58 / 1,055	0.075	1.11e-16	1.61e-14	133 / 639	0.054
Cellular Senescence	24 / 198	0.014	2.22e-16	2.75e-14	47 / 89	0.008
Transcriptional regulation by the AP-2 (TFAP2) family of transcription factors	13 / 52	0.004	3.14e-13	3.07e-11	15 / 44	0.004
Signal Transduction	85 / 3,158	0.226	3.30e-13	3.07e-11	836 / 2,130	0.181
TFAP2 (AP-2) family regulates transcription of growth factors and their receptors	10 / 21	0.002	3.53e-13	3.07e-11	11 / 18	0.002
PIP3 activates AKT signaling	24 / 312	0.022	3.46e-12	2.74e-10	69 / 86	0.007
Diseases of signal transduction	29 / 475	0.034	4.36e-12	3.14e-10	183 / 297	0.025
Transcriptional regulation by RUNX3	16 / 118	0.008	5.24e-12	3.51e-10	24 / 47	0.004
Intrinsic Pathway for Apoptosis	12 / 52	0.004	6.52e-12	4.05e-10	23 / 47	0.004
Transcriptional Regulation by TP53	29 / 486	0.035	7.53e-12	4.37e-10	182 / 259	0.022
PI3K/AKT Signaling in Cancer	16 / 130	0.009	2.16e-11	1.17e-09	21 / 21	0.002
Intracellular signaling by second messengers	24 / 351	0.025	3.83e-11	1.95e-09	69 / 105	0.009
Signaling by Non-Receptor Tyrosine Kinases	12 / 67	0.005	1.16e-10	5.32e-09	22 / 52	0.004
Signaling by PTK6	12 / 67	0.005	1.16e-10	5.32e-09	22 / 52	0.004
Cellular responses to stress	28 / 512	0.037	1.31e-10	5.63e-09	65 / 184	0.016
Mitotic G1-G1/S phases	17 / 173	0.012	1.56e-10	6.38e-09	64 / 98	0.008
Negative regulation of the PI3K/AKT network	15 / 130	0.009	2.25e-10	8.77e-09	8 / 10	8.48e-04
TP53 Regulates Transcription of Genes Involved in G1 Cell Cycle Arrest	8 / 20	0.001	3.29e-10	1.25e-08	14 / 17	0.001
Signaling by Receptor Tyrosine Kinases	27 / 518	0.037	8.17e-10	2.94e-08	307 / 631	0.054

Pathway name	Entities				Reactions	
	found	ratio	p-value	FDR*	found	ratio
Cellular responses to external stimuli	29 / 599	0.043	9.59e-10	3.26e-08	73 / 254	0.022

* False Discovery Rate

5. Pathway details

1. Generic Transcription Pathway (R-HSA-212436)

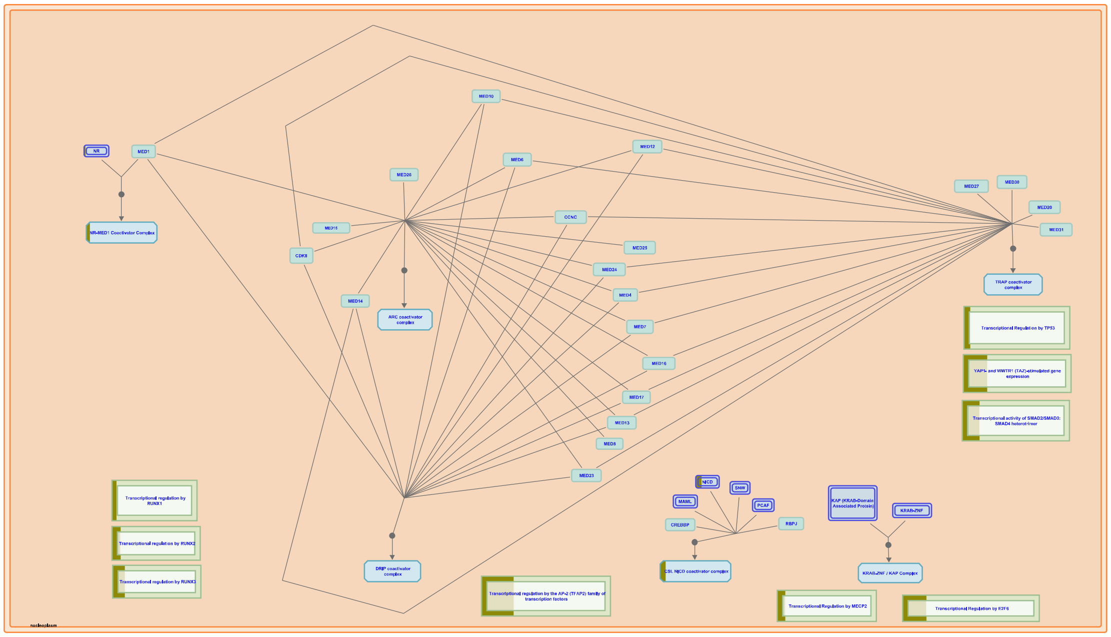

OVERVIEW OF TRANSCRIPTION REGULATION:

Detailed studies of gene transcription regulation in a wide variety of eukaryotic systems has revealed the general principles and mechanisms by which cell- or tissue-specific regulation of differential gene transcription is mediated (reviewed in Naar, 2001. Kadonaga, 2004, Maston, 2006, Barolo, 2002; Roeder, 2005, Rosenfeld, 2006). Of the three major classes of DNA polymerase involved in eukaryotic gene transcription, Polymerase II generally regulates protein-encoding genes. Figure 1 shows a diagram of the various components involved in cell-specific regulation of Pol-II gene transcription.

Core Promoter: Pol II-regulated genes typically have a Core Promoter where Pol II and a variety of general factors bind to specific DNA motifs:

i: the TATA box (TATA DNA sequence), which is bound by the "TATA-binding protein" (TBP).

ii: the Initiator motif (INR), where Pol II and certain other core factors bind, is present in many Pol II-regulated genes.

iii: the Downstream Promoter Element (DPE), which is present in a subset of Pol II genes, and where additional core factors bind.

The core promoter binding factors are generally ubiquitously expressed, although there are exceptions to this.

Proximal Promoter: immediately upstream (5') of the core promoter, Pol II target genes often have a Proximal Promoter region that spans up to 500 base pairs (b.p.), or even to 1000 b.p.. This region contains a number of functional DNA binding sites for a specific set of transcription activator (TA) and transcription repressor (TR) proteins. These TA and TR factors are generally cell- or tissue-specific in expression, rather than ubiquitous, so that the presence of their cognate binding sites in the proximal promoter region programs cell- or tissue-specific expression of the target gene, perhaps in conjunction with TA and TR complexes bound in distal enhancer regions.

Distal Enhancer(s): many or most Pol II regulated genes in higher eukaryotes have one or more distal Enhancer regions which are essential for proper regulation of the gene, often in a cell or tissue-specific pattern. Like the proximal promoter region, each of the distal enhancer regions typically contain a cluster of binding sites for specific TA and/or TR DNA-binding factors, rather than just a single site.

Enhancers generally have three defining characteristics:

i: They can be located very long distances from the promoter of the target gene they regulate, sometimes as far as 100 Kb, or more.

ii: They can be either upstream (5') or downstream (3') of the target gene, including within introns of that gene.

iii: They can function in either orientation in the DNA.

Combinatorial mechanisms of transcription regulation: The specific combination of TA and TR binding sites within the proximal promoter and/or distal enhancer(s) provides a "combinatorial transcription code" that mediates cell- or tissue-specific expression of the associated target gene. Each promoter or enhancer region mediates expression in a specific subset of the overall expression pattern. In at least some cases, each enhancer region functions completely independently of the others, so that the overall expression pattern is a linear combination of the expression patterns of each of the enhancer modules.

Co-Activator and Co-Repressor Complexes: DNA-bound TA and TR proteins typically recruit the assembly of specific Co-Activator (Co-A) and Co-Repressor (Co-R) Complexes, respectively, which are essential for regulating target gene transcription. Both Co-A's and Co-R's are multi-protein complexes that contain several specific protein components.

Co-Activator complexes generally contain at lease one component protein that has Histone Acetyl Transferase (HAT) enzymatic activity. This functions to acetylate Histones and/or other chromatin-associated factors, which typically increases that transcription activation of the target gene. By contrast, Co-Repressor complexes generally contain at lease one component protein that has Histone De-Acetylase (HDAC) enzymatic activity. This functions to de-acetylate Histones and/or other chromatin-associated factors. This typically increases the transcription repression of the target gene.

Adaptor (Mediator) complexes: In addition to the co-activator complexes that assemble on particular cell-specific TA factors, - there are at least two additional transcriptional co-activator complexes common to most cells. One of these is the Mediator complex, which functions as an "adaptor" complex that bridges between the tissue-specific co-activator complexes assembled in the proximal promoter (or distal enhancers). The human Mediator complex has been shown to contain at least 19 protein distinct components. Different combinations of these co-activator proteins are also found to be components of specific transcription Co-Activator complexes, such as the DRIP, TRAP and ARC complexes described below.

TBP/TAF complex: Another large Co-A complex is the "TBP-associated factors" (TAFs) that assemble on TBP (TATA-Binding Protein), which is bound to the TATA box present in many promoters. There are at least 23 human TAF proteins that have been identified. Many of these are ubiquitously expressed, but TAFs can also be expressed in a cell or tissue-specific pattern.

Specific Coactivator Complexes for DNA-binding Transcription Factors.

A number of specific co-activator complexes for DNA-binding transcription factors have been identified, including DRIP, TRAP, and ARC (reviewed in Bourbon, 2004, Blazek, 2005, Conaway, 2005, and Malik, 2005). The DRIP co-activator complex was originally identified and named as a specific complex associated with the Vitamin D Receptor member of the nuclear receptor family of transcription factors (Rachez, 1998). Similarly, the TRAP co-activator complex was originally identified as a complex that associates with the thyroid receptor (Yuan, 1998). It was later determined that all of the components of the DRIP complex are also present in the TRAP complex, and the ARC complex (discussed further below). For example, the DRIP205 and TRAP220 proteins were show to be identical, as were specific pairs of the other components of these complexes (Rachez, 1999).

In addition, these various transcription co-activator proteins identified in mammalian cells were found to be the orthologues or homologues of the Mediator ("adaptor") complex proteins (reviewed in Bourbon, 2004). The Mediator proteins were originally identified in yeast by Kornberg and colleagues, as complexes associated with DNA polymerase (Kelleher, 1990). In higher organisms, Adapter complexes bridge between the basal transcription factors (including Pol II) and tissue-specific transcription factors (TFs) bound to sites within upstream Proximal Promoter regions or distal Enhancer regions (Figure 1). However, many of the Mediator homologues can also be found in complexes associated with specific transcription factors in higher organisms. A unified nomenclature system for these adapter / co-activator proteins now labels them Mediator 1 through Mediator 31 (Bourbon, 2004). For example, the DRIP205 / TRAP220 proteins are now identified as Mediator 1 (Rachez, 1999), based on homology with yeast Mediator 1.

Example Pathway: Specific Regulation of Target Genes During Notch Signaling:

One well-studied example of cell-specific regulation of gene transcription is selective regulation of target genes during Notch signaling. Notch signaling was first identified in Drosophila, where it has been studied in detail at the genetic, molecular, biochemical and cellular levels (reviewed in Justice, 2002; Bray, 2006; Schweisguth, 2004; Louvri, 2006). In Drosophila, Notch signaling to the nucleus is thought always to be mediated by one specific DNA binding transcription factor, Suppressor of Hairless. In mammals, the homologous genes are called CBF1 (or RBPJkappa), while in worms they are called Lag-1, so that the acronym "CSL" has been given to this conserved transcription factor family. There are at least two human CSL homologues, which are now named RBPJ and RBPJL.

In Drosophila, Su(H) is known to be bifunctional, in that it represses target gene transcription in the absence of Notch signaling, but activates target genes during Notch signaling. At least some of the mammalian CSL homologues are believed also to be bifunctional, and to mediate target gene repression in the absence of Notch signaling, and activation in the presence of Notch signaling.

Notch Co-Activator and Co-Repressor complexes: This repression is mediated by at least one specific co-repressor complexes (Co-R) bound to CSL in the absence of Notch signaling. In Drosophila, this co-repressor complex consists of at least three distinct co-repressor proteins: Hairless, Groucho, and dCtBP (Drosophila C-terminal Binding Protein). Hairless has been show to bind directly to Su(H), and Groucho and dCtBP have been shown to bind directly to Hairless (Barolo, 2002). All three of the co-repressor proteins have been shown to be necessary for proper gene regulation during Notch signaling in vivo (Nagel, 2005).

In mammals, the same general pathway and mechanisms are observed, where CSL proteins are bifunctional DNA binding transcription factors (TFs), that bind to Co-Repressor complexes to mediate repression in the absence of Notch signaling, and bind to Co-Activator complexes to mediate activation in the presence of Notch signaling. However, in mammals, there may be multiple co-repressor complexes, rather than the single Hairless co-repressor complex that has been observed in Drosophila.

During Notch signaling in all systems, the Notch transmembrane receptor is cleaved and the Notch intracellular domain (NICD) translocates to the nucleus, where it there functions as a specific transcription co-activator for CSL proteins. In the nucleus, NICD replaces the Co-R complex bound to CSL, thus resulting in de-repression of Notch target genes in the nucleus (Figure 2). Once bound to CSL, NICD and CSL proteins recruit an additional co-activator protein, Mastermind, to form a CSL-NICD-Mam ternary co-activator (Co-A) complex. This Co-R complex was initially thought to be sufficient to mediate activation of at least some Notch target genes. However, there now is evidence that still other co-activators and additional DNA-binding transcription factors are required in at least some contexts (reviewed in Barolo, 2002).

Thus, CSL is a good example of a bifunctional DNA-binding transcription factor that mediates repression of specific targets genes in one context, but activation of the same targets in another context. This bifunctionality is mediated by the association of specific Co-Repressor complexes vs. specific Co-Activator complexes in different contexts, namely in the absence or presence of Notch signaling.

References

Näär AM, Lemon BD & Tjian R (2001). Transcriptional coactivator complexes. Annu Rev Biochem, 70, 475-501.

Kadonaga JT (2004). Regulation of RNA polymerase II transcription by sequence-specific DNA binding factors. Cell, 116, 247-57.

Maston GA, Evans SK & Green MR (2006). Transcriptional regulatory elements in the human genome. Annu Rev Genomics Hum Genet, 7, 29-59.

Barolo S & Posakony JW (2002). Three habits of highly effective signaling pathways: principles of transcriptional control by developmental cell signaling. Genes Dev, 16, 1167-81.

Roeder RG (2005). Transcriptional regulation and the role of diverse coactivators in animal cells. FEBS Lett, 579, 909-15.

Edit history

Date	Action	Author
2008-02-09	Created	Caudy M
2008-02-26	Reviewed	Freedman LP
2018-08-24	Modified	Schmidt EE

Elements found in this pathway

Input	UniProt Id	Input	UniProt Id	Input	UniProt Id
AKT1	P31749	AKT2	P31751	APAF1	O14727
AURKB	Q96GD4	BCL2L11	O43521	BCL6	P41182
BMI1	P35226	CCND1	P24385	CCND2	P30279
CCNE1	P24864	CCNE2	O96020	CDK4	P11802
CDK6	Q00534	CDKN1A	P38936	CHEK1	O14757
CTGF	P29279	E2F1	Q01094	EGFR	P00533
ERBB2	P04626	ESR1	P03372	EZH2	Q15910
FAS	P25445	FOS	P01100	GSK3B	P49841
JAG1	P78504	KIT	P10721	KRAS	P01116
MAPK1	P28482	MET	P08581	MYC	P01106
NOTCH1	P46531	PPARA	Q07869	PTEN	P60484
RARB	P10826	RUNX2	Q13950-2, Q13950-1	SERPINE1	P05121
SMAD1	Q15797	SMAD2	Q15796	SMAD4	Q13485
SMAD7	O15105	SOCS3	O14543	SOX2	P48431
SP1	P08047	SUZ12	Q15022	TNFRSF10B	O14763, Q9UBN6
TP53	P04637	VEGFA	P15692	YWHAZ	P63104

Input	Ensembl Id	Input	Ensembl Id	Input	Ensembl Id
APAF1	ENSG00000120868	BCL2L11	ENSG00000153094	BCL6	ENSG00000113916
CCND1	ENSG00000110092	CDKN1A	ENST00000244741, ENSG00000124762	CHEK1	ENST00000438015, ENSG00000149554
CTGF	ENSG00000118523	E2F1	ENSG00000101412	EGFR	ENSG00000146648
ERBB2	ENSG00000141736	ESR1	ENSG00000091831	FAS	ENSG00000026103
JAG1	ENSG00000101384	KIT	ENSG00000157404	MET	ENSG00000105976
MYC	ENSG00000136997	NOTCH1	ENST00000277541, ENSG00000148400	PTEN	ENST00000371953, ENSG00000171862
RUNX2	ENSG00000124813	SERPINE1	ENSG00000106366	SMAD7	ENSG00000101665
SOCS3	ENSG00000184557	TNFRSF10B	ENSG00000120889	TP53	ENSG00000141510
VEGFA	ENSG00000112715				

2. RNA Polymerase II Transcription (R-HSA-73857)

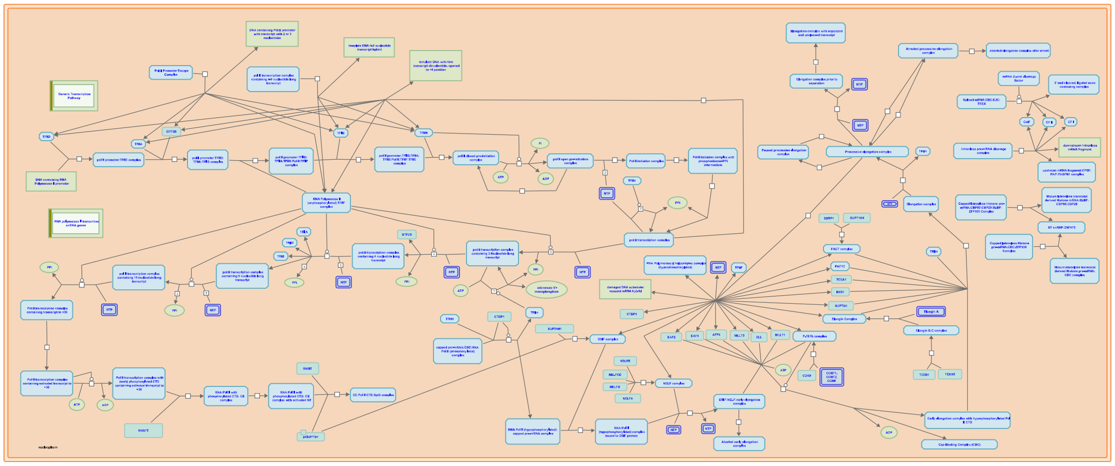

Cellular compartments: nucleoplasm.

RNA polymerase II (Pol II) is the central enzyme that catalyses DNA- directed mRNA synthesis during the transcription of protein-coding genes. Pol II consists of a 10-subunit catalytic core, which alone is capable of elongating the RNA transcript, and a complex of two subunits, Rpb4/7, that is required for transcription initiation.

The transcription cycle is divided in three major phases: initiation, elongation, and termination. Transcription initiation include promoter DNA binding, DNA melting, and initial synthesis of short RNA transcripts. The transition from initiation to elongation, is referred to as promoter escape and leads to a stable elongation complex that is characterized by an open DNA region or transcription bubble. The bubble contains the DNA-RNA hybrid, a heteroduplex of eight to nine base pairs. The growing 3-end of the RNA is engaged with the polymerase complex active site. Ultimately transcription terminates and Pol II dissocitates from the template.

References

Cramer P (2004). Structure and function of RNA polymerase II. Adv. Protein Chem., 67, 1-42.

Edit history

Date	Action	Author
2005-01-12	Created	Reinberg D, Timmers HTM, Conaway JW, Proudfoot NJ, Conaway RC
2018-08-24	Modified	Schmidt EE

Elements found in this pathway

Input	UniProt Id	Input	UniProt Id	Input	UniProt Id
AKT1	P31749	AKT2	P31751	APAF1	O14727
AURKB	Q96GD4	BCL2L11	O43521	BCL6	P41182
BMI1	P35226	CCND1	P24385	CCND2	P30279
CCNE1	P24864	CCNE2	O96020	CDK4	P11802
CDK6	Q00534	CDKN1A	P38936	CHEK1	O14757
CTGF	P29279	E2F1	Q01094	EGFR	P00533
ERBB2	P04626	ESR1	P03372	EZH2	Q15910
FAS	P25445	FOS	P01100	GSK3B	P49841

Input	UniProt Id	Input	UniProt Id	Input	UniProt Id
JAG1	P78504	KIT	P10721	KRAS	P01116
MAPK1	P28482	MET	P08581	MYC	P01106
NOTCH1	P46531	PPARA	Q07869	PTEN	P60484
RARB	P10826	RUNX2	Q13950-2, Q13950-1	SERPINE1	P05121
SMAD1	Q15797	SMAD2	Q15796	SMAD4	Q13485
SMAD7	O15105	SOCS3	O14543	SOX2	P48431
SP1	P08047	SUZ12	Q15022	TNFRSF10B	O14763, Q9UBN6
TP53	P04637	VEGFA	P15692	YWHAZ	P63104

Input	Ensembl Id	Input	Ensembl Id	Input	Ensembl Id
APAF1	ENSG00000120868	BCL2L11	ENSG00000153094	BCL6	ENSG00000113916
CCND1	ENSG00000110092	CDKN1A	ENST00000244741, ENSG00000124762	CHEK1	ENST00000438015, ENSG00000149554
CTGF	ENSG00000118523	E2F1	ENSG00000101412	EGFR	ENSG00000146648
ERBB2	ENSG00000141736	ESR1	ENSG00000091831	FAS	ENSG00000026103
JAG1	ENSG00000101384	KIT	ENSG00000157404	MET	ENSG00000105976
MYC	ENSG00000136997	NOTCH1	ENST00000277541, ENSG00000148400	PTEN	ENST00000371953, ENSG00000171862
RUNX2	ENSG00000124813	SERPINE1	ENSG00000106366	SMAD7	ENSG00000101665
SOCS3	ENSG00000184557	TNFRSF10B	ENSG00000120889	TP53	ENSG00000141510
VEGFA	ENSG00000112715				

3. Interleukin-4 and Interleukin-13 signaling (R-HSA-6785807)

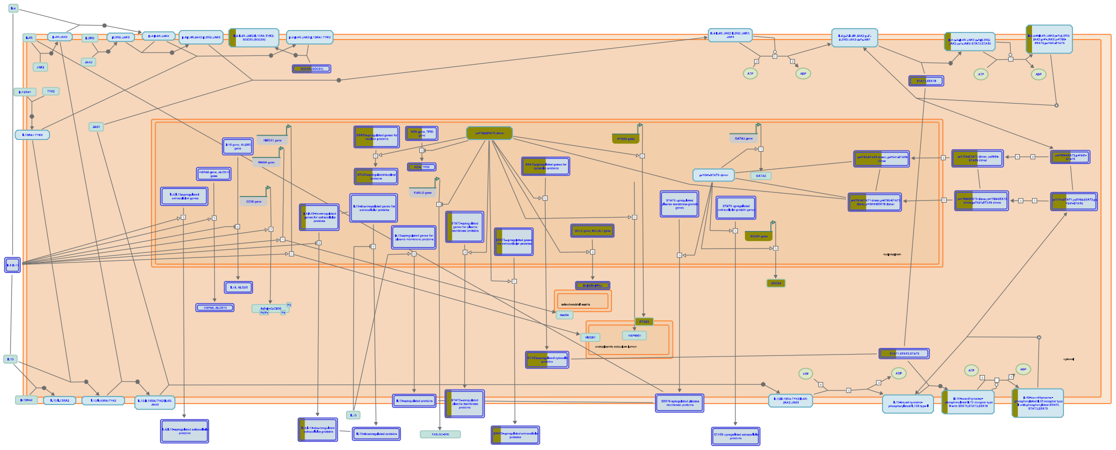

Interleukin-4 (IL4) is a principal regulatory cytokine during the immune response, crucially important in allergy and asthma (Nelms et al. 1999). When resting T cells are antigen-activated and expand in response to Interleukin-2 (IL2), they can differentiate as Type 1 (Th1) or Type 2 (Th2) T helper cells. The outcome is influenced by IL4. Th2 cells secrete IL4, which both stimulates Th2 in an autocrine fashion and acts as a potent B cell growth factor to promote humoral immunity (Nelms et al. 1999).

Interleukin-13 (IL13) is an immunoregulatory cytokine secreted predominantly by activated Th2 cells. It is a key mediator in the pathogenesis of allergic inflammation. IL13 shares many functional properties with IL4, stemming from the fact that they share a common receptor subunit. IL13 receptors are expressed on human B cells, basophils, eosinophils, mast cells, endothelial cells, fibroblasts, monocytes, macrophages, respiratory epithelial cells, and smooth muscle cells, but unlike IL4, not T cells. Thus IL13 does not appear to be important in the initial differentiation of CD4 T cells into Th2 cells, rather it is important in the effector phase of allergic inflammation (Hershey et al. 2003).

IL4 and IL13 induce “alternative activation” of macrophages, inducing an anti-inflammatory phenotype by signaling through IL4R alpha in a STAT6 dependent manner. This signaling plays an important role in the Th2 response, mediating anti-parasitic effects and aiding wound healing (Gordon & Martinez 2010, Loke et al. 2002)

There are two types of IL4 receptor complex (Andrews et al. 2006). Type I IL4R (IL4R1) is predominantly expressed on the surface of hematopoietic cells and consists of IL4R and IL2RG, the common gamma chain. Type II IL4R (IL4R2) is predominantly expressed on the surface of nonhematopoietic cells, it consists of IL4R and IL13RA1 and is also the type II receptor for IL13. (Obiri et al. 1995, Aman et al. 1996, Hilton et al. 1996, Miloux et al. 1997, Zhang et al. 1997). The second receptor for IL13 consists of IL4R and Interleukin-13 receptor alpha 2 (IL13RA2), sometimes called Interleukin-13 binding protein (IL13BP). It has a high affinity receptor for IL13 (Kd = 250 pmol/L) but is not sufficient to render cells responsive to IL13, even in the presence of IL4R (Donaldson et al. 1998). It is reported to exist in soluble form (Zhang et al. 1997) and when overexpressed reduces JAK-STAT signaling (Kawakami et al. 2001). It's function may be to prevent IL13 signalling via the functional IL4R:IL13RA1 receptor. IL13RA2 is overexpressed and enhances cell invasion in some human cancers (Joshi & Puri 2012).

The first step in the formation of IL4R1 (IL4:IL4R:IL2RB) is the binding of IL4 with IL4R (Hoffman et al. 1995, Shen et al. 1996, Hage et al. 1999). This is also the first step in formation of IL4R2 (IL4:IL4R:IL13RA1). After the initial binding of IL4 and IL4R, IL2RB binds (LaPorte et al. 2008), to form IL4R1. Alternatively, IL13RA1 binds, forming IL4R2. In contrast, the type II IL13 complex (IL13R2) forms with IL13 first binding to IL13RA1 followed by recruitment of IL4R (Wang et al. 2009).

Crystal structures of the IL4:IL4R:IL2RG, IL4:IL4R:IL13RA1 and IL13:IL4R:IL13RA1 complexes have been determined (LaPorte et al. 2008). Consistent with these structures, in monocytes IL4R is tyrosine phosphorylated in response to both IL4 and IL13 (Roy et al. 2002, Gordon & Martinez 2010) while IL13RA1 phosphorylation is induced only by IL13 (Roy et al. 2002, LaPorte et al. 2008) and IL2RG phosphorylation is induced only by IL4 (Roy et al. 2002).

Both IL4 receptor complexes signal through Jak/STAT cascades. IL4R is constitutively-associated with JAK2 (Roy et al. 2002) and associates with JAK1 following binding of IL4 (Yin et al. 1994) or IL13 (Roy et al. 2002). IL2RG constitutively associates with JAK3 (Boussiotis et al. 1994, Russell et al. 1994). IL13RA1 constitutively associates with TYK2 (Umeshita-Suyama et al. 2000, Roy et al. 2002, LaPorte et al. 2008, Bhattacharjee et al. 2013).

IL4 binding to IL4R1 leads to phosphorylation of JAK1 (but not JAK2) and STAT6 activation (Takeda et al. 1994, Ratthe et al. 2007, Bhattacharjee et al. 2013).

IL13 binding increases activating tyrosine-99 phosphorylation of IL13RA1 but not that of IL2RG. IL4 binding to IL2RG leads to its tyrosine phosphorylation (Roy et al. 2002). IL13 binding to IL4R2 leads to TYK2 and JAK2 (but not JAK1) phosphorylation (Roy & Cathcart 1998, Roy et al. 2002).

Phosphorylated TYK2 binds and phosphorylates STAT6 and possibly STAT1 (Bhattacharjee et al. 2013).

A second mechanism of signal transduction activated by IL4 and IL13 leads to the insulin receptor substrate (IRS) family (Kelly-Welch et al. 2003). IL4R1 associates with insulin receptor substrate 2 and activates the PI3K/Akt and Ras/MEK/Erk pathways involved in cell proliferation, survival and translational control. IL4R2 does not associate with insulin receptor substrate 2 and consequently the PI3K/Akt and Ras/MEK/Erk pathways are not activated (Busch-Dienstfertig & González-Rodríguez 2013).

References

Nelms K, Keegan AD, Zamorano J, Ryan JJ & Paul WE (1999). The IL-4 receptor: signaling mechanisms and biologic functions. Annu. Rev. Immunol., 17, 701-38.

Hershey GK (2003). IL-13 receptors and signaling pathways: an evolving web. J. Allergy Clin. Immunol., 111, 677-90; quiz 691.

Edit history

Date	Action	Author
2015-07-01	Authored	Jupe S
2015-07-01	Created	Jupe S
2016-09-02	Edited	Jupe S
2016-09-02	Reviewed	Leibovich SJ

Date	Action	Author
2018-08-30	Modified	Croft D

Elements found in this pathway

Input	UniProt Id	Input	UniProt Id	Input	UniProt Id
AKT1	P31749	BCL2	P10415	BCL2L1	Q07817
BCL6	P41182	CCND1	P24385	CDKN1A	P38936
FOS	P01100	FOXO1	Q12778	FSCN1	Q16658
HIF1A	Q16665	ICAM1	P05362	IL6	P05231
MCL1	Q07820	MMP2	P08253	MMP9	P14780
MYC	P01106	PIK3R1	P27986	PTGS2	P35354
SOCS1	O15524	SOCS3	O14543	SOX2	P48431
STAT3	P40763	TP53	P04637	VEGFA	P15692
ZEB1	P37275				

Input	Ensembl Id	Input	Ensembl Id	Input	Ensembl Id
AKT1	ENSG00000142208	BCL2	ENSG00000171791	BCL2L1	ENSG00000171552
BCL6	ENSG00000113916	CCND1	ENSG00000110092	CDKN1A	ENSG00000124762
FOS	ENSG00000170345	FOXO1	ENSG00000150907	FSCN1	ENSG00000075618
HIF1A	ENSG00000100644	ICAM1	ENSG00000090339	IL6	ENSG00000136244
MCL1	ENSG00000143384	MMP2	ENSG00000087245	MMP9	ENSG00000100985
MYC	ENSG00000136997	PIK3R1	ENSG00000145675	PTGS2	ENSG00000073756
SOCS1	ENSG00000185338	SOCS3	ENSG00000184557	SOX2	ENSG00000181449
TP53	ENSG00000141510	VEGFA	ENSG00000112715	ZEB1	ENSG00000148516

4. Gene expression (Transcription) (R-HSA-74160)

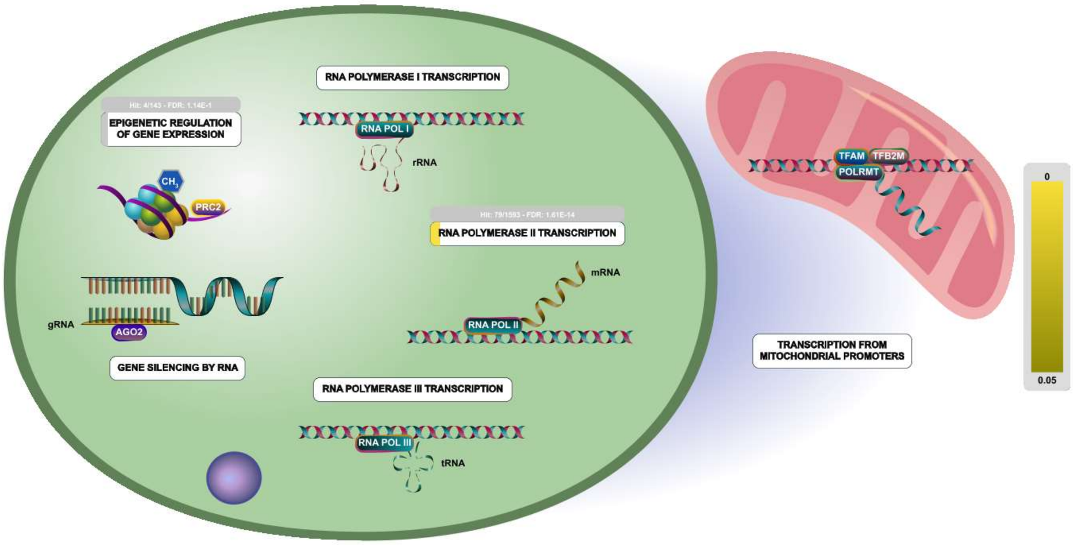

Gene expression encompasses transcription and translation and the regulation of these processes. RNA Polymerase I Transcription produces the large preribosomal RNA transcript (45S pre-rRNA) that is processed to yield 18S rRNA, 28S rRNA, and 5.8S rRNA, accounting for about half the RNA in a cell. RNA Polymerase II transcription produces messenger RNAs (mRNA) as well as a subset of non-coding RNAs including many small nucleolar RNAs (snRNA) and microRNAs (miRNA). RNA Polymerase III Transcription produces transfer RNAs (tRNA), 5S RNA, 7SL RNA, and U6 snRNA. Transcription from mitochondrial promoters is performed by the mitochondrial RNA polymerase, POLRMT, to yield long transcripts from each DNA strand that are processed to yield 12S rRNA, 16S rRNA, tRNAs, and a few RNAs encoding components of the electron transport chain. Regulation of gene expression can be divided into epigenetic regulation, transcriptional regulation, and post-transcription regulation (comprising translational efficiency and RNA stability). Epigenetic regulation of gene expression is the result of heritable chemical modifications to DNA and DNA-binding proteins such as histones. Epigenetic changes result in altered chromatin complexes that influence transcription. Gene Silencing by RNA mostly occurs post-transcriptionally but can also affect transcription. Small RNAs originating from the genome (miRNAs) or from exogenous RNA (siRNAs) are processed and transferred to the RNA-induced silencing complex (RISC), which interacts with complementary RNA to cause cleavage, translational inhibition, or transcriptional inhibition.

Edit history

Date	Action	Author
2003-09-11	Authored	Larsson NG, Comai L, Gustafsson CM, Reinberg D, Timmers HTM et al.
2003-09-11	Created	Proudfoot NJ, Kornblihtt AR
2008-12-03	Authored	Caudy M, Proudfoot NJ, Kornblihtt AR, D'Eustachio P
2016-12-29	Revised	D'Eustachio P
2018-08-21	Edited	Joshi-Tope G
2018-08-21	Reviewed	Paule M, Willis I, Zhao X
2018-08-24	Modified	Schmidt EE

Elements found in this pathway

Input	UniProt Id	Input	UniProt Id	Input	UniProt Id
AKT1	P31749	AKT2	P31751	APAF1	O14727
AURKB	Q96GD4	BCL2L11	O43521	BCL6	P41182
BMI1	P35226	CCND1	P24385	CCND2	P30279
CCNE1	P24864	CCNE2	O96020	CDK4	P11802
CDK6	Q00534	CDKN1A	P38936	CHEK1	O14757
CTGF	P29279	DNMT1	P26358	E2F1	Q01094
EGFR	P00533	ERBB2	P04626	ESR1	P03372
EZH2	Q15910	FAS	P25445	FOS	P01100
GSK3B	P49841	JAG1	P78504	KIT	P10721
KRAS	P01116	MAPK1	P28482	MET	P08581
MYC	P01106	NOTCH1	P46531	PPARA	Q07869
PTEN	P60484	RARB	P10826	RUNX2	Q13950-2, Q13950-1
SERPINE1	P05121	SMAD1	Q15797	SMAD2	Q15796
SMAD4	Q13485	SMAD7	O15105	SOCS3	O14543
SOX2	P48431	SP1	P08047	SUZ12	Q15022
TNFRSF10B	O14763, Q9UBN6	TP53	P04637	VEGFA	P15692
YWHAZ	P63104				

Input	Ensembl Id	Input	Ensembl Id	Input	Ensembl Id
APAF1	ENSG00000120868	BCL2L11	ENSG00000153094	BCL6	ENSG00000113916
CCND1	ENSG00000110092	CDKN1A	ENST00000244741, ENSG00000124762	CHEK1	ENST00000438015, ENSG00000149554
CTGF	ENSG00000118523	E2F1	ENSG00000101412	EGFR	ENSG00000146648
ERBB2	ENSG00000141736	ESR1	ENSG00000091831	FAS	ENSG00000026103
JAG1	ENSG00000101384	KIT	ENSG00000157404	MET	ENSG00000105976
MYC	ENSG00000136997	NOTCH1	ENST00000277541, ENSG00000148400	PTEN	ENST00000371953, ENSG00000171862
RUNX2	ENSG00000124813	SERPINE1	ENSG00000106366	SMAD7	ENSG00000101665
SOCS3	ENSG00000184557	TNFRSF10B	ENSG00000120889	TP53	ENSG00000141510
VEGFA	ENSG00000112715				

5. Signaling by Interleukins (R-HSA-449147)

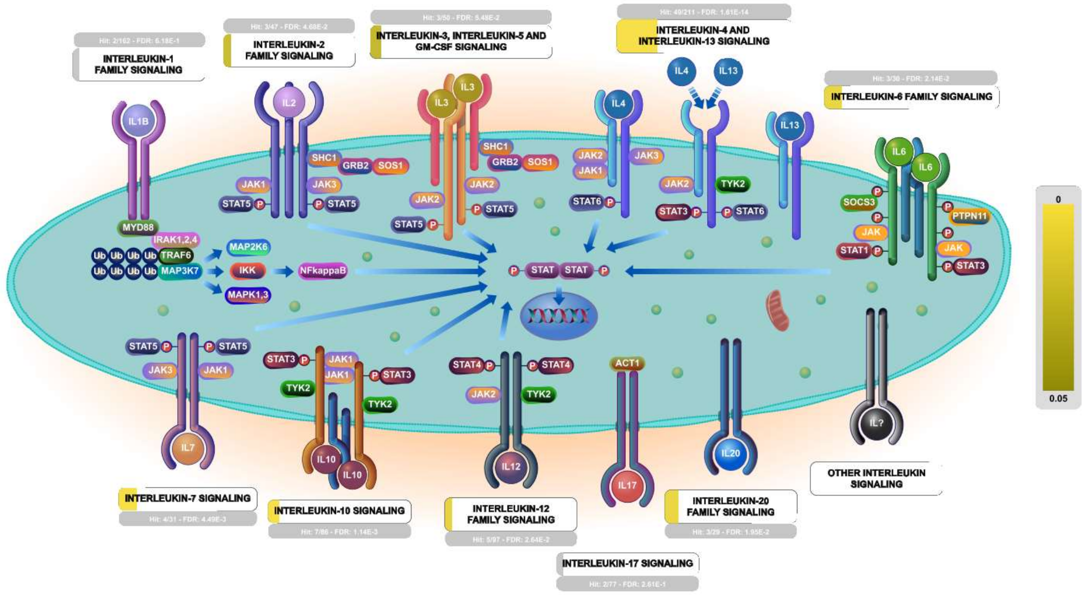

Cellular compartments: plasma membrane.

Interleukins are low molecular weight proteins that bind to cell surface receptors and act in an autocrine and/or paracrine fashion. They were first identified as factors produced by leukocytes but are now known to be produced by many other cells throughout the body. They have pleiotropic effects on cells which bind them, impacting processes such as tissue growth and repair, hematopoietic homeostasis, and multiple levels of the host defense against pathogens where they are an essential part of the immune system.

References

Vosshenrich CA & Di Santo JP (2002). Interleukin signaling. Curr Biol, 12, R760-3.

Dinarello CA (2009). Immunological and inflammatory functions of the interleukin-1 family. Annu Rev Immunol, 27, 519-50.

Akdis M, Aab A, Altunbulakli C, Azkur K, Costa RA, Crameri R, ... Akdis CA (2016). Interleukins (from IL-1 to IL-38), interferons, transforming growth factor , and TNF-: Receptors, functions, and roles in diseases. J. Allergy Clin. Immunol., 138, 984-1010.

Edit history

Date	Action	Author
2009-11-27	Created	Jupe S
2010-05-17	Reviewed	Pinteaux E
2010-05-17	Authored	Ray KP
2010-05-26	Edited	Jupe S
2018-08-24	Modified	Schmidt EE

Elements found in this pathway

Input	UniProt Id	Input	UniProt Id	Input	UniProt Id
AKT1	P31749	BCL2	P10415	BCL2L1	Q07817
BCL6	P41182	CCND1	P24385	CDC42	P60953
CDKN1A	P38936	FOS	P01100	FOXO1	Q12778
FSCN1	Q16658	HIF1A	Q16665	HMGB1	P09429
ICAM1	P05362	IL6	P05231	MAPK1	P28482
MCL1	Q07820	MMP2	P08253	MMP9	P14780
MYC	P01106	PDCD4	Q53EL6	PIK3CA	P42336
PIK3R1	P27986	PTGS2	P35354	SOCS1	O15524
SOCS3	O14543	SOX2	P48431	STAT3	P40763
TP53	P04637	VEGFA	P15692	YWHAZ	P63104
ZEB1	P37275				

Input	Ensembl Id	Input	Ensembl Id	Input	Ensembl Id
AKT1	ENSG00000142208	BCL2	ENSG00000171791	BCL2L1	ENSG00000171552
BCL6	ENSG00000113916	CCND1	ENSG00000110092	CDC42	ENSG00000070831
CDKN1A	ENSG00000124762	FOS	ENSG00000170345	FOXO1	ENSG00000150907
FSCN1	ENSG00000075618	HIF1A	ENSG00000100644	ICAM1	ENSG00000090339
IL6	ENSG00000136244	MCL1	ENSG00000143384	MMP2	ENSG00000087245
MMP9	ENSG00000100985	MYC	ENSG00000136997	PDCD4	ENSG00000150593
PIK3R1	ENSG00000145675	PTGS2	ENSG00000073756	SOCS1	ENSG00000185338
SOCS3	ENST00000330871, ENSG00000184557	SOX2	ENSG00000181449	TP53	ENSG00000141510
VEGFA	ENSG00000112715	ZEB1	ENSG00000148516		

6. Cytokine Signaling in Immune system (R-HSA-1280215)

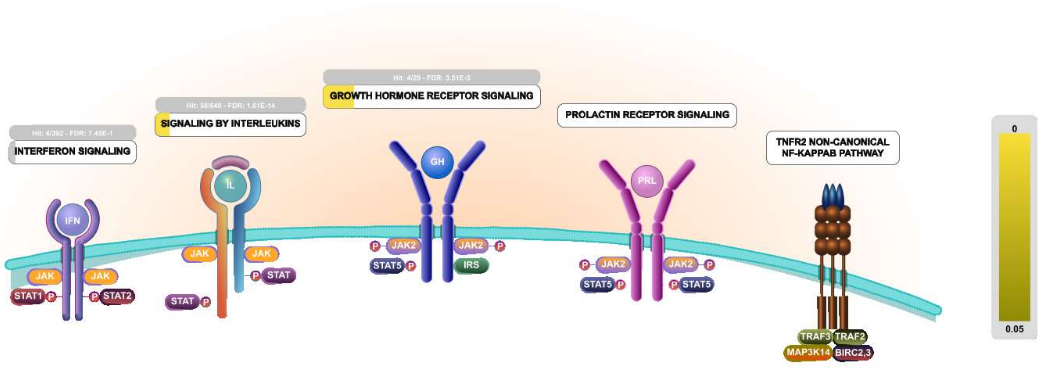

Cytokines are small proteins that regulate and mediate immunity, inflammation, and hematopoiesis. They are secreted in response to immune stimuli, and usually act briefly, locally, at very low concentrations. Cytokines bind to specific membrane receptors, which then signal the cell via second messengers, to regulate cellular activity.

References

Oppenheim J & Feldmann M (2002). *Cytokines and the immune system, Cytokine Reference* .

IMMPORT:Bioinformatics for the future of immunology. Retrieved from https://www.immport.org/immportWeb/queryref/geneListSummary.do

COPE. Retrieved from http://www.copewithcytokines.org/cope.cgi

Santamaria P (2003). Cytokines and chemokines in autoimmune disease: an overview. Adv Exp Med Biol, 520, 1-7.

Edit history

Date	Action	Author
2011-05-12	Created	Garapati P V
2011-05-22	Edited	Ray KP, Jupe S, Garapati P V
2011-05-22	Authored	Ray KP, Jupe S, Garapati P V
2011-05-29	Reviewed	Abdul-Sater AA, Schindler C, Pinteaux E
2018-08-24	Modified	Schmidt EE

Elements found in this pathway

Input	UniProt Id	Input	UniProt Id	Input	UniProt Id
AKT1	P31749	BCL2	P10415	BCL2L1	Q07817
BCL6	P41182	CCND1	P24385	CDC42	P60953
CDKN1A	P38936	FOS	P01100	FOXO1	Q12778
FSCN1	Q16658	HIF1A	Q16665	HMGB1	P09429
ICAM1	P05362	IL6	P05231	MAPK1	P28482
MCL1	Q07820	MMP2	P08253	MMP9	P14780
MYC	P01106	PDCD4	Q53EL6	PIK3CA	P42336

Input	UniProt Id	Input	UniProt Id	Input	UniProt Id
PIK3R1	P27986	PTGS2	P35354	SOCS1	O15524
SOCS3	O14543	SOX2	P48431	STAT3	P40763
TP53	P04637	VEGFA	P15692	YWHAZ	P63104
ZEB1	P37275				

Input	Ensembl Id	Input	Ensembl Id	Input	Ensembl Id
AKT1	ENSG00000142208	BCL2	ENSG00000171791	BCL2L1	ENSG00000171552
BCL6	ENSG00000113916	CCND1	ENSG00000110092	CDC42	ENSG00000070831
CDKN1A	ENSG00000124762	FOS	ENSG00000170345	FOXO1	ENSG00000150907
FSCN1	ENSG00000075618	HIF1A	ENSG00000100644	ICAM1	ENSG00000090339
IL6	ENSG00000136244	MCL1	ENSG00000143384	MMP2	ENSG00000087245
MMP9	ENSG00000100985	MYC	ENSG00000136997	PDCD4	ENSG00000150593
PIK3R1	ENSG00000145675	PTGS2	ENSG00000073756	SOCS1	ENSG00000185338
SOCS3	ENST00000330871, ENSG00000184557	SOX2	ENSG00000181449	TP53	ENSG00000141510
VEGFA	ENSG00000112715	ZEB1	ENSG00000148516		

7. Cellular Senescence (R-HSA-2559583)

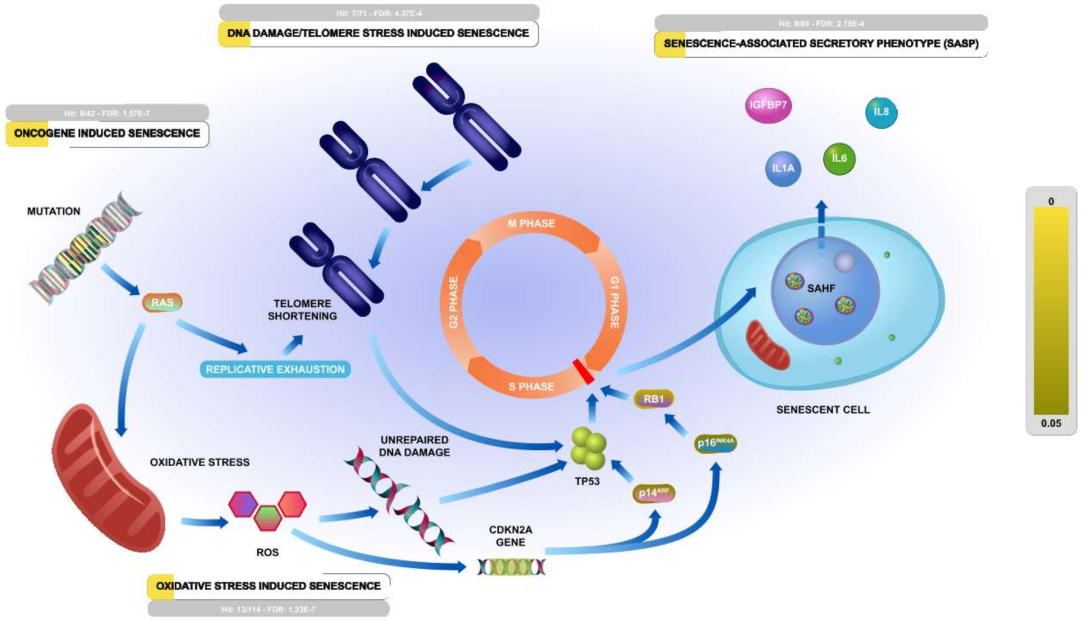

Cellular senescence involves irreversible growth arrest accompanied by phenotypic changes such as enlarged morphology, reorganization of chromatin through formation of senescence-associated heterochromatic foci (SAHF), and changes in gene expression that result in secretion of a number of proteins that alter local tissue environment, known as senescence-associated secretory phenotype (SASP).

Senescence is considered to be a cancer protective mechanism and is also involved in aging. Senescent cells accumulate in aged tissues (reviewed by Campisi 1997 and Lopez-Otin 2013), which may be due to an increased senescence rate and/or decrease in the rate of clearance of senescent cells. In a mouse model of accelerated aging, clearance of senescent cells delays the onset of age-related phenotypes (Baker et al. 2011).

Cellular senescence can be triggered by the aberrant activation of oncogenes or loss-of-function of tumor suppressor genes, and this type of senescence is known as the oncogene-induced senescence, with RAS signaling-induced senescence being the best studied. Oxidative stress, which may or may not be caused by oncogenic RAS signaling, can also trigger senescence. Finally, the cellular senescence program can be initiated by DNA damage, which may be caused by reactive oxygen species (ROS) during oxidative stress, and by telomere shortening caused by replicative exhaustion which may be due to oncogenic signaling. The senescent phenotype was first reported by Hayflick and Moorhead in 1961, when they proposed replicative senescence as a mechanism responsible for the cessation of mitotic activity and morphological changes that occur in human somatic diploid cell strains as a consequence of serial passaging, preventing the continuous culture of untransformed cells-the Hayflick limit (Hayflick and Moorhead 1961).

Secreted proteins that constitute the senescence-associated secretory phenotype (SASP), also known as the senescence messaging secretome (SMS), include inflammatory and immune-modulatory cytokines, growth factors, shed cell surface molecules and survival factors. The SASP profile is not significantly affected by the type of senescence trigger or the cell type (Coppe et al. 2008), but the persistent DNA damage may be a deciding SASP initiator (Rodier et al. 2009). SASP components function in an autocrine manner, reinforcing the senescent phenotype (Kuilman et al. 2008, Acosta et al. 2008), and in the paracrine manner, where they may promote epithelial-to-mesenchymal transition (EMT) and malignancy in the nearby premalignant or malignant cells (Coppe et al. 2008).

Senescent cells may remain viable for years, such as senescent melanocytes of moles and nevi, or they can be removed by phagocytic cells. The standard marker for immunohistochemical detection of senescent cells is senescence-associated beta-galactosidase (SA-beta-Gal), a lysosomal enzyme that is not required for senescence.

For reviews of this topic, please refer to Collado et al. 2007, Adams 2009, Kuilman et al. 2010. For a review of differential gene expression between senescent and immortalized cells, please refer to Fridman and Tainsky 2008.

References

Collado M, Blasco MA & Serrano M (2007). Cellular senescence in cancer and aging. Cell, 130, 223-33.

Adams PD (2009). Healing and hurting: molecular mechanisms, functions, and pathologies of cellular senescence. Mol. Cell, 36, 2-14.

Kuilman T, Michaloglou C, Mooi WJ & Peeper DS (2010). The essence of senescence. Genes Dev., 24 , 2463-79.

Campisi J & d'Adda di Fagagna F (2007). Cellular senescence: when bad things happen to good cells. Nat. Rev. Mol. Cell Biol., 8, 729-40.

Campisi J (1997). The biology of replicative senescence. Eur. J. Cancer, 33, 703-9.

Edit history

Date	Action	Author
2012-11-02	Created	Orlic-Milacic M
2013-07-15	Edited	Matthews L, D'Eustachio P
2013-07-15	Authored	Orlic-Milacic M
2013-09-03	Reviewed	Samarajiwa S
2013-09-30	Revised	Orlic-Milacic M
2018-08-24	Modified	Schmidt EE

Elements found in this pathway

Input	UniProt Id	Input	UniProt Id	Input	UniProt Id
BMI1	P35226	CCNE1	P24864	CCNE2	O96020
CDK4	P11802	CDK6	Q00534	CDKN1A	P38936
E2F1	O00716, Q01094	E2F2	Q14209	ETS1	P14921
EZH2	Q15910	FOS	P01100	HMGA1	P17096
HMGA2	P52926	IL6	P05231	MAPK1	P28482

Input	UniProt Id
SP1	P08047
TP53	P04637

Input	UniProt Id
STAT3	P40763

Input	UniProt Id
SUZ12	Q15022

Input	Ensembl Id
CDKN1A	ENSG00000124762
SUZ12	ENSG00000178691

Input	Ensembl Id
EZH2	ENSG00000106462

Input	Ensembl Id
IL6	ENSG00000136244

8. Transcriptional regulation by the AP-2 (TFAP2) family of transcription factors (R-HSA-8864260)

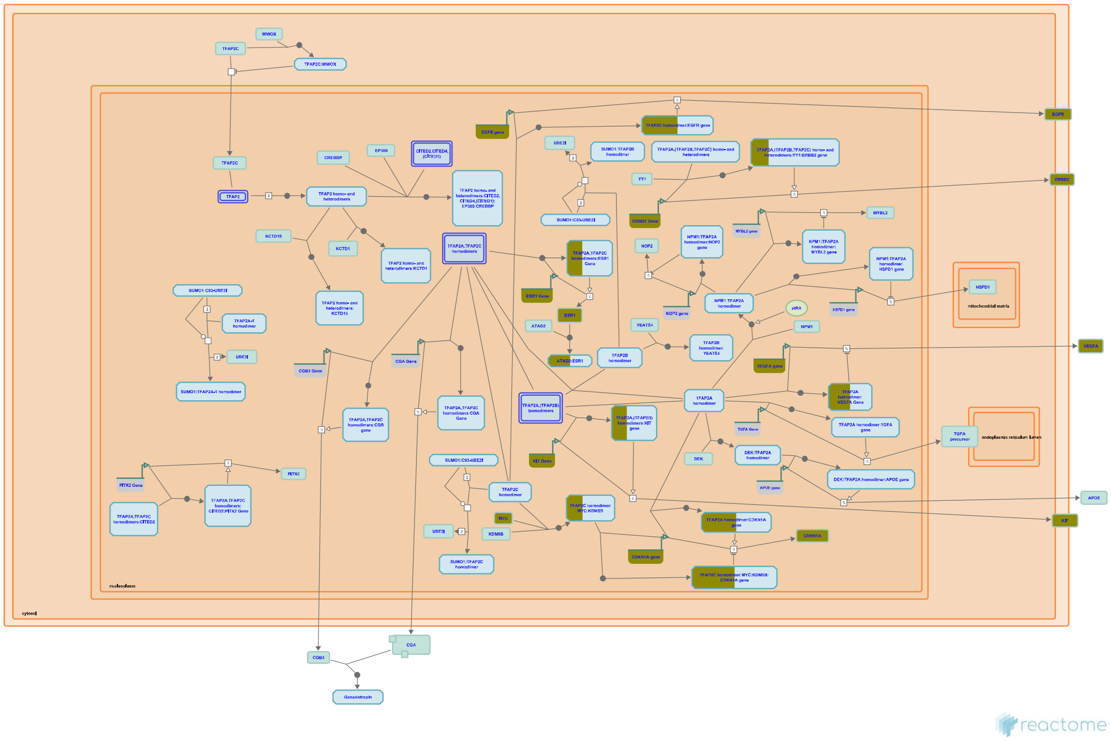

The AP-2 (TFAP2) family of transcription factors includes five proteins in mammals: TFAP2A (AP-2 alpha), TFAP2B (AP-2 beta), TFAP2C (AP-2 gamma), TFAP2D (AP-2 delta) and TFAP2E (AP-2 epsilon). The AP-2 family transcription factors are evolutionarily conserved in metazoans and are characterized by a helix-span-helix motif at the C-terminus, a central basic region, and the transactivation domain at the N-terminus. The helix-span-helix motif and the basic region enable dimerization and DNA binding (Eckert et al. 2005).

AP-2 dimers bind palindromic GC-rich DNA response elements that match the consensus sequence 5'-GCCNNNGGC-3' (Williams and Tjian 1991a, Williams and Tjian 1991b). Transcriptional co-factors from the CITED family interact with the helix-span-helix (HSH) domain of TFAP2 (AP-2) family of transcription factors and recruit transcription co-activators EP300 (p300) and CREBBP (CBP) to TFAP2-bound DNA elements. CITED2 shows the highest affinity for TFAP2 proteins, followed by CITED4, while CITED1 interacts with TFAP2s with a very low affinity. Mouse embryos defective for CITED2 exhibit neural crest defects, cardiac malformations and adrenal agenesis, which can at least in part be attributed to a defective Tfap2 transactivation (Bamforth et al. 2001, Braganca et al. 2002, Braganca et al. 2003). Transcriptional activity of AP-2 dimers in inhibited by binding of KCTD1 or KCTD15 to the AP-2 transactivation domain (Ding et al. 2009, Zarelli and Dawid 2013). Transcriptional activity of TFAP2A, TFAP2B and TFAP2C is negatively regulated by SUMOylation mediated by UBE2I (UBC9) (Eloranta and Hurst 2002, Berlato et al. 2011, Impens et al. 2014, Bogachek et al. 2014).

During embryonic development, AP-2 transcription factors stimulate proliferation and suppress terminal differentiation in a cell-type specific manner (Eckert et al. 2005).

TFAP2A and TFAP2C directly stimulate transcription of the estrogen receptor ESR1 gene (McPherson and Weigel 1999). TFAP2A expression correlates with ESR1 expression in breast cancer, and TFAP2C is frequently overexpressed in estrogen-positive breast cancer and endometrial cancer (deConinck et al. 1995, Turner et al. 1998). TFAP2A, TFAP2C, as well as TFAP2B can directly stimulate the expression of ERBB2, another important breast cancer gene (Bosher et al. 1996). Association of TFAP2A with the YY1 transcription factor significantly increases the ERBB2 transcription rate (Begon et al. 2005). In addition to ERBB2, the expression of another receptor tyrosine kinase, KIT, is also stimulated by TFAP2A and TFAP2B (Huang et al. 1998), while the expression of the VEGF receptor tyrosine kinase ligand VEGFA is repressed by TFAP2A (Ruiz et al. 2004, Li et al. 2012). TFAP2A stimulates transcription of the transforming growth factor alpha (TGFA) gene (Wang et al. 1997). TFAP2C regulates EGFR in luminal breast cancer (De Andrade et al. 2016).

TFAP2C plays a critical role in maintaining the luminal phenotype in human breast cancer and in influencing the luminal cell phenotype during normal mammary development (Cyr et al. 2015).

In placenta, TFAP2A and TFAP2C directly stimulate transcription of both subunits of the human chorionic gonadotropin, CGA and CGB (Johnson et al. 1997, LiCalsi et al. 2000).

TFAP2A and/or TFAP2C, in complex with CITED2, stimulate transcription of the PITX2 gene, involved in left-right patterning and heart development (Bamforth et al. 2004, Li et al. 2012).

TFAP2A and TFAP2C play opposing roles in transcriptional regulation of the CDKN1A (p21) gene locus. While TFAP2A stimulates transcription of the CDKN1A cyclin-dependent kinase inhibitor (Zeng et al. 1997, Williams et al. 2009, Scibetta et al. 2010), TFAP2C represses CDKN1A transcription (Williams et al. 2009, Scibetta et al. 2010, Wong et al. 2012). Transcription of the TFAP2A gene may be inhibited by CREB and E2F1 (Melnikova et al. 2010).

For review of the AP-2 family of transcription factors, please refer to Eckert et al. 2005.

References

Eckert D, Buhl S, Weber S, Jäger R & Schorle H (2005). The AP-2 family of transcription factors. Genome Biol., 6, 246.

Williams T & Tjian R (1991). Analysis of the DNA-binding and activation properties of the human transcription factor AP-2. Genes Dev., 5, 670-82.

Williams T & Tjian R (1991). Characterization of a dimerization motif in AP-2 and its function in heterologous DNA-binding proteins. Science, 251, 1067-71.

Bamforth SD, Bragança J, Eloranta JJ, Murdoch JN, Marques FI, Kranc KR, ... Bhattacharya S (2001). Cardiac malformations, adrenal agenesis, neural crest defects and exencephaly in mice lacking Cited2, a new Tfap2 co-activator. Nat. Genet., 29, 469-74.

Bamforth SD, Bragança J, Farthing CR, Schneider JE, Broadbent C, Michell AC, ... Bhattacharya S (2004). Cited2 controls left-right patterning and heart development through a Nodal-Pitx2c pathway. Nat. Genet., 36, 1189-96.

Edit history

Date	Action	Author
2016-03-14	Edited	Orlic-Milacic M
2016-03-14	Authored	Orlic-Milacic M

Date	Action	Author
2016-03-14	Created	Orlic-Milacic M
2016-05-04	Reviewed	Dawid IB, Zarelli VE
2016-05-17	Reviewed	Bogachek MV, Weigel RJ
2018-08-23	Modified	Schmidt EE

Elements found in this pathway

Input	UniProt Id	Input	UniProt Id	Input	UniProt Id
CDKN1A	P38936	EGFR	P00533	ERBB2	P04626
ESR1	P03372	KIT	P10721	MYC	P01106
VEGFA	P15692				

Input	Ensembl Id	Input	Ensembl Id	Input	Ensembl Id
CDKN1A	ENSG00000124762	EGFR	ENSG00000146648	ERBB2	ENSG00000141736
ESR1	ENSG00000091831	KIT	ENSG00000157404	VEGFA	ENSG00000112715

9. Signal Transduction (R-HSA-162582)

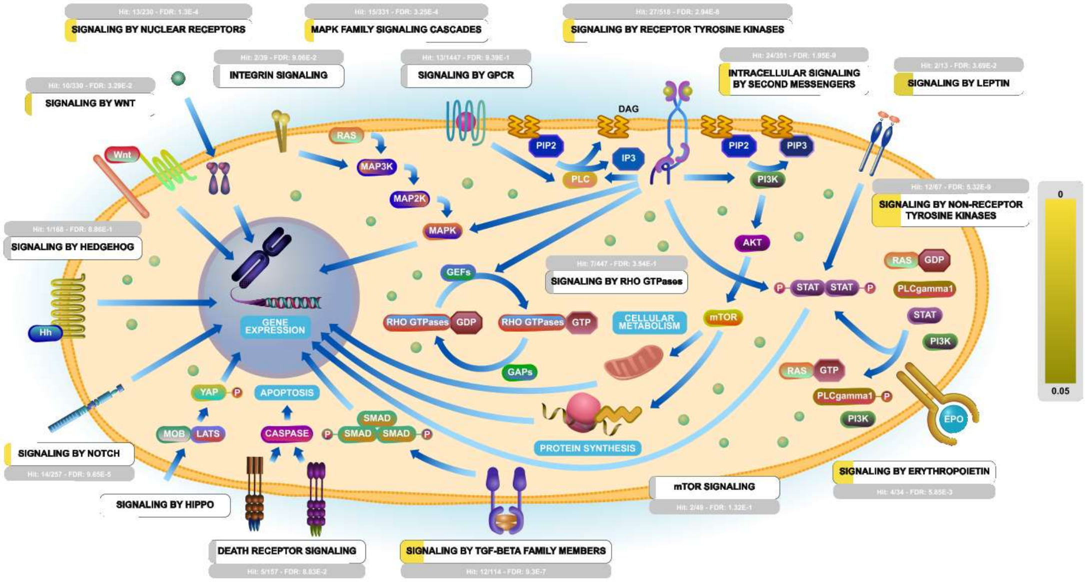

Signal transduction is a process in which extracellular signals elicit changes in cell state and activity. Transmembrane receptors sense changes in the cellular environment by binding ligands, such as hormones and growth factors, or reacting to other types of stimuli, such as light. Stimulation of transmembrane receptors leads to their conformational change which propagates the signal to the intracellular environment by activating downstream signaling cascades. Depending on the cellular context, this may impact cellular proliferation, differentiation, and survival. On the organism level, signal transduction regulates overall growth and behavior.

Receptor tyrosine kinases (RTKs) transmit extracellular signals by phosphorylating their protein partners on conserved tyrosine residues. Some of the best studied RTKs are EGFR (reviewed in Avraham and Yarden, 2011), FGFR (reviewed in Eswarakumar et al, 2005), insulin receptor (reviewed in Saltiel and Kahn, 2001), NGF (reviewed in Reichardt, 2006), PDGF (reviewed in Andrae et al, 2008) and VEGF (reviewed in Xie et al, 2004). RTKs frequently activate downstream signaling through RAF/MAP kinases (reviewed in McKay and Morrison, 2007 and Wellbrock et al 2004), AKT (reviewed in Manning and Cantley, 2007) and PLC- gamma (reviewed in Patterson et al, 2005), which ultimately results in changes in gene expression and cellular metabolism.

Receptor serine/threonine kinases of the TGF-beta family, such as TGF-beta receptors (reviewed in Kang et al. 2009) and BMP receptors (reviewed in Miyazono et al. 2009), transmit extracellular signals by phosphorylating regulatory SMAD proteins on conserved serine and threonine residues. This leads to formation of complexes of regulatory SMADs and SMAD4, which translocate to the nucleus where they act as transcription factors.

WNT receptors transmit their signal through beta-catenin. In the absence of ligand, beta-catenin is constitutively degraded in a ubiquitin-dependent manner. WNT receptor stimulation releases beta-catenin from the destruction complex, allowing it to translocate to the nucleus where it acts as a transcriptional regulator (reviewed in MacDonald et al, 2009 and Angers and Moon, 2009). WNT receptors were originally classified as G-protein coupled receptors (GPCRs). Although they are structurally related, GPCRs primarily transmit their signals through G-proteins, which are trimers of alpha, beta and gamma subunits. When a GPCR is activated, it acts as a guanine nucleotide exchange factor, catalyzing GDP to GTP exchange on the G-alpha subunit of the G protein and its dissociation from the gamma-beta heterodimer. The G-alpha subunit regulates the activity of adenylate cyclase, while the gamma-beta heterodimer can activate AKT and PLC signaling (reviewed in Rosenbaum et al. 2009, Oldham and Hamm 2008, Ritter and Hall 2009).

NOTCH receptors are activated by transmembrane ligands expressed on neighboring cells, which results in cleavage of NOTCH receptor and release of its intracellular domain. NOTCH intracellular domain translocates to the nucleus where it acts as a transcription factor (reviewed in Kopan and Ilagan, 2009).

Integrins are activated by extracellular matrix components, such as fibronectin and collagen, leading to conformational change and clustering of integrins on the cell surface. This results in activation of integrin-linked kinase and other cytosolic kinases and, in co-operation with RTK signaling, regulates survival, proliferation and cell shape and adhesion (reviewed in Hehlgans et al, 2007) .

Besides inducing changes in gene expression and cellular metabolism, extracellular signals that trigger the activation of Rho GTP-ases can trigger changes in the organization of cytoskeleton, thereby regulating cell polarity and cell-cell junctions (reviewed in Citi et al, 2011).

References

Manning BD & Cantley LC (2007). AKT/PKB signaling: navigating downstream. Cell, 129, 1261-74.

Kopan R & Ilagan MXG (2009). The canonical Notch signaling pathway: unfolding the activation mechanism. Cell, 137, 216-33.

Kang JS, Liu C & Derynck R (2009). New regulatory mechanisms of TGF-beta receptor function. Trends Cell Biol, 19, 385-94.

Miyazono K, Kamiya Y & Morikawa M (2010). Bone morphogenetic protein receptors and signal transduction. J Biochem, 147, 35-51.

Avraham R & Yarden Y (2011). Feedback regulation of EGFR signalling: decision making by early and delayed loops. Nat Rev Mol Cell Biol, 12, 104-17.

Edit history

Date	Action	Author
2005-04-01	Created	Joshi-Tope G
2005-05-06	Authored	Joshi-Tope G, Charalambous M, Gopinathrao G, Rothfels K, Bevan AP et al.
2018-08-21	Reviewed	Barroso I, Joutel A, Rush MG, Stanley FM
2018-08-24	Modified	Schmidt EE

Elements found in this pathway

Input	UniProt Id	Input	UniProt Id	Input	UniProt Id
AKT1	Q9Y243, P31749	AKT2	P31751	AURKB	Q96GD4
BCL2	P10415	BCL2L11	O43521	BMI1	P35226
BMPR2	Q13873	CCND1	P24385	CCNE1	P24864
CDC42	P60953	CDK4	P11802	CDKN1A	P38936
CHEK1	O14757	CXCL12	P48061	CXCR4	P61073
E2F1	O00716, Q01094	EGFR	P00533	ERBB2	P04626
ESR1	P03372-3, P03372-4, P03372	EZH2	Q15910	FAS	P25445
FOS	P01100	FOXO1	Q12778	GSK3B	P49841
HIF1A	Q16665	IGF1	P05019	IGF1R	P08069
IL6	P05231	ITGB3	P05106	JAG1	P78504
KIT	P10721	KRAS	P01116, P01111	MAPK1	P28482
MET	P08581	MMP9	P14780	MYC	P01106
NOTCH1	P46531	NRAS	P01111	PAK1	Q16512, Q13153
PHLPP1	O60346	PIK3CA	P42336	PIK3R1	P27986
PTEN	P60484	RARB	P10826	RHOB	P62745
SERPINE1	P05121	SFRP1	Q8N474	SMAD1	Q15797
SMAD2	Q15796	SMAD4	Q13485	SMAD7	O15105
SOCS1	O15524	SOCS3	O14543	SOCS6	O14544
SOX2	P48431	SP1	P08047	STAT3	P40763
SUZ12	Q15022	TGFBR2	P37173	TNFAIP3	P21580
TNFRSF10B	O14763, Q9UBN6	TP53	P04637	VEGFA	P15692-4, P15692
WNT1	P04628	YWHAZ	P63104		

Input	Ensembl Id	Input	Ensembl Id	Input	Ensembl Id
BCL2	ENSG00000171791	CCND1	ENSG00000110092	CXCL12	ENSG00000107562
MMP2	ENSG00000087245	MYC	ENST00000377970, ENSG00000136997	NOTCH1	ENST00000277541, ENSG00000148400
PTEN	ENST00000371953, ENSG00000171862	SERPINE1	ENSG00000106366	SMAD7	ENSG00000101665
SOCS3	ENSG00000184557				

10. TFAP2 (AP-2) family regulates transcription of growth factors and their receptors (R-HSA-8866910)

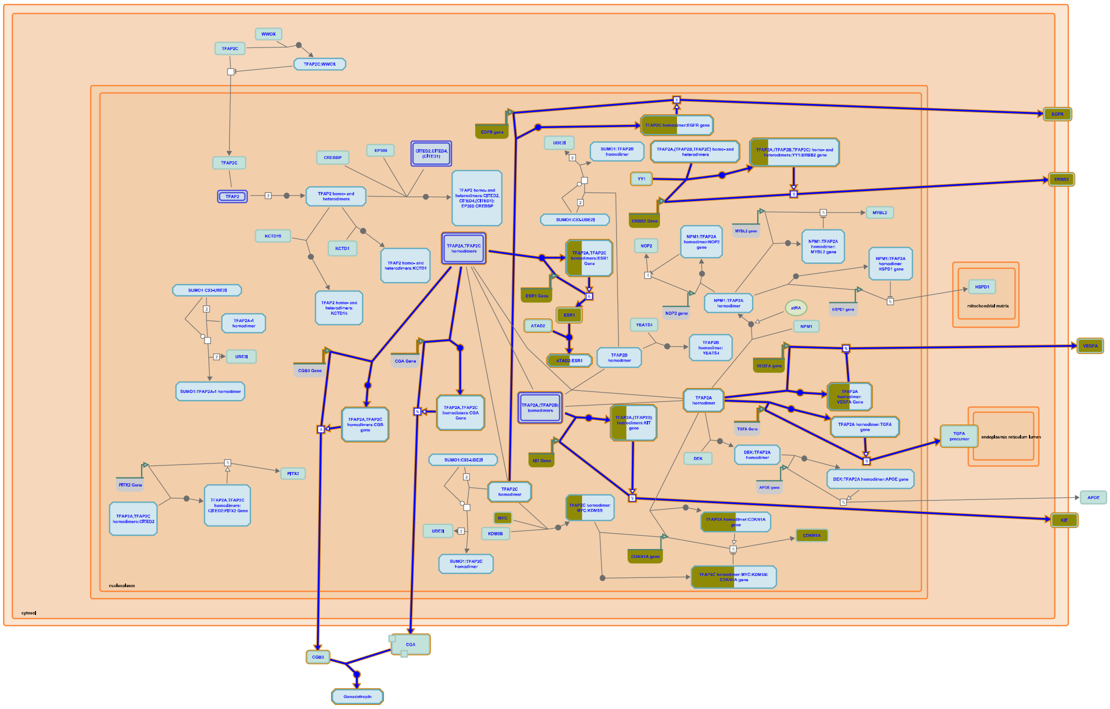

TFAP2A and TFAP2C directly stimulate transcription of the estrogen receptor ESR1 gene (McPherson and Weigel 1999). TFAP2A expression correlates with ESR1 expression in breast cancer, and TFAP2C is frequently overexpressed in estrogen-positive breast cancer and endometrial cancer (deConinck et al. 1995, Turner et al. 1998). TFAP2A, TFAP2C, as well as TFAP2B can directly stimulate the expression of ERBB2, another important breast cancer gene (Bosher et al. 1996). Association of TFAP2A with the YY1 transcription factor significantly increases the ERBB2 transcription rate (Begon et al. 2005). In addition to ERBB2, the expression of another receptor tyrosine kinase, KIT, is also stimulated by TFAP2A and TFAP2B (Huang et al. 1998), while the expression of the VEGF receptor tyrosine kinase ligand VEGFA is repressed by TFAP2A (Ruiz et al. 2004, Li et al. 2012). TFAP2A stimulates transcription of the transforming growth factor alpha (TGFA) gene (Wang et al. 1997). TFAP2C regulates EGFR expression in luminal breast cancer (De Andrade et al. 2016). In placenta, TFAP2A and TFAP2C directly stimulate transcription of both subunits of the human chorionic gonadotropin, CGA and CGB (Johnson et al. 1997, LiCalsi et al. 2000).

References

McPherson LA & Weigel RJ (1999). AP2alpha and AP2gamma: a comparison of binding site specificity and trans-activation of the estrogen receptor promoter and single site promoter constructs. Nucleic Acids Res., 27, 4040-9.

deConinck EC, McPherson LA & Weigel RJ (1995). Transcriptional regulation of estrogen receptor in breast carcinomas. Mol. Cell. Biol., 15, 2191-6.

Turner BC, Zhang J, Gumbs AA, Maher MG, Kaplan L, Carter D, ... Williams T (1998). Expression of AP-2 transcription factors in human breast cancer correlates with the regulation of multiple growth factor signalling pathways. Cancer Res., 58, 5466-72.

Johnson W, Albanese C, Handwerger S, Williams T, Pestell RG & Jameson JL (1997). Regulation of the human chorionic gonadotropin alpha- and beta-subunit promoters by AP-2. J. Biol. Chem., 272, 15405-12.

LiCalsi C, Christophe S, Steger DJ, Buescher M, Fischer W & Mellon PL (2000). AP-2 family members regulate basal and cAMP-induced expression of human chorionic gonadotropin. Nucleic Acids Res., 28, 1036-43.

Edit history

Date	Action	Author
2016-03-14	Edited	Orlic-Milacic M
2016-03-14	Authored	Orlic-Milacic M
2016-04-04	Created	Orlic-Milacic M
2016-05-04	Reviewed	Dawid IB, Zarelli VE
2016-05-17	Reviewed	Bogachek MV, Weigel RJ
2018-08-23	Modified	Schmidt EE

Elements found in this pathway

Input	UniProt Id	Input	UniProt Id	Input	UniProt Id
EGFR	P00533	ERBB2	P04626	ESR1	P03372
KIT	P10721	VEGFA	P15692		

Input	Ensembl Id	Input	Ensembl Id	Input	Ensembl Id
EGFR	ENSG00000146648	ERBB2	ENSG00000141736	ESR1	ENSG00000091831
KIT	ENSG00000157404	VEGFA	ENSG00000112715		

11. PIP3 activates AKT signaling (R-HSA-1257604)

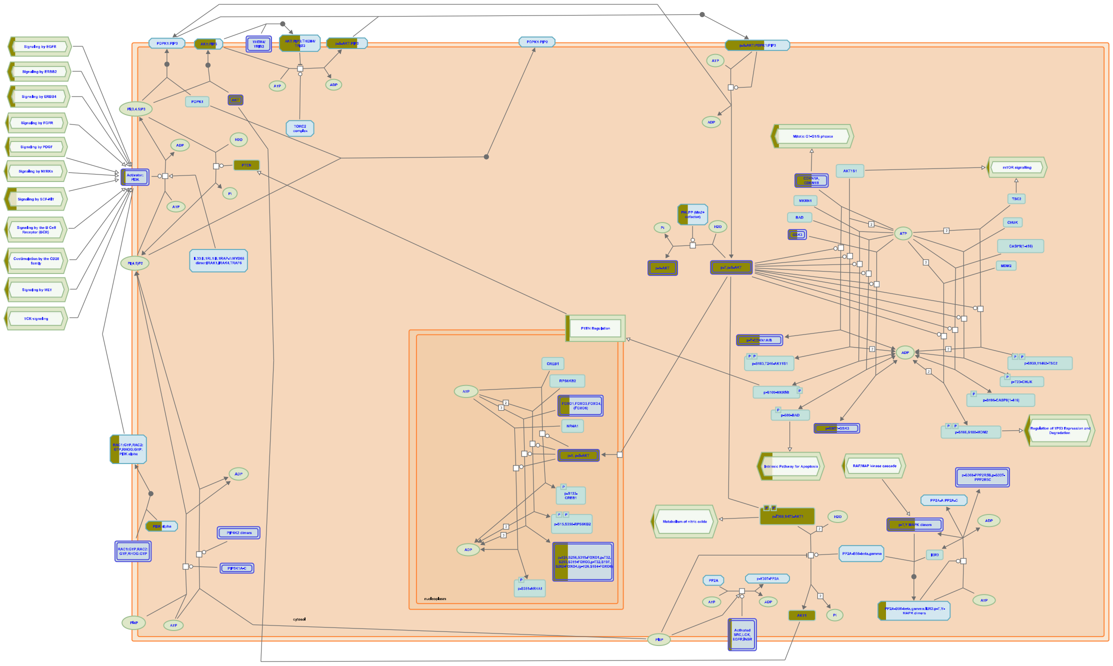

Signaling by AKT is one of the key outcomes of receptor tyrosine kinase (RTK) activation. AKT is activated by the cellular second messenger PIP3, a phospholipid that is generated by PI3K. In ustimulated cells, PI3K class IA enzymes reside in the cytosol as inactive heterodimers composed of p85 regulatory subunit and p110 catalytic subunit. In this complex, p85 stabilizes p110 while inhibiting its catalytic activity. Upon binding of extracellular ligands to RTKs, receptors dimerize and undergo autophosphorylation. The regulatory subunit of PI3K, p85, is recruited to phosphorylated cytosolic RTK domains either directly or indirectly, through adaptor proteins, leading to a conformational change in the PI3K IA heterodimer that relieves inhibition of the p110 catalytic subunit. Activated PI3K IA phosphorylates PIP2, converting it to PIP3; this reaction is negatively regulated by PTEN phosphatase. PIP3 recruits AKT to the plasma membrane, allowing TORC2 to phosphorylate a conserved serine residue of AKT. Phosphorylation of this serine induces a conformation change in AKT, exposing a conserved threonine residue that is then phosphorylated by PDPK1 (PDK1). Phosphorylation of both the threonine and the serine residue is required to fully activate AKT. The active AKT then dissociates from PIP3 and phosphorylates a number of cytosolic and nuclear proteins that play important roles in cell survival and metabolism. For a recent review of AKT signaling, please refer to Manning and Cantley, 2007.

Edit history

Date	Action	Author
2007-11-08	Reviewed	Greene LA
2011-05-02	Created	Orlic-Milacic M
2012-06-21	Revised	Orlic-Milacic M
2012-08-13	Reviewed	Yuzugullu H, Thorpe L, Zhao JJ
2018-08-24	Modified	Schmidt EE

Elements found in this pathway

Input	UniProt Id	Input	UniProt Id	Input	UniProt Id
AKT1	Q9Y243, P31749	AKT2	P31751	BMI1	P35226
CDKN1A	P38936	EGFR	P00533	ERBB2	P04626
ESR1	P03372-3, P03372-4, P03372	EZH2	Q15910	FOXO1	Q12778
GSK3B	P49841	KIT	P10721	MAPK1	P28482
MET	P08581	PHLPP1	O60346	PIK3CA	P42336
PIK3R1	P27986	PTEN	P60484	SUZ12	Q15022
TP53	P04637				

Input	Ensembl Id	Input	Ensembl Id	Input	Ensembl Id
PTEN	ENST00000371953, ENSG00000171862				

12. Diseases of signal transduction (R-HSA-5663202)

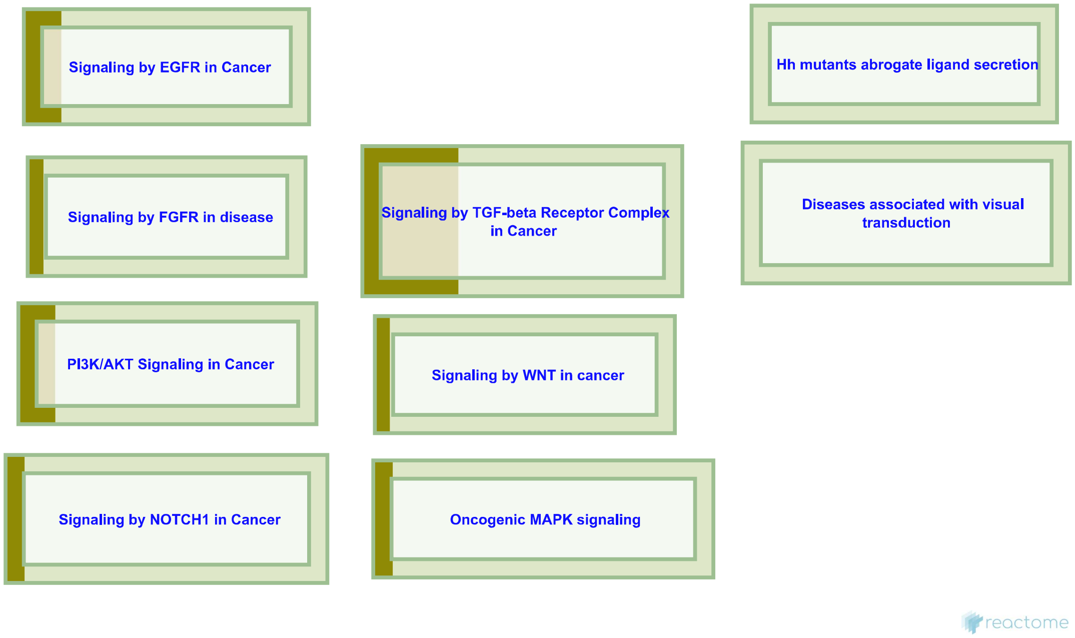

Signaling processes are central to human physiology (e.g., Pires-da Silva & Sommer 2003), and their disruption by either germ-line and somatic mutation can lead to serious disease. Here, the molecular consequences of mutations affecting visual signal transduction and signaling by diverse growth factors are annotated.

References

Pires-daSilva A & Sommer RJ (2003). The evolution of signalling pathways in animal development. Nat. Rev. Genet., 4, 39-49.

Edit history

Date	Action	Author
2015-01-16	Created	D'Eustachio P
2016-11-08	Modified	Shorser S

Elements found in this pathway

Input	UniProt Id	Input	UniProt Id	Input	UniProt Id
AKT1	Q9Y243, P31749	AKT2	P31751	BCL2L11	O43521
CDKN1A	P38936	EGFR	P00533	ERBB2	P04626
ESR1	P03372-3, P03372-4, P03372	FOXO1	Q12778	GSK3B	P49841
ITGB3	P05106	JAG1	P78504	KIT	P10721
KRAS	P01116, P01111	MAPK1	P28482	MET	P08581
MYC	P01106	NOTCH1	P46531	NRAS	P01111
PIK3CA	P42336	PIK3R1	P27986	PTEN	P60484
SMAD2	Q15796	SMAD4	Q13485	STAT3	P40763
TGFBR2	P37173				

Input	Ensembl Id	Input	Ensembl Id	Input	Ensembl Id
MYC	ENSG00000136997				

13. Transcriptional regulation by RUNX3 (R-HSA-8878159)

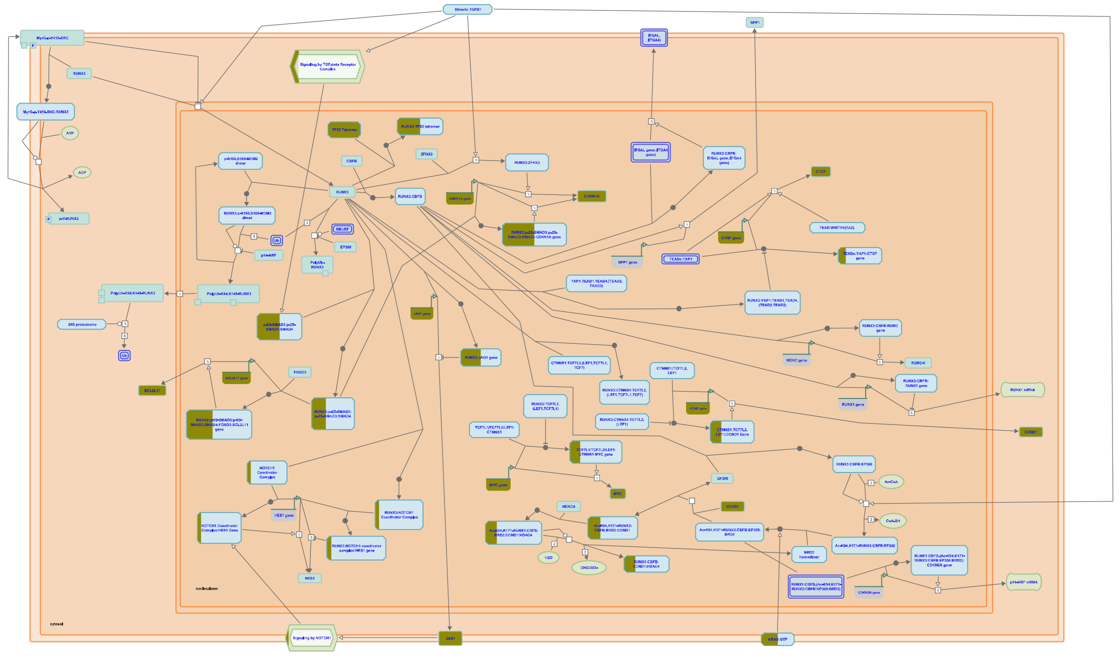

The transcription factor RUNX3 is a RUNX family member. All RUNX family members, RUNX1, RUNX2 and RUNX3, possess a highly conserved Runt domain, involved in DNA binding. For a more detailed description of the structure of RUNX proteins, please refer to the pathway 'Transcriptional regulation by RUNX1'. Similar to RUNX1 and RUNX2, RUNX3 forms a transcriptionally active heterodimer with CBFB (CBF-beta). Studies in mice have shown that RUNX3 plays a role in neurogenesis and development of T lymphocytes. RUNX3 is implicated as a tumor suppressor gene in various human malignancies.

During nervous system formation, the Cbfb:Runx3 complex is involved in development of mouse proprioceptive dorsal root ganglion neurons by regulating expression of Ntrk3 (Neurotrophic tyrosine kinase receptor type 3) and possibly other genes (Inoue et al. 2002, Kramer et al. 2006, Nakamura et al. 2008, Dykes et al. 2011, Ogihara et al. 2016). It is not yet known whether RUNX3 is involved in human neuronal development and neuronal disorders.

RUNX3 plays a major role in immune response. RUNX3 regulates development of T lymphocytes. In mouse hematopoietic stem cells, expression of Runx3 is regulated by the transcription factor TAL1 (Landry et al. 2008). RUNX3 promotes the CD8+ lineage fate in developing thymocytes. In the CD4+ thymocyte lineage in mice, the transcription factor ThPOK induces transcription of SOCS family members, which repress Runx3 expression (Luckey et al. 2014). RUNX3, along with RUNX1 and ETS1, is implicated in regulation of transcription of the CD6 gene, encoding a lymphocyte surface receptor expressed on developing and mature T cells (Arman et al. 2009). RUNX3 and ThPOK regulate intestinal CD4+ T cell immunity in a TGF-beta and retinoic acid-dependent manner, which is important for cellular defense against intestinal pathogens (Reis et al. 2013). Besides T lymphocytes, RUNX3 is a key transcription factor in the commitment of innate lymphoid cells ILC1 and ILC3 (Ebihara et al. 2015). RUNX3 regulates expression of CD11A and CD49D integrin genes, involved in immune and inflammatory responses (Dominguez-Soto et al. 2005). RUNX3 is involved in mouse TGF-beta-mediated dendritic cell function and its deficiency is linked to airway inflammation (Fainaru et al. 2004).

In addition to its developmental role, RUNX3 is implicated as a tumor suppressor. The loss of RUNX3 expression and function was first causally linked to the genesis and progression of human gastric cancer (Li et al. 2002). Expression of RUNX3 increases in human pancreatic islet of Langerhans cells but not in pancreatic adenocarcinoma cells in response to differentiation stimulus (serum withdrawal) (Levkovitz et al. 2010). Hypermethylation of the RUNX3 gene is associated with an increased risk for progression of Barrett's esophagus to esophageal adenocarcinoma (Schulmann et al. 2005). Hypermethylation-mediated silencing of the RUNX3 gene expression is also frequent in granulosa cell tumors (Dhillon et al. 2004) and has also been reported in colon cancer (Weisenberger et al. 2006), breast cancer (Lau et al. 2006, Huang et al. 2012), bladder cancer (Wolff et al. 2008) and gastric cancer (Li et al. 2002). In colorectal cancer, RUNX3 is one of the five markers in a gene panel used to classify CpG island methylator phenotype (CIMP+) (Weisenberger et al. 2006).

RUNX3 and CBFB are frequently downregulated in gastric cancer. RUNX3 cooperates with TGF-beta to maintain homeostasis in the stomach and is involved in TGF-beta-induced cell cycle arrest of stomach epithelial cells. Runx3 knockout mice exhibit decreased sensitivity to TGF-beta and develop gastric epithelial hyperplasia (Li et al. 2002, Chi et al. 2005). RUNX3-mediated inhibition of binding of TEADs:YAP1 complexes to target promoters is also implicated in gastric cancer suppression (Qiao et al. 2016).

RUNX3 is a negative regulator of NOTCH signaling and RUNX3-mediated inhibition of NOTCH activity may play a tumor suppressor role in hepatocellular carcinoma (Gao et al. 2010, Nishina et al. 2011).

In addition to RUNX3 silencing through promoter hypermethylation in breast cancer (Lau et al. 2006), Runx3+/- mice are predisposed to breast cancer development. RUNX3 downregulates estrogen receptor alpha (ESR1) protein levels in a proteasome-dependent manner (Huang et al. 2012).

Besides its tumor suppressor role, mainly manifested through its negative effect on cell proliferation, RUNX3 can promote cancer cell invasion by stimulating expression of genes involved in metastasis, such as osteopontin (SPP1) (Whittle et al. 2015).

References

Ogihara Y, Masuda T, Ozaki S, Yoshikawa M & Shiga T (2016). Runx3-regulated expression of two Ntrk3 transcript variants in dorsal root ganglion neurons. Dev Neurobiol, 76, 313-22.

Dykes IM, Tempest L, Lee SI & Turner EE (2011). Brn3a and Islet1 act epistatically to regulate the gene expression program of sensory differentiation. J. Neurosci., 31, 9789-99.

Luckey MA, Kimura MY, Waickman AT, Feigenbaum L, Singer A & Park JH (2014). The transcription factor ThPOK suppresses Runx3 and imposes CD4(+) lineage fate by inducing the SOCS suppressors of cytokine signaling. Nat. Immunol., 15, 638-45.

Levkovitz L, Yosef N, Gershengorn MC, Ruppin E, Sharan R & Oron Y (2010). A novel HMM-based method for detecting enriched transcription factor binding sites reveals RUNX3 as a potential target in pancreatic cancer biology. PLoS ONE, 5, e14423.

Arman M, Aguilera-Montilla N, Mas V, Puig-Kröger A, Pignatelli M, Guigó R, ... Lozano F (2009). The human CD6 gene is transcriptionally regulated by RUNX and Ets transcription factors in T cells. Mol. Immunol., 46, 2226-35.

Edit history

Date	Action	Author
2016-06-30	Created	Orlic-Milacic M
2016-12-13	Authored	Orlic-Milacic M
2017-01-31	Edited	Orlic-Milacic M
2017-01-31	Reviewed	Ito Y, Chuang LS
2018-08-23	Modified	Schmidt EE

Elements found in this pathway

Input	UniProt Id	Input	UniProt Id	Input	UniProt Id
BCL2L11	O43521	CCND1	P24385	CDKN1A	P38936
CTGF	P29279	JAG1	P78504	KRAS	P01116
MYC	P01106	NOTCH1	P46531	SMAD4	Q13485
TP53	P04637				

Input	Ensembl Id	Input	Ensembl Id	Input	Ensembl Id
BCL2L11	ENSG00000153094	CCND1	ENSG00000110092	CDKN1A	ENSG00000124762
CTGF	ENSG00000118523	JAG1	ENSG00000101384	MYC	ENSG00000136997

14. Intrinsic Pathway for Apoptosis (R-HSA-109606)

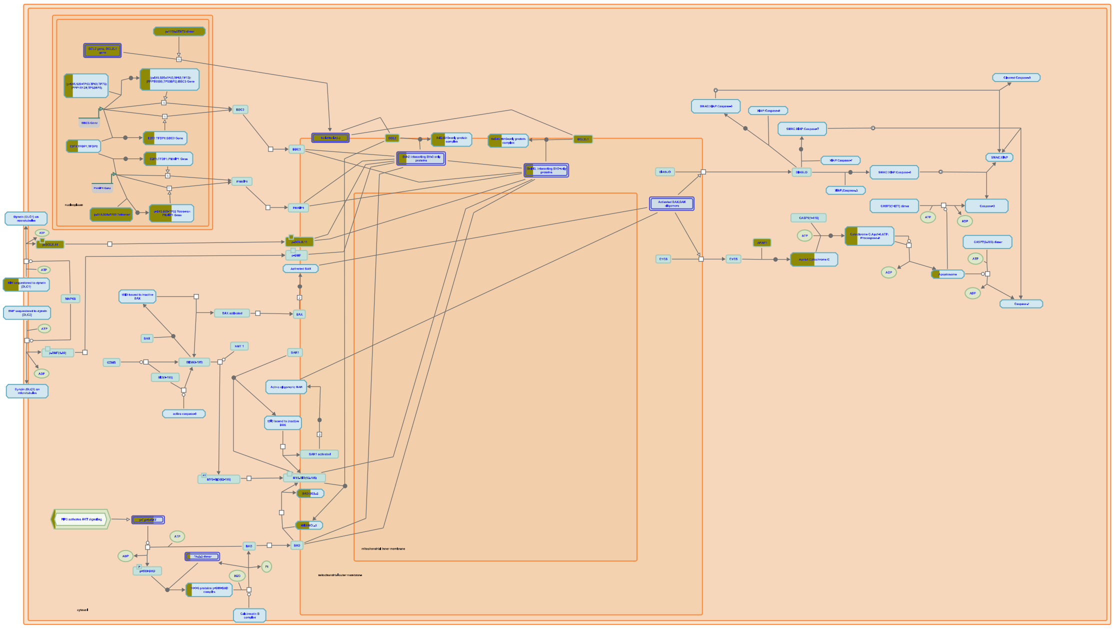

The intrinsic (Bcl-2 inhibitable or mitochondrial) pathway of apoptosis functions in response to various types of intracellular stress including growth factor withdrawal, DNA damage, unfolding stresses in the endoplasmic reticulum and death receptor stimulation. Following the reception of stress signals, proapoptotic BCL-2 family proteins are activated and subsequently interact with and inactivate antiapoptotic BCL-2 proteins. This interaction leads to the destabilization of the mitochondrial membrane and release of apoptotic factors. These factors induce the caspase proteolytic cascade, chromatin condensation, and DNA fragmentation, ultimately leading to cell death. The key players in the Intrinsic pathway are the Bcl-2 family of proteins that are critical death regulators residing immediately upstream of mitochondria. The Bcl-2 family consists of both anti- and proapoptotic members that possess conserved alpha-helices with sequence conservation clustered in BCL-2 Homology (BH) domains. Proapoptotic members are organized as follows:

1. "Multidomain" BAX family proteins such as BAX, BAK etc. that display sequence conservation in their BH1-3 regions. These proteins act downstream in mitochondrial disruption.

2. "BH3-only" proteins such as BID,BAD, NOXA, PUMA,BIM, and BMF have only the short BH3 motif. These act upstream in the pathway, detecting developmental death cues or intracellular damage. Anti-apoptotic members like Bcl-2, Bcl-XL and their relatives exhibit homology in all segments BH1-4. One of the critical functions of BCL-2/BCL-XL proteins is to maintain the integrity of the mitochondrial outer membrane.

References

Saelens X, Festjens N, Vande Walle L, van Gurp M, van Loo G & Vandenabeele P (2004). Toxic proteins released from mitochondria in cell death. Oncogene, 23, 2861-74.

Wang X (2001). The expanding role of mitochondria in apoptosis. Genes Dev, 15, 2922-33.

Salvesen GS & Duckett CS (2002). IAP proteins: blocking the road to death's door. Nat Rev Mol Cell Biol, 3, 401-10.

Edit history

Date	Action	Author
2004-02-17	Created	Alnemri E
2004-08-06	Authored	Matthews L
2018-08-24	Modified	Schmidt EE

Elements found in this pathway

Input	UniProt Id	Input	UniProt Id	Input	UniProt Id
AKT1	P31749	AKT2	P31751	APAF1	O14727
BCL2	P10415	BCL2L1	Q07817	BCL2L11	O43521
E2F1	Q01094	STAT3	P40763	TP53	P04637
YWHAZ	P63104				

Input	Ensembl Id	Input	Ensembl Id	Input	Ensembl Id
BCL2	ENSG00000171791	BCL2L1	ENSG00000171552		

15. Transcriptional Regulation by TP53 (R-HSA-3700989)

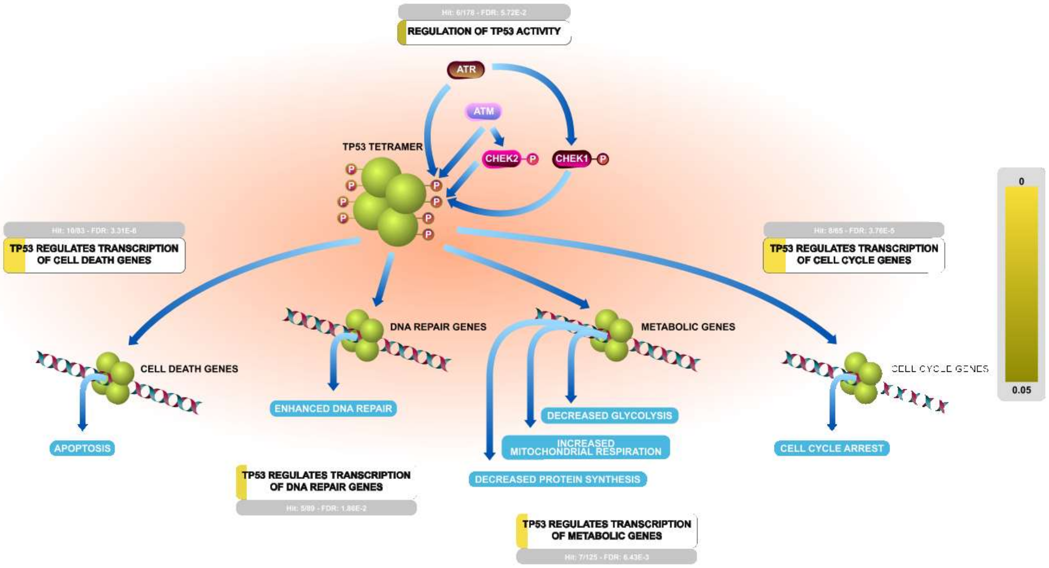

The tumor suppressor TP53 (encoded by the gene p53) is a transcription factor. Under stress conditions, it recognizes specific responsive DNA elements and thus regulates the transcription of many genes involved in a variety of cellular processes, such as cellular metabolism, survival, senescence, apoptosis and DNA damage response. Because of its critical function, p53 is frequently mutated in around 50% of all malignant tumors. For a recent review, please refer to Vousden and Prives 2009 and Kruiswijk et al. 2015.

References

Vousden KH & Prives C (2009). Blinded by the Light: The Growing Complexity of p53. Cell, 137, 413-31.

Kruiswijk F, Labuschagne CF & Vousden KH (2015). p53 in survival, death and metabolic health: a lifeguard with a licence to kill. Nat. Rev. Mol. Cell Biol., 16, 393-405.

Edit history

Date	Action	Author
2013-06-11	Created	Orlic-Milacic M
2015-10-14	Edited	Orlic-Milacic M
2015-10-14	Authored	Orlic-Milacic M
2016-02-04	Reviewed	Zaccara S, Inga A
2018-08-24	Modified	Schmidt EE

Elements found in this pathway

Input	UniProt Id	Input	UniProt Id	Input	UniProt Id
AKT1	P31749	AKT2	P31751	APAF1	O14727
AURKB	Q96GD4	BCL6	P41182	CCNE1	P24864
CCNE2	O96020	CDKN1A	P38936	CHEK1	O14757
E2F1	Q01094	FAS	P25445	FOS	P01100
PTEN	P60484	TNFRSF10B	O14763, Q9UBN6	TP53	P04637

Input	UniProt Id	Input	UniProt Id	Input	UniProt Id
YWHAZ	P63104				

Input	Ensembl Id	Input	Ensembl Id	Input	Ensembl Id
APAF1	ENSG00000120868	BCL6	ENSG00000113916	CDKN1A	ENST00000244741, ENSG00000124762
CHEK1	ENST00000438015, ENSG00000149554	E2F1	ENSG00000101412	FAS	ENSG00000026103
PTEN	ENST00000371953, ENSG00000171862	TNFRSF10B	ENSG00000120889	TP53	ENSG00000141510

16. PI3K/AKT Signaling in Cancer (R-HSA-2219528)

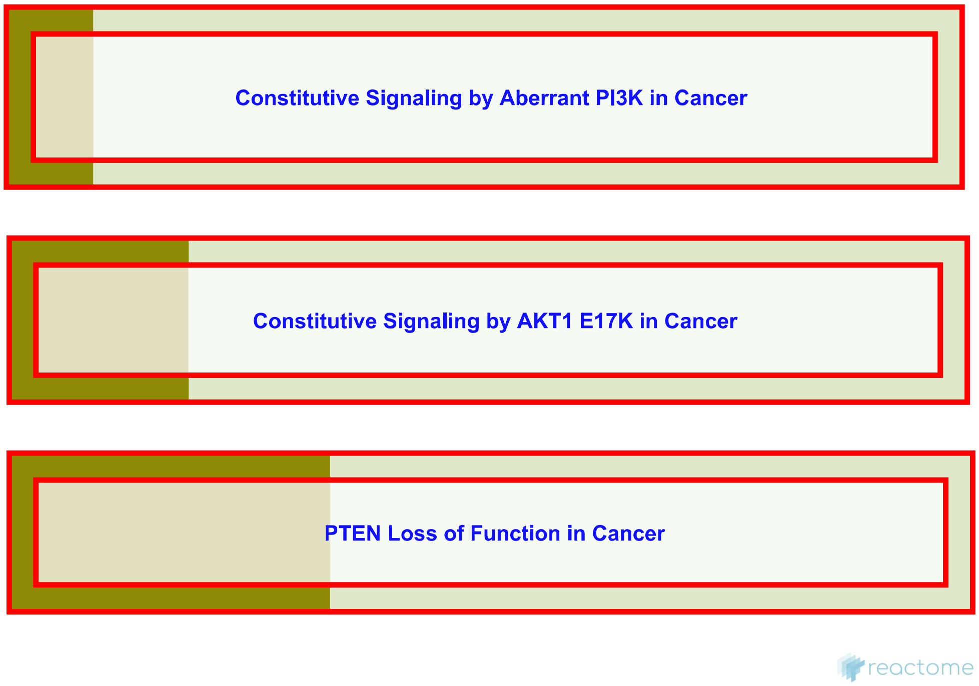

Diseases: cancer.

Class IA PI3K is a heterodimer of a p85 regulatory subunit (encoded by PIK3R1, PIK3R2 or PIK3R3) and a p110 catalytic subunit (encoded by PIK3CA, PIK3CB or PIK3CD). In the absence of activating signals, the regulatory subunit stabilizes the catalytic subunit while inhibiting its activity. The complex becomes activated when extracellular signals stimulate the phosphorylation of the cytoplasmic domains of transmembrane receptors or receptor-associated proteins. The p85 regulatory subunit binds phosphorylated motifs of activator proteins, which induces a conformational change that relieves p85-mediated inhibition of the p110 catalytic subunit and enables PI3K to phosphorylate PIP2 to form PIP3. The phosphoinositide kinase activity of PI3K is opposed by the phosphoinositide phosphatase activity of PTEN.

PIP3 acts as a messenger that recruits PDPK1 (PDK1) and AKT (AKT1, AKT2 or AKT3) to the plasma membrane. PDPK1 also possesses a low affinity for PIP2, so small amounts of PDPK1 are always present at the membrane. Binding of AKT to PIP3 induces a conformational change that enables TORC2 complex to phosphorylate AKT at a conserved serine residue (S473 in AKT1). Phosphorylation at the serine residue enables AKT to bind to PDPK1 and exposes a conserved threonine residue (T308) that is phosphorylated by PDPK1. AKT phosphorylated at both serine and threonine residues dissociates from the plasma membrane and acts as a serine/threonine kinase that phosphorylates a number of cytosolic and nuclear targets involved in regulation of cell metabolism, survival and gene expression. For a recent review, please refer to Manning and Cantley, 2007.

Signaling by PI3K/AKT is frequently constitutively activated in cancer. This activation can be via gain-of-function mutations in PI3KCA (encoding catalytic subunit p110alpha), PIK3R1 (encoding regulatory subunit p85alpha) and AKT1. The PI3K/AKT pathway can also be constitutively activated by loss-of-function mutations in tumor suppressor genes such as PTEN.

Gain-of-function mutations activate PI3K signaling by diverse mechanisms. Mutations affecting the helical domain of PIK3CA and mutations affecting nSH2 and iSH2 domains of PIK3R1 impair inhibitory interactions between these two subunits while preserving their association. Mutations in the catalytic domain of PIK3CA enable the kinase to achieve an active conformation. PI3K complexes with gain-of-function mutations therefore produce PIP3 and activate downstream AKT in the absence of growth factors (Huang et al. 2007, Zhao et al. 2005, Miled et al. 2007, Horn et al. 2008, Sun et al. 2010, Jaiswal et al. 2009, Zhao and Vogt 2010, Urick et al. 2011). While AKT1 gene copy number, expression level and phosphorylation are often increased in cancer, only one low frequency point mutation has been repeatedly reported in cancer and functionally studied. This mutation represents a substitution of a glutamic acid residue with lysine at position 17 of AKT1, and acts by enabling AKT1 to bind PIP2. PIP2-bound AKT1 is phosphorylated by TORC2 complex and by PDPK1 that is always present at the plasma membrane, due to low affinity for PIP2. Therefore, E17K substitution abrogates the need for PI3K in AKT1 activation (Carpten et al. 2007, Landgraf et al. 2008).

Loss-of-function mutations affecting the phosphatase domain of PTEN are frequently found in sporadic cancers (Kong et al. 1997, Lee et al. 1999, Han et al. 2000), as well as in PTEN hamartoma tumor syndromes (PHTS) (Marsh et al. 1998). PTEN can also be inactivated by gene deletion or epigenetic silencing, or indirectly by overexpression of microRNAs that target PTEN mRNA (Huse et al. 2009). Cells with deficient PTEN function have increased levels of PIP3, and therefore increased AKT activity. For a recent review, please refer to Hollander et al. 2011.

Because of their clear involvement in human cancers, PI3K and AKT are targets of considerable interest in the development of small molecule inhibitors. Although none of the currently available inhibitors display preference for mutant variants of PIK3CA or AKT, several inhibitors targeting the wild-type kinases are undergoing clinical trials. These include dual PI3K/mTOR inhibitors, class I PI3K inhibitors, pan-PI3K inhibitors, and pan-AKT inhibitors. While none have yet been approved for clinical use, these agents show promise for future therapeutics. In addition, isoform-specific PI3K and AKT inhibitors are currently being developed, and may provide more specific treatments along with reduced side-effects. For a recent review, please refer to Liu et al. 2009.

References

Manning BD & Cantley LC (2007). AKT/PKB signaling: navigating downstream. Cell, 129, 1261-74.

Liu P, Cheng H, Roberts TM & Zhao JJ (2009). Targeting the phosphoinositide 3-kinase pathway in cancer. Nat Rev Drug Discov, 8, 627-44.

Hollander MC, Blumenthal GM & Dennis PA (2011). PTEN loss in the continuum of common cancers, rare syndromes and mouse models. Nat. Rev. Cancer, 11, 289-301.

Huang CH, Mandelker D, Schmidt-Kittler O, Samuels Y, Velculescu VE, Kinzler KW, ... Amzel LM (2007). The structure of a human p110alpha/p85alpha complex elucidates the effects of oncogenic PI3Kalpha mutations. Science, 318, 1744-8.

Zhao JJ, Liu Z, Wang L, Shin E, Loda MF & Roberts TM (2005). The oncogenic properties of mutant p110alpha and p110beta phosphatidylinositol 3-kinases in human mammary epithelial cells. Proc. Natl. Acad. Sci. U.S.A., 102, 18443-8.

Edit history

Date	Action	Author
2012-05-01	Created	Orlic-Milacic M

Date	Action	Author
2012-07-18	Authored	Orlic-Milacic M
2012-08-03	Edited	Matthews L
2012-08-13	Reviewed	Yuzugullu H, Thorpe L, Zhao JJ
2015-02-12	Modified	Orlic-Milacic M

Elements found in this pathway

Input	UniProt Id	Input	UniProt Id	Input	UniProt Id
AKT1	Q9Y243, P31749	AKT2	P31751	CDKN1A	P38936
EGFR	P00533	ERBB2	P04626	ESR1	P03372-3, P03372-4, P03372
FOXO1	Q12778	GSK3B	P49841	KIT	P10721
MET	P08581	PIK3CA	P42336	PIK3R1	P27986
PTEN	P60484				

17. Intracellular signaling by second messengers (R-HSA-9006925)

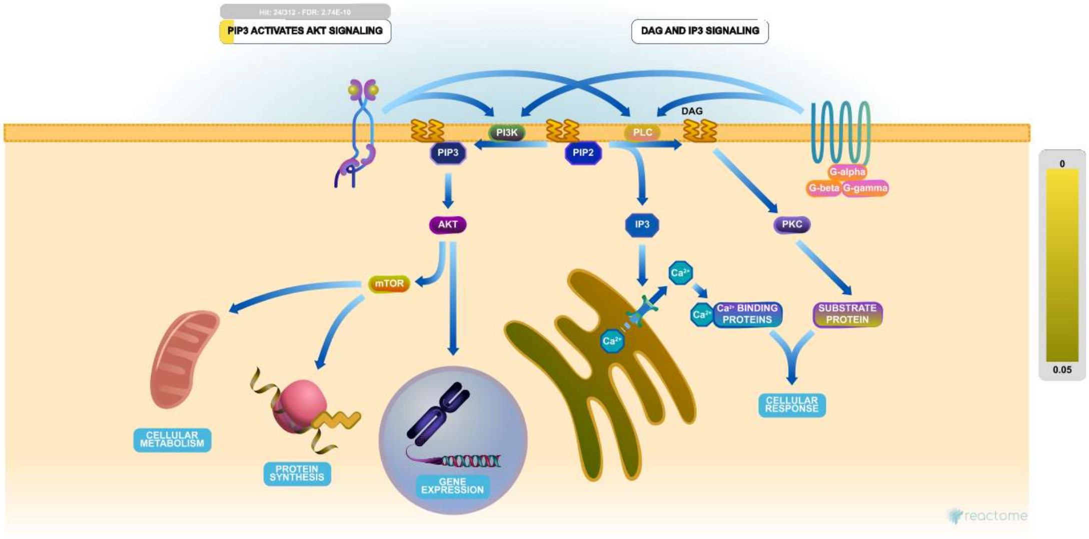

Second messengers are generated within the cell as a downstream step in signal transduction cascades initiated by the interaction of an external stimulus with a cell surface receptor. Common second messengers include DAG, cAMP, cGMP, IP3, Ca2+ and phosphatidylinositols (reviewed in Kang et al, 2015; Raker et al, 2016; Li and Marshall, 2015; Pinto et al, 2015; Ahmad et al, 2015).

References

Ahmad F, Murata T, Shimizu K, Degerman E, Maurice D & Manganiello V (2015). Cyclic nucleotide phosphodiesterases: important signaling modulators and therapeutic targets. Oral Dis, 21, e25-50.

Raker VK, Becker C & Steinbrink K (2016). The cAMP Pathway as Therapeutic Target in Autoimmune and Inflammatory Diseases. Front Immunol, 7, 123.

Pinto MC, Kihara AH, Goulart VA, Tonelli FM, Gomes KN, Ulrich H & Resende RR (2015). Calcium signaling and cell proliferation. Cell. Signal., 27, 2139-49.

Kang DS, Yang YR, Lee C, Kim S, Ryu SH & Suh PG (2016). Roles of phosphoinositide-specific phospholipase C1 in brain development. Adv Biol Regul, 60, 167-73.

Levine TP & Patel S (2016). Signalling at membrane contact sites: two membranes come together to handle second messengers. Curr. Opin. Cell Biol., 39, 77-83.

Edit history

Date	Action	Author
2017-05-24	Edited	Rothfels K
2017-05-24	Authored	Rothfels K
2017-05-24	Created	Rothfels K
2017-06-22	Reviewed	D'Eustachio P
2018-08-24	Modified	Schmidt EE

Elements found in this pathway

Input	UniProt Id	Input	UniProt Id	Input	UniProt Id
AKT1	Q9Y243, P31749	AKT2	P31751	BMI1	P35226
CDKN1A	P38936	EGFR	P00533	ERBB2	P04626
ESR1	P03372-3, P03372-4, P03372	EZH2	Q15910	FOXO1	Q12778
GSK3B	P49841	KIT	P10721	MAPK1	P28482
MET	P08581	PHLPP1	O60346	PIK3CA	P42336
PIK3R1	P27986	PTEN	P60484	SUZ12	Q15022
TP53	P04637				

Input	Ensembl Id	Input	Ensembl Id	Input	Ensembl Id
PTEN	ENST00000371953, ENSG00000171862				

18. Signaling by Non-Receptor Tyrosine Kinases (R-HSA-9006927)

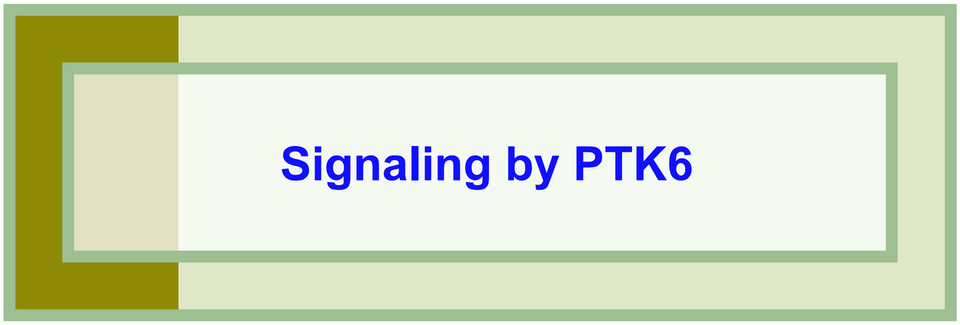

In addition to receptor tyrosine kinases, the human genome encodes at least 32 non-receptor tyrosine kinases (non-RTKs). These cytosolic tyrosine kinases lack a transmembrane domain but are recruited into signal transduction cascades through interaction with other plasma-bound receptors, which may or may not themselves have intrinsic catalytic activity. In this way, non-RTKs essentially function as an (additional) enzymatic subunit of the signaling complex and contribute to many of the same downstream signaling pathways. The non-RTKs can be grouped into 9 families (ABL, FES, SYK, JAK, TEC, FAK, ACK, SRC, FRK and CSK) based on their domain structure (reviewed in Neet and Hunter, 1996).

References

Neet K & Hunter T (1996). Vertebrate non-receptor protein-tyrosine kinase families. Genes Cells, 1, 147-69.

Edit history

Date	Action	Author
2017-05-24	Edited	Rothfels K
2017-05-24	Authored	Rothfels K
2017-05-24	Created	Rothfels K
2017-06-22	Reviewed	D'Eustachio P
2018-08-24	Modified	Schmidt EE

Elements found in this pathway

Input	UniProt Id	Input	UniProt Id	Input	UniProt Id
AKT1	P31749	CCND1	P24385	CCNE1	P24864
CDK4	P11802	EGFR	P00533	ERBB2	P04626
HIF1A	Q16665	KRAS	P01116, P01111	NRAS	P01111
SOCS3	O14543	STAT3	P40763		

Input	Ensembl Id	Input	Ensembl Id	Input	Ensembl Id
SOCS3	ENSG00000184557				

19. Signaling by PTK6 (R-HSA-8848021)

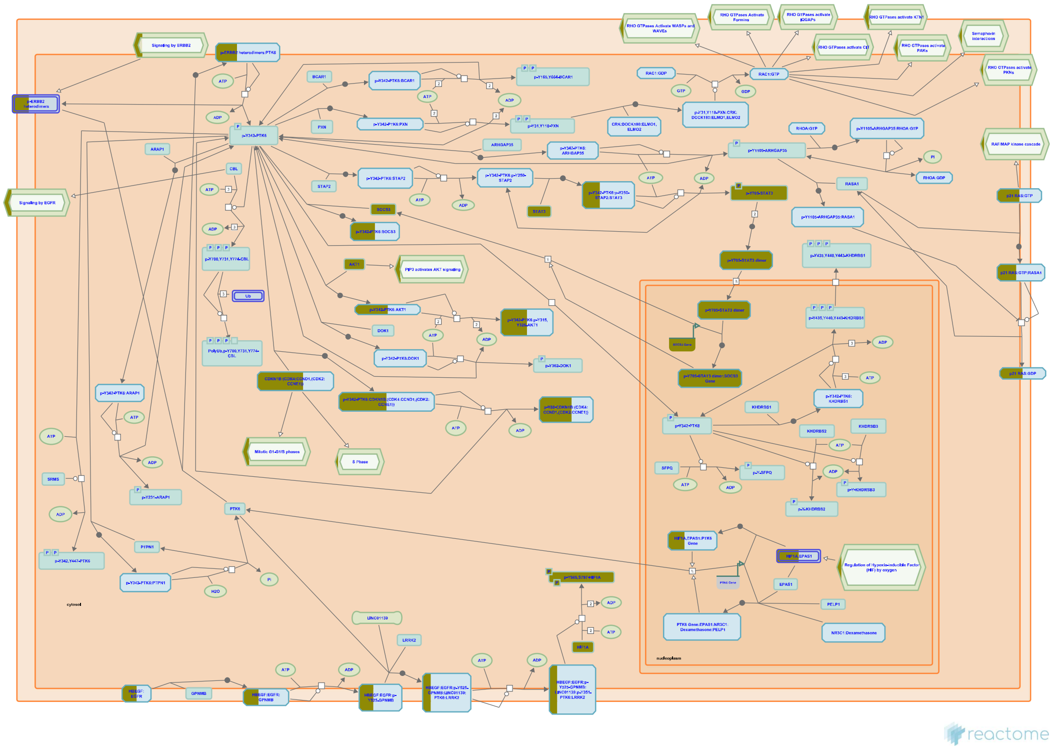

PTK6 (BRK) is an oncogenic non-receptor tyrosine kinase that functions downstream of ERBB2 (HER2) (Xiang et al. 2008, Peng et al. 2015) and other receptor tyrosine kinases, such as EGFR (Kamalati et al. 1996) and MET (Castro and Lange 2010). Since ERBB2 forms heterodimers with EGFR and since MET can heterodimerize with both ERBB2 and EGFR (Tanizaki et al. 2011), it is not clear if MET and EGFR activate PTK6 directly or act through ERBB2. Levels of PTK6 increase under hypoxic conditions (Regan Anderson et al. 2013, Pires et al. 2014). The kinase activity of PTK6 is negatively regulated by PTPN1 phosphatase (Fan et al. 2013) and SRMS kinase (Fan et al. 2015), as well as the STAT3 target SOCS3 (Gao et al. 2012).

PTK6 activates STAT3-mediated transcription (Ikeda et al. 2009, Ikeda et al. 2010) and may also activate STAT5-mediated transcription (Ikeda et al. 2011). PTK6 promotes cell motility and migration by regulating the activity of RHO GTPases RAC1 (Chen et al. 2004) and RHOA (Shen et al. 2008), and possibly by affecting motility-related kinesins (Lukong and Richard 2008). PTK6 crosstalks with AKT1 (Zhang et al. 2005, Zheng et al. 2010) and RAS signaling cascades (Shen et al. 2008, Ono et al. 2014) and may be involved in MAPK7 (ERK5) activation (Ostrander et al. 2007, Zheng et al. 2012). PTK6 enhances EGFR signaling by inhibiting EGFR down-regulation (Kang et al. 2010, Li et al. 2012, Kang and Lee 2013). PTK6 may also enhance signaling by IGF1R (Fan et al. 2013) and ERBB3 (Kamalati et al. 2000).

PTK6 promotes cell cycle progression by phosphorylating and inactivating CDK inhibitor CDKN1B (p27) (Patel et al. 2015).

PTK6 activity is upregulated in osteopontin (OPN or SPP1)-mediated signaling, leading to increased VEGF expression via PTK6/NF-kappaB/ATF4 signaling path. PTK6 may therefore play a role in VEGF-dependent tumor angiogenesis (Chakraborty et al. 2008).

PTK6 binds and phosphorylates several nuclear RNA-binding proteins, including SAM68 family members (KHDRSB1, KHDRSB2 and KHDRSB3) (Derry et al. 2000, Haegebarth et al. 2004, Lukong et al. 2005) and SFPQ (PSF) (Lukong et al. 2009). The biological role of PTK6 in RNA processing is not known.

For a review of PTK6 function, please refer to Goel and Lukong 2015.

References

Xiang B, Chatti K, Qiu H, Lakshmi B, Krasnitz A, Hicks J, ... Muthuswamy SK (2008). Brk is coamplified with ErbB2 to promote proliferation in breast cancer. Proc. Natl. Acad. Sci. U.S.A., 105, 12463-8.

Peng M, Ball-Kell SM & Tyner AL (2015). Protein tyrosine kinase 6 promotes ERBB2-induced mammary gland tumorigenesis in the mouse. Cell Death Dis, 6, e1848.

Kamalati T, Jolin HE, Mitchell PJ, Barker KT, Jackson LE, Dean CJ, ... Crompton MR (1996). Brk, a breast tumor-derived non-receptor protein-tyrosine kinase, sensitizes mammary epithelial cells to epidermal growth factor. J. Biol. Chem., 271, 30956-63.

Kamalati T, Jolin HE, Fry MJ & Crompton MR (2000). Expression of the BRK tyrosine kinase in mammary epithelial cells enhances the coupling of EGF signalling to PI 3-kinase and Akt, via erbB3 phosphorylation. Oncogene, 19, 5471-6.

Castro NE & Lange CA (2010). Breast tumor kinase and extracellular signal-regulated kinase 5 mediate Met receptor signaling to cell migration in breast cancer cells. Breast Cancer Res., 12, R60.

Edit history

Date	Action	Author
2015-12-04	Created	Orlic-Milacic M
2016-01-05	Edited	Orlic-Milacic M
2016-01-05	Authored	Orlic-Milacic M
2016-02-07	Reviewed	Pires IM
2018-08-24	Modified	Schmidt EE

Elements found in this pathway

Input	UniProt Id	Input	UniProt Id	Input	UniProt Id
AKT1	P31749	CCND1	P24385	CCNE1	P24864
CDK4	P11802	EGFR	P00533	ERBB2	P04626
HIF1A	Q16665	KRAS	P01116, P01111	NRAS	P01111
SOCS3	O14543	STAT3	P40763		

Input	Ensembl Id	Input	Ensembl Id	Input	Ensembl Id
SOCS3	ENSG00000184557				

20. Cellular responses to stress (R-HSA-2262752)

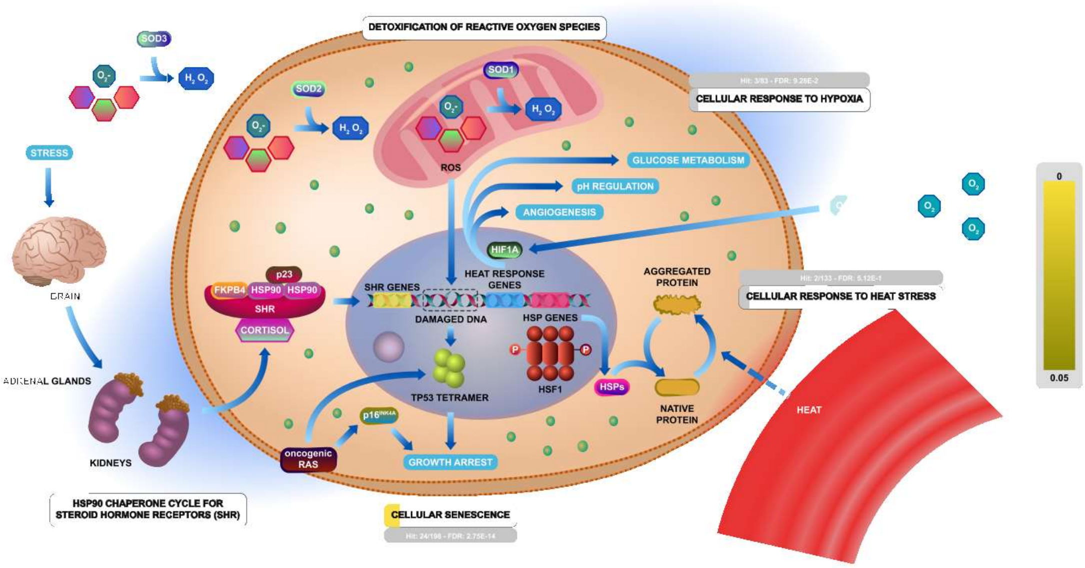

Cells are subject to external molecular and physical stresses such as foreign molecules that perturb metabolic or signaling processes, and changes in temperature or pH. The ability of cells and tissues to modulate molecular processes in response to such external stresses is essential to the maintenance of tissue homeostasis (Kultz 2005). Specific stress-related processes annotated here are **cellular response to hypoxia**, **detoxification of reactive oxygen species**, **cellular response to heat stress**, **cellular senescence**, and the **HSP90 chaperone cycle for steroid hormone receptors**.

References

Kültz D (2005). Molecular and evolutionary basis of the cellular stress response. Annu. Rev. Physiol. , 67, 225-57.

Edit history

Date	Action	Author
2012-05-20	Edited	Matthews L
2012-05-20	Reviewed	D'Eustachio P
2012-05-20	Authored	Matthews L
2012-05-20	Created	Matthews L
2018-08-24	Modified	Schmidt EE

Elements found in this pathway

Input	UniProt Id	Input	UniProt Id	Input	UniProt Id
BMI1	P35226	CCNE1	P24864	CCNE2	O96020
CDK4	P11802	CDK6	Q00534	CDKN1A	P38936
E2F1	O00716, Q01094	E2F2	Q14209	ETS1	P14921
EZH2	Q15910	FOS	P01100	GSK3B	P49841
HIF1A	Q16665	HMGA1	P17096	HMGA2	P52926
IL6	P05231	MAPK1	P28482	SP1	P08047
STAT3	P40763	SUZ12	Q15022	TP53	P04637

Input	UniProt Id
VEGFA	P15692

Input	Ensembl Id
CDKN1A	ENSG00000124762
EZH2	ENSG00000106462
IL6	ENSG00000136244
SUZ12	ENSG00000178691
VEGFA	ENSG00000112715

21. Mitotic G1-G1/S phases (R-HSA-453279)

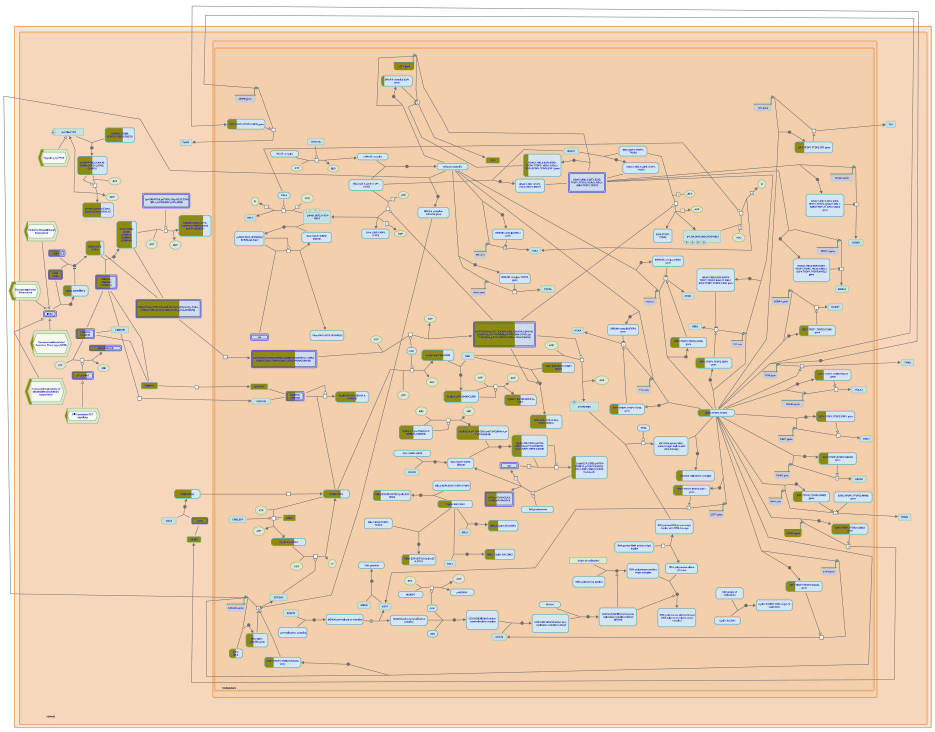

Mitotic G1-G1/S phase involves G1 phase of the mitotic interphase and G1/S transition, when a cell commits to DNA replication and divison genetic and cellular material to two daughter cells.

During early G1, cells can enter a quiescent G0 state. In quiescent cells, the evolutionarily conserved DREAM complex, consisting of the pocket protein family member p130 (RBL2), bound to E2F4 or E2F5, and the MuvB complex, represses transcription of cell cycle genes (reviewed by Sadasivam and DeCaprio 2013).

During early G1 phase in actively cycling cells, transcription of cell cycle genes is repressed by another pocket protein family member, p107 (RBL1), which forms a complex with E2F4 (Ferreira et al. 1998, Cobrinik 2005). RB1 tumor suppressor, the product of the retinoblastoma susceptibility gene, is the third member of the pocket protein family. RB1 binds to E2F transcription factors E2F1, E2F2 and E2F3 and inhibits their transcriptional activity, resulting in prevention of G1/S transition (Chellappan et al. 1991, Bagchi et al. 1991, Chittenden et al. 1991, Lees et al. 1993, Hiebert 1993, Wu et al. 2001). Once RB1 is phosphorylated on serine residue S795 by Cyclin D:CDK4/6 complexes, it can no longer associate with and inhibit E2F1-3. Thus, CDK4/6-mediated phosphorylation of RB1 leads to transcriptional activation of E2F1-3 target genes needed for the S phase of the cell cycle (Connell-Crowley et al. 1997). CDK2, in complex with cyclin E, contributes to RB1 inactivation and also activates proteins needed for the initiation of DNA replication (Zhang 2007). Expression of D type cyclins is regulated by extracellular mitogens (Cheng et al. 1998, Depoortere et al. 1998). Catalytic activities of CDK4/6 and CDK2 are controlled by CDK inhibitors of the INK4 family (Serrano et al. 1993, Hannon and Beach 1994, Guan et al. 1994, Guan et al. 1996, Parry et al. 1995) and the Cip/Kip family, respectively.

References

Cobrinik D (2005). Pocket proteins and cell cycle control. Oncogene, 24, 2796-809.

Sadasivam S & DeCaprio JA (2013). The DREAM complex: master coordinator of cell cycle-dependent gene expression. Nat. Rev. Cancer, 13, 585-95.

Ferreira R, Magnaghi-Jaulin L, Robin P, Harel-Bellan A & Trouche D (1998). The three members of the pocket proteins family share the ability to repress E2F activity through recruitment of a histone deacetylase. Proc Natl Acad Sci U S A, 95, 10493-8.

Connell-Crowley L, Harper JW & Goodrich DW (1997). Cyclin D1/Cdk4 regulates retinoblastoma protein-mediated cell cycle arrest by site-specific phosphorylation. Mol Biol Cell, 8, 287-301.

Chellappan SP, Hiebert S, Mudryj M, Horowitz JM & Nevins JR (1991). The E2F transcription factor is a cellular target for the RB protein. Cell, 65, 1053-61.

Edit history

Date	Action	Author
2010-01-19	Edited	Matthews L
2010-01-20	Authored	Matthews L
2010-01-20	Created	Matthews L
2011-06-16	Reviewed	Grana X
2011-08-25	Reviewed	MacPherson D
2011-08-26	Revised	Orlic-Milacic M
2011-08-26	Authored	Orlic-Milacic M
2017-02-08	Edited	Orlic-Milacic M
2018-07-10	Reviewed	Manfredi JJ
2018-08-24	Modified	Schmidt EE

Elements found in this pathway

Input	UniProt Id	Input	UniProt Id	Input	UniProt Id
AKT1	P31749	AKT2	P31751	CCND1	P24385
CCND2	P30279	CCNE1	P24864	CCNE2	O96020
CDK4	P11802	CDK6	Q00534	CDKN1A	P38936
CDKN1C	P49918	E2F1	O00716, Q01094	E2F2	Q14209
MYC	P01106	WEE1	P30291		

Input	Ensembl Id	Input	Ensembl Id	Input	Ensembl Id
CCNE1	ENSG00000105173	E2F1	ENSG00000101412		

22. Negative regulation of the PI3K/AKT network (R-HSA-199418)

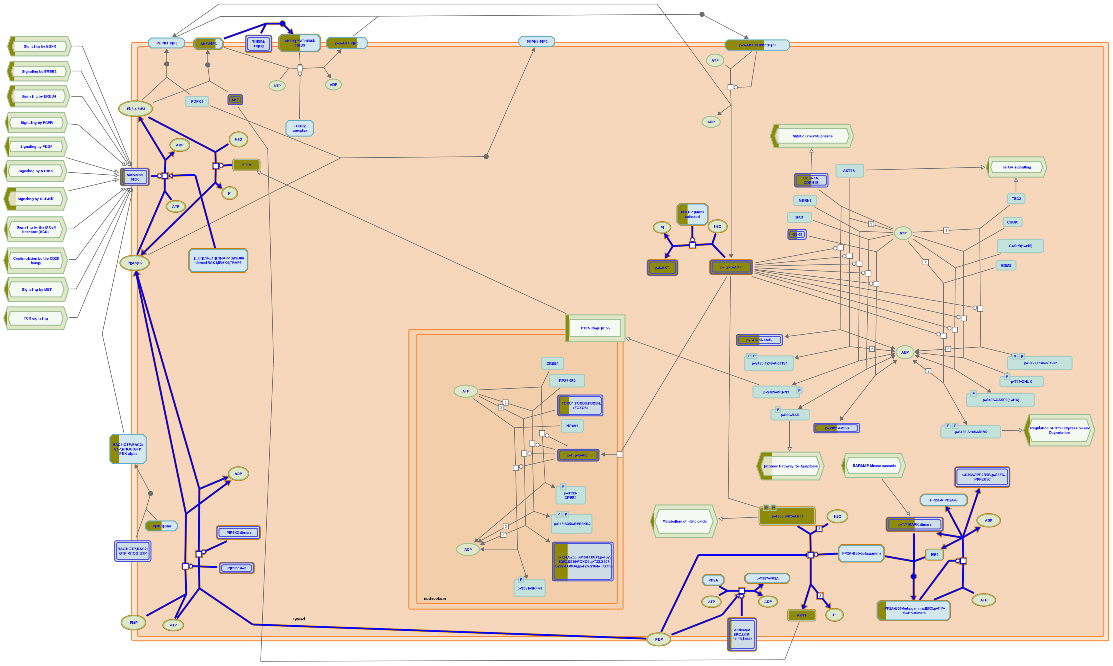

The PI3K/AKT network is negatively regulated by phosphatases that dephosphorylate PIP3, thus hampering AKT activation.

Edit history

Date	Action	Author
2006-10-10	Authored	Nasi S, Annibali D
2007-07-10	Created	Jassal B
2007-11-08	Reviewed	Greene LA
2012-06-21	Revised	Orlic-Milacic M
2012-08-13	Reviewed	Yuzugullu H, Thorpe L, Zhao JJ
2018-08-30	Modified	Croft D

Elements found in this pathway

Input	UniProt Id	Input	UniProt Id	Input	UniProt Id
AKT1	Q9Y243, P31749	AKT2	P31751	EGFR	P00533
ERBB2	P04626	ESR1	P03372-3, P03372-4, P03372	KIT	P10721
MAPK1	P28482	MET	P08581	PHLPP1	O60346
PIK3CA	P42336	PIK3R1	P27986	PTEN	P60484

23. TP53 Regulates Transcription of Genes Involved in G1 Cell Cycle Arrest (R-HSA-6804116)

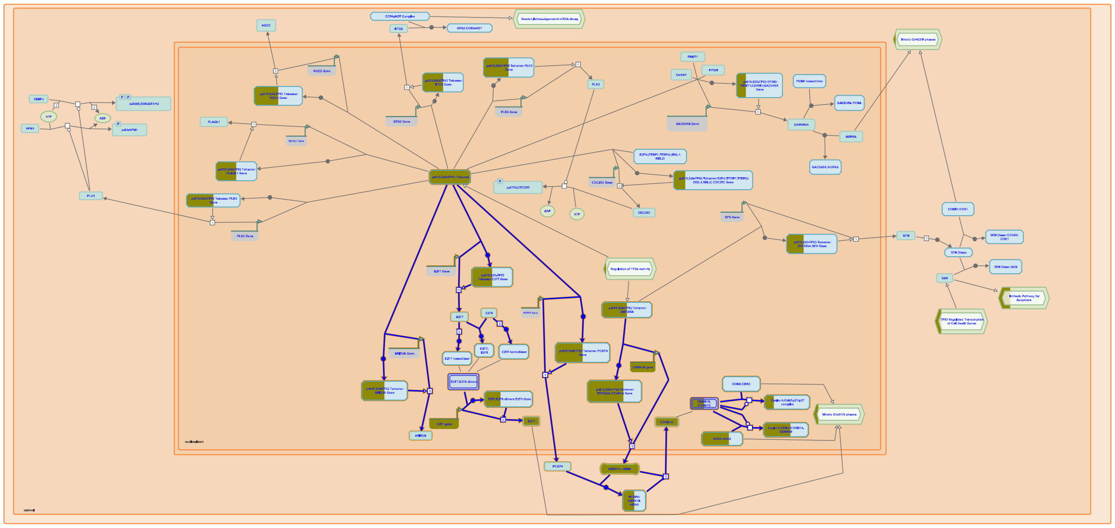

The most prominent TP53 target involved in G1 arrest is the inhibitor of cyclin-dependent kinases CDKN1A (p21). CDKN1A is one of the earliest genes induced by TP53 (El-Deiry et al. 1993). CDKN1A binds and inactivates CDK2 in complex with cyclin A (CCNA) or E (CCNE), thus preventing G1/S transition (Harper et al. 1993). Considering its impact on the cell cycle outcome, CDKN1A expression levels are tightly regulated. For instance, under prolonged stress, TP53 can induce the transcription of an RNA binding protein PCBP4, which can bind and destabilize CDKN1A mRNA, thus alleviating G1 arrest and directing the affected cell towards G2 arrest and, possibly, apoptosis (Zhu and Chen 2000, Scoumanne et al. 2011). Expression of E2F7 is directly induced by TP53. E2F7 contributes to G1 cell cycle arrest by repressing transcription of E2F1, a transcription factor that promotes expression of many genes needed for G1/S transition (Aksoy et al. 2012, Carvajal et al. 2012). ARID3A is a direct transcriptional target of TP53 (Ma et al. 2003) that may promote G1 arrest by cooperating with TP53 in induction of CDKN1A transcription (Lestari et al. 2012). However, ARID3A may also promote G1/S transition by stimulating transcriptional activity of E2F1 (Suzuki et al. 1998, Peeper et al. 2002).

TP53 has co-factors that are key determinants of transcriptional selectivity within the p53 network. For instance, the zinc finger transcription factor ZNF385A (HZF) is a direct transcriptional target of TP53 that can form a complex with TP53 and facilitate TP53-mediated induction of CDKN1A, strongly favouring cell cycle arrest over apoptosis (Das et al. 2007).

References

el-Deiry WS, Tokino T, Velculescu VE, Levy DB, Parsons R, Trent JM, ... Vogelstein B (1993). WAF1, a potential mediator of p53 tumor suppression. Cell, 75, 817-25.

Harper JW, Adami GR, Wei N, Keyomarsi K & Elledge SJ (1993). The p21 Cdk-interacting protein Cip1 is a potent inhibitor of G1 cyclin-dependent kinases. Cell, 75, 805-16.

Zhu J & Chen X (2000). MCG10, a novel p53 target gene that encodes a KH domain RNA-binding protein, is capable of inducing apoptosis and cell cycle arrest in G(2)-M. Mol. Cell. Biol., 20, 5602-18.

Scoumanne A, Cho SJ, Zhang J & Chen X (2011). The cyclin-dependent kinase inhibitor p21 is regulated by RNA-binding protein PCBP4 via mRNA stability. Nucleic Acids Res., 39, 213-24.

Aksoy O, Chicas A, Zeng T, Zhao Z, McCurrach M, Wang X & Lowe SW (2012). The atypical E2F family member E2F7 couples the p53 and RB pathways during cellular senescence. Genes Dev., 26, 1546-57.

Edit history

Date	Action	Author
2015-10-08	Created	Orlic-Milacic M
2015-10-14	Edited	Orlic-Milacic M
2015-10-14	Authored	Orlic-Milacic M
2016-02-04	Reviewed	Zaccara S, Inga A
2017-01-03	Revised	Orlic-Milacic M
2018-08-30	Modified	Croft D

Elements found in this pathway

Input	UniProt Id	Input	UniProt Id	Input	UniProt Id
CCNE1	P24864	CCNE2	O96020	CDKN1A	P38936
E2F1	Q01094	TP53	P04637		

Input	Ensembl Id	Input	Ensembl Id	Input	Ensembl Id
CDKN1A	ENST00000244741, ENSG00000124762	E2F1	ENSG00000101412		

24. Signaling by Receptor Tyrosine Kinases (R-HSA-9006934)

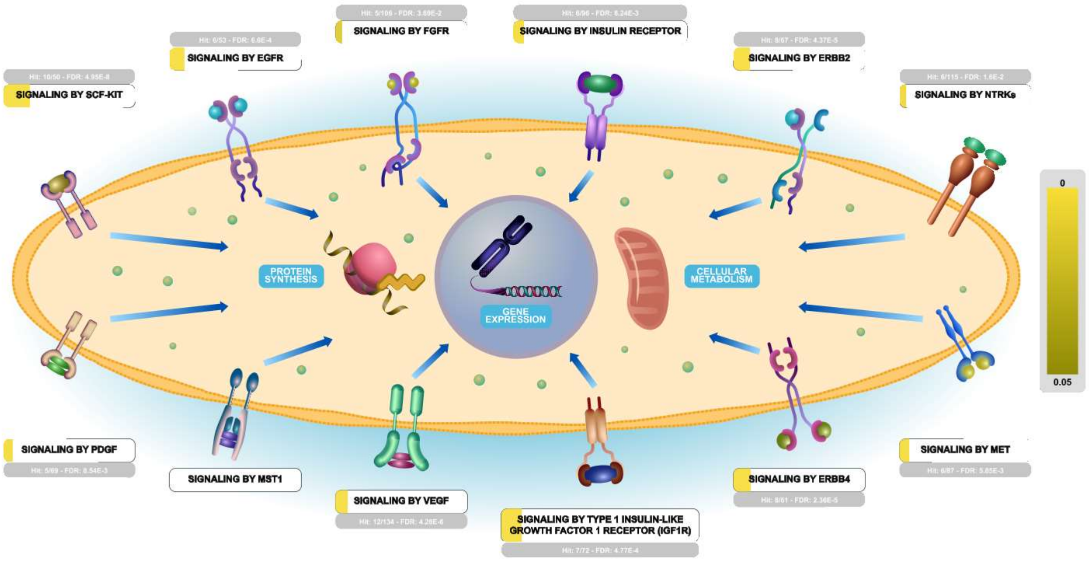

Receptor tyrosine kinases (RTKs) are a major class of cell surface proteins involved in Signal Transduction. Human cells contain ~60 RTKs, grouped into 20 subfamilies based on their domain architecture. All RTK subfamilies are characterized by an extracellular ligand-binding domain, a single transmembrane region and an intracellular region consisting of the tyrosine kinase domain and additional regulatory and protein interaction domains. In general, RTKs associate into dimers upon ligand binding and are activated by autophosphorylation on conserved intracellular tyrosine residues. Autophosphorylation increases the catalytic efficiency of the receptor and provides binding sites for the assembly of downstream signaling complexes (reveiwed in Lemmon and Schlessinger, 2010). Common signaling pathways activated downstream of RTK activation include RAF/MAP kinase cascades (reviewed in McKay and Morrison, 2007 and Wellbrock et al 2004), AKT signaling (reviewed in Manning and Cantley, 2007) and PLC-gamma mediated signaling (reviewed in Patterson et al). Activation of these pathways ultimately results in changes in gene expression and cellular metabolism.

References

Lemmon MA & Schlessinger J (2010). Cell signaling by receptor tyrosine kinases. Cell, 141, 1117-34.

McKay MM & Morrison DK (2007). Integrating signals from RTKs to ERK/MAPK. Oncogene, 26, 3113-21.

Wellbrock C, Karasarides M & Marais R (2004). The RAF proteins take centre stage. Nat Rev Mol Cell Biol, 5, 875-85.

Manning BD & Cantley LC (2007). AKT/PKB signaling: navigating downstream. Cell, 129, 1261-74.

Patterson RL, van Rossum DB, Nikolaidis N, Gill DL & Snyder SH (2005). Phospholipase C-gamma: diverse roles in receptor-mediated calcium signaling. Trends Biochem Sci, 30, 688-97.

Edit history

Date	Action	Author
2017-05-24	Edited	Rothfels K

Date	Action	Author
2017-05-24	Authored	Rothfels K
2017-05-24	Created	Rothfels K
2017-06-22	Reviewed	D'Eustachio P
2018-08-24	Modified	Schmidt EE

Elements found in this pathway

Input	UniProt Id	Input	UniProt Id	Input	UniProt Id
AKT1	Q9Y243, P31749	AKT2	P31751	CDC42	P60953
CHEK1	O14757	CXCL12	P48061	EGFR	P00533
ERBB2	P04626	ESR1	P03372	IGF1	P05019
IGF1R	P08069	ITGB3	P05106	KIT	P10721
KRAS	P01116, P01111	MAPK1	P28482	MET	P08581
MMP9	P14780	NRAS	P01111	PAK1	Q13153
PIK3CA	P42336	PIK3R1	P27986	SOCS1	O15524
SOCS6	O14544	STAT3	P40763	VEGFA	P15692-4, P15692

Input	Ensembl Id	Input	Ensembl Id	Input	Ensembl Id
CXCL12	ENSG00000107562				

25. Cellular responses to external stimuli (R-HSA-8953897)

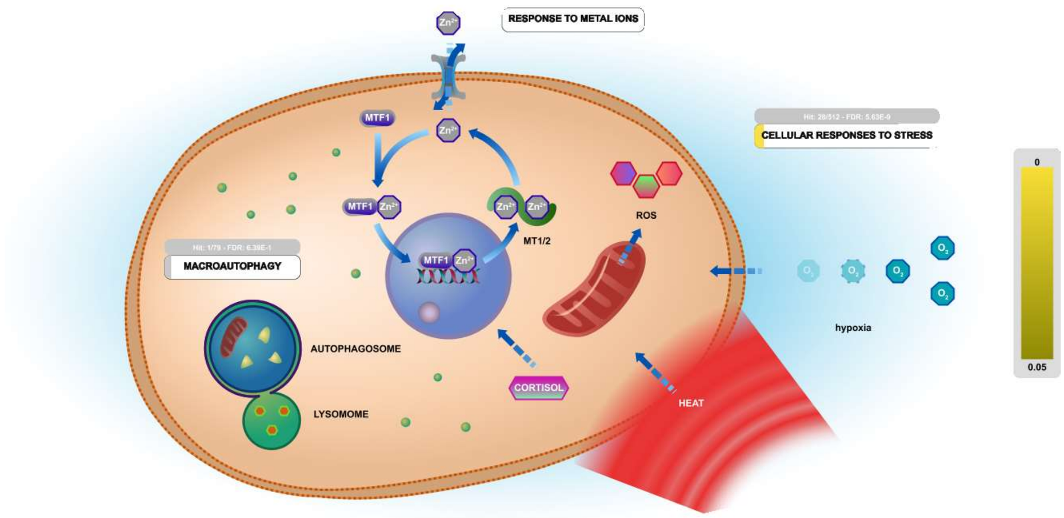

Individual cells detect and respond to diverse external molecular and physical signals. Appropriate responses to these signals are essential for normal development, maintenance of homeostasis in mature tissues, and effective defensive responses to potentially noxious agents (Kultz 2005). It is convenient, if somewhat arbitrary, to distinguish responses to signals involved in development and homeostasis from ones involved in stress responses, and that classification is followed here, with **macroautophagy** and **responses to metal ions** classified as responses to normal external stimuli, while responses to hypoxia, reactive oxygen species, and heat, and the process of cellular senescence are classified as **stress responses**. Signaling cascades are integral components of all of these response mechanisms but because of their number and diversity, they are grouped in a separate signal transduction superpathway in Reactome.

References

Kültz D (2005). Molecular and evolutionary basis of the cellular stress response. Annu. Rev. Physiol. , 67, 225-57.

Edit history

Date	Action	Author
2012-05-20	Reviewed	D'Eustachio P
2015-05-13	Reviewed	Tooze SA
2015-09-03	Reviewed	Klionsky DJ
2015-09-19	Reviewed	Atrian S
2016-12-30	Edited	D'Eustachio P
2016-12-30	Authored	D'Eustachio P
2016-12-30	Created	D'Eustachio P
2018-08-24	Modified	Schmidt EE

Elements found in this pathway

Input	UniProt Id	Input	UniProt Id	Input	UniProt Id
BECN1	Q14457	BMI1	P35226	CCNE1	P24864
CCNE2	O96020	CDK4	P11802	CDK6	Q00534
CDKN1A	P38936	E2F1	O00716, Q01094	E2F2	Q14209
ETS1	P14921	EZH2	Q15910	FOS	P01100
GSK3B	P49841	HIF1A	Q16665	HMGA1	P17096
HMGA2	P52926	IL6	P05231	MAPK1	P28482
SP1	P08047	STAT3	P40763	SUZ12	Q15022
TP53	P04637	VEGFA	P15692		

Input	Ensembl Id	Input	Ensembl Id	Input	Ensembl Id
CDKN1A	ENSG00000124762	EZH2	ENSG00000106462	IL6	ENSG00000136244
SUZ12	ENSG00000178691	VEGFA	ENSG00000112715		

6. Identifiers found

Input	UniProt Id	Input	UniProt Id	Input	UniProt Id
AKT1	P31749	AKT2	P31751	APAF1	O14727
AURKB	Q96GD4	BCL2	P10415	BCL2L1	Q07817
BCL2L11	O43521	BCL6	P41182	BECN1	Q14457
BMI1	P35226	BMPR2	Q13873	CCND1	P24385
CCND2	P30279	CCNE1	P24864	CCNE2	O96020
CDC42	P60953	CDK4	P11802	CDK6	Q00534
CDKN1A	P38936	CDKN1C	P49918	CHEK1	O14757
CTGF	P29279	CXCL12	P48061	CXCR4	P61073
DNMT1	P26358	E2F1	Q01094	E2F2	Q14209
EGFR	P00533	ERBB2	P04626	ESR1	P03372
ETS1	P14921	EZH2	Q15910	FAS	P25445
FOS	P01100	FOXO1	Q12778	FSCN1	Q16658
GSK3B	P49841	HIF1A	Q16665	HMGA1	P17096
HMGA2	P52926	HMGB1	P09429	ICAM1	P05362
IGF1	P05019	IGF1R	P08069	IL6	P05231
ITGB3	P05106	JAG1	P78504	KIT	P10721
KRAS	P01116	MAPK1	P28482	MCL1	Q07820
MET	P08581	MMP2	P08253	MMP9	P14780
MYC	P01106	NOTCH1	P46531	NRAS	P01111
PAK1	Q16512, Q13153	PDCD4	Q53EL6	PHLPP1	O60346
PIK3CA	P42336	PIK3R1	P27986	PPARA	Q07869
PTEN	P60484	PTGS2	P35354	RARB	P10826
RHOB	P62745	RUNX2	Q13950-2, Q13950-1	SERPINE1	P05121
SFRP1	Q8N474	SMAD1	Q15797	SMAD2	Q15796
SMAD4	Q13485	SMAD7	O15105	SOCS1	O15524
SOCS3	O14543	SOCS6	O14544	SOX2	P48431
SP1	P08047	STAT3	P40763	SUZ12	Q15022
TGFBR2	P37173	TNFAIP3	P21580	TNFRSF10B	O14763, Q9UBN6
TP53	P04637	VEGFA	P15692	WEE1	P30291
WNT1	P04628	YWHAZ	P63104	ZEB1	P37275

Input	Ensembl Id	Input	Ensembl Id	Input	Ensembl Id
AKT1	ENSG00000142208	APAF1	ENSG00000120868	BCL2	ENSG00000171791
BCL2L1	ENSG00000171552	BCL2L11	ENSG00000153094	BCL6	ENSG00000113916
CCND1	ENSG00000110092	CCNE1	ENSG00000105173	CDC42	ENSG00000070831
CDKN1A	ENST00000244741, ENSG00000124762	CHEK1	ENST00000438015, ENSG00000149554	CTGF	ENSG00000118523
CXCL12	ENSG00000107562	E2F1	ENSG00000101412	EGFR	ENSG00000146648
ERBB2	ENSG00000141736	ESR1	ENSG00000091831	EZH2	ENSG00000106462
FAS	ENSG00000026103	FOS	ENSG00000170345	FOXO1	ENSG00000150907
FSCN1	ENSG00000075618	HIF1A	ENSG00000100644	ICAM1	ENSG00000090339
IL6	ENSG00000136244	JAG1	ENSG00000101384	KIT	ENSG00000157404
MCL1	ENSG00000143384	MET	ENSG00000105976	MMP2	ENSG00000087245
MMP9	ENSG00000100985	MYC	ENSG00000136997	NOTCH1	ENST00000277541, ENSG00000148400
PDCD4	ENSG00000150593	PIK3R1	ENSG00000145675	PTEN	ENST00000371953, ENSG00000171862

Input	Ensembl Id	Input	Ensembl Id	Input	Ensembl Id
PTGS2	ENSG00000073756	RUNX2	ENSG00000124813	SERPINE1	ENSG00000106366
SMAD7	ENSG00000101665	SOCS1	ENSG00000185338	SOCS3	ENSG00000184557
SOX2	ENSG00000181449	SUZ12	ENSG00000178691	TNFRSF10B	ENSG00000120889
TP53	ENSG00000141510	VEGFA	ENSG00000112715	ZEB1	ENSG00000148516

7. Identifiers not found

HOXA10	MTDH	TCEAL1	TGFBR3
