# Supplementary material for: Panoramic Visualization of Circulating MicroRNAs Across Neurodegenerative Diseases in Humans
Source: Mol Neurobiol. 2019 Apr 29;56(11):7380–407. doi: 10.1007/s12035-019-1615-1 (PMC6815273; doi:10.1007/s12035-019-1615-1)
Supplement: Supplementary file 5 — (PDF 310 kb) [file 12035_2019_1615_MOESM5_ESM.pdf]

miRNA	Condition	Expression	Analysis Method	Source	C'somal location	C'somal location -2	Ref
**brain-miR-112**	AD	Up	NGS & qPCR	Blood			(1)
**brain-miR-161**	AD	Up	NGS & qPCR	Blood			(1)
**hsa-let-7a**	MS	Down	qPCR	Plasma	NC_000009.12 (94175957..94176036)		(2)
**hsa-let-7a**	PD	Down	miRGenes qPCR panel	Plasma	NC_000009.12 (94175957..94176036)		(3)
**hsa-let-7a-5p**	fALS	Up	Microarray Meta-analysis	Serum	NC_000009.12 (94175957..94176036)		(4)
**hsa-let-7a-5p**	sALS	Up	Microarray Meta-analysis	Serum	NC_000009.12 (94175957..94176036)		(4)
**hsa-let-7b**	sALS	Down	qPCR	CSF	NC_000022.11 (46113686..46113768)		(5)
**hsa-let-7c-5p**	MS	Down	Exiqon qPCR panel & qPCR	Serum	NC_000021.9 (16539828..16539911)		(6)
**hsa-let-7d-3p**	fALS	Down	Exiqon qPCR panel & qPCR	Serum	NC_000009.12 (94178834..94178920)		(7)
**hsa-let-7d-3p**	MS	Up	Exiqon qPCR panel & qPCR	Blood	NC_000009.12 (94178834..94178920)		(1)
**hsa-let-7d-3p**	sALS	Down	Exiqon qPCR panel & qPCR	Serum	NC_000009.12 (94178834..94178920)		(7)
**hsa-let-7d-5p**	AD	Down	nCounter Nanostring & qPCR	Plasma	NC_000009.12 (94178834..94178920)		(8)
**hsa-let-7d-5p**	AD	Down	qPCR	Plasma	NC_000009.12 (94178834..94178920)		(9)
**hsa-let-7d-5p**	AD	Down	NGS & qPCR	Serum	NC_000009.12 (94178834..94178920)		(10)
**hsa-let-7d-5p**	fALS	Up	Microarray meta-analysis	Serum	NC_000009.12 (94178834..94178920)		(4)
**hsa-let-7d-5p**	sALS	Up	Microarray meta-analysis	Serum	NC_000009.12 (94178834..94178920)		(4)
**hsa-let-7f**	PD	Down	miRGenes qPCR panel	Plasma	NC_000009.12 (94176347..94176433)	NC_000023.11 (53557192..53557274, complement)	(3)

**hsa-let-7f-5p**	AD	Down	NGS & qPCR	Blood	NC_000009.12 (94176347..94176433)	NC_000023.11 (53557192..53557274, complement)	(1)
**hsa-let-7f-5p**	PD	Up	NGS	CSF	NC_000009.12 (94176347..94176433)	NC_000023.11 (53557192..53557274, complement)	(11)
**hsa-let-7g**	AD	Down	Microarray & qPCR	Serum	NC_000003.12 (52268278..52268361, complement)		(12)
**hsa-let-7g-3p**	PD	Up	NGS	CSF	NC_000003.12 (52268278..52268361, complement)		(13)
**hsa-let-7g-3p**	PD	Up	TLDA	CSF exosomes	NC_000003.12 (52268278..52268361, complement)		(14)
**hsa-let-7g-5p**	AD	Down	nCounter Nanostring & qPCR	Plasma	NC_000003.12 (52268278..52268361, complement)		(8)
**hsa-let-7g-5p**	AD	Down	NGS & qPCR	Plasma	NC_000003.12 (52268278..52268361, complement)		(10)
**hsa-let-7g-5p**	AD	Down	qPCR	Plasma	NC_000003.12 (52268278..52268361, complement)		(9)
**hsa-let-7i-5p**	AD	Up	qPCR	CSF	NC_000012.12 (62603686..62603769)		(15)
**hsa-let-7i-5p**	fALS	Up	Microarray meta-analysis	Serum	NC_000012.12 (62603686..62603769)		(4)
**hsa-let-7i-5p**	sALS	Up	Microarray meta-analysis	Serum	NC_000012.12 (62603686..62603769)		(4)
**hsa-miR-1**	fALS	Up	Exiqon qPCR panel & qPCR	Serum	NC_000020.11 (62554306..62554376)	NC_000018.10 (21829004..21829088, complement)	(7)
**hsa-miR-1**	PD	Down	TLDA	CSF exosomes	NC_000020.11 (62554306..62554376)	NC_000018.10 (21829004..21829088, complement)	(14)
**hsa-miR-1**	sALS	Up	Exiqon qPCR panel & qPCR	Serum	NC_000020.11 (62554306..62554376)	NC_000018.10 (21829004..21829088, complement)	(7)

**hsa-miR-100**	AD	Up	OpenArray qPCR	CSF	NC_000011.10 (122152229..122152308, complement)		(16)
**hsa-miR-101-3p**	AD	Up	NGS & qPCR	Serum exosomes	NC_000001.11 (65058434..65058508, complement)		(17)
**hsa-miR-101-5p**	AD	Down	NGS	CSF	NC_000001.11 (65058434..65058508, complement)		(13)
**hsa-miR-103a-3p**	AD	Down	NGS & qPCR	Blood	NC_000005.10 (168560896..168560973, complement)		(1)
**hsa-miR-103a-3p**	AD	Down	Openarray qPCR	CSF	NC_000005.10 (168560896..168560973, complement)		(16)
**hsa-miR-103a-3p**	AD	Down	Exiqon qPCR panel	Plasma	NC_000005.10 (168560896..168560973, complement)		(18)
**hsa-miR-103a-3p**	PD	Up	qPCR	PBMCs	NC_000005.10 (168560896..168560973, complement)		(19)
**hsa-miR-106a-5p**	AD	Up	NGS & qPCR	Serum exosomes	NC_000023.11 (134170198..134170278, complement)		(17)
**hsa-miR-106a-5p**	fALS	Up	Microarray meta-analysis	Serum	NC_000023.11 (134170198..134170278, complement)		(4)
**hsa-miR-106a-5p**	sALS	Up	Microarray meta-analysis	Serum	NC_000023.11 (134170198..134170278, complement)		(4)
**hsa-miR-106b-3p**	AD	Up	NGS & qPCR	Serum	NC_000007.14 (100093993..100094074, complement)		(20)
**hsa-miR-106b-5p**	AD	Up	NGS & qPCR	Serum exosomes	NC_000023.11 (134170198..134170278, complement)		(17)
**hsa-miR-107**	AD	Down	NGS & qPCR	Blood	NC_000010.11 (89592747..89592827, complement)		(1)
**hsa-miR-10a-5p**	AD	Down	NGS	CSF	NC_000005.10 (160485352..160485450)		(13)

**hsa-miR-10a-5p**	PD	Down	NGS	CSF	NC_000005.10 (160485352..160485450)		(13)
**hsa-miR-10a-5p**	PD	Up	TLDA	CSF exosomes	NC_000005.10 (160485352..160485450)		(14)
**hsa-miR-10b-5p**	PD	Up	NGS	CSF	NC_000002.12 (176150303..176150412)		(11)
**hsa-miR-1224-5p**	PD	Down	NGS	CSF	NC_000003.12 (184241405..184241489)		(13)
**hsa-miR-122-5p**	MS	Down	NGS & ddPCR	Serum exosomes	NC_000018.10 (58451074..58451158)		(21)
**hsa-miR-1234-3p**	sALS	Down	Microarray & qPCR(a, b)	Leukocytes	NC_000008.11 (144400086..144400165, complement)		(22)
**hsa-miR-1234-3p**	sALS	Down	Microarray & qPCR(a, b)	Serum	NC_000008.11 (144400086..144400165, complement)		(23)
**hsa-miR-124**	PD	Down	qPCR	Plasma	NC_000008.11 (9903388..9903472, complement)		(24)
**hsa-miR-124-3p**	AD	Down	NGS	CSF	NC_000008.11 (9903388..9903472, complement)		(13)
**hsa-miR-1246**	AD	Up	NGS & qPCR	Serum	NC_000002.12 (176600980..176601052, complement)		(20)
**hsa-miR-125a**	MS	Up	Microarray & qPCR	PBMCs	NC_000019.10 (51693254..51693339)		(25)
**hsa-miR-125a-3p**	AD	Up	NGS	Serum	NC_000019.10 (51693254..51693339)		(13)
**hsa-miR-125a-5p**	PD	Down	NGS	CSF	NC_000019.10 (51693254..51693339)		(11)
**hsa-miR-125b**	AD	Up	TLDA	CSF	NC_000011.10 (122099757..122099844, complement)		(26)
**hsa-miR-125b**	AD	Down	qPCR	CSF	NC_000011.10 (122099757..122099844, complement)		(27)

**hsa-miR-125b**	AD	Down	Microarray & qPCR	Serum	NC_000011.10 (122099757..122099844, complement)		(28)
**hsa-miR-125b**	MS	Up	Microarray	PBMCs	NC_000011.10 (122099757..122099844, complement)		(29)
**hsa-miR-125b-1-3p**	AD	Up	NGS	Serum	NC_000011.10 (122099757..122099844, complement)		(13)
**hsa-miR-125b-5p**	AD	Up	Exiqon panel & qPCR	CSF	NC_000011.10 (122099757..122099844, complement)		(30)
**hsa-miR-125b-5p**	AD	Down	Exiqon panel & qPCR	CSF	NC_000011.10 (122099757..122099844, complement)		(31)
**hsa-miR-126**	AD	Down	Microarray & qPCR	Serum	NC_000009.12 (136670602..136670686)		(12)
**hsa-miR-1260a**	AD	Up	Exiqon qPCR panel	Plasma	NC_000014.9 (77266218..77266290)		(18)
**hsa-miR-126-5p**	AD	Down	NGS & qPCR	Serum	NC_000009.12 (136670602..136670686)		(20)
**hsa-miR-127-3p**	AD	Up	NGS	Serum	NC_000014.9 (100882979..100883075)		(13)
**hsa-miR-127-3p**	MS	Down	NGS	Serum exosomes	NC_000014.9 (100882979..100883075)		(32)
**hsa-miR-127-3p**	PD	Down	NGS	CSF	NC_000014.9 (100882979..100883075)		(13)
**hsa-miR-1274a**	AD	Down	Openarray qPCR	CSF	NC_000005.9 (41475734..41475804)		(16)
**hsa-miR-1274b**	PD	Up	NGS	PBMCs	NC_000019.9 (58024375..58024441, complement)		(33)
**hsa-miR-1275**	sALS	Down	Microarray & qPCR	Leukocytes	NC_000006.12 (33999972..34000051, complement)		(22)
**hsa-miR-128**	PD	Down	NGS	CSF	NC_000002.12 (135665397..135665478)		(13)
**hsa-miR-1281**	fALS	Down	Microarray Meta-analysis	Serum	NC_000022.11 (41092513..41092566)		(4)

**hsa-miR-1281**	sALS	Down	Microarray Meta-analysis	Serum	NC_000022.11 (41092513..41092566)		(4)
**hsa-miR-128-3p**	MS	Up	qPCR	Serum	NC_000002.12 (135665397..135665478)	NC_000003.12 (35744476..35744559)	(34)
**hsa-miR-1285-3p**	AD	Up	NGS	Serum	NC_000007.14 (92204015..92204098, complement)	NC_000002.12 (70252918..70253005, complement)	(13)
**hsa-miR-1285-5p**	AD	Down	NGS & qPCR	Blood	NC_000007.14 (92204015..92204098, complement)	NC_000002.12 (70252918..70253005, complement)	(1)
**hsa-miR-1291**	AD	Up	TLDA	CSF	NC_000012.12 (48654444..48654530, complement)		(31)
**hsa-miR-1294**	PD	Down	NGS	Serum	NC_000005.10 (154347106..154347247)		(13)
**hsa-miR-129-5p**	AD	Down	NGS	CSF	NC_000007.14 (128207872..128207943)		(13)
**hsa-miR-1306-5p**	AD	Down	NGS & qPCR	Serum exosomes	NC_000022.11 (20086058..20086142)		(17)
**hsa-miR-1307-5p**	AD	Up	NGS	Serum	NC_000010.11 (103394253..103394401, complement)		(13)
**hsa-miR-130a-3p**	AD	Up	Microarray & qPCR	PBMCs	NC_000011.10 (57641198..57641286)		(35)
**hsa-miR-130b-3p**	fALS	Up	Microarray Meta-analysis	Serum	NC_000022.11 (21653304..21653385)		(4)
**hsa-miR-130b-3p**	sALS	Up	Microarray Meta-analysis	Serum	NC_000022.11 (21653304..21653385)		(4)
**hsa-miR-132-3p**	AD	Down	NGS	CSF	NC_000017.11 (2049908..2050008, complement)		(13)
**hsa-miR-132-3p**	sALS	Down	qPCR	CSF	NC_000017.11 (2049908..2050008, complement)		(5)
**hsa-miR-132-5p**	PD	Down	NGS	CSF	NC_000017.11 (2049908..2050008, complement)		(13)

**hsa-miR-132-5p**	PD	Up	TLDA	CSF exosomes	NC_000017.11 (2049908..2050008, complement)		(14)
**hsa-miR-132-5p**	sALS	Down	qPCR	CSF	NC_000017.11 (2049908..2050008, complement)		(5)
**hsa-miR-133a-3p**	fALS	Up	Exiqon qPCR panel & qPCR	Serum	NC_000018.10 (21825698..21825785, complement)	NC_000020.11 (62564912..62565013)	(7)
**hsa-miR-133a-3p**	sALS	Up	Exiqon qPCR panel & qPCR	Serum	NC_000018.10 (21825698..21825785, complement)	NC_000020.11 (62564912..62565013)	(7)
**hsa-miR-133b**	fALS	Up	Exiqon qPCR panel & qPCR	Serum	NC_000006.12 (52148923..52149041)		(7)
**hsa-miR-133b**	PD	Down	qPCR	Plasma	NC_000006.12 (52148923..52149041)		(36)
**hsa-miR-133b**	sALS	Up	qPCR	Plasma	NC_000006.12 (52148923..52149041)		(7)
**hsa-miR-134**	AD	Down	NGS	CSF	NC_000014.9 (101054687..101054759)		(13)
**hsa-miR-135a**	AD	Up	qPCR	Serum	NC_000003.12 (52294219..52294308, complement)		(37)
**hsa-miR-135a-5p**	AD	Up	NGS	Serum	NC_000003.12 (52294219..52294308, complement)		(13)
**hsa-miR-136-3p**	AD	Down	NGS	CSF	NC_000014.9 (100884702..100884783)		(13)
**hsa-miR-136-3p**	AD	Down	TLDA	CSF exosomes	NC_000014.9 (100884702..100884783)		(14)
**hsa-miR-136-3p**	fALS	Down	Exiqon qPCR panel & qPCR	Serum	NC_000014.9 (100884702..100884783)		(7)
**hsa-miR-136-3p**	PD	Down	NGS	CSF	NC_000014.9 (100884702..100884783)		(13)
**hsa-miR-136-3p**	sALS	Down	Exiqon qPCR panel & qPCR	Serum	NC_000014.9 (100884702..100884783)		(7)
**hsa-miR-136-5p**	AD	Down	NGS	CSF	NC_000014.9 (100884702..100884783)		(13)

**hsa-miR-137**	AD	Down	qPCR	Serum	NC_000001.11 (98046070..98046171, complement)		(38)
**hsa-miR-137**	MS	Up	qPCR	CSF	NC_000001.11 (98046070..98046171, complement)		(39)
**hsa-miR-137**	PD	Up	Up(a)	Plasma	NC_000001.11 (98046070..98046171, complement)		(24)
**hsa-miR-138-5p**	AD	Down	NGS	CSF	NC_000007.14 (148807372..148884349, complement)		(13)
**hsa-miR-139-5p**	AD	Down	NGS	CSF	NC_000011.10 (72615063..72615130, complement)		(13)
**hsa-miR-139-5p**	fALS	Down	Exiqon qPCR panel & qPCR	Serum	NC_000011.10 (72615063..72615130, complement)		(7)
**hsa-miR-139-5p**	sALS	Down	Exiqon qPCR panel & qPCR	Serum	NC_000011.10 (72615063..72615130, complement)		(7)
**hsa-miR-1-3p**	PD	Up	Microarray & qPCR	Blood	NC_000020.11 (62554306..62554376)		(40)
**hsa-miR-140-3p**	MS	Up	Exiqon qPCR panel & qPCR	Serum	NC_000016.10 (69933081..69933180)		(6)
**hsa-miR-140-5p**	AD	Down	TLDA	CSF	NC_000016.10 (69933081..69933180)		(31)
**hsa-miR-141**	PD	Down	NGS & qPCR	Serum	NC_000012.12 (6964097..6964191)		(41)
**hsa-miR-141-3p**	AD	Down	NGS & qPCR	Plasma exosomes	NC_000012.12 (6964097..6964191)		(42)
**hsa-miR-142-3p**	AD	Down	TLDA	CSF	NC_000017.11 (58331232..58331318, complement)		(31)
**hsa-miR-142-3p**	AD	Down	Exiqon qPCR panel	CSF	NC_000017.11 (58331232..58331318, complement)		(9)
**hsa-miR-142-3p**	AD	Down	Exiqon qPCR panel	Plasma	NC_000017.11 (58331232..58331318, complement)		(18)

**hsa-miR-142-3p**	MS	Up	microarray & qPCR	PBMCs	NC_000017.11 (58331232..58331318, complement)		(43)
**hsa-miR-142-3p**	PD	Down	miRGenes qPCR panel	Plasma	NC_000017.11 (58331232..58331318, complement)		(3)
**hsa-miR-142-5p**	AD	Up	qPCR	Blood	NC_000017.11 (58331232..58331318, complement)		(15)
**hsa-miR-143-3p**	AD	Down	TLDA & qPCR	Serum	NC_000005.10 (149428918..149429023)		(31)
**hsa-miR-143-3p**	AD	Up	NGS & qPCR	Serum exosomes	NC_000005.10 (149428918..149429023)		(17)
**hsa-miR-143-3p**	fALS	Down	qPCR	CSF	NC_000005.10 (149428918..149429023)		(5)
**hsa-miR-143-3p**	sALS	Down	qPCR	CSF	NC_000005.10 (149428918..149429023)		(44)
**hsa-miR-143-3p**	sALS	Up	Microarray & qPCR	Serum	NC_000005.10 (149428918..149429023)		(45)
**hsa-miR-143-5p**	sALS	Up	qPCR	CSF	NC_000005.10 (149428918..149429023)		(5)
**hsa-miR-143-5p**	sALS	Down	Microarray & qPCR	Serum	NC_000005.10 (149428918..149429023)		(44)
**hsa-miR-144-5p**	AD	Up	NGS & qPCR	Serum	NC_000017.11 (28861533..28861618, complement)		(20)
**hsa-miR-144-5p**	PD	Up	qPCR	CSF	NC_000017.11 (28861533..28861618, complement)		(46)
**hsa-miR-144-5p**	sALS	Up	Exiqon qPCR panel & qPCR	Serum	NC_000017.11 (28861533..28861618, complement)		(7)
**hsa-miR-144-5p**	sALS	Up	Exiqon qPCR panel & qPCR	Serum	NC_000017.11 (28861533..28861618, complement)		(7)
**hsa-miR-145**	MS	Up	Fluorescent nano-biosensor	Plasma	NC_000005.10 (149430646..149430733)		(47)

**hsa-miR-145-3p**	AD	Down	TLDA	CSF	NC_000005.10 (149430646..149430733)		(31)
**hsa-miR-145-5p**	AD	Down	TLDA	CSF	NC_000005.10 (149430646..149430733)		(31)
**hsa-miR-1469**	fALS	Down	Microarray Meta-analysis	Serum	NC_000015.10 (96333261..96333307)		(4)
**hsa-miR-1469**	sALS	Down	Microarray Meta-analysis	Serum	NC_000015.10 (96333261..96333307)		(4)
**hsa-miR-146a**	AD	Up	OpenArray qPCR	CSF	NC_000005.10 (160485352..160485450)		(16)
**hsa-miR-146a**	AD	Down	qPCR	CSF	NC_000005.10 (160485352..160485450)		(27)
**hsa-miR-146a**	AD	Down	qPCR	Plasma	NC_000005.10 (160485352..160485450)		(27)
**hsa-miR-146a**	MS	Up	microarray & qPCR	PBMCs	NC_000005.10 (160485352..160485450)		(43)
**hsa-miR-146a-5p**	AD	Down	TLDA	CSF	NC_000005.10 (160485352..160485450)		(31)
**hsa-miR-146a-5p**	AD	Down	qPCR	Plasma	NC_000005.10 (160485352..160485450)		(9)
**hsa-miR-146a-5p**	PD	Down	qPCR	Serum	NC_000005.10 (160485352..160485450)		(48)
**hsa-miR-146b**	MS	Up	Microarray & qPCR	PBMCs	NC_000010.11 (102436512..102436584)		(25)
**hsa-miR-146b**	MS	Up	Microarray & qPCR	PBMCs	NC_000010.11 (102436512..102436584)		(43)
**hsa-miR-146b-5p**	PD	Down	NGS & qPCR	Serum	NC_000010.11 (102436512..102436584)		(41)
**hsa-miR-148b-3p**	PD	Down	Microarray	Serum	NC_000012.12 (54337216..54337314)		(49)

**hsa-miR-148b-5p**	AD	Down	NGS & qPCR	Serum	NC_000012.12 (54337216..54337314)		(20)
**hsa-miR-149**	sALS	Down	Microarray & qPCR	Leukocytes	NC_000002.12 (240456001..240456089)		(22)
**hsa-miR-150**	MS	Up	TLDA & qPCR	CSF	NC_000019.10 (49500785..49500868, complement)		(50)
**hsa-miR-150**	MS	Up	Microarray	PBMCs	NC_000019.10 (49500785..49500868, complement)		(29)
**hsa-miR-150-5p**	AD	Down	NGS & qPCR	Plasma exosomes	NC_000019.10 (49500785..49500868, complement)		(42)
**hsa-miR-150-5p**	PD	Up	NGS	PBMCs	NC_000019.10 (49500785..49500868, complement)		(33)
**hsa-miR-151a-3p**	PD	Up	NGS	CSF	NC_000008.11 (140732564..140732653, complement)		(11)
**hsa-miR-151a-5p**	AD	Down	Exiqon qPCR panel	Plasma	NC_000008.11 (140732564..140732653, complement)		(18)
**hsa-miR-151a-5p**	fALS	Up	Microarray meta-analysis	Serum	NC_000008.11 (140732564..140732653, complement)		(4)
**hsa-miR-151a-5p**	sALS	Up	Microarray meta-analysis	Serum	NC_000008.11 (140732564..140732653, complement)		(4)
**hsa-miR-152**	MS	Down	Microarray & qPCR	PBMCs	NC_000017.11 (48037161..48037247, complement)		(25)
**hsa-miR-153**	PD	Up	TLDA	CSF exosomes	NC_000002.12 (219294111..219294200, complement)		(14)
**hsa-miR-15-3p**	AD	Down	NGS & qPCR	Serum exosomes			(17)
**hsa-miR-155**	MS	Up	Microarray & qPCR	PBMCs	NC_000021.9 (25573980..25574044)	NC_000007.14 (157574336..157574422, complement)	(43)

**hsa-miR-15a**	MS	Up	Microarray & qPCR	PBMCs	NC_000013.11 (50049119..50049201, complement)		(43)
**hsa-miR-15a-5p**	AD	Up	qPCR	CSF	NC_000013.11 (50049119..50049201, complement)		(15)
**hsa-miR-15a-5p**	AD	Up	NGS & qPCR	Serum exosomes	NC_000013.11 (50049119..50049201, complement)		(17)
**hsa-miR-15b**	MS	Down	Microarray & qPCR	Plasma	NC_000003.12 (160404588..160404685)		(43)
**hsa-miR-15b**	MS	Down	qPCR	Serum	NC_000003.12 (160404588..160404685)		(51)
**hsa-miR-15b**	MS	Up	Microarray	T cells	NC_000003.12 (160404588..160404685)		(29)
**hsa-miR-15b**	PD	Down	Microarray & qPCR	Serum	NC_000003.12 (160404588..160404685)		(52)
**hsa-miR-15b-5p**	AD	Down	qPCR	Plasma	NC_000003.12 (160404588..160404685)		(9)
**hsa-miR-15b-5p**	fALS	Down	Human miFinder PCR array	CSF	NC_000003.12 (160404588..160404685)		(53)
**hsa-miR-15b-5p**	MS	Down	NGS	Serum exosomes	NC_000003.12 (160404588..160404685)		(32)
**hsa-miR-15b-5p**	sALS	Down	TLDA	CSF	NC_000003.12 (160404588..160404685)		(31)
**hsa-miR-16-2**	PD	Up	TLDA	CSF exosomes	NC_000003.12 (160404745..160404825)		(14)
**hsa-miR-16-2-3p**	MS	Up	NGS, microarray & qPCR	Blood	NC_000003.12 (160404745..160404825)		(54)
**hsa-miR-16-2-3p**	PD	Up	Microarray & qPCR	Blood	NC_000003.12 (160404745..160404825)		(40)
**hsa-miR-16-2-3p**	PD	Down	NGS	Serum	NC_000003.12 (160404745..160404825)		(13)

**hsa-miR-16-5p**	AD	Down	TLDA	CSF	NC_000013.11 (50048973..50049061, complement)	NC_000003.12 (160404745..160404825)	(31)
**hsa-miR-16-5p**	AD	Down	Exiqon panel & qPCR	CSF	NC_000013.11 (50048973..50049061, complement)	NC_000003.12 (160404745..160404825)	(30)
**hsa-miR-16-5p**	MS	Up	qPCR	CSF	NC_000013.11 (50048973..50049061, complement)	NC_000003.12 (160404745..160404825)	(39)
**hsa-miR-17-5p**	fALS	Up	Microarray Meta-analysis	Serum	NC_000013.11 (91350605..91350688)		(4)
**hsa-miR-17-5p**	sALS	Up	Microarray Meta-analysis	Serum	NC_000013.11 (91350605..91350688)		(4)
**hsa-miR-181-5p**	fALS	Up	Microarray meta-analysis	Serum			(4)
**hsa-miR-181-5p**	sALS	Up	Human miFinder PCR array	CSF			(53)
**hsa-miR-181a**	PD	Down	NGS & qPCR	Serum	NC_000001.11 (198859044..198859153, complement)	NC_000009.12 (124692442..124692551)	(52)
**hsa-miR-181a-3p**	AD	Down	NGS	CSF	NC_000001.11 (198859044..198859153, complement)	NC_000009.12 (124692442..124692551)	(13)
**hsa-miR-181a-5p**	AD	Down	NGS	CSF	NC_000001.11 (198859044..198859153, complement)	NC_000009.12 (124692442..124692551)	(13)
**hsa-miR-181c**	AD	Down	qPCR	Serum	NC_000019.10 (13874699..13874808)		(38)
**hsa-miR-181c-3p**	AD	Down	NGS & qPCR	Serum	NC_000019.10 (13874699..13874808)		(20)
**hsa-miR-181d**	AD	Down	NGS	CSF	NC_000019.10 (13874875..13875011)		(13)
**hsa-miR-1825**	fALS	Down	Microarray meta-analysis	Serum	NC_000020.11 (32237795..32237847)		(4)
**hsa-miR-1825**	sALS	Down	Microarray & qPCR	Leukocytes	NC_000020.11 (32237795..32237847)		(22)

**hsa-miR-1825**	sALS	Down	Microarray meta-analysis	Serum	NC_000020.11 (32237795..32237847)		(4)
**hsa-miR-1825**	sALS	Down	Microarray & qPCR	Serum	NC_000020.11 (32237795..32237847)		(23)
**hsa-miR-182-5p**	AD	Down	NGS	Serum	NC_000007.14 (129770383..129770492, complement)		(13)
**hsa-miR-1826**	PD	Up	Microarray & qPCR	Plasma	NC_000016.9 (33965508..33965592)		(55)
**hsa-miR-183**	sALS	Down	microarray & qPCR	Leukocytes	NC_000007.14 (129774905..129775014, complement)		(56)
**hsa-miR-184**	AD	Down	NGS	CSF	NC_000015.10 (79209788..79209871)		(13)
**hsa-miR-184**	AD	Up	NGS	Serum	NC_000015.10 (79209788..79209871)		(13)
**hsa-miR-185**	PD	Down	NGS & qPCR	Serum	NC_000022.11 (20033139..20033220)		(52)
**hsa-miR-18a-5p**	AD	Down	Exiqon qPCR panel	Plasma	NC_000013.11 (91350751..91350821)		(18)
**hsa-miR-18b-5p**	AD	Up	NGS & qPCR	Serum exosomes	NC_000023.11 (134170041..134170111, complement)		(17)
**hsa-miR-1915-3p**	fALS	Down	Microarray & qPCR	Serum	NC_000010.11 (21496562..21496641, complement)		(44)
**hsa-miR-191-5p**	AD	Down	nCounter Nanostring & qPCR	Plasma	NC_000003.12 (49020618..49020709, complement)		(8)
**hsa-miR-191-5p**	AD	Down	NGS & qPCR	Serum	NC_000003.12 (49020618..49020709, complement)		(10)
**hsa-miR-191-5p**	MS	Up	qPCR	Serum	NC_000003.12 (49020618..49020709, complement)		(34)
**hsa-miR-192-3p**	fALS	Up	Exiqon qPCR panel & qPCR	Serum	NC_000011.10 (64891137..64891246, complement)		(7)

**hsa-miR-192-3p**	sALS	Up	Exiqon qPCR panel & qPCR	Serum	NC_000011.10 (64891137..64891246, complement)		(7)
**hsa-miR-192-5p**	fALS	Up	Exiqon qPCR panel & qPCR	Serum	NC_000011.10 (64891137..64891246, complement)		(7)
**hsa-miR-192-5p**	sALS	Up	Exiqon qPCR panel & qPCR	Serum	NC_000011.10 (64891137..64891246, complement)		(7)
**hsa-miR-193a-3p**	PD	Down	NGS & qPCR	Serum	NC_000017.11 (31559996..31560083)		(41)
**hsa-miR-193a-5p**	AD	Down	TLDA	CSF	NC_000017.11 (31559996..31560083)		(31)
**hsa-miR-193b**	AD	Down	qPCR	Serum	NC_000016.10 (14303967..14304049)		(37)
**hsa-miR-193b**	sALS	Down	microarray & qPCR	Leukocytes	NC_000016.10 (14303967..14304049)		(56)
**hsa-miR-194-5p**	AD	Down	qPCR	Blood	C_000001.11 (220118157..220118241, complement)	NC_000011.10 (64891355..64891439, complement)	(15)
**hsa-miR-195**	PD	Up	NGS & qPCR	Serum	NC_000017.11 (7017615..7017701, complement)		(52)
**hsa-miR-195**	PD	Up	qPCR	Serum exosomes	NC_000017.11 (7017615..7017701, complement)		(57)
**hsa-miR-195-5p**	AD	Down	TLDA	CSF	NC_000017.11 (7017615..7017701, complement)		(31)
**hsa-miR-196b-5p**	MS	Down	NGS & ddPCR	Serum exosomes	NC_000007.14 (27169480..27169563, complement)		(21)
**hsa-miR-197**	AD	Down	Microarray & qPCR	Serum	NC_000001.11 (109598893..109598967)		(12)
**hsa-miR-197**	MS	Up	Microarray	PBMCs	NC_000001.11 (109598893..109598967)		(29)
**hsa-miR-199a**	MS	Down	Microarray & qPCR	PBMCs	NC_000019.10 (10817426..10817496, complement)	NC_000001.11 (172144535..172144644, complement)	(25)

**hsa-miR-199b-5p**	AD	Down	NGS	CSF	NC_000009.12 (128244721..128244830, complement)		(13)
**hsa-miR-199b-5p**	PD	Up	NGS	PBMCs	NC_000009.12 (128244721..128244830, complement)		(33)
**hsa-miR-19a**	MS	Up	Microarray & qPCR	PBMCs	NC_000013.11 (91350891..91350972)		(43)
**hsa-miR-19a-3p**	fALS	Up	Exiqon qPCR panel & qPCR	Serum	NC_000013.11 (91350891..91350972)		(7)
**hsa-miR-19a-3p**	PD	Up	NGS	CSF	NC_000013.11 (91350891..91350972)		(13)
**hsa-miR-19a-3p**	PD	Down	qPCR	Serum	NC_000013.11 (91350891..91350972)		(58)
**hsa-miR-19a-3p**	sALS	Up	Exiqon qPCR panel & qPCR	Serum	NC_000013.11 (91350891..91350972)		(7)
**hsa-miR-19b**	PD	Down	qPCR	Serum exosomes	NC_000013.11 (91351192..91351278)	NC_000023.11 (134169671..134169766, complement)	(57)
**hsa-miR-19b-3p**	PD	Down	TLDA	CSF	NC_000013.11 (91351192..91351278)	NC_000023.11 (134169671..134169766, complement)	(31)
**hsa-miR-19b-3p**	PD	Up	NGS	CSF	NC_000013.11 (91350891..91350972)	NC_000023.11 (134169671..134169766, complement)	(13)
**hsa-miR-19b-3p**	PD	Down	TLDA	CSF exosomes	NC_000013.11 (91351192..91351278)	NC_000023.11 (134169671..134169766, complement)	(14)
**hsa-miR-19b-3p**	PD	Down	qPCR	Serum	NC_000013.11 (91351192..91351278)	NC_000023.11 (134169671..134169766, complement)	(58)
**hsa-miR-200a-3p**	AD	Up	Exiqon qPCR panel	Plasma	NC_000001.11 (1167863..1167952)		(18)
**hsa-miR-200a-3p**	PD	Up	qPCR	CSF	NC_000001.11 (1167863..1167952)		(46)
**hsa-miR-200c**	MS	Up	Microarray & qPCR	PBMCs	NC_000012.12 (6963699..6963766)		(25)

**hsa-miR-202-3p**	AD	Up	TLDA	CSF	NC_000010.11 (133247511..133247620, complement)		(31)
**hsa-miR-205**	PD	Up	qPCR	CSF	NC_000001.11 (209432133..209432242)		(59)
**hsa-miR-206**	fALS	Up	TLDA & qPCR	Serum	NC_000006.12 (52144349..52144434)		(45)
**hsa-miR-206**	fALS	Up	qPCR	Serum	NC_000006.12 (52144349..52144434)		(60)
**hsa-miR-206**	sALS	Up	TLDA & qPCR	Serum	NC_000006.12 (52144349..52144434)		(45)
**hsa-miR-206**	sALS	Up	qPCR	Serum	NC_000006.12 (52144349..52144434)		(60)
**hsa-miR-20a-5p**	AD	Up	NGS & qPCR	Serum exosomes	NC_000013.11 (91351065..91351135)		(17)
**hsa-miR-20a-5p**	MS	Down	NGS, microarray & qPCR	Blood	NC_000013.11 (91351065..91351135)		(54)
**hsa-miR-21**	MS	Up	Microarray & qPCR	PBMCs	NC_000017.11 (59841266..59841337)		(43)
**hsa-miR-2110**	fALS	Up	Exiqon qPCR panel & qPCR	Serum	NC_000010.11 (114174105..114174179, complement)		(7)
**hsa-miR-2110**	sALS	Up	Exiqon qPCR panel & qPCR	Serum	NC_000010.11 (114174105..114174179, complement)		(7)
**hsa-miR-212-3p**	PD	Down	NGS	CSF	NC_000017.11 (2050271..2050380, complement)		(13)
**hsa-miR-214**	PD	Down	NGS & qPCR	Serum	NC_000001.11 (172138798..172138907, complement)		(41)
**hsa-miR-214**	PD	Down	qPCR	Serum	NC_000001.11 (172138798..172138907, complement)		(48)
**hsa-miR-21-5p**	AD	Down	NGS	Serum	NC_000017.11 (59841266..59841337)		(13)
**hsa-miR-21-5p**	fALS	Down	Human miFinder PCR array	CSF	NC_000017.11 (59841266..59841337)		(53)

**hsa-miR-21-5p**	PD	Up	NGS	PBMCs	NC_000017.11 (59841266..59841337)		(33)
**hsa-miR-21-5p**	sALS	Down	Human miFinder PCR array	CSF	NC_000017.11 (59841266..59841337)		(53)
**hsa-miR-218-5p**	AD	Down	NGS	CSF	NC_000004.12 (20528275..20528384)		(13)
**hsa-miR-219**	AD	Down	OpenArray qPCR	CSF	NC_000006.12 (33207835..33207944)	NC_000009.12 (128392618..128392714, complement)	(16)
**hsa-miR-219**	MS	Down	OpenArray qPCR	CSF	NC_000006.12 (33207835..33207944)	NC_000009.12 (128392618..128392714, complement)	(61)
**hsa-miR-219-2-3p**	AD	Up	NGS	Serum	NC_000009.12 (128392618..128392714, complement)		(13)
**hsa-miR-22**	MS	Up	Microarray & qPCR	PBMCs	NC_000017.11 (1713903..1713987, complement)		(43)
**hsa-miR-221**	PD	Down	qPCR	Serum	NC_000023.11 (45746157..45746266, complement)		(48)
**hsa-miR-221**	PD	Down	NGS & qPCR	Serum	NC_000023.11 (45746157..45746266, complement)		(52)
**hsa-miR-221-3p**	AD	Up	NGS & qPCR	Serum	NC_000023.11 (45746157..45746266, complement)		(20)
**hsa-miR-221-3p**	fALS	Up	Microarray meta-analysis	Serum	NC_000023.11 (45746157..45746266, complement)		(4)
**hsa-miR-221-3p**	sALS	Up	Microarray meta-analysis	Serum	NC_000023.11 (45746157..45746266, complement)		(4)
**hsa-miR-222**	AD	Up	TLDA	CSF	NC_000023.11 (45747015..45747124, complement)		(26)
**hsa-miR-222**	PD	Down	miRGenes qPCR panel	Plasma	NC_000023.11 (45747015..45747124, complement)		(3)

**hsa-miR-222**	PD	Down	Microarray & qPCR	Plasma	NC_000023.11 (45747015..45747124, complement)		(55)
**hsa-miR-222-3p**	PD	Up	Microarray & qPCR	Plasma	NC_000023.11 (45747015..45747124, complement)		(55)
**hsa-miR-223**	MS	Down	qPCR	Serum	NC_000023.11 (66018870..66018979)		(51)
**hsa-miR-223-3p**	AD	Down	TLDA	CSF	NC_000023.11 (66018870..66018979)		(31)
**hsa-miR-223-3p**	MS	Down	NGS	Serum exosomes	NC_000023.11 (66018870..66018979)		(32)
**hsa-miR-223-5p**	PD	Up	Microarray	Serum	NC_000023.11 (66018870..66018979)		(49)
**hsa-miR-22-3p**	AD	Down	NGS & qPCR	Serum	NC_000017.11 (1713903..1713987, complement)		(20)
**hsa-miR-22-3p**	PD	Down	NGS	CSF	NC_000017.11 (1713903..1713987, complement)		(11)
**hsa-miR-22-5p**	AD	Up	NGS	Serum	NC_000017.11 (1713903..1713987, complement)		(13)
**hsa-miR-22-5p**	PD	Up	Microarray & qPCR	Blood	NC_000017.11 (1713903..1713987, complement)		(40)
**hsa-miR-2278**	fALS	Up	Microarray Meta-analysis	Serum	NC_000009.12 (94809962..94810057)		(4)
**hsa-miR-2278**	sALS	Up	Microarray Meta-analysis	Serum	NC_000009.12 (94809962..94810057)		(4)
**hsa-miR-23-3p**	MS	Down	NGS	Serum exosomes			(32)
**hsa-miR-2392**	fALS	Up	Microarray Meta-analysis	Serum	NC_000014.9 (100814491..100814574)		(4)
**hsa-miR-2392**	sALS	Up	Microarray Meta-analysis	Serum	NC_000014.9 (100814491..100814574)		(4)
**hsa-miR-23a**	MS	Down	qPCR	Serum	NC_000019.10 (13836587..13836659, complement)		(51)
**hsa-miR-23a-3p**	AD	Down	NGS & qPCR	Plasma exosomes	NC_000019.10 (13836587..13836659, complement)		(42)

**hsa-miR-23b-3p**	AD	Down	NGS & qPCR	Plasma exosomes	NC_000009.12 (95085208..95085304)		(42)
**hsa-miR-24**	MS	Up	qPCR	CSF	NC_000009.12 (95086021..95086088)		(39)
**hsa-miR-24**	PD	Up	qPCR	CSF	NC_000009.12 (95086021..95086088)		(59)
**hsa-miR-24**	PD	Up	qPCR	Serum exosomes	NC_000009.12 (95086021..95086088)		(57)
**hsa-miR-24-3p**	AD	Down	TLDA	CSF	NC_000009.12 (95086021..95086088)		(31)
**hsa-miR-24-3p**	AD	Down	NGS & qPCR	Plasma exosomes	NC_000009.12 (95086021..95086088)		(42)
**hsa-miR-24-3p**	MS	Up	qPCR	Serum	NC_000009.12 (95086021..95086088)		(34)
**hsa-miR-24-3p**	PD	Up	Microarray	Serum	NC_000009.12 (95086021..95086088)		(49)
**hsa-miR-25-3p**	fALS	Up	Microarray meta-analysis	Serum	NC_000007.14 (100093560..100093643, complement)		(4)
**hsa-miR-25-3p**	MS	Up	Exiqon qPCR panel & qPCR	Serum	NC_000007.14 (100093560..100093643, complement)		(6)
**hsa-miR-25-3p**	sALS	Up	Microarray meta-analysis	Serum	NC_000007.14 (100093560..100093643, complement)		(4)
**hsa-miR-25-5p**	AD	Down	Microarray & qPCR	PBMCs	NC_000007.14 (100093560..100093643, complement)		(35)
**hsa-miR-26a-2-3p**	PD	Up	Microarray & qPCR	Blood	NC_000012.12 (57824609..57824692, complement)		(40)
**hsa-miR-26a-5p**	AD	Down	NGS & qPCR	Serum	NC_000003.12 (37969404..37969480)	NC_000012.12 (57824609..57824692, complement)	(20)
**hsa-miR-26a-5p**	fALS	Up	Microarray meta-analysis	Serum	NC_000003.12 (37969404..37969480)	NC_000012.12 (57824609..57824692, complement)	(4)

**hsa-miR-26a-5p**	sALS	Up	Microarray meta-analysis	Serum	NC_000003.12 (37969404..37969480)	NC_000012.12 (57824609..57824692, complement)	(4)
**hsa-miR-26b**	AD	Down	Microarray & qPCR	Serum	NC_000002.12 (218402646..218402722)		(28)
**hsa-miR-26b-3p**	AD	Up	NGS & qPCR	Serum	NC_000002.12 (218402646..218402722)		(10)
**hsa-miR-26b-5p**	AD	Down	NGS & qPCR	Blood	NC_000002.12 (218402646..218402722)		(1)
**hsa-miR-26b-5p**	AD	Down	TLDA	CSF	NC_000002.12 (218402646..218402722)		(31)
**hsa-miR-27a**	PD	Up	miRGenes qPCR panel	Plasma	NC_000019.10 (13836440..13836517, complement)		(3)
**hsa-miR-27a-3p**	AD	Down	Exiqon qPCR panel & qPCR	CSF	NC_000019.10 (13836440..13836517, complement)		(62)
**hsa-miR-27a-3p**	AD	Up	Exiqon qPCR panel & qPCR	Serum	NC_000019.10 (13836440..13836517, complement)		(10)
**hsa-miR-27a-3p**	MS	Up	NGS & qPCR	Serum	NC_000019.10 (13836440..13836517, complement)		(6)
**hsa-miR-27a-3p**	PD	Down	NGS	CSF	NC_000019.10 (13836440..13836517, complement)		(11)
**hsa-miR-2861**	fALS	Down	Microarray Meta-analysis	Serum	NC_000009.12 (127785918..127786007)		(4)
**hsa-miR-2861**	sALS	Down	Microarray Meta-analysis	Serum	NC_000009.12 (127785918..127786007)		(4)
**hsa-miR-296**	AD	Up	OpenArray qPCR	CSF	NC_000020.11 (58817615..58817694, complement)		(16)
**hsa-miR-29a**	AD	Down	Microarray & qPCR	CSF	NC_000007.14 (130876747..130876810, complement)		(12)
**hsa-miR-29a**	AD	Up	qPCR	CSF	NC_000007.14 (130876747..130876810, complement)		(27)

**hsa-miR-29a**	AD	Up	qPCR	Serum	NC_000007.14 (130876747..130876810, complement)		(63)
**hsa-miR-29a**	AD	Down	qPCR	Serum	NC_000007.14 (130876747..130876810, complement)		(38)
**hsa-miR-29a**	PD	Down	qPCR	Serum	NC_000007.14 (130876747..130876810, complement)		(64)
**hsa-miR-29a-3p**	AD	Down	TLDA	CSF	NC_000007.14 (130876747..130876810, complement)		(31)
**hsa-miR-29a-3p**	PD	Up	Microarray & qPCR	Blood	NC_000007.14 (130876747..130876810, complement)		(40)
**hsa-miR-29a-3p**	PD	Up	qPCR	PBMCs	NC_000007.14 (130876747..130876810, complement)		(19)
**hsa-miR-29a-3p**	PD	Down	qPCR	Serum	NC_000007.14 (130876747..130876810, complement)		(58)
**hsa-miR-29b**	AD	Up	qPCR	CSF	NC_000007.14 (130877459..130877539, complement)		(27)
**hsa-miR-29b**	AD	Down	qPCR	Serum	NC_000007.14 (130877459..130877539, complement)		(38)
**hsa-miR-29b-3p**	AD	Down	NGS & qPCR	Plasma exosomes	NC_000007.14 (130877459..130877539, complement)		(42)
**hsa-miR-29b-3p**	PD	Down	qPCR	Serum	NC_000007.14 (130877459..130877539, complement)		(64)
**hsa-miR-29c**	AD	Down	TLDAA	CSF exosomes	NC_000001.11 (207801852..207801939, complement)		(14)
**hsa-miR-29c**	PD	Down	qPCR	Serum	NC_000001.11 (207801852..207801939, complement)		(48)

**hsa-miR-29c**	PD	Down	qPCR	Serum	NC_000001.11 (207801852..207801939, complement)		(64)
**hsa-miR-29c-3p**	AD	Down	qPCR	CSF	NC_000001.11 (207801852..207801939, complement)		(15)
**hsa-miR-29c-3p**	PD	Up	NGS	PBMCs	NC_000001.11 (207801852..207801939, complement)		(33)
**hsa-miR-29c-3p**	PD	Down	qPCR	Serum	NC_000001.11 (207801852..207801939, complement)		(58)
**hsa-miR-301a-3p**	AD	Down	Exiqon qPCR panel	Plasma	NC_000017.11 (59151136..59151221, complement)		(18)
**hsa-miR-301a-3p**	MS	Down	NGS & ddPCR	Serum exosomes	NC_000017.11 (59151136..59151221, complement)		(21)
**hsa-miR-3065-5p**	AD	Up	NGS & qPCR	Serum exosomes	NC_000017.11 (59151136..59151221, complement)		(17)
**hsa-miR-30a-3p**	AD	Down	TLDA	CSF	NC_000006.12 (71403551..71403621, complement)		(31)
**hsa-miR-30a-3p**	PD	Down	NGS	Serum	NC_000006.12 (71403551..71403621, complement)		(13)
**hsa-miR-30a-5p**	PD	Up	Microarray & qPCR	Blood	NC_000006.12 (71403551..71403621, complement)		(40)
**hsa-miR-30a-5p**	PD	Up	qPCR	Plasma	NC_000006.12 (71403551..71403621, complement)		(65)
**hsa-miR-30b-5p**	AD	Down	Exiqon qPCR panel & qPCR	Plasma	NC_000008.11 (134800520..134800607, complement)		(18)
**hsa-miR-30b-5p**	fALS	Down	Exiqon qPCR panel & qPCR	Serum	NC_000008.11 (134800520..134800607, complement)		(7)

**hsa-miR-30b-5p**	MS	Down	NGS	Serum exosomes	NC_000008.11 (134800520..134800607, complement)		(32)
**hsa-miR-30b-5p**	PD	Up	qPCR	PBMCs	NC_000008.11 (134800520..134800607, complement)		(19)
**hsa-miR-30b-5p**	PD	Up	qPCR	Plasma	NC_000008.11 (134800520..134800607, complement)		(65)
**hsa-miR-30b-5p**	sALS	Down	Exiqon qPCR panel & qPCR	Serum	NC_000008.11 (134800520..134800607, complement)		(7)
**hsa-miR-30c**	MS	Up	Microarray	PBMCs	NC_000001.11 (40757284..40757372)	NC_000006.12 (71376960..71377031, complement)	(29)
**hsa-miR-30c-2-3p**	AD	Up	NGS	Serum	NC_000006.12 (71376960..71377031, complement)		(13)
**hsa-miR-30c-5p**	PD	Down	Microarray	Serum	NC_000001.11 (40757284..40757372)	NC_000006.12 (71376960..71377031, complement)	(49)
**hsa-miR-30d-5p**	AD	Down	TLDA	CSF	NC_000008.11 (134804876..134804945, complement)		(31)
**hsa-miR-30e-5p**	AD	Down	NGS & qPCR	Serum	NC_000001.11 (40754355..40754446)		(10)
**hsa-miR-30e-5p**	AD	Up	NGS & qPCR	Serum exosomes	NC_000001.11 (40754355..40754446)		(17)
**hsa-miR-30e-5p**	PD	Up	NGS	PBMCs	NC_000001.11 (40754355..40754446)		(33)
**hsa-miR-30e-5p**	PD	Down	NGS	Serum	NC_000001.11 (40754355..40754446)		(13)
**hsa-miR-3158-3p**	AD	Up	NGS & qPCR	Serum	NC_000010.11 (101601417..101601497)		(10)
**hsa-miR-3175**	fALS	Up	Microarray Meta-analysis	Serum	NC_000015.10 (92904399..92904475)		(4)
**hsa-miR-3175**	sALS	Up	Microarray Meta-analysis	Serum	NC_000015.10 (92904399..92904475)		(4)

**hsa-miR-3176**	AD	Up	NGS	Serum	NC_000016.10 (543277..543366)		(13)
**hsa-miR-3185**	fALS	Down	Microarray Meta-analysis	Serum	NC_000017.11 (48724408..48724475, complement)		(4)
**hsa-miR-3185**	sALS	Down	Microarray Meta-analysis	Serum	NC_000017.11 (48724408..48724475, complement)		(4)
**hsa-miR-3196**	fALS	Down	Microarray Meta-analysis	Serum	NC_000020.11 (63238779..63238842)		(4)
**hsa-miR-3196**	sALS	Down	Microarray Meta-analysis	Serum	NC_000020.11 (63238779..63238842)		(4)
**hsa-miR-3200-3p**	AD	Down	NGS	CSF	NC_000022.11 (30731557..30731641)		(13)
**hsa-miR-320a**	AD	Up	Exiqon qPCR panel & qPCR	Plasma	NC_000008.11 (22244962..22245043, complement)		(18)
**hsa-miR-320a**	fALS	Down	Exiqon qPCR panel & qPCR	Serum	NC_000008.11 (22244962..22245043, complement)		(7)
**hsa-miR-320a**	MS	Up	Exiqon qPCR panel & qPCR	Serum	NC_000008.11 (22244962..22245043, complement)		(6)
**hsa-miR-320a**	sALS	Down	Exiqon qPCR panel & qPCR	Serum	NC_000008.11 (22244962..22245043, complement)		(7)
**hsa-miR-320a-3p**	PD	Down	NGS	PBMCs	NC_000008.11 (22244962..22245043, complement)		(33)
**hsa-miR-320b**	AD	Up	Exiqon qPCR panel & qPCR	Plasma	NC_000001.11 (116671749..116671827)	NC_000001.11 (224257004..224257141, complement)	(18)
**hsa-miR-320b**	fALS	Down	Exiqon qPCR panel & qPCR	Serum	NC_000001.11 (116671749..116671827)	NC_000001.11 (224257004..224257141, complement)	(7)
**hsa-miR-320b**	MS	Up	Exiqon qPCR panel & qPCR	Serum	NC_000001.11 (116671749..116671827)	NC_000001.11 (224257004..224257141, complement)	(6)

**hsa-miR-320b**	sALS	Down	Exiqon qPCR panel & qPCR	Serum	NC_000001.11 (116671749..116671827)	NC_000001.11 (224257004..224257141, complement)	(7)
**hsa-miR-320c**	AD	Up	Exiqon qPCR panel & qPCR	Plasma	NC_000018.10 (21683510..21683597)	NC_000018.10 (24321686..24321735)	(18)
**hsa-miR-320c**	fALS	Down	Exiqon qPCR panel & qPCR	Serum	NC_000018.10 (21683510..21683597)	NC_000018.10 (24321686..24321735)	(7)
**hsa-miR-320c**	sALS	Down	Exiqon qPCR panel & qPCR	Serum	NC_000018.10 (21683510..21683597)	NC_000018.10 (24321686..24321735)	(7)
**hsa-miR-323a-3p**	AD	Down	NGS	CSF	NC_000014.9 (101025732..101025817)		(13)
**hsa-miR-324-3p**	fALS	Up	Microarray Meta-analysis	Serum	NC_000017.11 (7223297..7223379, complement)		(4)
**hsa-miR-324-3p**	PD	Up	Microarray	Serum	NC_000017.11 (7223297..7223379, complement)		(49)
**hsa-miR-324-3p**	sALS	Up	Microarray Meta-analysis	Serum	NC_000017.11 (7223297..7223379, complement)		(4)
**hsa-miR-326**	AD	Down	NGS	CSF	NC_000011.10 (75335092..75335186, complement)		(13)
**hsa-miR-326**	MS	Up	Microarray & qPCR	PBMCs	NC_000011.10 (75335092..75335186, complement)		(43)
**hsa-miR-328**	MS	Down	Microarray & qPCR	PBMCs	NC_000016.10 (67202321..67202395, complement)		(25)
**hsa-miR-328**	sALS	Down	Microarray & qPCR	Leukocytes	NC_000016.10 (67202321..67202395, complement)		(22)
**hsa-miR-328-3p**	AD	Down	TLDA	CSF	NC_000016.10 (67202321..67202395, complement)		(31)
**hsa-miR-329**	AD	Down	NGS	CSF	NC_000014.9 (101026785..101026864)		(13)
**hsa-miR-331-3p**	fALS	Down	Exiqon qPCR panel & qPCR	Serum	NC_000012.12 (95308420..95308513)		(7)

**hsa-miR-331-3p**	sALS	Down	Exiqon qPCR panel & qPCR	Serum	NC_000012.12 (95308420..95308513)		(7)
**hsa-miR-331-5p**	AD	Down	TLDA	CSF exosomes	NC_000012.12 (95308420..95308513)		(14)
**hsa-miR-331-5p**	PD	Up	TLDA	CSF exosomes	NC_000012.12 (95308420..95308513)		(14)
**hsa-miR-331-5p**	PD	Up	qPCR	Plasma	NC_000012.12 (95308420..95308513)		(66)
**hsa-miR-335**	AD	Down	OpenArray qPCR	CSF	NC_000007.14 (130496111..130496204)		(16)
**hsa-miR-335-5p**	AD	Up	NGS & qPCR	Serum exosomes	NC_000007.14 (130496111..130496204)		(17)
**hsa-miR-338-3p**	PD	Up	NGS	Serum	NC_000017.11 (81125883..81125949, complement)		(13)
**hsa-miR-338-3p**	sALS	Up	Microarray & qPCR	Leukocytes	NC_000017.11 (81125883..81125949, complement)		(22)
**hsa-miR-339-5p**	AD	Up	Microarray & qPCR	PBMCs	NC_000007.14 (1022933..1023026, complement)		(35)
**hsa-miR-33a-5p**	AD	Down	Exiqon qPCR panel	Plasma	NC_000022.11 (41900944..41901012)		(18)
**hsa-miR-33b-5p**	AD	Down	NGS	CSF	NC_000017.11 (17813836..17813931, complement)		(13)
**hsa-miR-340-5p**	AD	Down	TLDA	CSF	NC_000005.10 (180015303..180015397, complement)		(31)
**hsa-miR-342-3p**	AD	Down	NGS & qPCR	Plasma exosomes	NC_000014.9 (100109655..100109753)		(42)
**hsa-miR-342-3p**	AD	Down	NGS & qPCR	Serum	NC_000014.9 (100109655..100109753)		(10)
**hsa-miR-342-3p**	AD	Down	NGS & qPCR	Serum exosomes	NC_000014.9 (100109655..100109753)		(17)
**hsa-miR-342-3p**	MS	Down	NGS	Serum exosomes	NC_000014.9 (100109655..100109753)		(32)

**hsa-miR-342-5p**	AD	Down	NGS & qPCR	Plasma exosomes	NC_000014.9 (100109655..100109753)		(42)
**hsa-miR-342-5p**	AD	Down	NGS & qPCR	Serum exosomes	NC_000014.9 (100109655..100109753)		(17)
**hsa-miR-34a**	AD	Down	qPCR	CSF	NC_000001.11 (9151668..9151777, complement)		(27)
**hsa-miR-34a**	AD	Down	qPCR	Plasma	NC_000001.11 (9151668..9151777, complement)		(27)
**hsa-miR-34a-5p**	AD	Down	qPCR	Plasma	NC_000001.11 (9151668..9151777, complement)		(9)
**hsa-miR-34b-3p**	AD	Up	NGS	Serum	NC_000011.10 (111512938..111513021)		(13)
**hsa-miR-34b-5p**	AD	Up	NGS	Serum	NC_000011.10 (111512938..111513021)		(13)
**hsa-miR-34c-5p**	AD	Down	qPCR	Plasma	NC_000011.10 (111513439..111513515)		(9)
**hsa-miR-34c-5p**	AD	Up	NGS	Serum	NC_000011.10 (111513439..111513515)		(13)
**hsa-miR-3607-3p**	AD	Up	Microarray & qPCR	Serum	NC_000005.10 (86620506..86620568)		(35)
**hsa-miR-3613-5p**	fALS	Down	Microarray Meta-analysis	Serum	NC_000013.11 (49996415..49996501, complement)		(4)
**hsa-miR-3613-5p**	sALS	Down	Microarray Meta-analysis	Serum	NC_000013.11 (49996415..49996501, complement)		(4)
**hsa-miR-361-5p**	AD	Up	NGS & qPCR	Serum exosomes	NC_000023.11 (85903636..85903707, complement)		(17)
**hsa-miR-3622b-3p**	AD	Up	OpenArray qPCR	CSF	NC_000008.11 (27701673..27701767, complement)		(16)
**hsa-miR-365a-3p**	AD	Down	TLDA	CSF	NC_000016.10 (14309285..14309371)		(31)
**hsa-miR-365a-3p**	MS	Down	Exiqon qPCR panel & qPCR	Serum	NC_000016.10 (14309285..14309371)		(6)

**hsa-miR-3665**	fALS	Down	Microarray & qPCR	Serum	NC_000013.11 (77698012..77698116, complement)		(44)
**hsa-miR-3665**	fALS	Down	Microarray Meta-analysis	Serum	NC_000013.11 (77698012..77698116, complement)		(4)
**hsa-miR-3665**	sALS	Down	Microarray & qPCR	Serum	NC_000013.11 (77698012..77698116, complement)		(23)
**hsa-miR-3665**	sALS	Down	Microarray Meta-analysis	Serum	NC_000013.11 (77698012..77698116, complement)		(4)
**hsa-miR-370**	MS	Down	NGS	Serum exosomes	NC_000014.9 (100911139..100911213)		(32)
**hsa-miR-370**	PD	Down	NGS	CSF	NC_000014.9 (100911139..100911213)		(13)
**hsa-miR-374-5p**	fALS	Down	TLDA & qPCR	Serum	NC_000023.11 (74287286..74287357, complement)		(45)
**hsa-miR-374-5p**	sALS	Down	TLDA & qPCR	Serum	NC_000023.11 (74287286..74287357, complement)		(45)
**hsa-miR-375**	AD	Down	OpenArray qPCR	CSF	NC_000002.12 (219001645..219001708, complement)		(16)
**hsa-miR-375**	AD	Down	NGS	Serum	NC_000002.12 (219001645..219001708, complement)		(13)
**hsa-miR-375**	MS	Down	qPCR	Serum	NC_000002.12 (219001645..219001708, complement)		(34)
**hsa-miR-376c-3p**	MS	Up	qPCR	Serum	NC_000014.9 (101039690..101039755)		(34)
**hsa-miR-377-5p**	AD	Down	NGS	CSF	NC_000014.9 (101062050..101062118)		(13)
**hsa-miR-378a-3p**	AD	Up	TLDA	CSF	NC_000005.10 (149732825..149732890)		(31)

**hsa-miR-378i**	fALS	Up	Microarray Meta-analysis	Serum	NC_000022.11 (41923222..41923297, complement)		(4)
**hsa-miR-378i**	sALS	Up	Microarray Meta-analysis	Serum	NC_000022.11 (41923222..41923297, complement)		(4)
**hsa-miR-381**	AD	Down	NGS	CSF	NC_000014.9 (101045920..101045994)		(13)
**hsa-miR-384**	AD	Up	qPCR	Serum	NC_000023.11 (76919273..76919360, complement)		(37)
**hsa-miR-3935**	sALS	Down	Microarray & qPCR	Leukocytes	NC_000016.10 (56245520..56245623)		(56)
**hsa-miR-3940-5p**	fALS	Down	Microarray Meta-analysis	Serum	NC_000019.10 (6416410..6416511, complement)		(4)
**hsa-miR-3940-5p**	sALS	Down	Microarray Meta-analysis	Serum	NC_000019.10 (6416410..6416511, complement)		(4)
**hsa-miR-3960**	fALS	Down	Microarray Meta-analysis	Serum	NC_000009.12 (127785833..127785923)		(4)
**hsa-miR-3960**	sALS	Down	Microarray Meta-analysis	Serum	NC_000009.12 (127785833..127785923)		(4)
**hsa-miR-409-3p**	MS	Down	NGS	Serum exosomes	NC_000014.9 (101065300..101065378)		(32)
**hsa-miR-409-3p**	PD	Down	NGS	CSF	NC_000014.9 (101065300..101065378)		(13)
**hsa-miR-409-3p**	PD	Up	TLDA	CSF exosomes	NC_000014.9 (101065300..101065378)		(14)
**hsa-miR-410**	AD	Down	NGS	CSF	NC_000014.9 (101065912..101065991)		(13)
**hsa-miR-423-5p**	PD	Down	NGS	CSF	NC_000017.11 (30117079..30117172)		(11)
**hsa-miR-424-5p**	AD	Up	NGS & qPCR	Serum exosomes	NC_000023.11 (134546614..134546711, complement)		(17)
**hsa-miR-424-5p**	PD	Up	NGS	PBMCs	NC_000023.11 (134546614..134546711, complement)		(33)

**hsa-miR-425-5p**	AD	Up	Microarray & qPCR	PBMCs	NC_000003.12 (49020148..49020234, complement)		(35)
**hsa-miR-425-5p**	fALS	Down	Exiqon qPCR panel & qPCR	Serum	NC_000003.12 (49020148..49020234, complement)		(7)
**hsa-miR-425-5p**	sALS	Down	Exiqon qPCR panel & qPCR	Serum	NC_000003.12 (49020148..49020234, complement)		(7)
**hsa-miR-4270**	fALS	Down	Microarray Meta-analysis	Serum	NC_000003.12 (15496239..15496308, complement)		(4)
**hsa-miR-4270**	sALS	Down	Microarray Meta-analysis	Serum	NC_000003.12 (15496239..15496308, complement)		(4)
**hsa-miR-4297**	AD	Up	Microarray & qPCR	PBMCs	NC_000010.11 (129843299..129843374, complement)		(35)
**hsa-miR-4299**	sALS	Down	Microarray & qPCR	Plasma	NC_000011.10 (11656651..11656722, complement)		(67)
**hsa-miR-4306**	fALS	Up	Microarray Meta-analysis	Serum	NC_000013.11 (99643059..99643149)		(4)
**hsa-miR-4306**	sALS	Up	Microarray Meta-analysis	Serum	NC_000013.11 (99643059..99643149)		(4)
**hsa-miR-431-3p**	AD	Down	NGS	CSF	NC_000014.9 (100881007..100881120)		(13)
**hsa-miR-431-3p**	PD	Down	NGS	CSF	NC_000014.9 (100881007..100881120)		(13)
**hsa-miR-432-5p**	MS	Down	NGS	Serum exosomes	NC_000014.9 (100884483..100884576)		(32)
**hsa-miR-433**	AD	Down	NGS	CSF	NC_000014.9 (100881886..100881978)		(13)
**hsa-miR-433**	PD	Down	NGS	CSF	NC_000014.9 (100881886..100881978)		(13)
**hsa-miR-433**	PD	Down	qPCR	Plasma	NC_000014.9 (100881886..100881978)		(36)
**hsa-miR-4448**	PD	Down	NGS	CSF	NC_000003.12 (183886800..183886885)		(13)

**hsa-miR-4449**	AD	Down	OpenArray qPCR	CSF	NC_000004.12 (52712682..52712747)		(16)
**hsa-miR-4466**	fALS	Down	Microarray Meta-analysis	Serum	NC_000006.12 (156779678..156779731, complement)		(4)
**hsa-miR-4466**	sALS	Down	Microarray Meta-analysis	Serum	NC_000006.12 (156779678..156779731, complement)		(4)
**hsa-miR-4467**	AD	Up	OpenArray qPCR	CSF	NC_000007.14 (102471469..102471531)		(16)
**hsa-miR-4485-3p**	fALS	Up	Microarray Meta-analysis	Serum	NC_000011.10 (10508270..10508326, complement)		(4)
**hsa-miR-4485-3p**	sALS	Up	Microarray Meta-analysis	Serum	NC_000011.10 (10508270..10508326, complement)		(4)
**hsa-miR-4488**	fALS	Down	Microarray Meta-analysis	Serum	NC_000011.10 (61508596..61508657)		(4)
**hsa-miR-4488**	sALS	Down	Microarray Meta-analysis	Serum	NC_000011.10 (61508596..61508657)		(4)
**hsa-miR-4497**	fALS	Down	Microarray Meta-analysis	Serum	NC_000012.12 (109833348..109833436)		(4)
**hsa-miR-4497**	sALS	Down	Microarray Meta-analysis	Serum	NC_000012.12 (109833348..109833436)		(4)
**hsa-miR-4507**	fALS	Down	Microarray Meta-analysis	Serum	NC_000014.9 (105858124..105858175, complement)		(4)
**hsa-miR-4507**	sALS	Down	Microarray Meta-analysis	Serum	NC_000014.9 (105858124..105858175, complement)		(4)
**hsa-miR-4508**	fALS	Down	Microarray Meta-analysis	Serum	NC_000015.10 (23562062..23562131, complement)		(4)
**hsa-miR-4508**	sALS	Down	Microarray Meta-analysis	Serum	NC_000015.10 (23562062..23562131, complement)		(4)
**hsa-miR-451**	sALS	Down	Microarray & qPCR	Leukocytes	NC_000017.11 (28861369..28861440, complement)		(56)

**hsa-miR-451**	sALS	Down	Microarray & qPCR	Leukocytes	NC_000017.11 (28861369..28861440, complement)		(22)
**hsa-miR-4516**	fALS	Down	Microarray Meta-analysis	Serum	NC_000016.10 (2133119..2133204)		(4)
**hsa-miR-4516**	sALS	Down	Microarray Meta-analysis	Serum	NC_000016.10 (2133119..2133204)		(4)
**hsa-miR-451a**	AD	Down	Exiqon panel & qPCR	CSF	NC_000017.11 (28861369..28861440, complement)		(30)
**hsa-miR-451a**	fALS	Up	Microarray meta-analysis	Serum	NC_000017.11 (28861369..28861440, complement)		(4)
**hsa-miR-451a**	MS	Down	NGS	Serum exosomes	NC_000017.11 (28861369..28861440, complement)		(32)
**hsa-miR-451a**	sALS	Up	Microarray meta-analysis	Serum	NC_000017.11 (28861369..28861440, complement)		(4)
**hsa-miR-4530**	fALS	Down	Microarray & qPCR	Serum	NC_000019.10 (39409623..39409678, complement)		(44)
**hsa-miR-4530**	fALS	Down	Microarray Meta-analysis	Serum	NC_000019.10 (39409623..39409678, complement)		(4)
**hsa-miR-4530**	sALS	Down	Microarray & qPCR	Serum	NC_000019.10 (39409623..39409678, complement)		(44)
**hsa-miR-4530**	sALS	Down	Microarray Meta-analysis	Serum	NC_000019.10 (39409623..39409678, complement)		(4)
**hsa-miR-4532**	fALS	Down	Microarray Meta-analysis	Serum	NC_000020.11 (57895394..57895444)		(4)
**hsa-miR-4532**	sALS	Down	Microarray Meta-analysis	Serum	NC_000020.11 (57895394..57895444)		(4)
**hsa-miR-455-3p**	AD	Up	Microarray & qPCR	CSF	NC_000009.12 (114209434..114209529)		(68)
**hsa-miR-455-3p**	AD	Up	Microarray & qPCR	CSF	NC_000009.12 (114209434..114209529)		(69)

**hsa-miR-455-3p**	fALS	Down	Microarray Meta-analysis	Serum	NC_000009.12 (114209434..114209529)		(4)
**hsa-miR-455-3p**	sALS	Down	Microarray Meta-analysis	Serum	NC_000009.12 (114209434..114209529)		(4)
**hsa-miR-4639-5p**	PD	Up	Microarray and qPCR	Plasma	NC_000006.12 (16141556..16141624)		(70)
**hsa-miR-4649-5p**	sALS	Down	Microarray & qPCR	Plasma	NC_000007.14 (44110849..44110912)		(67)
**hsa-miR-4668-5p**	AD	Up	Microarray & qPCR	Serum	NC_000009.12 (111932100..111932169)		(68)
**hsa-miR-4674**	AD	Down	OpenArray qPCR	CSF	NC_000009.12 (136546173..136546259, complement)		(16)
**hsa-miR-4707-5p**	fALS	Down	Microarray Meta-analysis	Serum	NC_000014.9 (22956950..22957029, complement)		(4)
**hsa-miR-4707-5p**	sALS	Down	Microarray Meta-analysis	Serum	NC_000014.9 (22956950..22957029, complement)		(4)
**hsa-miR-4734**	fALS	Down	Microarray Meta-analysis	Serum	NC_000017.11 (38702262..38702331, complement)		(4)
**hsa-miR-4734**	sALS	Down	Microarray Meta-analysis	Serum	NC_000017.11 (38702262..38702331, complement)		(4)
**hsa-miR-4741**	fALS	Down	Microarray Meta-analysis	Serum	NC_000018.10 (22933349..22933438)		(4)
**hsa-miR-4741**	sALS	Down	Microarray Meta-analysis	Serum	NC_000018.10 (22933349..22933438)		(4)
**hsa-miR-4745-5p**	fALS	Down	Microarray Meta-analysis	Serum	NC_000019.10 (804940..805001)		(4)
**hsa-miR-4745-5p**	fALS	Down	Microarray & qPCR	Serum	NC_000019.10 (804940..805001)		(44)

**hsa-miR-4745-5p**	sALS	Down	Microarray Meta-analysis	Serum	NC_000019.10 (804940..805001)		(4)
**hsa-miR-4745-5p**	sALS	Down	Microarray & qPCR	Serum	NC_000019.10 (804940..805001)		(44)
**hsa-miR-4763-3p**	fALS	Down	Microarray Meta-analysis	Serum	NC_000022.11 (46113566..46113657)		(4)
**hsa-miR-4763-3p**	sALS	Down	Microarray Meta-analysis	Serum	NC_000022.11 (46113566..46113657)		(4)
**hsa-miR-4787-5p**	fALS	Down	Microarray Meta-analysis	Serum	NC_000003.12 (50675080..50675163)		(4)
**hsa-miR-4787-5p**	sALS	Down	Microarray Meta-analysis	Serum	NC_000003.12 (50675080..50675163)		(4)
**hsa-miR-483-3p**	AD	Down	NGS & qPCR	Serum	NC_000011.10 (2134134..2134209, complement)		(10)
**hsa-miR-483-5p**	AD	Up	Exiqon qPCR panel & qPCR	Plasma	NC_000011.10 (2134134..2134209, complement)		(18)
**hsa-miR-484**	AD	Up	TLDA	CSF	NC_000016.10 (15643294..15643372)		(31)
**hsa-miR-485-5p**	AD	Up	TLDA	CSF exosomes	NC_000014.9 (101055419..101055491)		(14)
**hsa-miR-485-5p**	PD	Up	NGS	CSF	NC_000014.9 (101055419..101055491)		(13)
**hsa-miR-485-5p**	PD	Up	TLDA	CSF exosomes	NC_000014.9 (101055419..101055491)		(14)
**hsa-miR-486-5p**	AD	Up	Exiqon qPCR panel & qPCR	Plasma	NC_000001.11 (177029363..177029445, complement)		(18)
**hsa-miR-486-5p**	MS	Up	Exiqon qPCR panel & qPCR	Serum	NC_000001.11 (177029363..177029445, complement)		(6)
**hsa-miR-488-3p**	AD	Down	NGS	CSF	NC_000001.11 (177029363..177029445, complement)		(13)

**hsa-miR-494**	MS	Up	Microarray	PBMCs	NC_000014.9 (101029634..101029714)		(29)
**hsa-miR-495**	AD	Down	NGS	CSF	NC_000014.9 (101033755..101033836)		(13)
**hsa-miR-496**	fALS	Down	Exiqon qPCR panel & qPCR	Serum	NC_000014.9 (101060573..101060674)		(7)
**hsa-miR-496**	sALS	Down	Exiqon qPCR panel & qPCR	Serum	NC_000014.9 (101060573..101060674)		(7)
**hsa-miR-5000-5p**	AD	Down	Microarray & qPCR	PBMCs	NC_000002.12 (75090812..75090914)		(35)
**hsa-miR-5010-3p**	AD	Up	NGS & qPCR	Blood	NC_000017.11 (42514188..42514307)		(1)
**hsa-miR-501-3p**	AD	Down	NGS & qPCR	Serum	NC_000023.11 (50009722..50009805)		(71)
**hsa-miR-502-3p**	AD	Up	Exiqon qPCR panel & qPCR	Plasma	NC_000023.11 (50009722..50009805)		(18)
**hsa-miR-505**	PD	Down	Microarray & qPCR	Plasma	NC_000023.11 (139924148..139924231, complement)		(55)
**hsa-miR-505-3p**	AD	Up	OpenArray qPCR	CSF	NC_000023.11 (139924148..139924231, complement)		(16)
**hsa-miR-505-3p**	PD	Up	Microarray & qPCR	Plasma	NC_000023.11 (139924148..139924231, complement)		(55)
**hsa-miR-519b-3p**	AD	Up	TLDA	CSF	NC_000019.10 (53695213..53695293)		(31)
**hsa-miR-520b**	AD	Up	TLDA	CSF	NC_000019.10 (53701227..53701287)		(31)
**hsa-miR-532-5p**	AD	Down	NGS & qPCR	Blood	NC_000023.11 (50003148..50003238)		(1)
**hsa-miR-532-5p**	AD	Down	TLDA	CSF	NC_000023.11 (50003148..50003238)		(31)
**hsa-miR-532-5p**	MS	Down	NGS & ddPCR	Serum exosomes	NC_000023.11 (50003148..50003238)		(21)

**hsa-miR-542-3p**	PD	Up	qPCR	CSF	NC_000023.11 (134541341..134541437, complement)		(46)
**hsa-miR-545-3p**	AD	Down	qPCR	Plasma	NC_000023.11 (74287104..74287209, complement)		(9)
**hsa-miR-5699**	AD	Down	Microarray & qPCR	PBMCs	NC_000010.11 (641689..641778, complement)		(35)
**hsa-miR-572**	MS	Down	qPCR	Serum	NC_000004.12 (11368827..11368921)		(72)
**hsa-miR-574-5p**	sALS	Up	qPCR	CSF	NC_000004.12 (38868032..38868127)		(5)
**hsa-miR-582-5p**	AD	Up	NGS & qPCR	Serum exosomes	NC_000005.10 (59703606..59703703, complement)		(17)
**hsa-miR-583**	sALS	Down	Microarray & qPCR	Leukocytes	NC_000005.10 (96079138..96079212)		(22)
**hsa-miR-584-5p**	AD	Down	TLDA	CSF	NC_000005.10 (149062313..149062409, complement)		(31)
**hsa-miR-584-5p**	fALS	Up	Microarray meta-analysis	Serum	NC_000005.10 (149062313..149062409, complement)		(4)
**hsa-miR-584-5p**	sALS	Up	Microarray meta-analysis	Serum	NC_000005.10 (149062313..149062409, complement)		(4)
**hsa-miR-590-5p**	AD	Down	TLDA	CSF	NC_000007.14 (74191198..74191294)		(31)
**hsa-miR-590-5p**	AD	Up	qPCR	CSF	NC_000007.14 (74191198..74191294)		(15)
**hsa-miR-597-5p**	AD	Up	TLDA	CSF	NC_000008.11 (9741672..9741768)		(31)
**hsa-miR-598**	AD	Down	NGS	CSF	NC_000008.11 (11035206..11035302, complement)		(13)
**hsa-miR-603**	AD	Up	TLDA	CSF	NC_000010.11 (24275685..24275781)		(31)
**hsa-miR-605-5p**	AD	Down	Exiqon panel & qPCR	CSF	NC_000010.11 (51299573..51299655)		(30)

**hsa-miR-6119-5p**	AD	Up	NGS & qPCR	Serum			(20)
**hsa-miR-626**	PD	Down	Microarray & qPCR	Plasma	NC_000015.10 (41691585..41691678)		(55)
**hsa-miR-638**	fALS	Down	Microarray meta-analysis	Serum	NC_000019.10 (10718404..10718503)		(4)
**hsa-miR-638**	sALS	Down	Microarray & qPCR	Leukocytes	NC_000019.10 (10718404..10718503)		(22)
**hsa-miR-638**	sALS	Down	Microarray meta-analysis	Serum	NC_000019.10 (10718404..10718503)		(4)
**hsa-miR-648a**	MS	Up	qPCR	Plasma	NC_000022.11 (17980868..17980961, complement)		(2)
**hsa-miR-652-3p**	fALS	Up	Microarray Meta-analysis	Serum	NC_000023.11 (110055329..110055426)		(4)
**hsa-miR-652-3p**	sALS	Up	Microarray Meta-analysis	Serum	NC_000023.11 (110055329..110055426)		(4)
**hsa-miR-665**	sALS	Down	Microarray & qPCR	Leukocytes	NC_000014.9 (100875033..100875104)		(22)
**hsa-miR-671-3p**	AD	Up	NGS	Serum	NC_000007.14 (151238421..151238538)		(13)
**hsa-miR-671-5p**	PD	Up	NGS	PBMCs	NC_000007.14 (151238421..151238538)		(33)
**hsa-miR-708**	AD	Down	OpenArray qPCR	CSF	NC_000011.10 (79402022..79402109, complement)		(16)
**hsa-miR-708-3p**	AD	Down	NGS	CSF	NC_000011.10 (79402022..79402109, complement)		(13)
**hsa-miR-708-5p**	AD	Down	NGS	CSF	NC_000011.10 (79402022..79402109, complement)		(13)
**hsa-miR-7-1-3p**	AD	Down	NGS, microarray & qPCR	Plasma	NC_000009.12 (83969748..83969857, complement)		(54)
**hsa-miR-744-5p**	fALS	Up	Microarray Meta-analysis	Serum	NC_000017.11 (12081899..12081996)		(4)

**hsa-miR-744-5p**	sALS	Up	Microarray Meta-analysis	Serum	NC_000017.11 (12081899..12081996)		(4)
**hsa-miR-760**	AD	Down	NGS	CSF	NC_000001.11 (93846832..93846911)		(13)
**hsa-miR-760**	fALS	Up	Microarray Meta-analysis	Serum	NC_000001.11 (93846832..93846911)		(4)
**hsa-miR-760**	sALS	Up	Microarray Meta-analysis	Serum	NC_000001.11 (93846832..93846911)		(4)
**hsa-miR-766**	AD	Up	OpenArray qPCR	CSF	NC_000023.11 (119646738..119646848, complement)		(16)
**hsa-miR-769-5p**	AD	Down	NGS	CSF	NC_000019.10 (46018932..46019049)		(13)
**hsa-miR-769-5p**	PD	Down	NGS	PBMCs	NC_000019.10 (46018932..46019049)		(33)
**hsa-miR-873-3p**	AD	Up	NGS	Serum	NC_000009.12 (28888879..28888955, complement)		(13)
**hsa-miR-873-3p**	PD	Up	NGS	CSF	NC_000009.12 (28888879..28888955, complement)		(13)
**hsa-miR-873-5p**	AD	Down	NGS	CSF	NC_000009.12 (28888879..28888955, complement)		(13)
**hsa-miR-874**	AD	Down	NGS	CSF	NC_000005.10 (137647572..137647649, complement)		(13)
**hsa-miR-885-5p**	AD	Down	NGS & qPCR	Serum	NC_000003.12 (10394489..10394562, complement)		(10)
**hsa-miR-887**	AD	Up	NGS	Serum	NC_000007.14 (129770383..129770492, complement)		(13)
**hsa-miR-9**	AD	Down	qPCR	Serum	NC_000001.11 (156420341..156420429, complement)	NC_000005.10 (88666853..88666939, complement)	(38)
**hsa-miR-92a**	MS	Up	qPCR	Plasma	NC_000013.11 (91351314..91351391)		(2)

**hsa-miR-92b-3p**	PD	Down	NGS	PBMCs	NC_000001.11 (155195177..155195272)		(33)
**hsa-miR-92c**	MS	Up	Microarray	PBMCs			(29)
**hsa-miR-93-5p**	AD	Up	NGS & qPCR	Serum exosomes	NC_000007.14 (100093768..100093847, complement)		(17)
**hsa-miR-9-3p**	AD	Down	NGS	CSF	NC_000001.11 (156420341..156420429, complement)		(13)
**hsa-miR-95**	AD	Down	NGS	CSF	NC_000004.12 (8005301..8005381, complement)		(13)
**hsa-miR-9-5p**	AD	Down	NGS	CSF	NC_000001.11 (156420341..156420429, complement)		(13)
**hsa-miR-98-5p**	AD	Down	NGS & qPCR	Serum	NC_000023.11 (53556223..53556341, complement)		(10)
**hsa-miR-99b-3p**	fALS	Up	Microarray Meta-analysis	Serum	NC_000019.10 (51692612..51692681)		(4)
**hsa-miR-99b-3p**	sALS	Up	Microarray Meta-analysis	Serum	NC_000019.10 (51692612..51692681)		(4)

1. Leidinger P, *et al.* (2013) A blood based 12-miRNA signature of Alzheimer disease patients. *Genome Biology* 14(7):R78-R78.
2. Kacperska MJ, *et al.* (2015) Selected Extracellular microRNA as Potential Biomarkers of Multiple Sclerosis Activity—Preliminary Study. *Journal of Molecular Neuroscience* 56(1):154-163.
3. Chen L, *et al.* (2018) Identification of aberrant circulating miRNAs in Parkinson's disease plasma samples. *Brain and Behavior* 8(4):e00941.
4. Taguchi Yh & Wang H (2018) Exploring microRNA Biomarker for Amyotrophic Lateral Sclerosis. *International Journal of Molecular Sciences* 19(5):1318.
5. Freischmidt A, Müller K, Ludolph AC, & Weishaupt JH (2013) Systemic dysregulation of TDP-43 binding microRNAs in amyotrophic lateral sclerosis. *Acta Neuropathologica Communications* 1:42-42.
6. Regev K, *et al.* (2016) Comprehensive evaluation of serum microRNAs as biomarkers in multiple sclerosis. *Neurology® Neuroimmunology & Neuroinflammation* 3(5):e267.
7. Raheja R, *et al.* (2018) Correlating serum micrornas and clinical parameters in amyotrophic lateral sclerosis. *Muscle & Nerve* 0(0).
8. Kumar P, *et al.* (2013) Circulating miRNA biomarkers for Alzheimer's disease. *PLoS One* 8(7):e69807.

9. Cosin-Tomas M*, et al.* (2017) Plasma miR-34a-5p and miR-545-3p as Early Biomarkers of Alzheimer's Disease: Potential and Limitations. *Molecular neurobiology* 54(7):5550-5562.
10. Tan L*, et al.* (2014) Genome-wide serum microRNA expression profiling identifies serum biomarkers for Alzheimer's disease. *J Alzheimers Dis* 40(4):1017-1027.
11. dos Santos MCT*, et al.* (2018) miRNA-based signatures in cerebrospinal fluid as potential diagnostic tools for early stage Parkinson's disease. *Oncotarget* 9(25):17455-17465.
12. Jiang N*, et al.* (2018) Meta-microRNAs as potential noninvasive markers for early diagnosis of Alzheimer's disease. *bioRxiv*.
13. Burgos K*, et al.* (2014) Profiles of Extracellular miRNA in Cerebrospinal Fluid and Serum from Patients with Alzheimer's and Parkinson's Diseases Correlate with Disease Status and Features of Pathology. *PLOS ONE* 9(5):e94839.
14. Gui Y, Liu H, Zhang L, Lv W, & Hu X (2015) Altered microRNA profiles in cerebrospinal fluid exosome in Parkinson disease and Alzheimer disease. *Oncotarget* 6(35):37043-37053.
15. Sørensen SS, Nygaard A-B, & Christensen T (2016) miRNA expression profiles in cerebrospinal fluid and blood of patients with Alzheimer's disease and other types of dementia – an exploratory study. *Translational Neurodegeneration* 5(1):6.
16. Denk J*, et al.* (2015) MicroRNA Profiling of CSF Reveals Potential Biomarkers to Detect Alzheimer`s Disease. *PLOS ONE* 10(5):e0126423.
17. Cheng L*, et al.* (2015) Prognostic serum miRNA biomarkers associated with Alzheimer's disease shows concordance with neuropsychological and neuroimaging assessment. *Molecular psychiatry* 20(10):1188-1196.
18. Nagaraj S*, et al.* (2017) Profile of 6 microRNA in blood plasma distinguish early stage Alzheimer's disease patients from non-demented subjects. *Oncotarget* 8(10):16122-16143.
19. Serafin A*, et al.* (2015) Overexpression of blood microRNAs 103a, 30b, and 29a in L-dopa-treated patients with PD. *Neurology* 84(7):645-653.
20. Guo R*, et al.* (2017) A 9-microRNA Signature in Serum Serves as a Noninvasive Biomarker in Early Diagnosis of Alzheimer's Disease. *J Alzheimers Dis* 60(4):1365-1377.
21. Selmaj I*, et al.* (2017) Global exosome transcriptome profiling reveals biomarkers for multiple sclerosis. *Ann Neurol* 81(5):703-717.
22. De Felice B*, et al.* (2012) A miRNA signature in leukocytes from sporadic amyotrophic lateral sclerosis. *Gene* 508(1):35-40.
23. Freischmidt A*, et al.* (2015) Serum microRNAs in sporadic amyotrophic lateral sclerosis. *Neurobiol Aging* 36(9):2660.e2615-2620.
24. Li Y*, et al.* (2017) Novel and functional ATG12 gene variants in sporadic Parkinson's disease. *Neuroscience letters* 643:22-26.
25. Yang D*, et al.* (2014) MicroRNA Expression Aberration in Chinese Patients with Relapsing Remitting Multiple Sclerosis. *Journal of Molecular Neuroscience* 52(1):131-137.
26. Dangla-Valls A*, et al.* (2017) CSF microRNA Profiling in Alzheimer's Disease: a Screening and Validation Study. *Molecular neurobiology* 54(9):6647-6654.
27. Kiko T*, et al.* (2014) MicroRNAs in plasma and cerebrospinal fluid as potential markers for Alzheimer's disease. *J Alzheimers Dis* 39(2):253-259.
28. Galimberti D*, et al.* (2014) Circulating miRNAs as potential biomarkers in Alzheimer's disease. *J Alzheimers Dis* 42(4):1261-1267.
29. Sheng WH*, et al.* (2015) Identifying the biomarkers of multiple sclerosis based on non-coding RNA signature. *European review for medical and pharmacological sciences* 19(19):3635-3642.
30. McKeever PM*, et al.* (2018) MicroRNA Expression Levels Are Altered in the Cerebrospinal Fluid of Patients with Young-Onset Alzheimer's Disease. *Molecular neurobiology*.
31. Lusardi TA*, et al.* (2017) MicroRNAs in Human Cerebrospinal Fluid as Biomarkers for Alzheimer's Disease. *Journal of Alzheimer's disease : JAD* 55(3):1223-1233.
32. Ebrahimkhani S*, et al.* (2017) Exosomal microRNA signatures in multiple sclerosis reflect disease status. *Scientific Reports* 7(1):14293.

33. Pasinetti GM (2012) Role of Personalized Medicine in the Identification and Characterization of Parkinson's Disease in Asymptomatic Subjects. *Journal of Alzheimer's Disease and Parkinsonism* 2(3):e118.
34. Vistbakka J, Elovaara I, Lehtimaki T, & Hagman S (2017) Circulating microRNAs as biomarkers in progressive multiple sclerosis. *Multiple sclerosis (Houndmills, Basingstoke, England)* 23(3):403-412.
35. Ren RJ*, et al.* (2016) Peripheral Blood MicroRNA Expression Profiles in Alzheimer's Disease: Screening, Validation, Association with Clinical Phenotype and Implications for Molecular Mechanism. *Molecular neurobiology* 53(8):5772-5781.
36. Zhang X*, et al.* (2017) Reduced Circulating Levels of miR-433 and miR-133b Are Potential Biomarkers for Parkinson’s Disease. *Frontiers in Cellular Neuroscience* 11:170.
37. Yang TT, Liu CG, Gao SC, Zhang Y, & Wang PC (2018) The Serum Exosome Derived MicroRNA-135a, -193b, and -384 Were Potential Alzheimer's Disease Biomarkers. *Biomedical and environmental sciences : BES* 31(2):87-96.
38. Geekiyanage H, Jicha GA, Nelson PT, & Chan C (2012) Blood serum miRNA: non-invasive biomarkers for Alzheimer's disease. *Experimental neurology* 235(2):491-496.
39. Ehya F, Abdul Tehrani H, Garshasbi M, & Nabavi SM (2017) Identification of miR-24 and miR-137 as novel candidate multiple sclerosis miRNA biomarkers using multi-staged data analysis protocol. *Molecular Biology Research Communications* 6(3):127-140.
40. Margis R, Margis R, & Rieder CR (2011) Identification of blood microRNAs associated to Parkinsonis disease. *Journal of biotechnology* 152(3):96-101.
41. Dong H*, et al.* (2016) A panel of four decreased serum microRNAs as a novel biomarker for early Parkinson's disease. *Biomarkers : biochemical indicators of exposure, response, and susceptibility to chemicals* 21(2):129-137.
42. Lugli G*, et al.* (2015) Plasma Exosomal miRNAs in Persons with and without Alzheimer Disease: Altered Expression and Prospects for Biomarkers. *PLoS One* 10(10):e0139233.
43. Ma X*, et al.* (2014) Expression, Regulation and Function of MicroRNAs in Multiple Sclerosis. *International Journal of Medical Sciences* 11(8):810-818.
44. Freischmidt A*, et al.* (2014) Serum microRNAs in patients with genetic amyotrophic lateral sclerosis and pre-manifest mutation carriers. *Brain : a journal of neurology* 137(Pt 11):2938-2950.
45. Waller R*, et al.* (2017) Serum miRNAs miR-206, 143-3p and 374b-5p as potential biomarkers for amyotrophic lateral sclerosis (ALS). *Neurobiology of Aging* 55:123-131.
46. Mo M*, et al.* (2017) MicroRNA expressing profiles in A53T mutant alpha-synuclein transgenic mice and Parkinsonian. *Oncotarget* 8(1):15-28.
47. Mansourian N, Rahaie M, & Hosseini M (2017) A Nanobiosensor Based on Fluorescent DNA-Hosted Silver Nanocluster and HCR Amplification for Detection of MicroRNA Involved in Progression of Multiple Sclerosis. *Journal of fluorescence* 27(5):1679-1685.
48. Ma W*, et al.* (2016) Serum miR-221 serves as a biomarker for Parkinson's disease. *Cell biochemistry and function* 34(7):511-515.
49. Vallelunga A*, et al.* (2014) Identification of circulating microRNAs for the differential diagnosis of Parkinson's disease and Multiple System Atrophy. *Frontiers in Cellular Neuroscience* 8(156).
50. Bergman P*, et al.* (2016) Circulating miR-150 in CSF is a novel candidate biomarker for multiple sclerosis. *Neurology(R) neuroimmunology & neuroinflammation* 3(3):e219.
51. Fenoglio C*, et al.* (2016) Effect of fingolimod treatment on circulating miR-15b, miR23a and miR-223 levels in patients with multiple sclerosis. *Journal of Neuroimmunology* 299:81-83.
52. Ding H*, et al.* (2016) Identification of a panel of five serum miRNAs as a biomarker for Parkinson's disease. *Parkinsonism & related disorders* 22:68-73.
53. Benigni M*, et al.* (2016) Identification of miRNAs as Potential Biomarkers in Cerebrospinal Fluid from Amyotrophic Lateral Sclerosis Patients. *Neuromolecular medicine* 18(4):551-560.

54. Keller A*, et al.* (2014) Comprehensive analysis of microRNA profiles in multiple sclerosis including next-generation sequencing. *Multiple sclerosis (Houndmills, Basingstoke, England)* 20(3):295-303.
55. Khoo SK*, et al.* (2012) Plasma-based circulating MicroRNA biomarkers for Parkinson's disease. *Journal of Parkinson's disease* 2(4):321-331.
56. Chen Y*, et al.* (2016) Aberration of miRNAs Expression in Leukocytes from Sporadic Amyotrophic Lateral Sclerosis. *Frontiers in Molecular Neuroscience* 9:69.
57. Cao XY*, et al.* (2017) MicroRNA biomarkers of Parkinson's disease in serum exosome-like microvesicles. *Neuroscience letters* 644:94-99.
58. Botta-Orfila T*, et al.* (2014) Identification of blood serum micro-RNAs associated with idiopathic and LRRK2 Parkinson's disease. *Journal of neuroscience research* 92(8):1071-1077.
59. Marques TM*, et al.* (2017) MicroRNAs in Cerebrospinal Fluid as Potential Biomarkers for Parkinson's Disease and Multiple System Atrophy. *Molecular neurobiology* 54(10):7736-7745.
60. Toivonen JM*, et al.* (2014) MicroRNA-206: a potential circulating biomarker candidate for amyotrophic lateral sclerosis. *PLoS One* 9(2):e89065.
61. Bruinsma IB*, et al.* (2017) Regulator of oligodendrocyte maturation, miR-219, a potential biomarker for MS. *Journal of neuroinflammation* 14(1):235.
62. Sala Frigerio C*, et al.* (2013) Reduced expression of hsa-miR-27a-3p in CSF of patients with Alzheimer disease. *Neurology* 81(24):2103-2106.
63. Muller M*, et al.* (2016) MicroRNA-29a Is a Candidate Biomarker for Alzheimer's Disease in Cell-Free Cerebrospinal Fluid. *Molecular neurobiology* 53(5):2894-2899.
64. Bai X*, et al.* (2017) Downregulation of blood serum microRNA 29 family in patients with Parkinson’s disease. *Scientific Reports* 7:5411.
65. Schwienbacher C*, et al.* (2017) Plasma and White Blood Cells Show Different miRNA Expression Profiles in Parkinson's Disease. *Journal of molecular neuroscience : MN* 62(2):244-254.
66. Cardo LF*, et al.* (2013) Profile of microRNAs in the plasma of Parkinson's disease patients and healthy controls. *Journal of neurology* 260(5):1420-1422.
67. Takahashi I*, et al.* (2015) Identification of plasma microRNAs as a biomarker of sporadic Amyotrophic Lateral Sclerosis. *Molecular Brain* 8:67.
68. Kumar S, Vijayan M, & Reddy PH (2017) MicroRNA-455-3p as a potential peripheral biomarker for Alzheimer's disease. *Human molecular genetics* 26(19):3808-3822.
69. Kumar S & Reddy PH (2018) MicroRNA-455-3p as a Potential Biomarker for Alzheimer's Disease: An Update. *Frontiers in Aging Neuroscience* 10:41.
70. Chen Y*, et al.* (2017) MicroRNA-4639 Is a Regulator of DJ-1 Expression and a Potential Early Diagnostic Marker for Parkinson’s Disease. *Frontiers in Aging Neuroscience* 9(232).
71. Hara N*, et al.* (2017) Serum microRNA miR-501-3p as a potential biomarker related to the progression of Alzheimer’s disease. *Acta Neuropathologica Communications* 5(1):10.
72. Mancuso R*, et al.* (2015) MicroRNA-572 expression in multiple sclerosis patients with different patterns of clinical progression. *Journal of Translational Medicine* 13:148.

Panoramic visualization of circulating microRNAs across neurodegenerative diseases in humans, Acta Neuropathologica, Samuel Brennan, Matthew Keon, Bing Liu, Zheng Su, Nitin Saksena, Neurodegenerative Disease section, Iggy Get Out, 19a Boundary Street, Darlinghurst NSW 2010. Sydney. Australia. E-mail: nitin@iggygetout.com
