# Supplementary material for: Panoramic Visualization of Circulating MicroRNAs Across Neurodegenerative Diseases in Humans
Source: Mol Neurobiol. 2019 Apr 29;56(11):7380–407. doi: 10.1007/s12035-019-1615-1 (PMC6815273; doi:10.1007/s12035-019-1615-1)
Supplement: Supplementary file 6 — (PDF 242 kb) [file 12035_2019_1615_MOESM6_ESM.pdf]

miRNA Cluster	Species	UCSC Acc.	Xsome	Strand	MirClust Coordinate	UCSC Genome	Length
mir-18 mir-19 mir-20 mir-92 mir-106 mir-363	*Homo sapiens*	hg19	chrX	-	133303408-133304308	BED	901
mir-17 mir-18 mir-19 mir-20 mir-92	*Homo sapiens*	hg19	chr13	+	92002859-92003645	BED	787
mir-323 mir-329 mir-380 mir-758 mir-1197	*Homo sapiens*	hg19	chr14	+	101491354-101493520	BED	2167
mir-18 mir-19 mir-20 mir-92 mir-106	*Homo sapiens*	hg19	chrX	-	133303408-133304308	BED	901
mir-18 mir-19 mir-92 mir-106 mir-363	*Homo sapiens*	hg19	chrX	-	133303408-133304308	BED	901
mir-18 mir-19 mir-20 mir-106 mir-363	*Homo sapiens*	hg19	chrX	-	133303408-133304308	BED	901
mir-18 mir-19 mir-20 mir-92	*Homo sapiens*	hg19	chr13	+	92002859-92003645	BED	787
mir-18 mir-19 mir-20 mir-92	*Homo sapiens*	hg19	chrX	-	133303408-133304308	BED	901
mir-17 mir-19 mir-20 mir-92	*Homo sapiens*	hg19	chr13	+	92002859-92003645	BED	787
mir-17 mir-18 mir-19 mir-92	*Homo sapiens*	hg19	chr13	+	92002859-92003645	BED	787
mir-17 mir-18 mir-19 mir-20	*Homo sapiens*	hg19	chr13	+	92002859-92003645	BED	787
mir-323 mir-329 mir-380 mir-758	*Homo sapiens*	hg19	chr14	+	101491354-101493520	BED	2167
mir-323 mir-380 mir-758 mir-1197	*Homo sapiens*	hg19	chr14	+	101491354-101493520	BED	2167
mir-323 mir-329 mir-758 mir-1197	*Homo sapiens*	hg19	chr14	+	101491354-101493520	BED	2167
mir-18 mir-19 mir-92 mir-106	*Homo sapiens*	hg19	chrX	-	133303408-133304308	BED	901
mir-18 mir-19 mir-20 mir-106	*Homo sapiens*	hg19	chrX	-	133303408-133304308	BED	901
mir-18 mir-19 mir-20 mir-363	*Homo sapiens*	hg19	chrX	-	133303408-133304308	BED	901
mir-18 mir-19 mir-106 mir-363	*Homo sapiens*	hg19	chrX	-	133303408-133304308	BED	901
mir-19 mir-20 mir-92 mir-363	*Homo sapiens*	hg19	chrX	-	133303408-133304308	BED	901

mir-20 mir-92 mir-106 mir-363	*Homo sapiens*	hg19	chrX	-	133303408-133304308	BED	901
mir-369 mir-409 mir-410 mir-656	*Homo sapiens*	hg19	chr14	+	101530832-101533138	BED	2307
mir-134 mir-382 mir-485 mir-668	*Homo sapiens*	hg19	chr14	+	101520643-101522637	BED	1995
mir-381 mir-487 mir-539 mir-889	*Homo sapiens*	hg19	chr14	+	101512257-101515983	BED	3727
mir-369 mir-409 mir-410 mir-541	*Homo sapiens*	hg19	chr14	+	101530832-101533138	BED	2307
mir-369 mir-409 mir-410 mir-412	*Homo sapiens*	hg19	chr14	+	101530832-101533138	BED	2307
mir-369 mir-409 mir-541 mir-656	*Homo sapiens*	hg19	chr14	+	101530832-101533138	BED	2307
mir-19 mir-20 mir-92	*Homo sapiens*	hg19	chr13	+	92002859-92003645	BED	787
mir-19 mir-20 mir-92	*Homo sapiens*	hg19	chrX	-	133303408-133304308	BED	901
mir-23 mir-24 mir-27	*Homo sapiens*	hg19	chr19	-	13947101-13947473	BED	373
mir-23 mir-24 mir-27	*Homo sapiens*	hg19	chr9	+	97847490-97848370	BED	881
mir-18 mir-19 mir-92	*Homo sapiens*	hg19	chr13	+	92002859-92003645	BED	787
mir-18 mir-19 mir-92	*Homo sapiens*	hg19	chrX	-	133303408-133304308	BED	901
mir-18 mir-19 mir-20	*Homo sapiens*	hg19	chr13	+	92002859-92003645	BED	787
mir-18 mir-19 mir-20	*Homo sapiens*	hg19	chrX	-	133303408-133304308	BED	901
mir-17 mir-19 mir-92	*Homo sapiens*	hg19	chr13	+	92002859-92003645	BED	787
mir-17 mir-19 mir-20	*Homo sapiens*	hg19	chr13	+	92002859-92003645	BED	787
mir-17 mir-20 mir-92	*Homo sapiens*	hg19	chr13	+	92002859-92003645	BED	787
mir-17 mir-18 mir-19	*Homo sapiens*	hg19	chr13	+	92002859-92003645	BED	787
mir-323 mir-329 mir-758	*Homo sapiens*	hg19	chr14	+	101491354-101493520	BED	2167
mir-323 mir-380 mir-758	*Homo sapiens*	hg19	chr14	+	101491354-101493520	BED	2167
mir-19 mir-20 mir-363	*Homo sapiens*	hg19	chrX	-	133303408-133304308	BED	901
mir-19 mir-92 mir-363	*Homo sapiens*	hg19	chrX	-	133303408-133304308	BED	901

mir-25 mir-93 mir-106	*Homo sapiens*	hg19	chr7	-	99691183-99691697	BED	515
mir-323 mir-758 mir-1197	*Homo sapiens*	hg19	chr14	+	101491354-101493520	BED	2167
mir-18 mir-19 mir-106	*Homo sapiens*	hg19	chrX	-	133303408-133304308	BED	901
mir-369 mir-409 mir-410	*Homo sapiens*	hg19	chr14	+	101530832-101533138	BED	2307
mir-18 mir-20 mir-106	*Homo sapiens*	hg19	chrX	-	133303408-133304308	BED	901
mir-20 mir-92 mir-363	*Homo sapiens*	hg19	chrX	-	133303408-133304308	BED	901
mir-381 mir-487 mir-539	*Homo sapiens*	hg19	chr14	+	101512257-101515983	BED	3727
mir-20 mir-92 mir-106	*Homo sapiens*	hg19	chrX	-	133303408-133304308	BED	901
mir-18 mir-19 mir-363	*Homo sapiens*	hg19	chrX	-	133303408-133304308	BED	901
mir-369 mir-409 mir-656	*Homo sapiens*	hg19	chr14	+	101530832-101533138	BED	2307
mir-369 mir-409 mir-412	*Homo sapiens*	hg19	chr14	+	101530832-101533138	BED	2307
mir-20 mir-106 mir-363	*Homo sapiens*	hg19	chrX	-	133303408-133304308	BED	901
mir-92 mir-106 mir-363	*Homo sapiens*	hg19	chrX	-	133303408-133304308	BED	901
mir-382 mir-485 mir-668	*Homo sapiens*	hg19	chr14	+	101520643-101522637	BED	1995
mir-369 mir-409 mir-541	*Homo sapiens*	hg19	chr14	+	101530832-101533138	BED	2307
let-7 mir-99 mir-125	*Homo sapiens*	hg19	chr19	+	52195865-52196592	BED	728
mir-23 mir-27	*Homo sapiens*	hg19	chr19	-	13947101-13947473	BED	373
mir-23 mir-27	*Homo sapiens*	hg19	chr9	+	97847490-97848370	BED	881
mir-19 mir-92	*Homo sapiens*	hg19	chr13	+	92002859-92003645	BED	787
mir-19 mir-92	*Homo sapiens*	hg19	chrX	-	133303408-133304308	BED	901
mir-19 mir-20	*Homo sapiens*	hg19	chr13	+	92002859-92003645	BED	787
mir-19 mir-20	*Homo sapiens*	hg19	chrX	-	133303408-133304308	BED	901
mir-20 mir-92	*Homo sapiens*	hg19	chr13	+	92002859-92003645	BED	787
mir-20 mir-92	*Homo sapiens*	hg19	chrX	-	133303408-133304308	BED	901
mir-18 mir-19	*Homo sapiens*	hg19	chr13	+	92002859-92003645	BED	787
mir-18 mir-19	*Homo sapiens*	hg19	chrX	-	133303408-133304308	BED	901
mir-24 mir-27	*Homo sapiens*	hg19	chr19	-	13947101-13947473	BED	373
mir-24 mir-27	*Homo sapiens*	hg19	chr9	+	97847490-97848370	BED	881

mir-15 mir-16	*Homo sapiens*	hg19	chr13	-	50623109-50623337	BED	229
mir-15 mir-16	*Homo sapiens*	hg19	chr3	+	160122376-160122613	BED	238
mir-18 mir-20	*Homo sapiens*	hg19	chr13	+	92002859-92003645	BED	787
mir-18 mir-20	*Homo sapiens*	hg19	chrX	-	133303408-133304308	BED	901
mir-17 mir-19	*Homo sapiens*	hg19	chr13	+	92002859-92003645	BED	787
mir-17 mir-92	*Homo sapiens*	hg19	chr13	+	92002859-92003645	BED	787
mir-17 mir-20	*Homo sapiens*	hg19	chr13	+	92002859-92003645	BED	787
let-7 mir-125	*Homo sapiens*	hg19	chr19	+	52195865-52196592	BED	728
mir-130 mir-301	*Homo sapiens*	hg19	chr22	+	22007270-22007674	BED	405
let-7 mir-99	*Homo sapiens*	hg19	chr19	+	52195865-52196592	BED	728
let-7 mir-99	*Homo sapiens*	hg19	chr21	+	17911409-17912231	BED	823
mir-144 mir-451	*Homo sapiens*	hg19	chr17	-	27188387-27188636	BED	250
mir-96 mir-183	*Homo sapiens*	hg19	chr7	-	129414532-129414854	BED	323
mir-221 mir-222	*Homo sapiens*	hg19	chrX	-	45605585-45606530	BED	946
mir-132 mir-212	*Homo sapiens*	hg19	chr17	-	1953202-1953674	BED	473
mir-302 mir-367	*Homo sapiens*	hg19	chr4	-	113569030-113569713	BED	684
mir-194 mir-215	*Homo sapiens*	hg19	chr1	-	220291195-220291583	BED	389
mir-25 mir-93	*Homo sapiens*	hg19	chr7	-	99691183-99691697	BED	515
mir-141 mir-200	*Homo sapiens*	hg19	chr12	+	7072862-7073354	BED	493
mir-299 mir-411	*Homo sapiens*	hg19	chr14	+	101489662-101490193	BED	532
mir-19 mir-363	*Homo sapiens*	hg19	chrX	-	133303408-133304308	BED	901
mir-93 mir-106	*Homo sapiens*	hg19	chr7	-	99691183-99691697	BED	515
mir-329 mir-758	*Homo sapiens*	hg19	chr14	+	101491354-101493520	BED	2167
mir-323 mir-758	*Homo sapiens*	hg19	chr14	+	101491354-101493520	BED	2167
mir-191 mir-425	*Homo sapiens*	hg19	chr3	-	49057581-49058142	BED	562
mir-369 mir-409	*Homo sapiens*	hg19	chr14	+	101530832-101533138	BED	2307
mir-20 mir-363	*Homo sapiens*	hg19	chrX	-	133303408-133304308	BED	901
mir-18 mir-106	*Homo sapiens*	hg19	chrX	-	133303408-133304308	BED	901

mir-192 mir-194	*Homo sapiens*	hg19	chr11	-	64658609-64658911	BED	303
mir-599 mir-875	*Homo sapiens*	hg19	chr8	-	100548864-100549089	BED	226
mir-92 mir-363	*Homo sapiens*	hg19	chrX	-	133303408-133304308	BED	901
mir-20 mir-106	*Homo sapiens*	hg19	chrX	-	133303408-133304308	BED	901
mir-19 mir-106	*Homo sapiens*	hg19	chrX	-	133303408-133304308	BED	901
mir-381 mir-487	*Homo sapiens*	hg19	chr14	+	101512257-101515983	BED	3727
mir-195 mir-497	*Homo sapiens*	hg19	chr17	-	6920934-6921341	BED	408
mir-380 mir-1197	*Homo sapiens*	hg19	chr14	+	101491354-101493520	BED	2167
mir-92 mir-106	*Homo sapiens*	hg19	chrX	-	133303408-133304308	BED	901
mir-106 mir-363	*Homo sapiens*	hg19	chrX	-	133303408-133304308	BED	901
mir-188 mir-532	*Homo sapiens*	hg19	chrX	+	49767754-49768194	BED	441
mir-485 mir-668	*Homo sapiens*	hg19	chr14	+	101520643-101522637	BED	1995
mir-494 mir-1193	*Homo sapiens*	hg19	chr14	+	101495971-101496466	BED	496
mir-134 mir-382	*Homo sapiens*	hg19	chr14	+	101520643-101522637	BED	1995
mir-376 mir-654	*Homo sapiens*	hg19	chr14	+	101505686-101507782	BED	2097
mir-154 mir-496	*Homo sapiens*	hg19	chr14	+	101526092-101527011	BED	920
mir-431 mir-433	*Homo sapiens*	hg19	chr14	+	101347344-101348315	BED	972
mir-450 mir-542	*Homo sapiens*	hg19	chrX	-	133674215-133675467	BED	1253
mir-424 mir-503	*Homo sapiens*	hg19	chrX	-	133680358-133680741	BED	384

Panoramic visualization of circulating microRNAs across neurodegenerative diseases in humans, Acta Neuropathologica, Samuel Brennan, Matthew Keon, Bing Liu, Zheng Su, Nitin Saksena, Neurodegenerative Disease section, Iggy Get Out, 19a Boundary Street, Darlinghurst NSW 2010. Sydney. Australia. E-mail: nitin@iggygetout.com
