# Supplementary material for: Panoramic Visualization of Circulating MicroRNAs Across Neurodegenerative Diseases in Humans
Source: Mol Neurobiol. 2019 Apr 29;56(11):7380–407. doi: 10.1007/s12035-019-1615-1 (PMC6815273; doi:10.1007/s12035-019-1615-1)
Supplement: Supplementary file 7 — (DOCX 36 kb) [file 12035_2019_1615_MOESM7_ESM.docx]

| miRNA | Condition | Expression | Analysis Method | Source | C’somal location | C’somal location -2 | Ref |
| --- | --- | --- | --- | --- | --- | --- | --- |
| hsa-miR-30a-3p | AD | Down | TLDA | CSF | NC_000006.12 (71403551..71403621, complement) |  | (1) |
| hsa-miR-30a-3p | PD | Down | NGS | Serum | NC_000006.12 (71403551..71403621, complement) |  | (2) |
| hsa-miR-30a-5p | PD | Up | Microarray & qPCR | Blood | NC_000006.12 (71403551..71403621, complement) |  | (3) |
| hsa-miR-30a-5p | PD | Up | qPCR | Plasma | NC_000006.12 (71403551..71403621, complement) |  | (4) |
| hsa-miR-30b-5p | AD | Down | Exiqon qPCR panel & qPCR | Plasma | NC_000008.11 (134800520..134800607, complement) |  | (5) |
| hsa-miR-30b-5p | fALS | Down | Exiqon qPCR panel & qPCR | Serum | NC_000008.11 (134800520..134800607, complement) |  | (6) |
| hsa-miR-30b-5p | MS | Down | NGS | Serum exosomes | NC_000008.11 (134800520..134800607, complement) |  | (7) |
| hsa-miR-30b-5p | PD | Up | qPCR | PBMCs | NC_000008.11 (134800520..134800607, complement) |  | (8) |
| hsa-miR-30b-5p | PD | Up | qPCR | Plasma | NC_000008.11 (134800520..134800607, complement) |  | (4) |
| hsa-miR-30b-5p | sALS | Down | Exiqon qPCR panel & qPCR | Serum | NC_000008.11 (134800520..134800607, complement) |  | (6) |
| hsa-miR-30c | MS | Up | Microarray | PBMCs | NC_000001.11 (40757284..40757372) | NC_000006.12 (71376960..71377031, complement) | (9) |
| hsa-miR-30c-2-3p | AD | Up | NGS | Serum | NC_000006.12 (71376960..71377031, complement) |  | (2) |
| hsa-miR-30c-5p | PD | Down | Microarray | Serum | NC_000001.11 (40757284..40757372) | NC_000006.12 (71376960..71377031, complement) | (10) |
| hsa-miR-30d-5p | AD | Down | TLDA | CSF | NC_000008.11 (134804876..134804945, complement) |  | (1) |
| hsa-miR-30e-5p | AD | Down | NGS & qPCR | Serum | NC_000001.11 (40754355..40754446) |  | (11) |
| hsa-miR-30e-5p | AD | Up | NGS & qPCR | Serum exosomes | NC_000001.11 (40754355..40754446) |  | (12) |
| hsa-miR-30e-5p | PD | Up | NGS | PBMCs | NC_000001.11 (40754355..40754446) |  | (13) |
| hsa-miR-30e-5p | PD | Down | NGS | Serum | NC_000001.11 (40754355..40754446) |  | (2) |

1. Lusardi TA*, et al.* (2017) MicroRNAs in Human Cerebrospinal Fluid as Biomarkers for Alzheimer’s Disease. *Journal of Alzheimer's disease : JAD* 55(3):1223-1233.

2. Burgos K*, et al.* (2014) Profiles of Extracellular miRNA in Cerebrospinal Fluid and Serum from Patients with Alzheimer's and Parkinson's Diseases Correlate with Disease Status and Features of Pathology. *PLOS ONE* 9(5):e94839.

3. Margis R, Margis R, & Rieder CR (2011) Identification of blood microRNAs associated to Parkinsonis disease. *Journal of biotechnology* 152(3):96-101.

4. Schwienbacher C*, et al.* (2017) Plasma and White Blood Cells Show Different miRNA Expression Profiles in Parkinson's Disease. *Journal of molecular neuroscience : MN* 62(2):244-254.

5. Nagaraj S*, et al.* (2017) Profile of 6 microRNA in blood plasma distinguish early stage Alzheimer's disease patients from non-demented subjects. *Oncotarget* 8(10):16122-16143.

6. Raheja R*, et al.* (2018) Correlating serum micrornas and clinical parameters in amyotrophic lateral sclerosis. *Muscle & Nerve* 0(0).

7. Ebrahimkhani S*, et al.* (2017) Exosomal microRNA signatures in multiple sclerosis reflect disease status. *Scientific Reports* 7(1):14293.

8. Serafin A*, et al.* (2015) Overexpression of blood microRNAs 103a, 30b, and 29a in L-dopa-treated patients with PD. *Neurology* 84(7):645-653.

9. Sheng WH*, et al.* (2015) Identifying the biomarkers of multiple sclerosis based on non-coding RNA signature. *European review for medical and pharmacological sciences* 19(19):3635-3642.

10. Vallelunga A*, et al.* (2014) Identification of circulating microRNAs for the differential diagnosis of Parkinson's disease and Multiple System Atrophy. *Frontiers in Cellular Neuroscience* 8(156).

11. Tan L*, et al.* (2014) Genome-wide serum microRNA expression profiling identifies serum biomarkers for Alzheimer's disease. *J Alzheimers Dis* 40(4):1017-1027.

12. Cheng L*, et al.* (2015) Prognostic serum miRNA biomarkers associated with Alzheimer's disease shows concordance with neuropsychological and neuroimaging assessment. *Molecular psychiatry* 20(10):1188-1196.

13. Pasinetti GM (2012) Role of Personalized Medicine in the Identification and Characterization of Parkinson's Disease in Asymptomatic Subjects. *Journal of Alzheimer's Disease and Parkinsonism* 2(3):e118.
